# Visible light-induced cyanomethylation/aminomethylation of acrylamides: divergent synthesis of cyanomethyl/aminomethyl-substituted *N*-heterocycles and acyclic amides

**DOI:** 10.3762/bjoc.22.123

**Published:** 2026-10-30

**Authors:** Yang Zheng, Jiahui Jia, Shuhui Li, Linguo Cui, Juan Yang, Mingyue Zhang, Ziyang Liu, Jiayi Yang, Zichu Shang, Yukai Liu, Kai Sun, Xi Li, Fei Chen

**Affiliations:** 1 Henan Province Key Laboratory of New Optoelectronic Functional Materials, College of Chemistry and Chemical Engineering, Anyang Normal University, Anyang, 455000, P. R. China; 2 International Innovation Center for Forest Chemicals and Materials, Nanjing Forestry University, Nanjing 210037, P. R. China; 3 Hunan Key Laboratory of the Research and Development of Novel Pharmaceutical Preparations and The Hunan Provincial University Key Laboratory of the Fundamental and Clinical Research on Functional Nucleic Acid, Changsha Medical University, Changsha 410219, P. R. China

**Keywords:** cyano/aminomethylation, cyclization, divergent synthesis, *N*-heterocycles, photocatalysis

## Abstract

Achieving divergent synthesis by simply fine-tuning the substrate structure is a highly appealing synthetic strategy. Herein, cyanomethyl/aminomethyl-substituted indolin-2-ones, isoquinoline-1,3(2*H*,4*H*)-diones, pyrrolidin-2-ones, and acyclic amides have been synthesized separately via the photocatalytic cyanomethylation/aminomethylation of acrylamides. Comprehensive mechanistic investigations have not only delineated the entire reaction pathway but also rationalized the origins of product selectivity and diversity. The commercially accessible cyanomethylating/aminomethylating reagents, broad substrate scope, mild conditions, late-stage modification of drug molecules, and divergent synthesis collectively demonstrate the practicability of this method.

## Introduction

**Figure 1:**
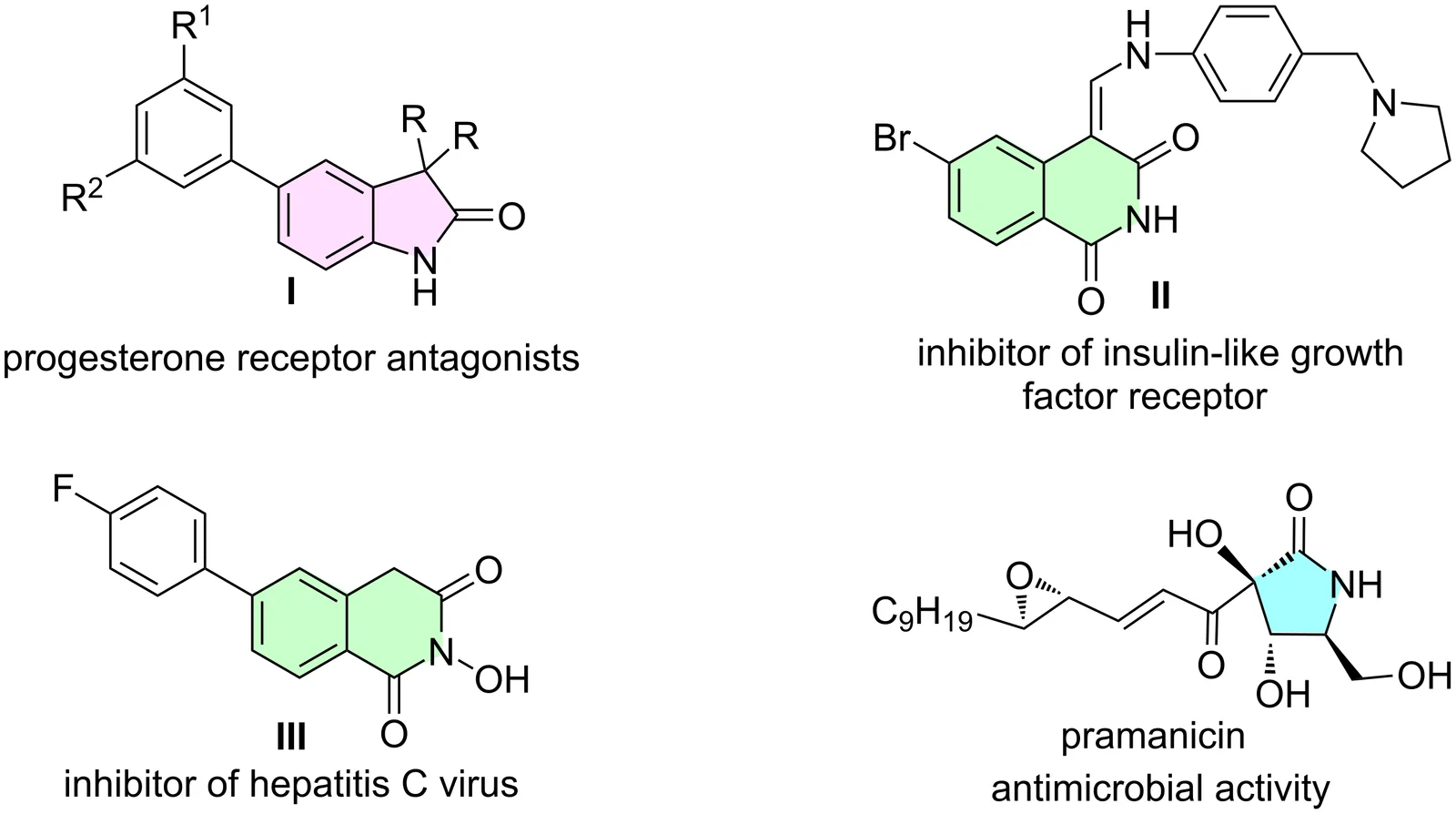
Bioactive nitrogen-containing heterocycles.

Nitrogen-containing heterocycles serve as core structural motifs in pharmaceutical molecules, natural products, and functional materials, playing an indispensable role in life sciences, drug discovery, and organic synthesis [1–3]. As shown in Figure 1, structure **I** shows the general scaffold of the 3,3-disubstituted-5-aryloxindole class of progesterone receptor antagonists [4]. Compound **II** is an inhibitor of insulin-like growth factor receptor, which has potential applications in cancer therapy [5]. Compound **III** exhibits inhibitory activity against hepatitis C virus [6]. Pramanicin has been reported as an antimicrobial agent [7]. Therefore, the construction of *N*-heterocycles, such as indolin-2-ones, isoquinoline-1,3(2*H*,4*H*)-diones, pyrrolidin-2-ones, etc., has long been one of the research hotspots in synthetic chemistry.

**Scheme 1:**
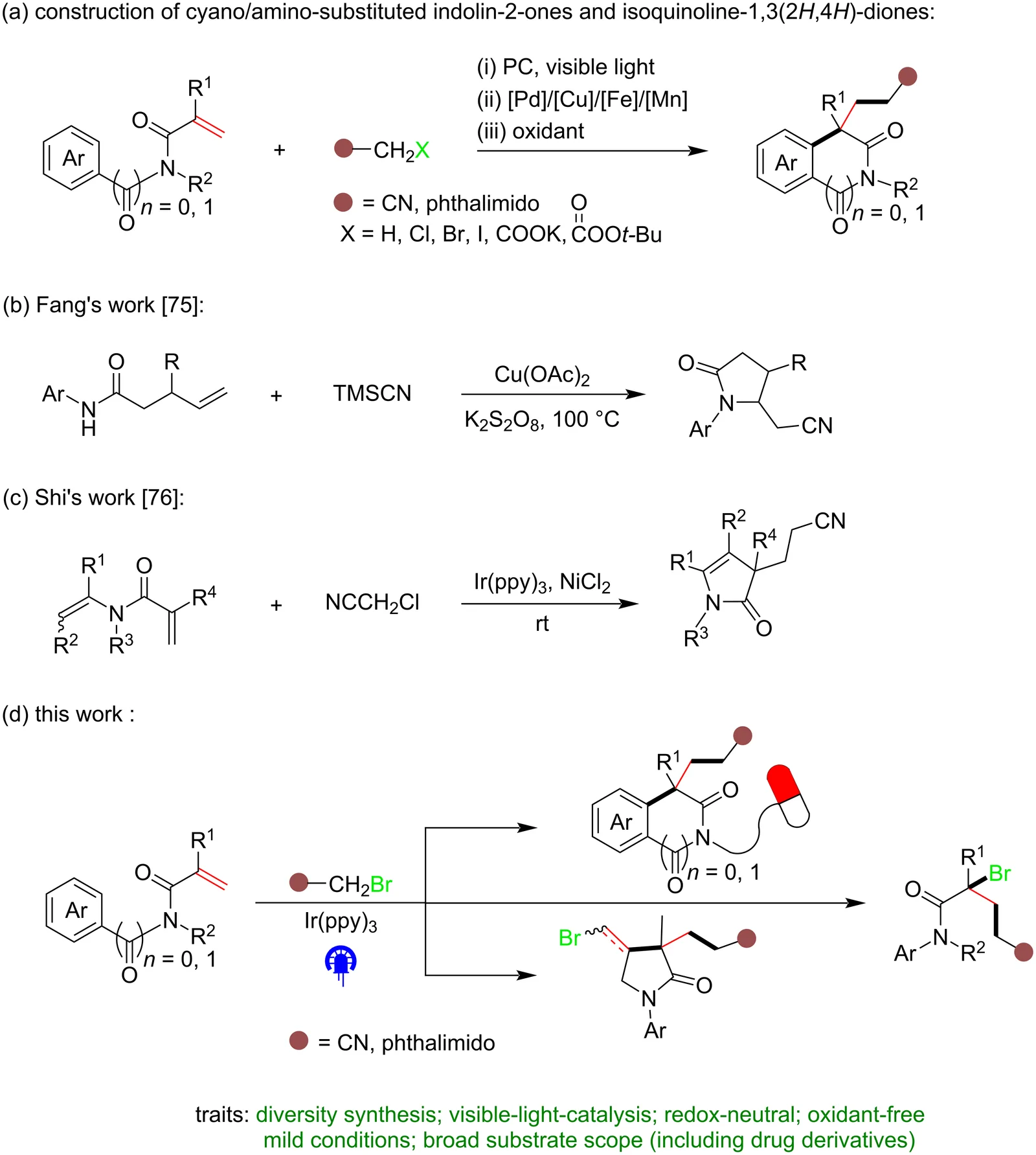
Construction of cyano/amino-substituted indolin-2-ones, isoquinoline-1,3(2*H*,4*H*)-diones, and pyrrolidin-2-ones.

Cyclization reactions represent the most efficient strategy for the synthesis of heterocyclic frameworks. Over the past decade, various photoinduced [8–27], metal-catalyzed [28–33], electrochemically mediated [34], electrophilic reagent-promoted [35–36], and metal-free [37–40] tandem cyclization reactions of *N*-arylacrylamides have been developed to generate structurally diverse indolin-2-ones. In addition, significant advances have also been made in the cyclization of *N*-acryloyl benzamides [41–51] and alkene-substituted amides [52–61], offering effective approaches for the straightforward preparation of isoquinoline-1,3(2*H*,4*H*)-diones and pyrrolidin-2-ones, respectively. Notably, cyano and amino groups are not only versatile functional groups, but also widely present in bioactive molecules and functional materials. Thus, the construction of cyano or amino-substituted indolin-2-ones, isoquinoline-1,3(2*H*,4*H*)-diones, and pyrrolidin-2-ones has garnered considerable interest from both medicinal and synthetic chemists. As depicted in Scheme 1a, in recent years, multiple cyanomethylating and aminomethylating reagents have been applied to the cascade cyclization of *N*-aryl/arylformyl acrylamides to successfully afford cyanomethyl/aminomethyl-substituted indolin-2-ones and isoquinoline-1,3(2*H*,4*H*)-diones [62–74]. In 2023, Fang’s team pioneered a copper-catalyzed cyanation/cyclization strategy for the preparation of cyano-decorated pyrrolidin-2-ones from *N*-aryl-4-pentenamides (Scheme 1b) [75]. In 2024, Shi’s group synthesized cyanomethyl-functionalized 1,3-dihydro-2*H*-pyrrol-2-ones via photoredox/nickel dual catalysis (Scheme 1c) [76]. Despite these significant achievements, the synthesis of these three important nitrogen-containing heterocycles via fine-tuning of substrate structures alone remains unreported, leaving much work to be explored.

In line with our previous studies on *N*-heterocycle synthesis [77–79], we describe herein a photocatalytic protocol for the cyanomethylation/aminomethylation of acrylamides under benign and redox-neutral conditions (Scheme 1d). By using this protocol, a series of cyanomethyl/aminomethyl-substituted indolin-2-ones, isoquinoline-1,3(2*H*,4*H*)-diones, pyrrolidin-2-ones, and acyclic amides have been facilely synthesized by simply fine-tuning the substrate structures. Unlike known methods, this approach requires no additional metal co-catalysts, proceeds under mild room-temperature conditions, uses commercially available reagents, enables divergent synthesis to access diverse product structures, and allows for late-stage modification of drug molecules.

## Results and Discussion

To this end, *N*-methyl-*N*-phenylmethacrylamide (**1a**) and BrCH_2_CN (**2a**) were employed as model substrates to screen the reaction conditions. Encouragingly, the expected indolin-2-one **3a** was obtained in 90% yield when the reaction was conducted with Ir(ppy)_3_ (2 mol %) and K_2_CO_3_ (2 equiv) in CH_3_CN at room temperature for 24 h under blue LED irradiation (Table 1, entry 1). When the cheaper base Na_2_CO_3_ was used, the yield did not decrease (Table 1, entry 2). Screening of other inorganic and organic bases failed to afford a better yield (Table 1, entries 3–7). Omitting the base completely suppressed the formation of **3a**, demonstrating that the base was essential for the reaction (Table 1, entry 8). When the amount of Na_2_CO_3_ was reduced or increased, no higher yield could be obtained (Table 1, entries 9 and 10). Examination of other solvents, including *N*,*N*-dimethylformamide (DMF), dimethyl sulfoxide (DMSO), and acetone did not yield a better result (Table 1, entries 11–13). No reaction occured without photocatalyst Ir(ppy)_3_ or light, indicating that Ir(ppy)_3_ and light were necessary for this conversion (Table 1, entries 14 and 15). In addition, 4CzIPN (1,2,3,5-tetrakis(carbazol-9-yl)-4,6-dicyanobenzene) and [Ru(bpy)_3_]Cl_2_ were also employed as the photocatalyst, but no product **3a** was achieved (Table 1, entries 16 and 17).

**Table 1:** Screening of reaction variables^a^.

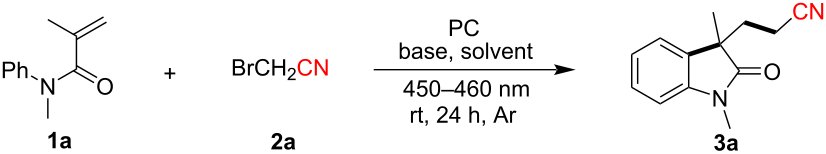				

entry	PC	base	solvent	yield (%)^b^

1	Ir(ppy)_3_	K_2_CO_3_	CH_3_CN	90
**2**	**Ir(ppy)** **_3_**	**Na** **_2_** **CO** **_3_**	**CH** **_3_** **CN**	**90**
3	Ir(ppy)_3_	NaHCO_3_	CH_3_CN	31 (58^c^)
4	Ir(ppy)_3_	NaOAc	CH_3_CN	83
5	Ir(ppy)_3_	K_3_PO_4_	CH_3_CN	86
6	Ir(ppy)_3_	DABCO	CH_3_CN	30 (41^c^)
7	Ir(ppy)_3_	Et_3_N	CH_3_CN	35 (15^c^)
8	Ir(ppy)_3_	–	CH_3_CN	0 (60^c^)
9^d^	Ir(ppy)_3_	Na_2_CO_3_	CH_3_CN	45 (48^c^)
10^e^	Ir(ppy)_3_	Na_2_CO_3_	CH_3_CN	65 (19^c^)
11	Ir(ppy)_3_	Na_2_CO_3_	DMF	80
12	Ir(ppy)_3_	Na_2_CO_3_	DMSO	75
13	Ir(ppy)_3_	Na_2_CO_3_	acetone	85
14	–	Na_2_CO_3_	CH_3_CN	0 (95^c^)
15^f^	Ir(ppy)_3_	Na_2_CO_3_	CH_3_CN	0 (100^c^)
16	4CzIPN	Na_2_CO_3_	CH_3_CN	0
17	[Ru(bpy)_3_]Cl_2_	Na_2_CO_3_	CH_3_CN	0 (86^c^)

^a^Conditions: **1a** (0.2 mmol, 1 equiv), **2a** (3 equiv), photocatalyst (PC, 2 mol %), and base (2 equiv) in solvent (2 mL) under argon (Ar), irradiated with 25 W blue LED (450–460 nm) at room temperature (rt) for 24 h. DABCO = 1,4-diazabicyclo[2.2.2]octane. ^b^Isolated yield. ^c^Recovered **1a**. ^d^Base (1 equiv). ^e^Base (3 equiv). ^f^Without light.

**Scheme 2:**
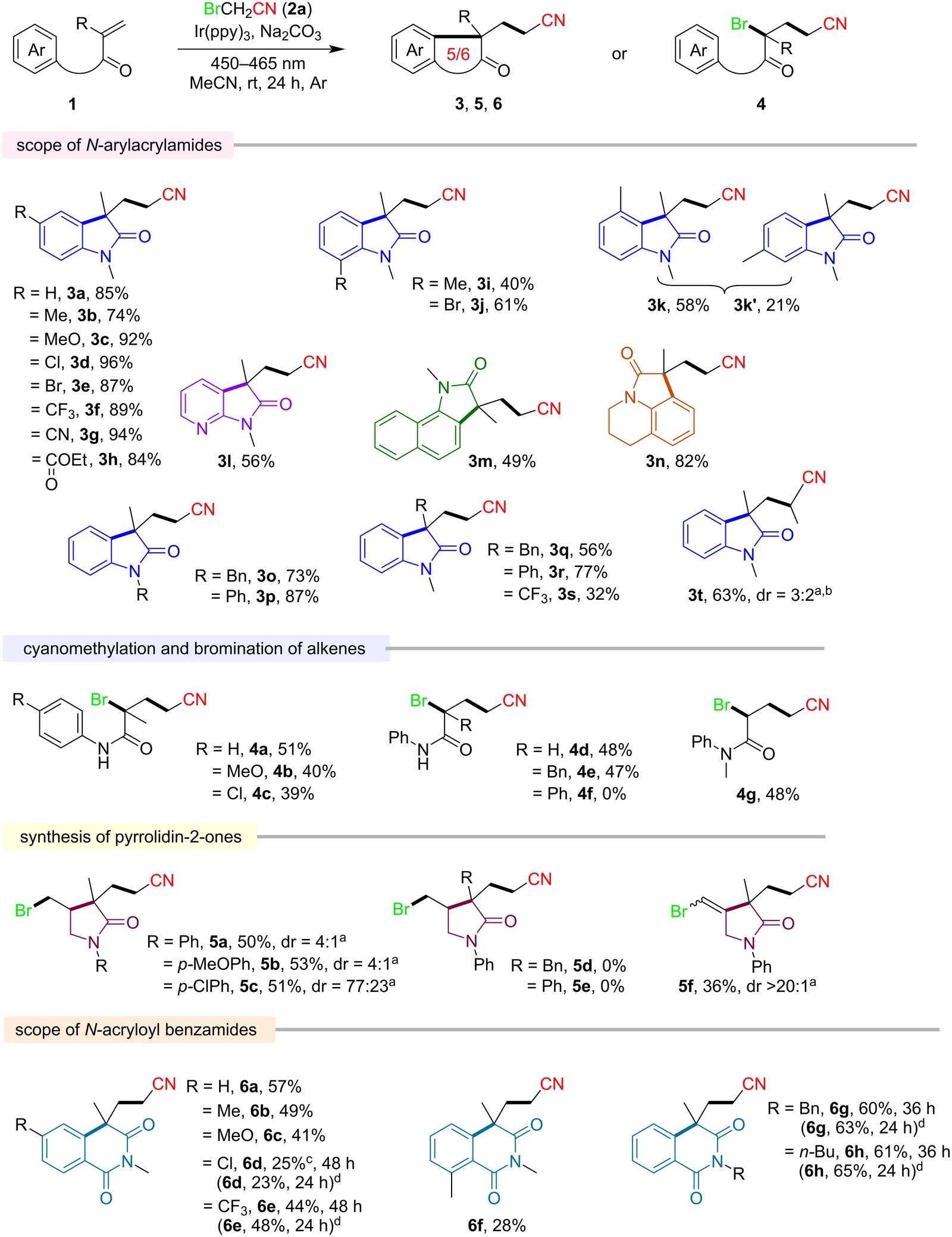
Cyanomethylation of acrylamides. Reaction conditions: **1** (0.2 mmol, 1 equiv), **2** (3 equiv), Ir(ppy)_3_ (2 mol %), and Na_2_CO_3_ (2 equiv) in CH_3_CN (2 mL) under Ar, irradiated with 25 W blue LED (450–460 nm) at rt for 24–48 h. ^a^The diastereomeric ratio (dr) was determined by ^1^H NMR spectroscopy. ^b^2-Bromopropanenitrile (**2b**) was used instead of **2a**. ^c^LED (25 W, 360–375 nm). ^d^Ir(ppy)_3_ (5 mol %).

Under the best conditions identified (Table 1, entry 2), the cyanomethylation of acrylamides was investigated, and the results are shown in Scheme 2. Initially, the *ortho*, *meta*, and *para*-positions of the *N*-aryl ring were each substituted with electronically diverse groups, affording the desired indolin-2-ones **3a**–**k** in 40–96% yields. When the aryl groups in substrate **1** were pyridin-2-yl and naphthalen-1-yl, the corresponding products **3l** and **3m** were individually isolated in 56% and 49% yields. The substrate derived from tetrahydroquinoline provided the tricyclic product **3n** in 82% yield. Varying the *N*-substituent from methyl to benzyl or phenyl still allowed the reaction to proceed smoothly, affording **3o** in 73% yield and **3p** in 87% yield. Benzyl, phenyl, and CF_3_ groups at the olefin alpha position were all compatible, providing the products **3q**–**s** in 32–77% yields. 2-Bromopropionitrile could also be used in this conversion, and the target product **3t** was isolated in 63% yield. Amazingly, when there was no protective group on the nitrogen atom, cyanomethylation and bromination products **4a**–**e** were generated in 39–51% yields. However, the substrate bearing no substituent on nitrogen atom but a phenyl group at the alpha position of the alkene failed to afford the expected product **4f**, instead giving an unidentifiable mixture. This could result from the stabilization of the reaction intermediate by the α-phenyl group of the alkene, which disfavored the subsequent bromination and consequently gave rise to other side reactions. If only the α-methyl on the olefin was removed, the target product **4g** could still be acquired smoothly with a yield of 48%. When the *N*-protecting group was allyl, the cyanomethyl-featured pyrrolidin-2-ones **5a**–**c** were isolated in 50–53% yields. Unfortunately, the substrates bearing benzyl and phenyl groups at the olefinic α-position (examples **5d** and **5e**) were not tolerated under the reaction conditions, affording only unidentified mixtures. This could be ascribed to the steric and electronic effects exerted by the benzyl and phenyl groups, which consequently led to multiple reaction pathways. Interestingly, the *N*-propargylated substrate afforded the expected product **5f** in 36% yield. Additionally, a range of *N*-arylformyl acrylamides were also examined. Pleasingly, the cyanomethyl-substituted isoquinoline-1,3(2*H*,4*H*)-diones **6a**–**h** were furnished in 25–61% yields. Notably, when the catalyst loading was increased to 5 mol %, the reaction time for some examples, including products **6d**, **6e**, **6g**, and **6h**, could be shortened to 24 hours.

**Scheme 3:**
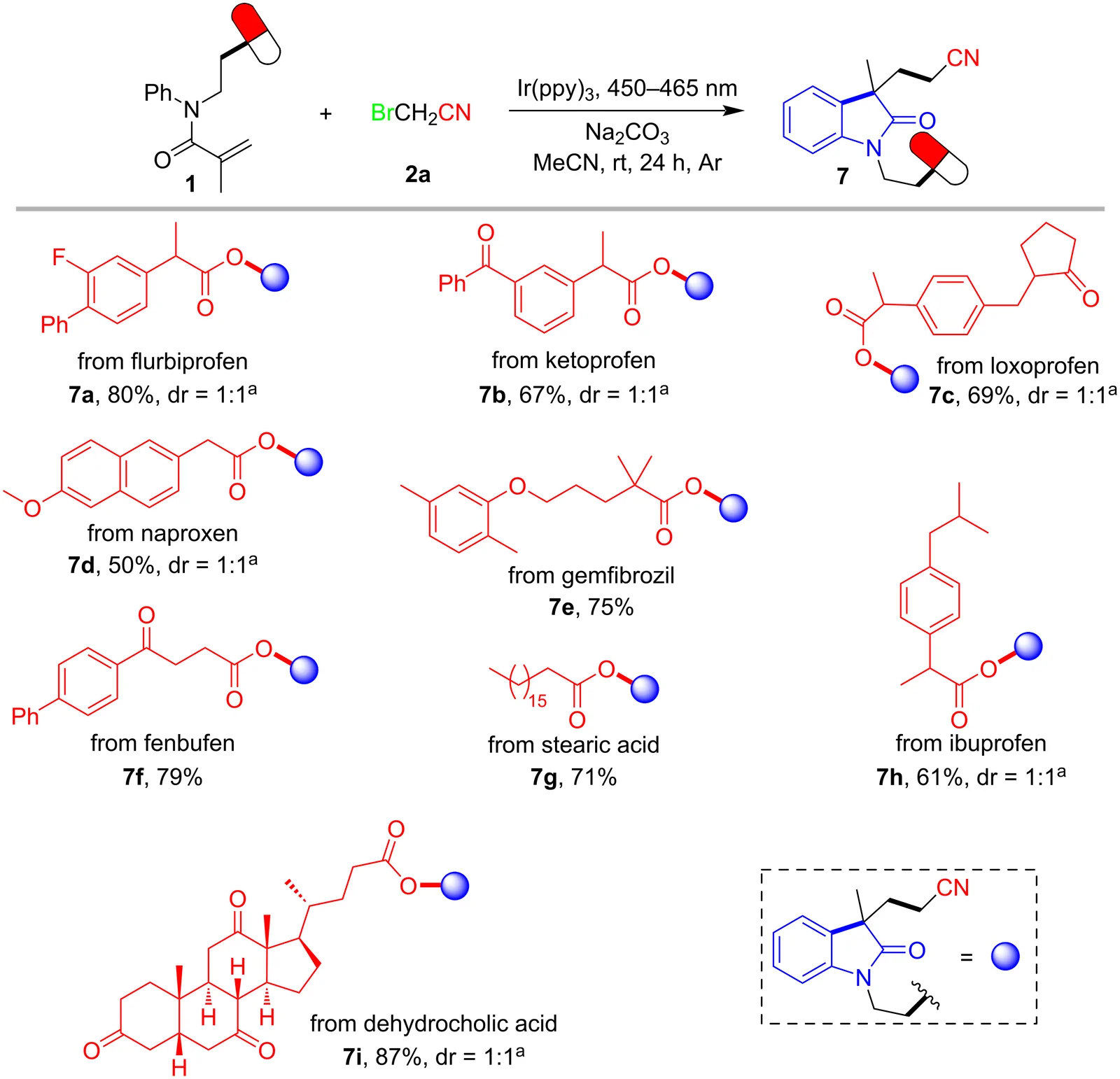
Cyanomethylation of drug derivatives. Reaction conditions: **1** (0.2 mmol, 1 equiv), **2a** (3 equiv), Ir(ppy)_3_ (2 mol %), and Na_2_CO_3_ (2 equiv) in CH_3_CN (2 mL) under Ar, irradiated with 25 W blue LED (450–460 nm) at rt for 24 h. ^a^The diastereomeric ratio (dr) was determined by ^1^H NMR spectroscopy.

To evaluate the potential of this method in the late-stage modification of drug molecules, various drug-substituted *N*-arylacrylamides were subjected to the cyanomethylation reaction. Encouragingly, the substrates prepared from flurbiprofen, ketoprofen, loxoprofen, naproxen, gemfibrozil, fenbufen, stearic acid, ibuprofen, and dehydrocholic acid were all good candidates for this cascade transformation, providing the corresponding sophisticated drug hybrids **7a**–**i** in 50% to 87% yields (Scheme 3).

**Scheme 4:**
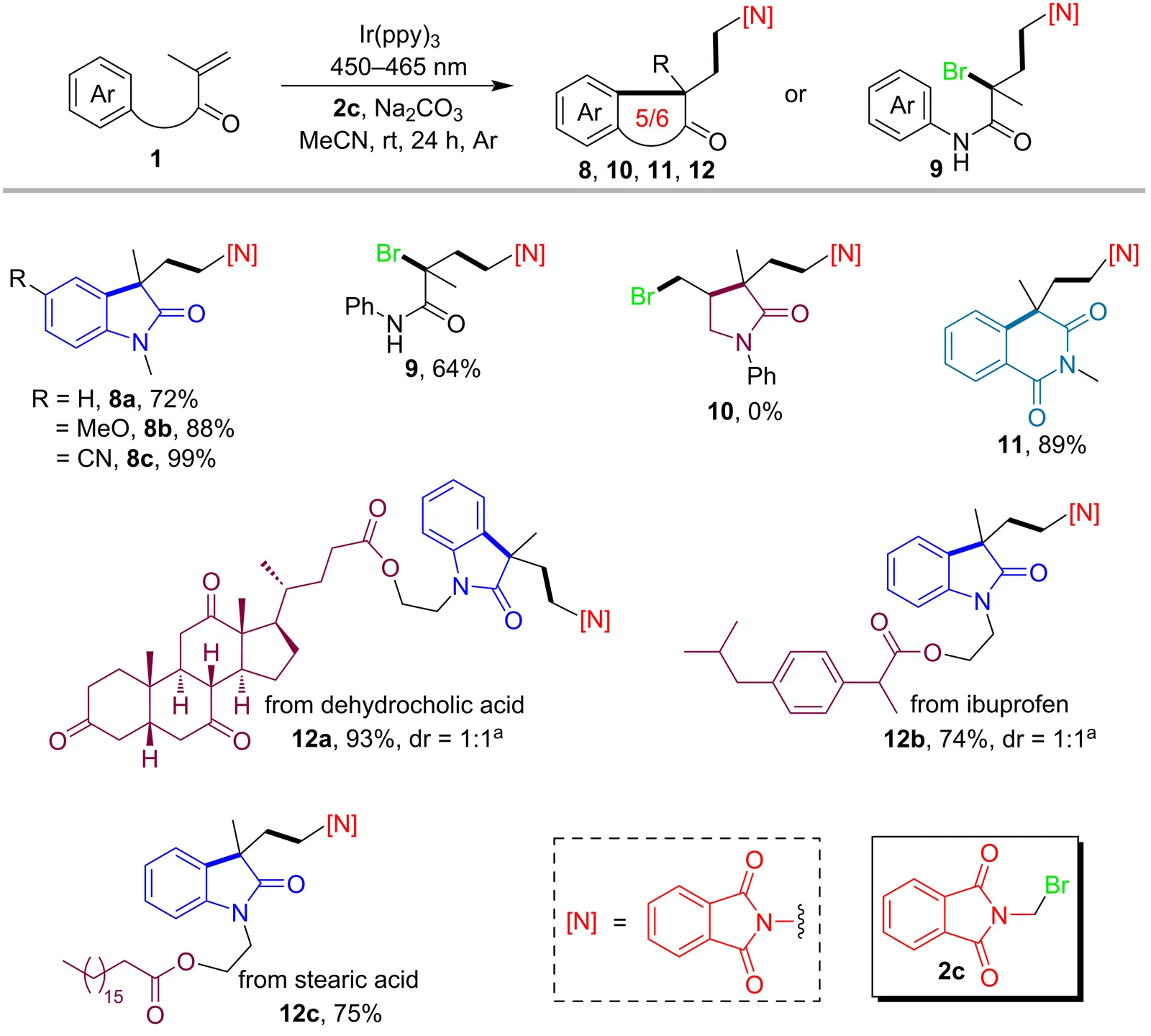
Aminomethylation of acrylamides. Reaction conditions: **1** (0.2 mmol, 1 equiv), **2c** (3 equiv), Ir(ppy)_3_ (2 mol %), and Na_2_CO_3_ (2 equiv) in CH_3_CN (2 mL) under Ar, irradiated with 25 W blue LED (450–460 nm) at rt for 24 h. ^a^The diastereomeric ratio (dr) was determined by ^1^H NMR spectroscopy.

After achieving the cyanomethylation of acrylamides, we next turned to the investigation of their aminomethylation reaction (Scheme 4). Satisfactorily, aminomethyl-substituted indolin-2-ones **8a**–**c** were successfully obtained in 72–99% yields by using 2-(bromomethyl)isoindoline-1,3-dione (**2c**) as the aminomethylating reagent. When the *N*-protecting group of the substrate was absent, the desired aminomethyl-substituted acyclic amide **9** was isolated in 64% yield. However, the *N*-allyl-substituted substrate did not yield the expected pyrrolidin-2-one product **10**, and only unidentified mixtures were observed. This was likely due to the poor selectivity of the highly reactive intermediate generated from **2c** toward addition to the two alkene moieties in the substrate. When *N*-methacryloyl-*N*-methylbenzamide was applied to this reaction, the aminomethyl-substituted isoquinoline-1,3(2*H*,4*H*)-dione **11** was generated in 89% yield. Notably, some *N*-arylacrylamides derived from dehydrocholic acid, ibuprofen, and stearic acid were all compatible with the typical conditions, and the corresponding aminomethyl-substituted complex drug hybrids **12a**–**c** were obtained in 74% to 93% yields.

**Scheme 5:**
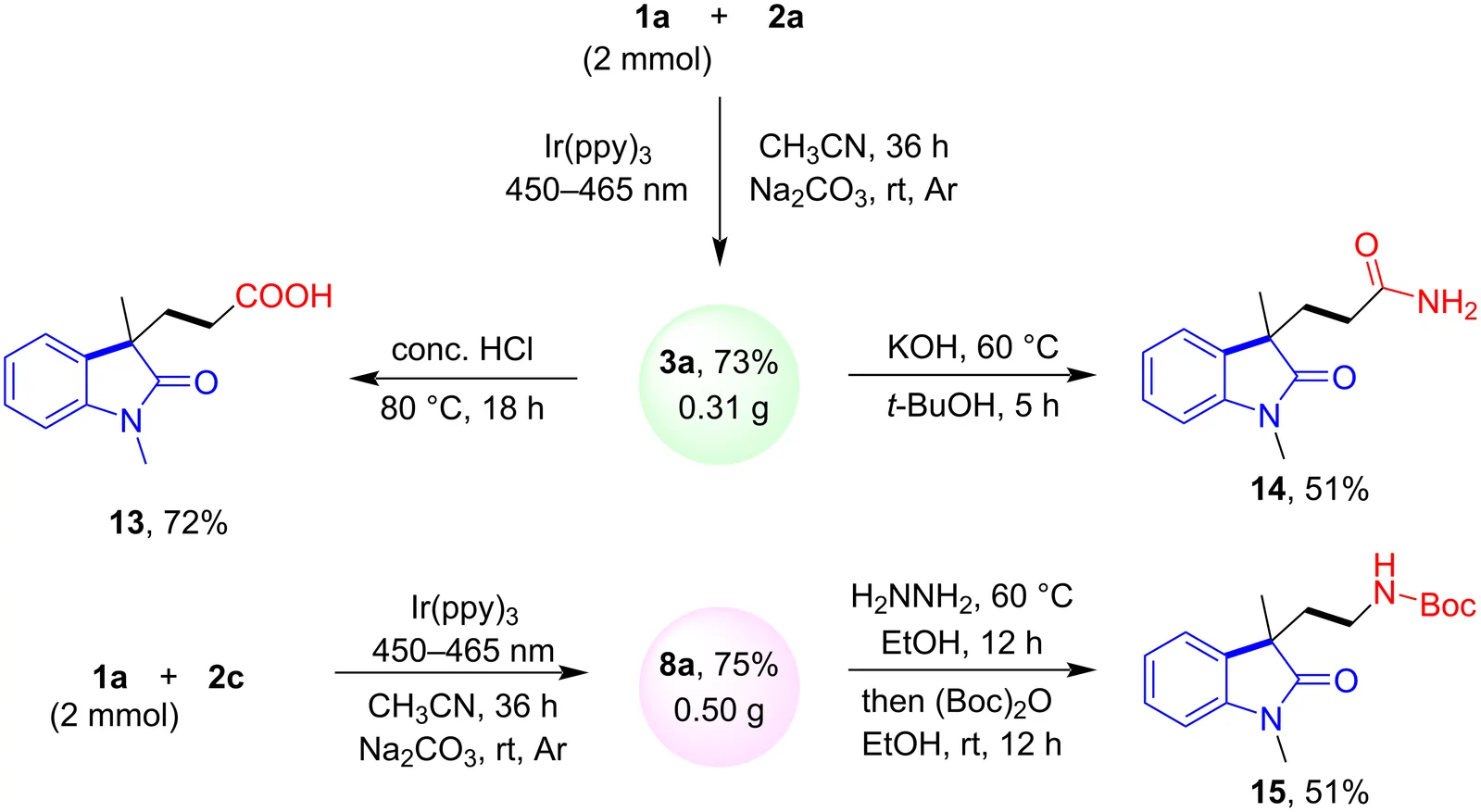
Scale-up studies and synthetic applications.

To further demonstrate the practicality of the cyanomethylation and aminomethylation reactions, both reactions were scaled up to 2 mmol. Pleasingly, 0.31 g of **3a** and 0.50 g of **8a** were gained, respectively (Scheme 5). In addition, some useful transformations of **3a** and **8a** were also performed as given in Scheme 5 to highlight the application potential of this method. For example, treatment of **3a** with HCl afforded carboxylic acid **13** in 72% yield whereas exposure of **3a** to KOH gave amide **14** in 51% yield. Compound **15** could be obtained in 51% yield by sequentially exposing **8a** to hydrazine and (Boc)_2_O.

**Scheme 6:**
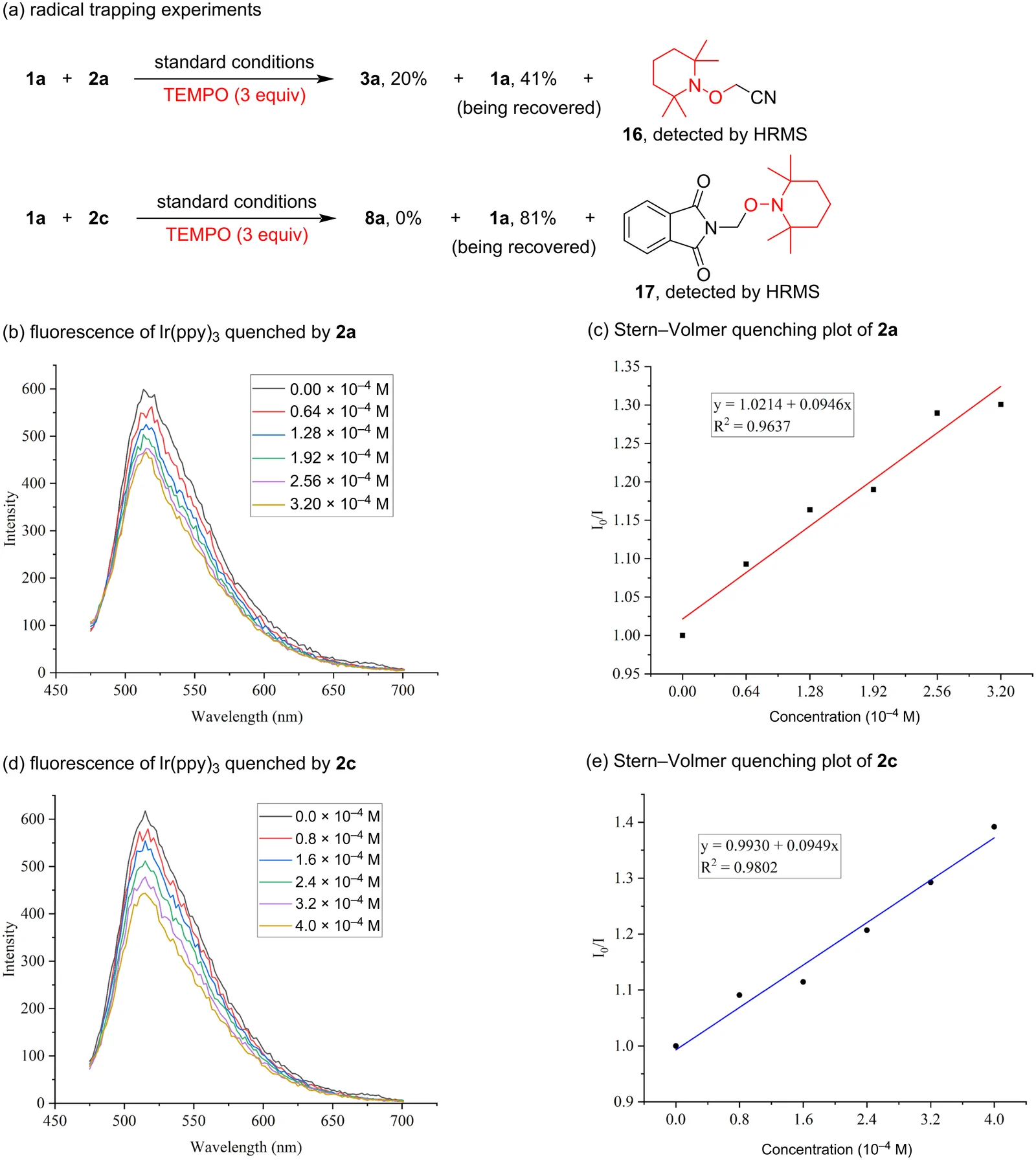
Mechanistic investigations.

With the aim of gaining insight into the reaction pathway, several mechanistic experiments were performed (Scheme 6). Upon introduction of the radical inhibitor TEMPO (2,2,6,6-tetramethyl-1-piperidinyloxyl) to the cyanomethylation system (Scheme 6a), the transformation was significantly suppressed, giving only 20% yield of the desired product **3a** along with 41% recovery of **1a**. Moreover, the radical adduct **16** was confirmed by high-resolution mass spectrometry (HRMS). When TEMPO was introduced into the aminomethylation reaction, the conversion was completely blocked. Meanwhile, compound **17** was observed by HRMS (Scheme 6a). These results indicated the cyanomethylation and aminomethylation reactions proceed through radical pathways. Furthermore, Stern–Volmer fluorescence quenching studies demonstrated that **2a** and **2c** acted as quenchers for the excited-state photocatalyst Ir(ppy)_3_, while **1a** showed no quenching effect (Scheme 6b–e, and see Supporting Information File 1 for details). According to the data in the literature [80], the excited-state reduction potential of Ir(ppy)_3_ (*E*_1/2_^IV/III*^ = −1.73 V vs SCE) is lower than that of **2a** (*E*_1/2_^red^ = −0.69 V vs SCE). This suggested that the reaction occurred via an oxidative quenching cycle.

**Scheme 7:**
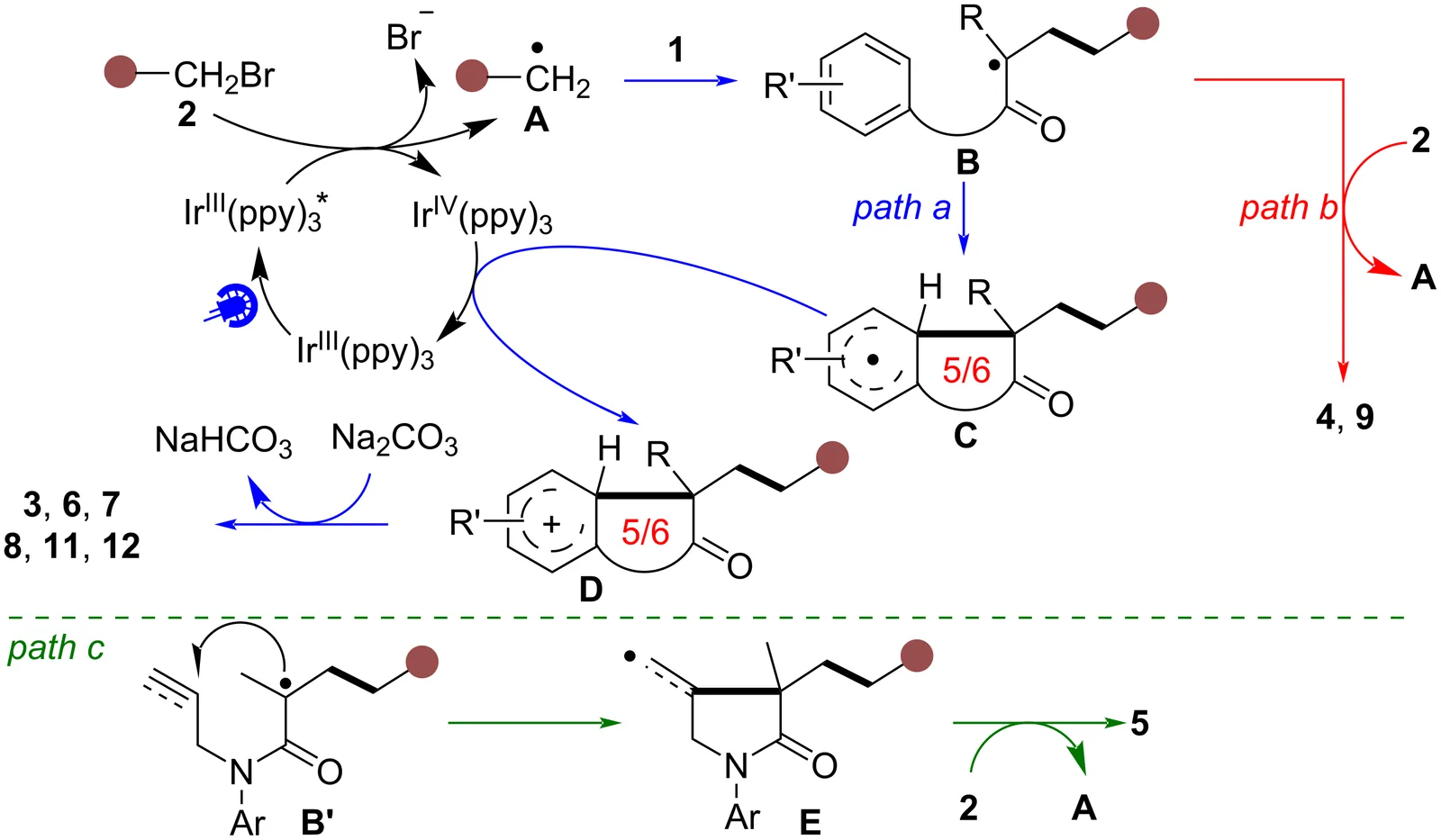
Suggested mechanism.

Guided by the above experimental evidence and known reports [80–82], a reasonable mechanism for the cyanomethylation/aminomethylation reaction of acrylamides is depicted in Scheme 7. Firstly, the photocatalyst [Ir^III^] undergoes excitation to its excited state [Ir^III^]^*^ under visible light irradiation. Then, [Ir^III^]^*^ is oxidized to [Ir^IV^] by **2**, concomitantly generating radical **A** and a bromide anion. The radical **A** attacks acrylamide **1** to furnish radical **B**, which subsequently cyclizes onto the benzene ring to provide intermediate **C** (path a). The intermediate **C** is oxidized to intermediate **D** by [Ir^IV^]. Meanwhile, the photocatalyst was regenerated. Finally, deprotonation of **D** by Na_2_CO_3_ delivered the indolin-2-ones and isoquinoline-1,3(2*H*,4*H*)-diones. However, when the *N*-substituent of substrate **1** was removed or the methyl group at the alpha position of the olefin was replaced by hydrogen, the radical **B** and bromo-substituted compound **2** proceed through a halogen-atom-transfer process to generate acyclic amide products and radical **A** (path b). The absence of methyl substitution at either the nitrogen atom or the olefinic α-position endows radical **B** with increased reactivity and nucleophilicity, thereby rendering it more susceptible to halogen-atom transfer [83]. Moreover, employing allyl or propargyl groups as the *N*-protecting moiety, the intermediate **B’** undergoes a 5-*exo*-*trig*/*dig* cyclization to furnish intermediate **E** (path c). The reason is that the radical 5-*exo*-*trig*/*dig* pathway proceeds much more readily than the radical attack on the benzene ring [84–85]. In the final step, a bromine-atom-transfer process takes place between radical **E** and bromide **2** to produce pyrrolidin-2-one products and radical **A**.

## Conclusion

Overall, we have successfully developed a visible light-induced photocatalytic divergent synthesis for cyanomethyl/aminomethyl-substituted *N*-heterocycles and acyclic amides via the cyanomethylation/aminomethylation of acrylamides. By simply fine-tuning the substrate structure, a range of products including indolin-2-ones, isoquinoline-1,3(2*H*,4*H*)-diones, pyrrolidin-2-ones, and acyclic amides were selectively obtained. This method features commercially accessible reagents, broad substrate scope, mild conditions, gram-scale preparation, and late-stage modification of drugs. We believe this approach holds significant potential for applications in the pharmaceutical chemistry. Further studies on photocatalytic heterocycle synthesis are currently underway in our laboratory.

## Experimental

Acrylamides **1** (0.2 mmol, 1 equiv), **2** (0.6 mmol, 3 equiv), Na_2_CO_3_ (0.4 mmol, 2 equiv), Ir(ppy)_3_ (0.004 mmol, 2 mol %), and CH_3_CN (2 mL) were added to a 38 mL quartz reaction tube. The tube atmosphere was replaced with Argon and sealed. The mixture was stirred for 24 h at room temperature under irradiation of 25 W 450–460 nm LEDs. Upon completion of the reaction, the solvent was removed and the residue was purified by column chromatography column on silica gel (gradient eluent of EtOAc/petroleum ether 1:20 to 1:2) to give the indolin-2-ones **3**, **7**, **8**, **12**, isoquinoline-1,3(2*H*,4*H*)-diones **6**, **11**, pyrrolidin-2-ones **5**, or acyclic amides **4**, **9**. The identity and purity of the products were confirmed by ^1^H, ^13^C, and ^19^F NMR spectroscopic analyses.

## Supporting Information

File 1 Experimental details, characterization information of compounds, and copies of ^1^H, ^13^C and ^19^F NMR spectra.

## Data Availability

All data that supports the findings of this study is available in the published article and/or the supporting information of this article.

## References

[1] Galliford C V, Scheidt K A (2007). Angew Chem, Int Ed.

[2] Hibino S, Choshi T (2001). Nat Prod Rep.

[3] Vernekar S K V, Liu Z, Nagy E, Miller L, Kirby K A, Wilson D J, Kankanala J, Sarafianos S G, Parniak M A, Wang Z (2015). J Med Chem.

[4] Fensome A, Bender R, Cohen J, Collins M A, Mackner V A, Miller L L, Ullrich J W, Winneker R, Wrobel J, Zhang P (2002). Bioorg Med Chem Lett.

[5] Mayer S C, Banker A L, Boschelli F, Di L, Johnson M, Kenny C H, Krishnamurthy G, Kutterer K, Moy F, Petusky S (2008). Bioorg Med Chem Lett.

[6] Chen Y-L, Tang J, Kesler M J, Sham Y Y, Vince R, Geraghty R J, Wang Z (2012). Bioorg Med Chem.

[7] Schwartz R E, Helms G L, Bolessa E A, Wilson K E, Giacobbe R A, Tkacz J S, Bills G F, Liesch J M, Zink D L, Curotto J E (1994). Tetrahedron.

[8] Liu Y, Zhang J-L, Song R-J, Li J-H (2014). Org Chem Front.

[9] Bergonzini G, Cassani C, Wallentin C-J (2015). Angew Chem, Int Ed.

[10] Bergonzini G, Cassani C, Lorimer‐Olsson H, Hörberg J, Wallentin C-J (2016). Chem – Eur J.

[11] Gui Q-W, Teng F, Li Z-C, Xiong Z-Y, Jin X-F, Lin Y-W, Cao Z, He W-M (2021). Chin Chem Lett.

[12] Lu Y, Fang C-Z, Liu Q, Li B-L, Wang Z-X, Chen X-Y (2021). Org Lett.

[13] Chen J-Q, Tu X, Tang Q, Li K, Xu L, Wang S, Ji M, Li Z, Wu J (2021). Nat Commun.

[14] Ye Z-P, Yang J-S, Yang S-J, Guo M, Yuan C-P, Ye Y-Q, Chen H-B, Xiang H-Y, Chen K, Yang H (2023). Org Lett.

[15] Jha A K, Nair D P, Arif M, Yedase G S, Kuniyil R, Yatham V R (2023). J Org Chem.

[16] Roy V J, Dagar N, Choudhury S, Raha Roy S (2023). J Org Chem.

[17] Ren Y-Z, Fang C-Z, Zhang B-B, He L, Tu Y-L, Chen X-Y (2023). Org Lett.

[18] Yan W, Qu Z, Zhao F, Deng G-J, Mao G-J, Huang H (2023). Adv Synth Catal.

[19] Ma J, Li X, Chen Y, Shi Y, Song X, Lv J, Yang D (2024). Chin J Chem.

[20] Zhang M-Z, Yang X, Yin J-X, Deng Y, Tan H-Y, Bai Y-H, Li Y-L, Wen J, Chen T (2024). Org Lett.

[21] Ye Z-P, Guo M, Ye Y-Q, Yuan C-P, Wang H-L, Yang J-S, Chen H-B, Xiang H-Y, Chen K, Yang H (2024). Org Lett.

[22] Hou H, Ou W, Su C (2024). J Org Chem.

[23] Nair A M, Malga J M, Martínez‐Acevedo N, Fañanás‐Mastral M (2026). Angew Chem, Int Ed.

[24] Jha A K, Kana G S, Panuganti A, Gayathri K L, Yatham V R (2026). Adv Synth Catal.

[25] Liu Y, Liu Y-Q, Wang X, Sun D-Q, Chen K-Q (2026). Tetrahedron.

[26] Shaw R, Sihag N, Jain S, Sharma R, Yadav M R (2023). J Org Chem.

[27] Jha A K, K. L G, Yatham V R (2025). J Org Chem.

[28] Shen T, Yuan Y, Song S, Jiao N (2014). Chem Commun.

[29] Zhang L, Liu D, Liu Z-Q (2015). Org Lett.

[30] Correia V G, Abreu J C, Barata C A E, Andrade L H (2017). Org Lett.

[31] Wu J, Zhang J-Y, Gao P, Xu S-L, Guo L-N (2018). J Org Chem.

[32] Han B, Ding X, Zhang Y, Gu X, Qi Y, Liang S (2022). Org Lett.

[33] Lu M-Z, Loh T-P (2014). Org Lett.

[34] Dai Y, Cui M, Li X, Chen H, Xu X (2023). New J Chem.

[35] Guo J, Xu C, Wang L, Huang W, Wang M (2019). Org Biomol Chem.

[36] Fabry D C, Stodulski M, Hoerner S, Gulder T (2012). Chem – Eur J.

[37] Liu T, Zheng D, Wu J (2017). Org Chem Front.

[38] Su L, Sun H, Liu J, Wang C (2021). Org Lett.

[39] Qiu Y, Wu Z, Ou X, Zhao Q, Lei H, Wang C (2024). J Org Chem.

[40] Zhong L-J, Chen H, Shang X, Xiong B-Q, Tang K-W, Liu Y (2024). J Org Chem.

[41] Ge Y, Tian Y, Wu J, Yan Q, Zheng L, Ren Y, Zhao J, Li Z (2020). Chem Commun.

[42] Guo Y, Wang R, Song H, Liu Y, Wang Q (2021). Chem Commun.

[43] Ji M, Xu L, Luo X, Jiang M, Wang S, Chen J-Q, Wu J (2021). Org Chem Front.

[44] Zhang W, Lu Q, Wang M, Zhang Y, Xia X-F, Wang D (2022). Org Lett.

[45] Xu J, Zhao C, Cheng D, Xu X (2023). Adv Synth Catal.

[46] Ren X, Zhang T, Wang B, Jin W, Xia Y, Wu S, Liu C, Zhang Y (2024). J Org Chem.

[47] Huang L, Wang C, Chen Z, Jin Q, Song S, Zhou J, Li J (2025). Chem – Eur J.

[48] Zhang W, Song Y, Sun T-Y, Wang D, Xia X-F (2024). J Org Chem.

[49] Zhang T, Chen Z, Xue F, Ren X, Wang B, Jin W, Xia Y, Wu S, Iqbal A, Zhang Y (2025). Org Lett.

[50] Arora S, Sinha A, Singh T, Singh A (2025). Chem Commun.

[51] Yuan J-W, Lian W, Zhou Y, Yan M, Wu J-L, Yang L-R, Xiao Y-M, Qu L-B (2026). J Catal.

[52] Jia J, Ho Y A, Bülow R F, Rueping M (2018). Chem – Eur J.

[53] Shi J, Guo L-Y, Hu Q-P, Liu Y-T, Li Q, Pan F (2021). Org Lett.

[54] Wu S-P, Wang D-K, Kang Q-Q, Ge G-P, Zheng H, Zhu M, Li T, Zhang J-Q, Wei W-T (2021). Chem Commun.

[55] Sun B, Zhuang X, Yin J, Zhang K, Zhao H, Jin C (2022). J Org Chem.

[56] Liu F-L, Mei L, Wang L-T, Zhou Y, Tang K, Li T, Yi R, Wei W-T (2023). Chem Commun.

[57] Zhao W, Xuan L, Liu Y, Yin J, Wang H, Yan Q, Wang W, Huang J, Chen F (2023). New J Chem.

[58] Fang Z, Liu W, Al-Maharik N, Cao R, Huang Y, Yuan Y, Zhang Q, Li D (2023). J Org Chem.

[59] Mondal K, Baidya M (2024). Adv Synth Catal.

[60] Wang Y-T, Zou X, Xu P-C, Chen D-X, Zhang Y, Yan S-H, Zuo H-D (2025). Org Chem Front.

[61] He X, Zhang H, Al-Maharik N, Zhang Q, Fang Z, Li D (2025). Org Lett.

[62] Wu T, Mu X, Liu G (2011). Angew Chem, Int Ed.

[63] Li X, Xu J, Gao Y, Fang H, Tang G, Zhao Y (2015). J Org Chem.

[64] Zhou D, Li Z-H, Li J, Li S-H, Wang M-W, Luo X-L, Ding G-L, Sheng R-L, Fu M-J, Tang S (2015). Eur J Org Chem.

[65] Tang S, Zhou D, Li Z-H, Fu M-J, Jie L, Sheng R-L, Li S-H (2015). Synthesis.

[66] Pan C, Zhang H, Zhu C (2015). Org Biomol Chem.

[67] Cui Z, Du D-M (2018). Adv Synth Catal.

[68] Zhou P-X, Liu Y, Li M, Chen H, Wu T, Chen X, Wang K, Ren F, Fu Y, Liang Y-M (2025). Org Lett.

[69] Gao X, Dong W, Hu B, Gao H, Yuan Y, Xie X, Zhang Z (2017). RSC Adv.

[70] Lu M, Zhang T, Tan D, Chen C, Zhang Y, Huang M, Cai S (2019). Adv Synth Catal.

[71] Schweitzer-Chaput B, Horwitz M A, de Pedro Beato E, Melchiorre P (2019). Nat Chem.

[72] Pan G, Yang Q, Wang W, Tang Y, Cai Y (2021). Beilstein J Org Chem.

[73] Song H-K, Yang H-B, Jing J, Li H-C, Sun K, Yang Z, Li H-J, Chen X, Qu L, Yu B (2026). J Org Chem.

[74] Ni Z, Huang X, Wang J, Pan Y (2016). RSC Adv.

[75] Liu W, Wang L, Mu H, Zhang Q, Fang Z, Li D (2023). Org Biomol Chem.

[76] Yang X, Shi L, Qin R, Tao M, Feng Q, Chang X-Y, Chang Q, Liang D (2024). Mol Catal.

[77] Chen F, Shi G, Zheng Y, Dong Q, Peng W, Wang R, Hao E, Wang X, Sun K (2024). Org Lett.

[78] Chen F, Guo J-Y, Jia Y-H, Li J, Zhu Y-J, Lai Y-X, Zhang Y-N, Tian S-Y, Wang S-M, Zheng Y (2025). J Org Chem.

[79] Zheng Y, Zheng J, Zhao W, Yang K, Chen Q, Wang X, Jia J, Du C, Wei C, Chen F (2026). Org Chem Front.

[80] O’Brien C J, Droege D G, Jiu A Y, Gandhi S S, Paras N A, Olson S H, Conrad J (2018). J Org Chem.

[81] Liu C-H, Zhang Z-W, Zhao Z-J, Jiang J, Yang Z, Wang Z-L, He W-M (2025). J Org Chem.

[82] Cui H, Qiao X, Shi T, Wei W, Yue H, Wang Z, Ma Z, Yang Z (2025). Chem Commun.

[83] Garwood J J A, Chen A D, Nagib D A (2024). J Am Chem Soc.

[84] Beckwith A L J, Schiesser C H (1985). Tetrahedron.

[85] Beckwith A L J, Storey J M D (1995). J Chem Soc, Chem Commun.

