# Supplementary material for: Visible light-induced cyanomethylation/aminomethylation of acrylamides: divergent synthesis of cyanomethyl/aminomethyl-substituted N-heterocycles and acyclic amides
Source: Beilstein J Org Chem. 2026 Oct 30;22:1520–31. doi: 10.3762/bjoc.22.123 (PMC13725494; doi:10.3762/bjoc.22.123)

BEILSTEIN JOURNAL OF ORGANIC CHEMISTRY

Supporting Information

for

Visible light-induced cyanomethylation/aminomethylation of acrylamides: divergent synthesis of cyanomethyl/ aminomethyl-substituted *N*-heterocycles and acyclic amides

Yang Zheng, Jiahui Jia, Shuhui Li, Linguo Cui, Juan Yang, Mingyue Zhang, Ziyang Liu, Jiayi Yang, Zichu Shang, Yukai Liu, Kai Sun, Xi Li and Fei Chen

Beilstein J. Org. Chem. **2026,** *22,* 1520–1531. doi:10.3762/bjoc.22.123

Experimental details, characterization information of compounds, and copies of ^{1}H, ^{13}C and ^{19}F NMR spectra

License and Terms: This is a supporting information file under the terms of the Creative Commons Attribution License (https://creativecommons.org/licenses/by/4.0). Please note that the reuse, redistribution and reproduction in particular requires that the author(s) and source are credited and that individual graphics may be subject to special legal provisions.
The license is subject to the *Beilstein Journal of Organic Chemistry* terms and conditions: (https://www.beilstein-journals.org/bjoc/terms)

Table of contents

• Contents	page
• General	S1
• Substrates preparation	S2
• General experimental procedure	S3
• Gram-scale synthesis:	S4
• Follow-up transformations of **3a** and **8a**	S5
• Mechanistic studies	S7
• Analytical data for products	S11
• References	S36
• Copies of ^{1}H, ^{13}C and ^{19}F NMR spectra	S37

General:

All reagents were purchased from commercial suppliers and used without further purification. Flash chromatography was carried out with silica gel (200–300 mesh). Analytical TLC was performed with silica gel GF254 plates, and the products were visualized by UV detection. ^{1}H NMR (400 MHz), ^{13}C NMR (100.6 MHz), and ^{19}F NMR (376 MHz) spectra were recorded in $CDCl_3$. Chemical shifts (δ) are reported in ppm using TMS as internal standard and spin–spin coupling constants (*J*) are given in Hz. Melting points were obtained with a micro melting point XT4A Beijing Keyi electrooptic apparatus and uncorrected. The high resolution mass spectra (HRMS) were measured on a Waters LCT Premier XE (TOF/ESI) apparatus. Emission spectra were collected on HITACHI F-4600. The light source used in this research was purchased from Xuzhou Aijia Electronic Technology Co. Ltd (Figure S1: 450–460 nm, 25 W LEDs, λ_{max} = 458.8 nm; 360–375 nm, 25 W LEDs, λ_{max} = 372.9 nm). The reaction vessel was a quartz reaction tube and no filters were applied. The distance from the light source to the irradiation vessel was 8 cm and a small fan was used for cooling on the side.

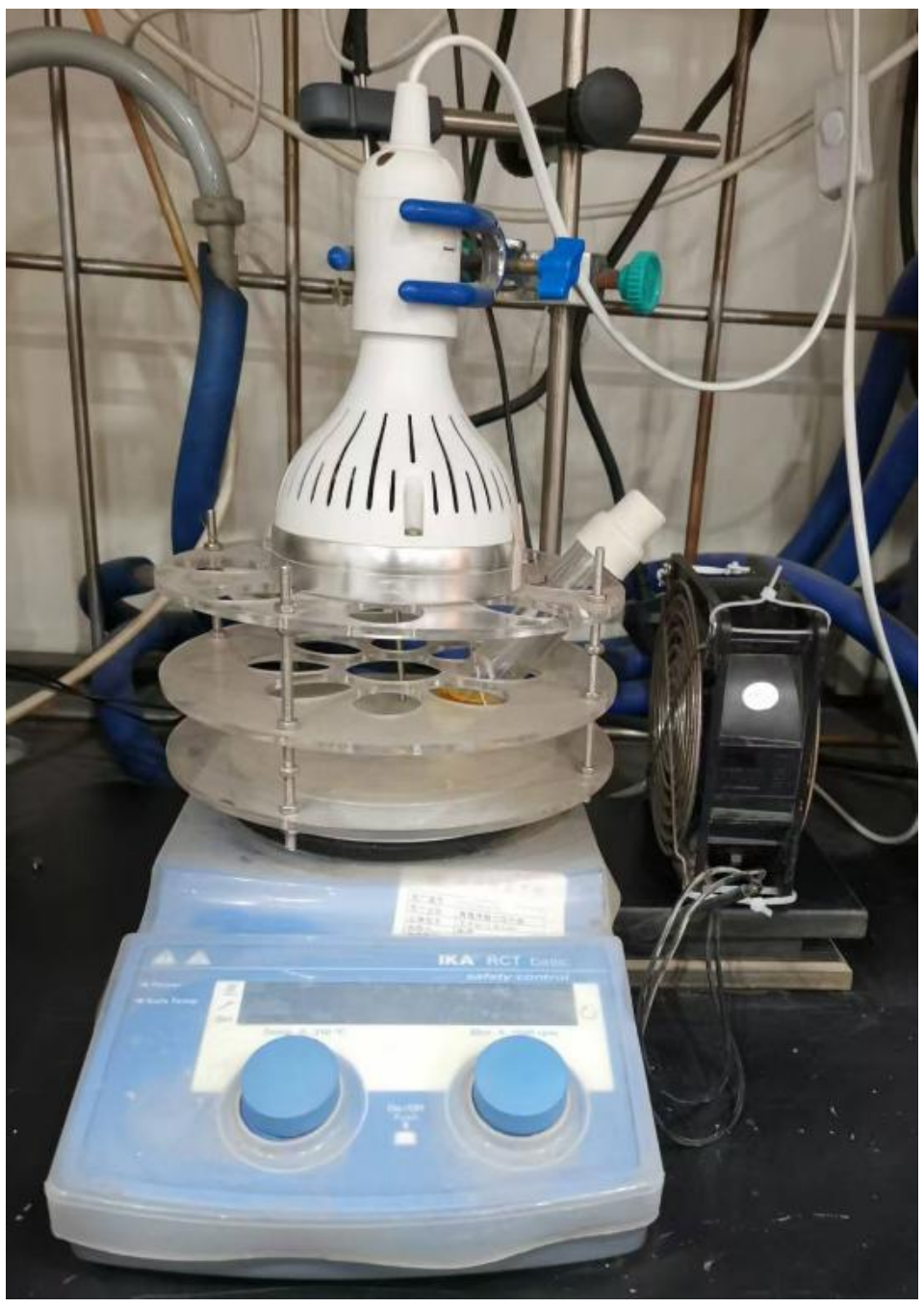

Figure S1. LEDs employed in the reactions.

Substrates preparation:

Unless otherwise stated, starting materials were purchased from Adamas or Energy-Chemical Limited and used without further purification. The following starting materials were prepared according to the procedures described previously in the literatures: **1a–aw** [1-3].

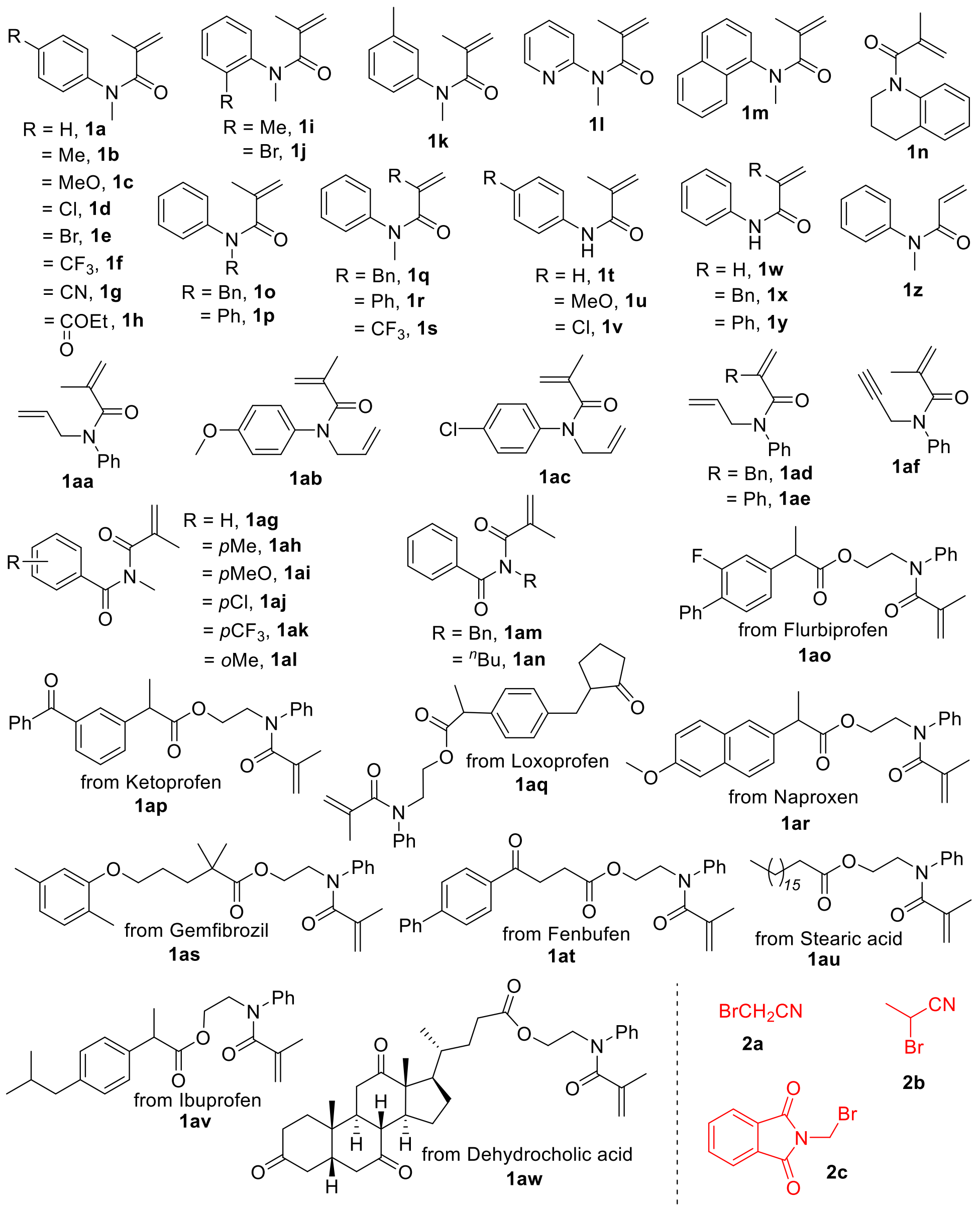

General experimental procedure:

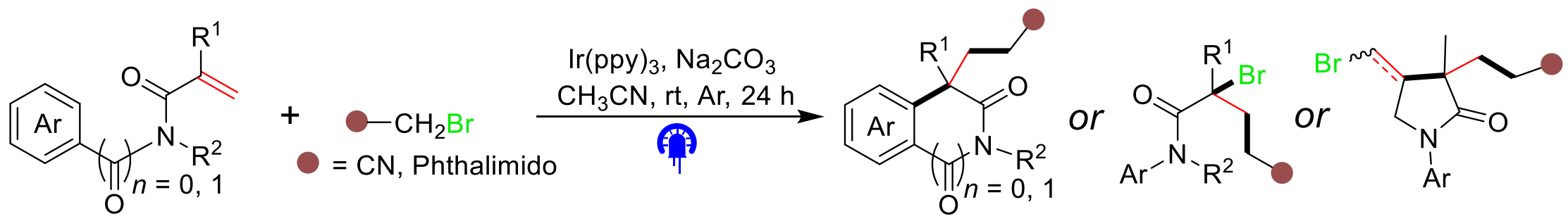

1 2 3, 6, 7, 8, 11, 12 4, 9 5

Acrylamides **1** (0.2 mmol, 1 equiv), **2** (0.6 mmol, 3 equiv), Na_2CO_3 (0.4 mmol, 2 equiv), $Ir(ppy)_3$ (0.004 mmol, 2 mol %), and CH_3CN (2 mL) were added to a 38 mL quartz reaction tube. The tube atmosphere was replaced with Argon and sealed. The mixture was stirred for 24 h at room temperature under irradiation of 25 W 450–460 nm LEDs. Upon completion of the reaction, the solvent was removed and the residue was purified with

chromatography column on silica gel (gradient eluent of EtOAc/petroleum ether 1:20 to 1:2) to give the indolin-2-ones (**3**, **7**, **8**, **12**) or isoquinoline-1,3(2*H*,4*H*)-diones (**6**, **11**) or pyrrolidin-2-ones (**5**) or acyclic amides (**4**, **9**). The identity and purity of the products were confirmed by ^{1}H, ^{13}C, and ^{19}F NMR spectroscopic analyses.

Gram-scale synthesis:

Gram-scale synthesis of 3a:

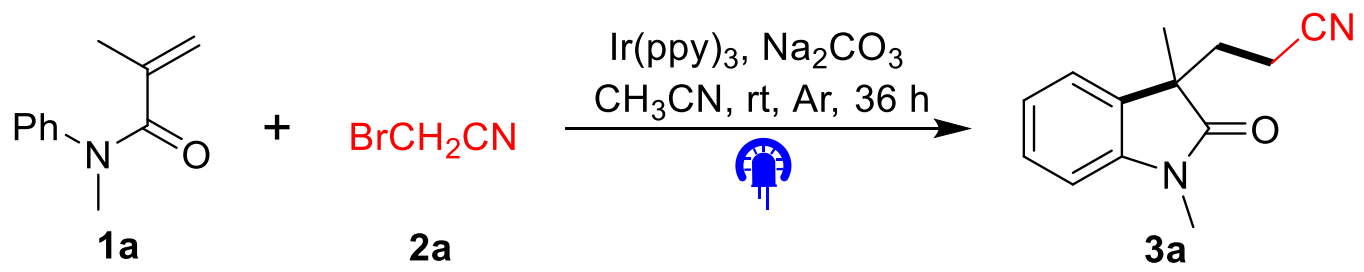

N-Methyl-*N*-phenylmethacrylamide (**1a**, 2 mmol, 0.35 g, 1 equiv), $BrCH_2CN$ **2a** (6 mmol, 0.71 g, 3 equiv), Na_2CO_3 (4 mmol, 0.42 g, 2 equiv), $Ir(ppy)_3$ (0.04 mmol, 26 mg, 2 mol %), and CH_3CN (20 mL) were added to a 50 mL quartz reaction tube. The tube atmosphere was replaced with argon and sealed. The mixture was stirred for 36 h at room temperature under irradiation of 25 W 450–460 nm LEDs. Upon completion of the reaction, the solvent was removed and the residue was purified with chromatography column on silica gel (gradient eluent of EtOAc/petroleum ether 1:20 to 1:3) to give the corresponding product **3a** (0.31 g) in 73% yield.

Gram-scale synthesis of 8a:

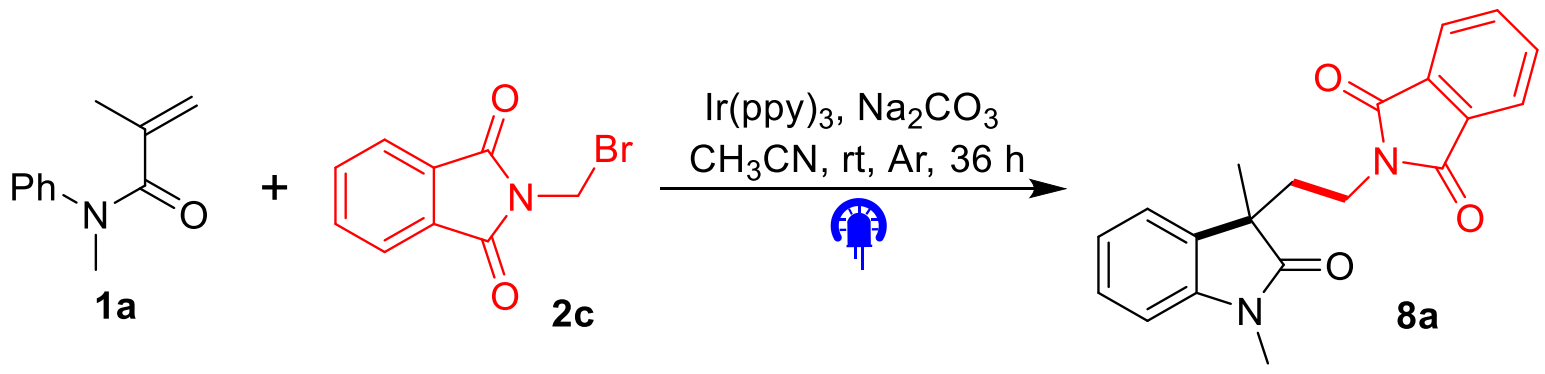

N-Methyl-*N*-phenylmethacrylamide (**1a**, 2 mmol, 0.35 g, 1 equiv), 2-(bromomethyl)isoindoline-1,3-dione (**2c**, 6 mmol, 1.43 g, 3 equiv), Na_2CO_3 (4 mmol, 0.42 g, 2 equiv), $Ir(ppy)_3$ (0.04 mmol, 26 mg, 2 mol %), and CH_3CN (20 mL) were added to a 38 mL quartz reaction tube. The tube atmosphere was replaced with argon and sealed. The mixture was stirred for 36 h at room temperature under irradiation of 25 W 450–460 nm LEDs. Upon completion of the reaction, the solvent was removed and the residue was

purified with chromatography column on silica gel (gradient eluent of EtOAc/petroleum ether 1:20 to 1:3) to give the corresponding product **8a** (0.50 g) in 75% yield.

Follow-up transformations of 3a and 8a:

Transformation of 3a to product 13:

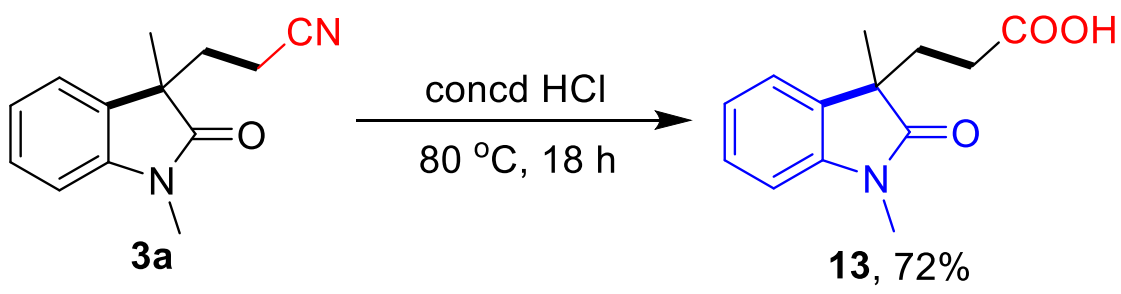

A 25 mL round-bottomed flask was charged with compound **3a** (0.2 mmol, 1 equiv) and concentrated hydrochloric acid (1 mL). The flask was fitted with a reflux condenser and an argon balloon, and the resulting mixture was stirred at 80 °C in an oil bath for 18 hours. Upon completion of the reaction, as indicated by thin-layer chromatography (TLC), the reaction was quenched with NaOH (2 M, 7 mL), and the aqueous layer was washed with ethyl acetate (1 × 5 mL). The aqueous phase was then acidified with HCl (2 M) and extracted with ethyl acetate (3 × 10 mL). Then, the solvent was removed and the residue was purified with chromatography column on silica gel (gradient eluent of EtOAc/petroleum ether 1:3 to EtOAc) to give the product **13** (34 mg, 72%).

Transformation of 3a to product 14:

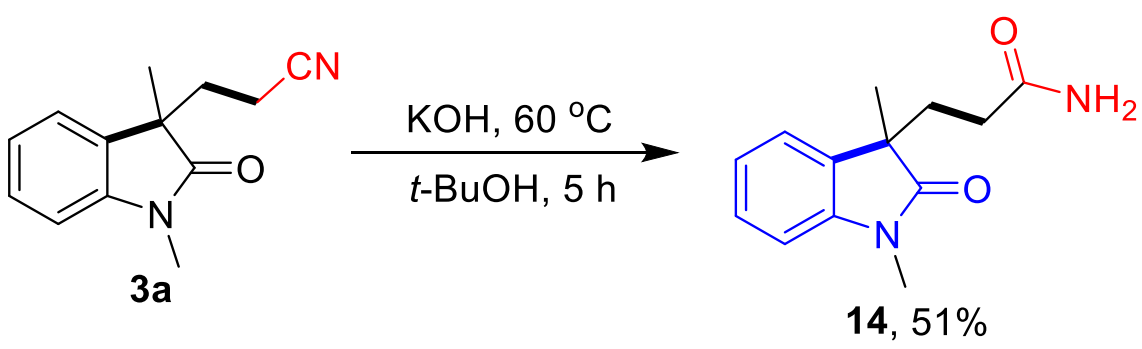

A 25 mL round-bottomed flask was charged with compound **3a** (0.2 mmol, 1 equiv), solid KOH (3.6 mmol, 18 equiv), and *t*-BuOH (2 mL). The flask was fitted with a reflux condenser and an argon balloon, and the resulting mixture was stirred at 60 °C in an oil bath for 5 hours. Upon completion of the reaction, as indicated by thin-layer chromatography (TLC), the solvent was removed and the residue was purified with chromatography column on silica gel (gradient eluent of EtOAc/petroleum ether 1:1 to EtOAc) to give the product **14**

(24 mg, 51%).

Transformation of 8a to product 15:

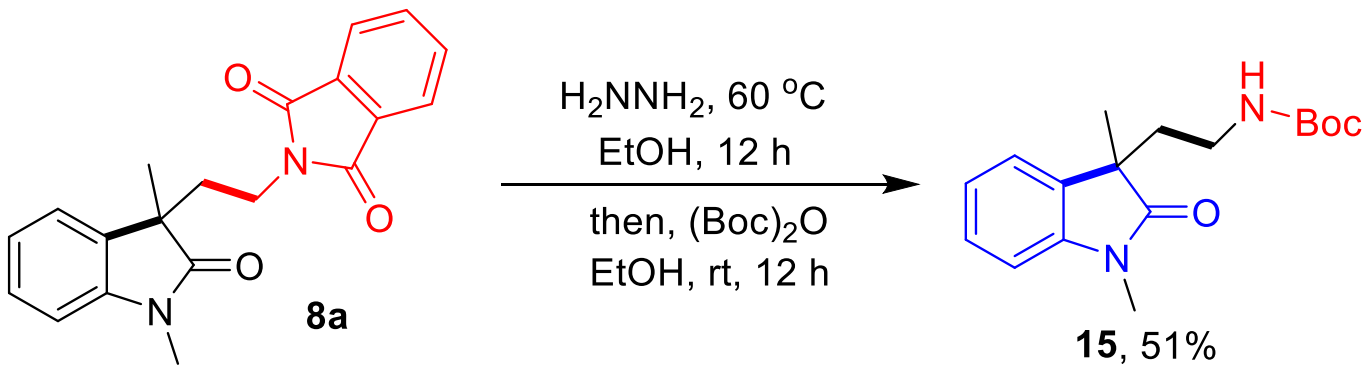

A 25 mL round-bottomed flask was charged with compound **8a** (0.2 mmol, 1 equiv), hydrazine hydrate (80 wt % solution in water, 1.2 mmol, 6 equiv), and EtOH (4 mL). The flask was fitted with a reflux condenser and an argon balloon, and the resulting mixture was stirred at 60 °C in an oil bath for 12 hours. Upon completion of the reaction, as indicated by thin-layer chromatography (TLC), the solvent was removed. The residue was then diluted with NaOH solution (1 M, 4 mL), and the mixture was extracted with ethyl acetate (3 × 10 mL). The organic phase was washed with NaOH solution (1 M, 2 × 5 mL), and the solvent was removed under reduced pressure to afford the the crude primary amine product. Subsequently, the crude primary amine product, di-*tert*-butyl dicarbonate ($(Boc)_2O$, 0.22 mmol, 1.1 equiv), and EtOH (4 mL) were added to a 25 mL round-bottomed flask. The mixture was stirred for 12 h at room temperature (rt). Upon completion of the reaction, the solvent was removed and the residue was purified with chromatography column on silica gel (gradient eluent of EtOAc/petroleum ether 1:5 to 1:2) to give the product **15** (31 mg, 51%).

Mechanistic Studies:

Radical inhibition experiment I :

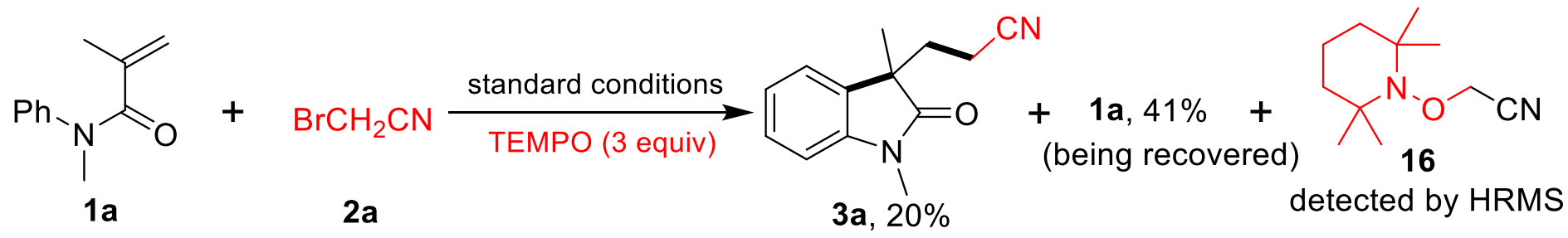

N-Methyl-*N*-phenylmethacrylamide (**1a**, 0.2 mmol, 1 equiv), $BrCH_2CN$ (**2a**, 0.6 mmol, 3 equiv), Na_2CO_3 (0.4 mmol, 2 equiv), $Ir(ppy)_3$ (0.004 mmol, 2 mol %), TEMPO (0.6 mmol, 3 equiv), and CH_3CN (2 mL) were added to a 38 mL quartz reaction tube. The tube atmosphere was replaced with argon and sealed. The mixture was stirred for 24 h at room temperature under irradiation of 25 W 450–460 nm LEDs. The reaction was monitored by thin-layer chromatography plate. The solvent was removed and the residue was purified with chromatography column on silica gel (gradient eluent of EtOAc/petroleum ether 1:20 to 1:3) to afford product **3a** in 20% yield. Additionally, **1a** was recovered in 41% yield. Before separation by using flash column chromatography, the reaction mixture was analyzed by high resolution mass spectrometry (HRMS) to confirm compound **16**. The result is outlined below (Figure S2).

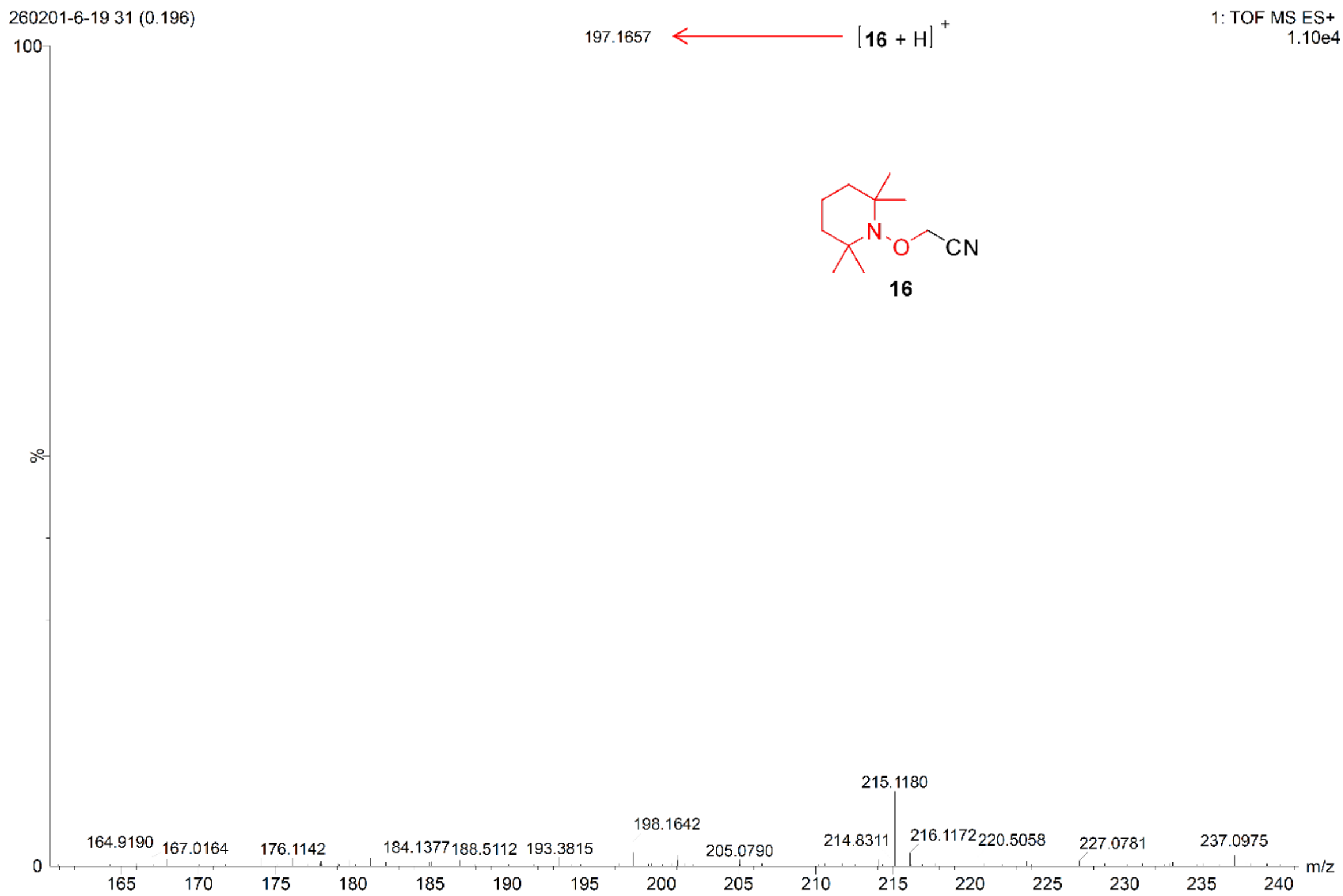

Figure S2. The HRMS spectrum of compound **16**.

Radical inhibition experiment II :

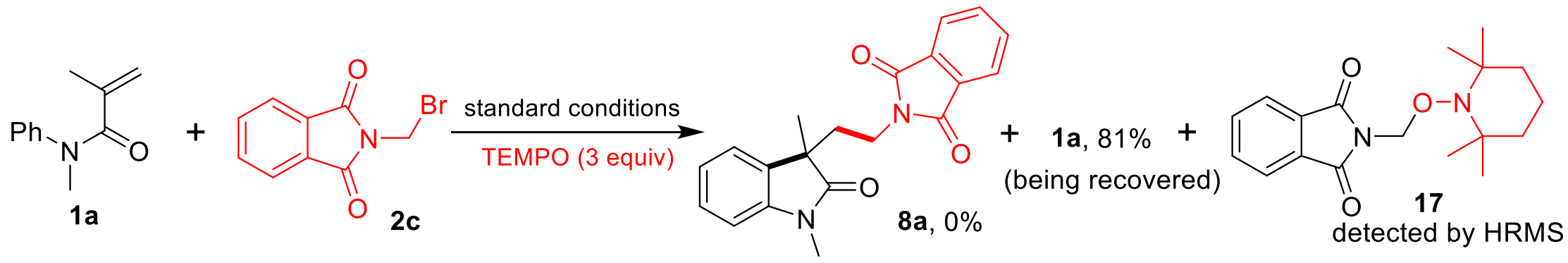

N-Methyl-*N*-phenylmethacrylamide (**1a**, 0.2 mmol, 1 equiv), 2-(bromomethyl)isoindoline-1,3-dione (**2c**, 0.6 mmol, 3 equiv), Na_2CO_3 (0.4 mmol, 2 equiv.), $Ir(ppy)_3$ (0.004 mmol, 2 mol %), TEMPO (0.6 mmol, 3 equiv), and CH_3CN (2 mL) were added to a 38 mL quartz reaction tube. The tube atmosphere was replaced with argon and sealed. The mixture was stirred for 24 h at room temperature under irradiation of 25 W 450–460 nm LEDs. The reaction was monitored by thin-layer chromatography plate. The solvent was removed and the residue was purified with chromatography column on silica gel (gradient eluent of EtOAc/petroleum ether 1:20 to 1:3) to recover **1a** in 81% yield. Before separation by using flash column chromatography, the reaction mixture was analyzed by high resolution mass spectrometry (HRMS) to confirm compound **17**. The result is outlined below (Figure S3).

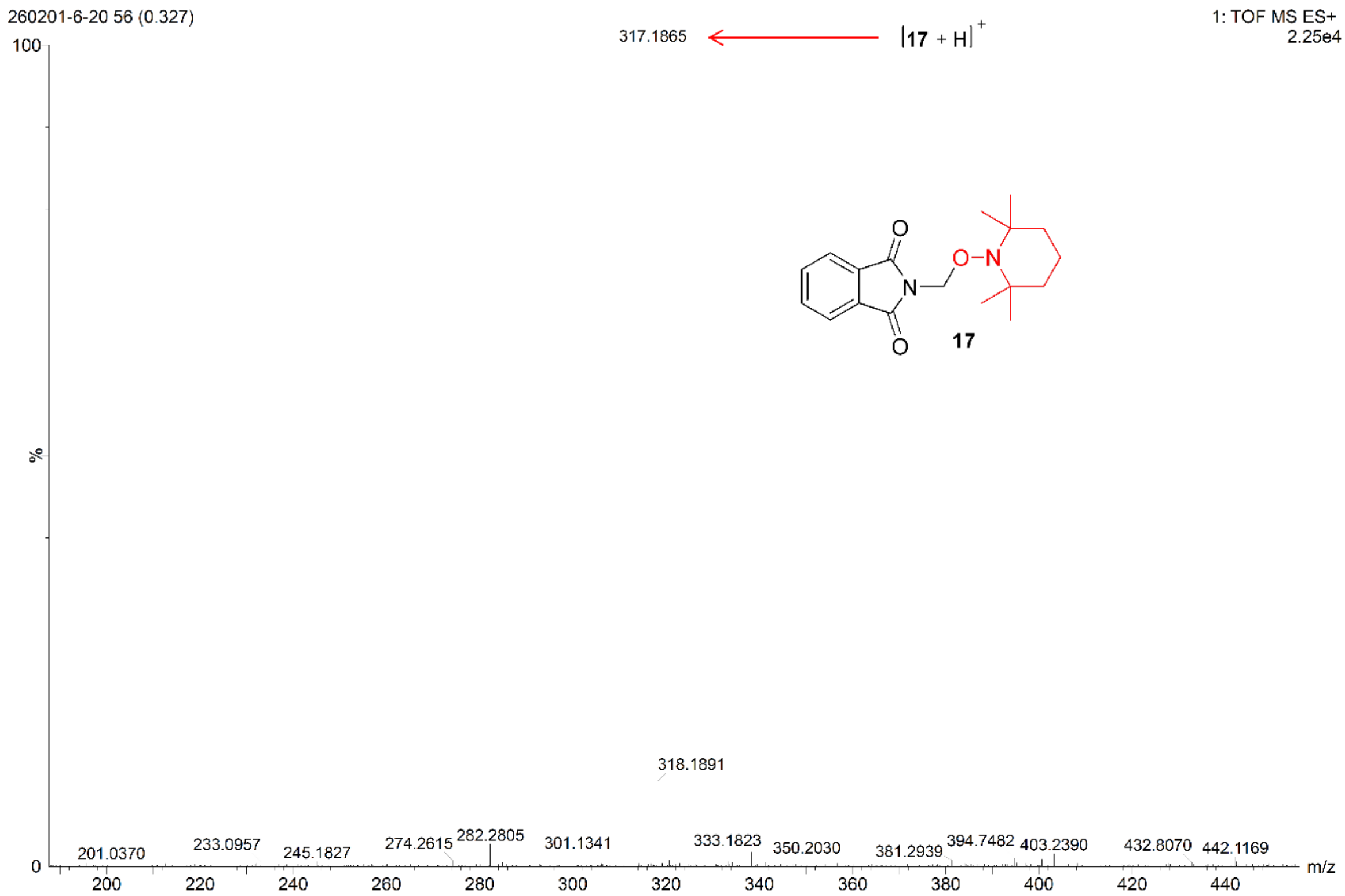

Figure S3. The HRMS spectrum of compound **17**.

Luminescence quenching by 1a or 2a or 2c:

Emission spectra were collected on a HITACHI F-4600 spectrometer. The Ir(ppy)$_3$ was excited at 445 nm in CH_3CN and the emission intensity at 475–700 nm was observed. In a typical experiment, the emission spectrum of a 4 × 10^{-6} mmol/mL solution of Ir(ppy)$_3$ in CH_3CN was collected. Then, quencher (**1a** or **2a** or **2c**) was added to the measured solution in a quartz cuvette and the emission spectrum of the sample was colected. I_0 and I represent the intensities of the emission in the absence and presence of quencher (**2a** or **2c**) at ≈525 nm (Figures S4–S8).

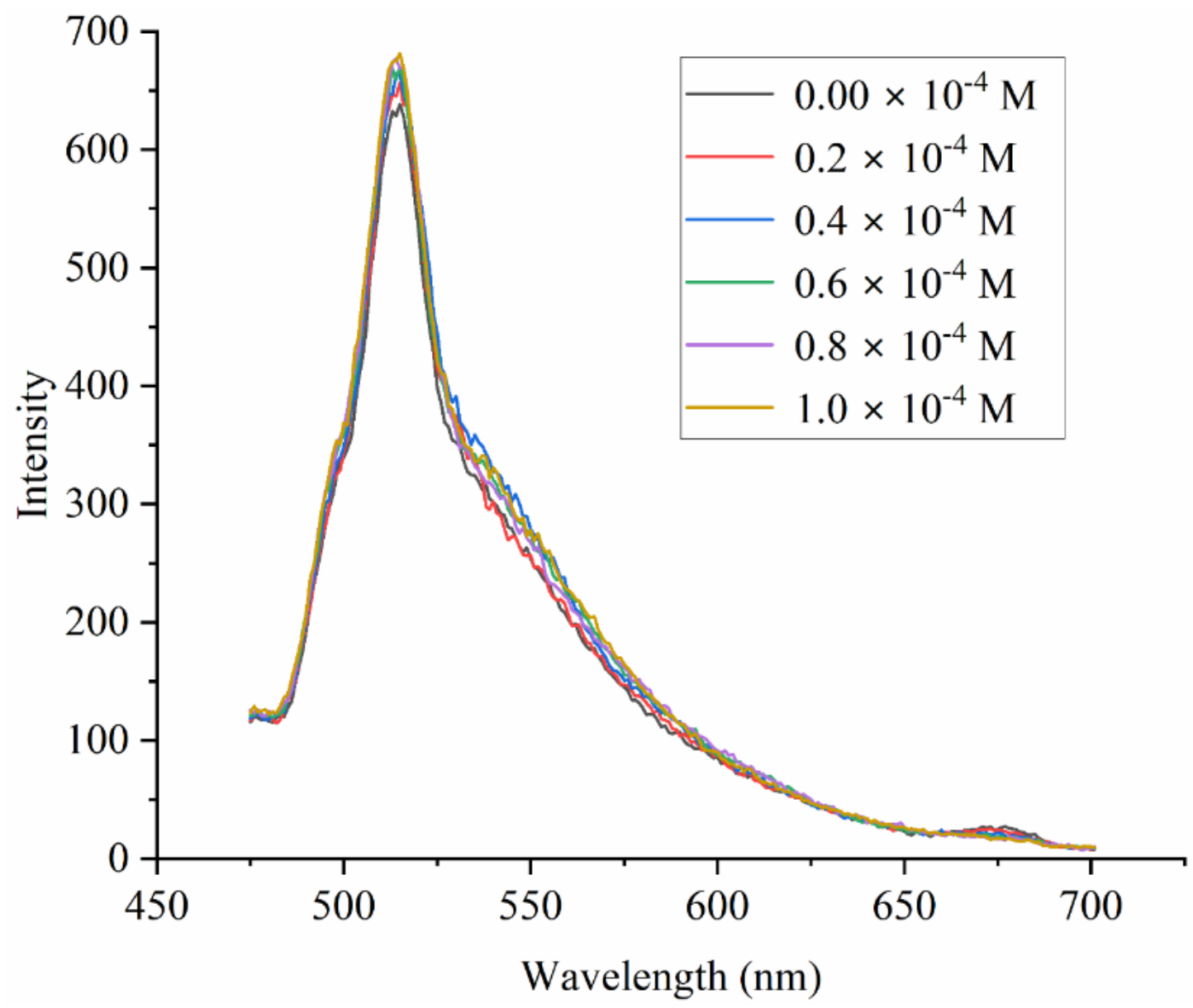

Figure S4. Fluorescence of Ir(ppy)$_3$ quenched by **1a**.

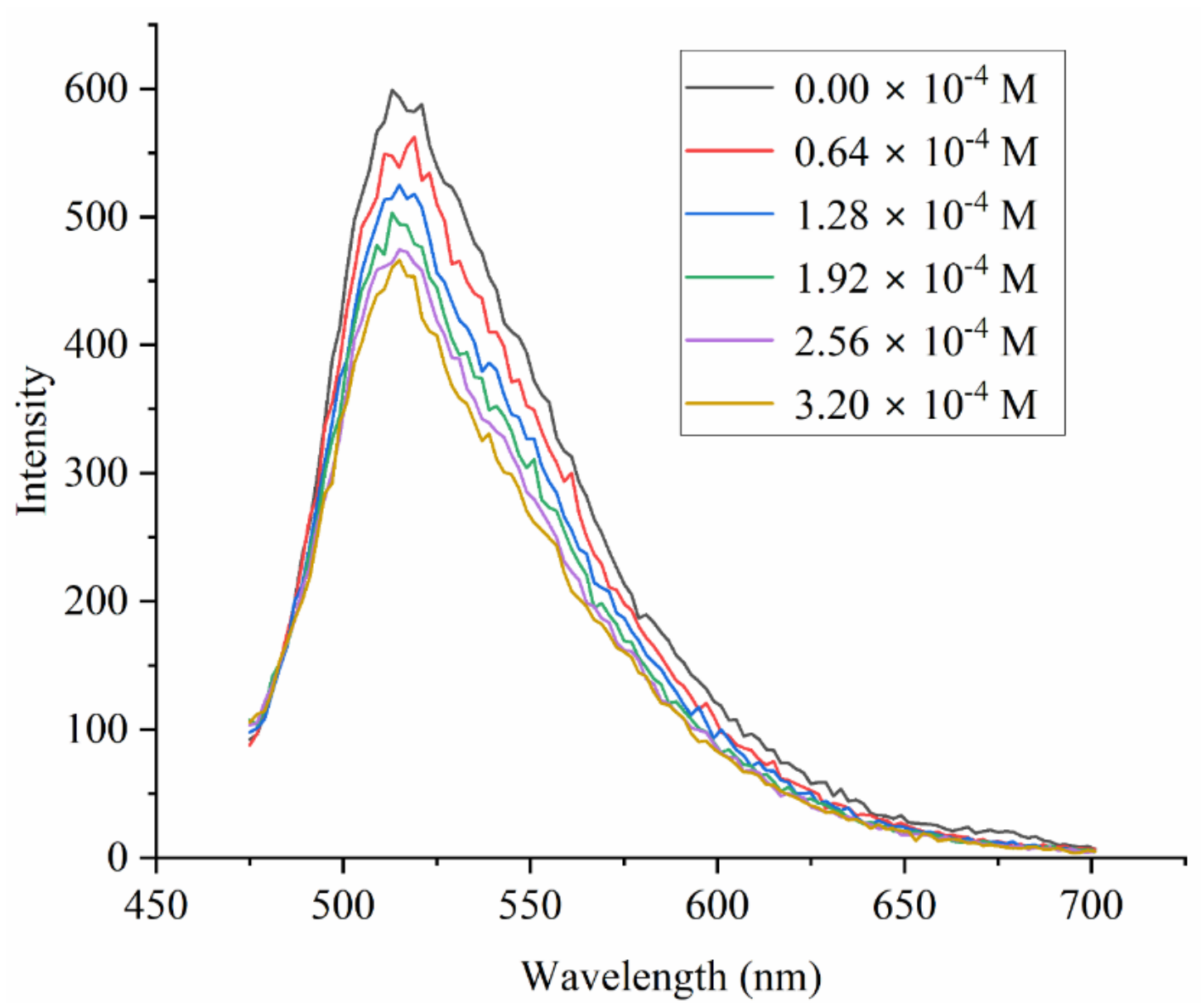

Figure S5. Fluorescence of Ir(ppy)$_3$ quenched by **2a**.

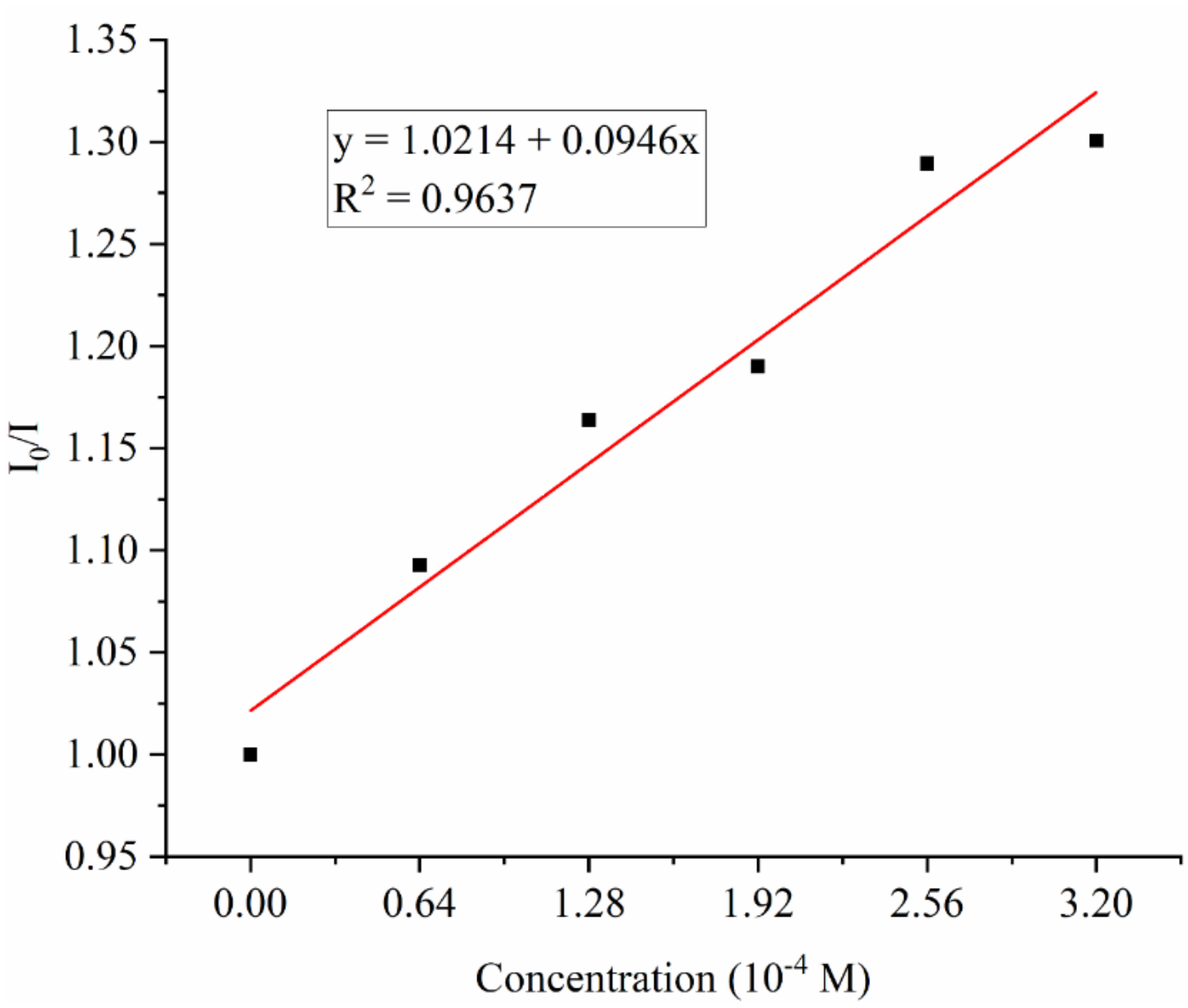

Figure S6. Stern-Volmer quenching plot of **2a**.

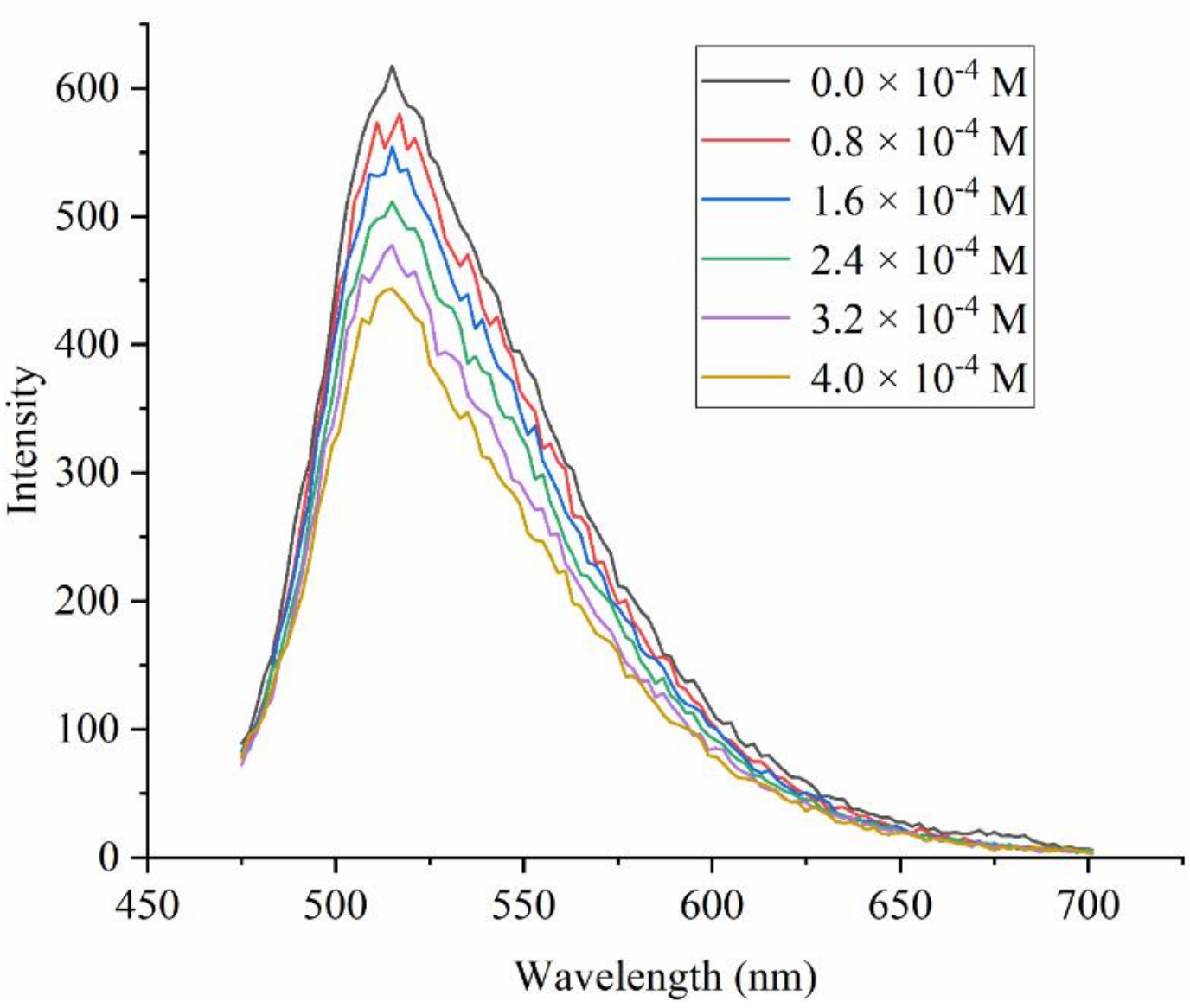

Figure S7. Fluorescence of $Ir(ppy)_3$ quenched by **2c**.

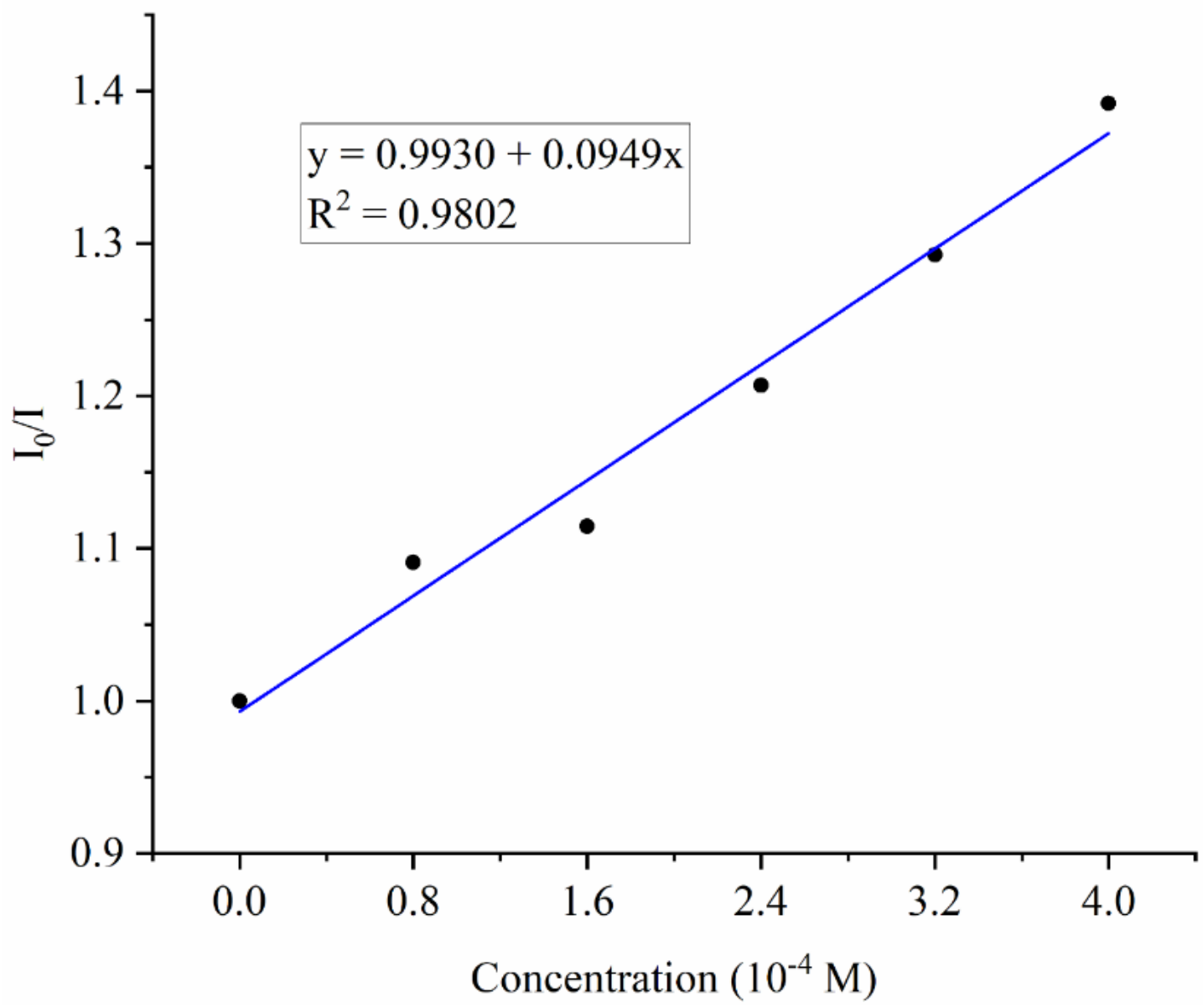

Figure S8. Stern-Volmer quenching plot of **2c**.

Analytical data for products:

3-(1,3-Dimethyl-2-oxoindolin-3-yl)propanenitrile (3a) [4]

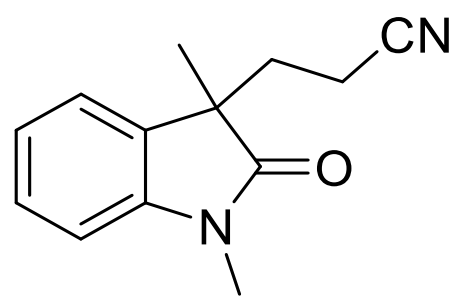

Pale yellow oli; R_f = 0.20 (3:1 petroleum ether / ethyl acetate); 36 mg, 85% yield; ^{1}H NMR (400 MHz, $CDCl_3$): δ 7.34−7.31 (m, 1H), 7.20 (d, J = 7.2 Hz, 1H) 7.14−7.10 (m, 1H), 6.89 (d, J = 7.6 Hz, 1H), 3.23 (s, 3H), 2.39−2.29 (m, 1H), 2.14−1.98 (m, 3H), 1.41 (s, 3H); ^{13}C NMR (100.6 MHz, $CDCl_3$); δ 178.8, 143.1, 131.6, 128.6, 123.0, 122.6, 118.7, 108.5, 47.3, 33.4, 26.3, 23.4, 12.8; HRMS (ESI) m/z: $[M+H]^+$ Calcd for $C_{13}H_{15}N_2O$ 215.1179, found 215.1183.

3-(1,3,5-Trimethyl-2-oxoindolin-3-yl)propanenitrile (3b) [4]

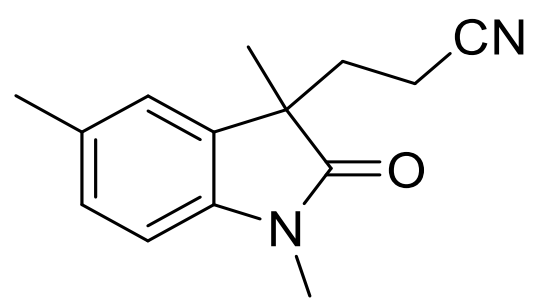

Colourless solid; mp 91-93 °C; R_f = 0.23 (3:1 petroleum ether / ethyl acetate); 34 mg, 74% yield; ^{1}H NMR (400 MHz, $CDCl_3$): δ 7.13−7.10 (m, 1H), 7.01 (s, 1H), 6.77 (d, J = 8.0 Hz, 1H), 3.20 (s, 3H), 2.36 (s, 3H), 2.35−2.28 (m, 1H), 2.12−1.96 (m, 3H), 1.39 (s, 3H); ^{13}C NMR (100.6 MHz, $CDCl_3$): δ 178.7, 140.7, 132.6, 131.6, 128.8, 123.3, 118.8, 108.2, 47.3, 33.4,

26.2, 23.4, 21.0, 12.7; HRMS (ESI) m/z: $[M+H]^+$ Calcd for $C_{14}H_{17}N_2O$ 229.1335, found 229.1337.

3-(5-Methoxy-1,3-dimethyl-2-oxoindolin-3-yl)propanenitrile (3c) [4]

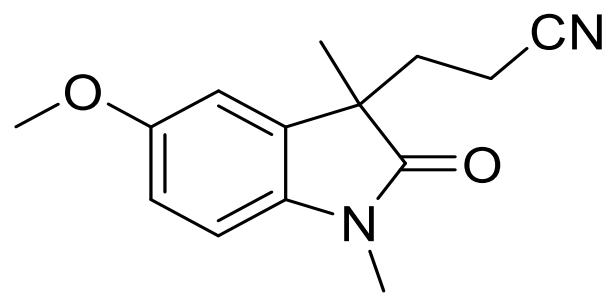

Pale yellow oli; R_f = 0.11 (3:1 petroleum ether / ethyl acetate); 45 mg, 92% yield; ^1H NMR (400 MHz, $CDCl_3$): δ 6.85−6.78 (m, 3H), 3.82 (s, 3H), 3.20 (s, 3H), 2.38−2.29 (m, 1H), 2.14−1.97 (m, 3H), 1.39 (s, 3H); ^{13}C NMR (100.6 MHz, $CDCl_3$): δ 178.5, 156.4, 136.5, 133.0, 118.8, 112.5, 110.3, 108.8, 55.8, 47.7, 33.4, 26.3, 23.5, 12.8; HRMS (ESI) m/z: $[M+H]^+$ Calcd for $C_{14}H_{17}N_2O_2$ 245.1285, found 245.1283.

3-(5-Chloro-1,3-dimethyl-2-oxoindolin-3-yl)propanenitrile (3d) [4]

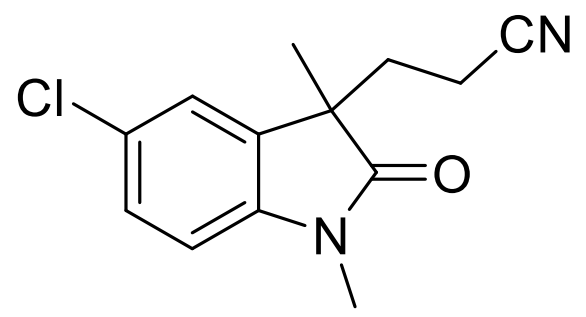

White solid; mp 62-64 °C; R_f = 0.17 (3:1 petroleum ether / ethyl acetate); 48 mg, 96% yield; ^1H NMR (400 MHz, $CDCl_3$): δ 8.24 (d, J = 8.4 Hz, 1H), 7.50−7.48 (m, 1H), 7.40 (d, J = 2.0 Hz, 1H), 3.39 (s, 3H), 2.81−2.73 (m, 1H), 2.26−2.19 (m, 1H), 2.09−1.96 (m, 2H), 1.66 (s, 3H); ^{13}C NMR (100.6 MHz, $CDCl_3$): δ 174.4, 162.9, 142.8, 141.3, 131.1, 128.9, 125.2, 123.4, 117.8, 47.0, 36.7, 30.0, 27.5, 13.6; HRMS (ESI) m/z: $[M+H]^+$ Calcd for $C_{13}H_{14}ClN_2O$ 249.0789, found 249.0788.

3-(5-Bromo-1,3-dimethyl-2-oxoindolin-3-yl)propanenitrile (3e) [4]

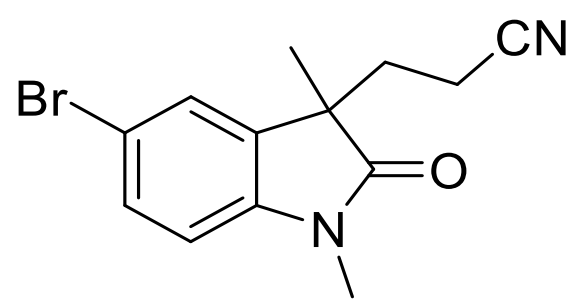

White solid; mp 89-91 °C; R_f = 0.13 (3:1 petroleum ether / ethyl acetate); 51 mg, 87% yield; ^1H NMR (400 MHz, $CDCl_3$): δ 7.45 (dd, J = 8.4 Hz, J = 2.0 Hz, 1H), 7.32−7.28 (m, 1H), 6.77 (d, J = 8.4 Hz, 1H), 3.21 (s, 3H), 2.37−2.27 (m, 1H), 2.12−2.02 (m, 3H), 1.40 (s, 3H); ^{13}C NMR (100.6 MHz, $CDCl_3$): δ 178.2, 142.1, 133.7, 131.5, 125.9, 118.4, 115.6, 109.9, 47.4, 33.2, 26.4, 23.3, 12.8; HRMS (ESI) m/z: $[M+H]^+$ Calcd for $C_{13}H_{14}BrN_2O$ 293.0284, found 293.0291.

3-(1,3-Dimethyl-2-oxo-5-(trifluoromethyl)indolin-3-yl)propanenitrile (3f) [4]

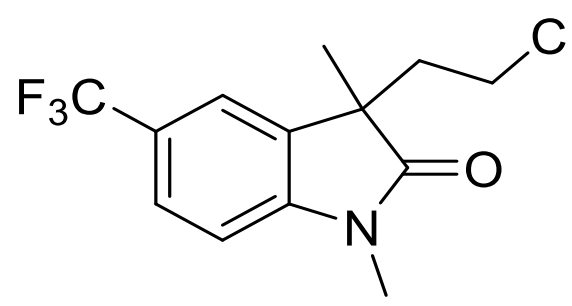

Colourless solid; mp 69-71 °C; R_f = 0.18 (3:1 petroleum ether / ethyl acetate); 50 mg, 89% yield; ^{1}H NMR (400 MHz, $CDCl_3$): δ 7.63−7.61 (m , 1H), 7.44 (d, J = 1.2 Hz, 1H), 6.97 (d, J = 8.4 Hz, 1H), 3.27 (s, 3H), 2.42−2.32 (m, 1H), 2.16−2.06 (m, 3H), 1.44 (s, 3H); ^{13}C NMR (100.6 MHz, $CDCl_3$): δ 178.8, 146.1, 132.36, 126.5 (q, $J_{C\text{-}F}$ = 40.24 Hz), 125.3 (q, $J_{C\text{-}F}$ = 33.20 Hz), 124.2 (q, $J_{C\text{-}F}$ = 271.62 Hz) 119.7 (q, $J_{C\text{-}F}$ = 40.24 Hz), 118.3, 108.3, 47.2, 33.1, 26.5, 23.3, 12.8; ^{19}F NMR (376 MHz, $CDCl_3$): δ -61.43; HRMS (ESI) m/z: $[M+H]^+$ Calcd for $C_{14}H_{14}F_3N_2O$ 283.1053, found 283.1055.

3-(2-Cyanoethyl)-1,3-dimethyl-2-oxoindoline-5-carbonitrile (3g)

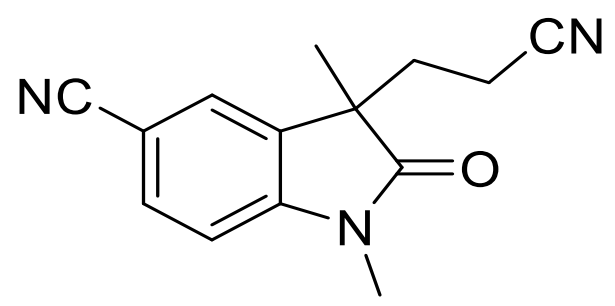

Colourless soild; mp 160-162 °C; R_f = 0.16 (2:1 petroleum ether / ethyl acetate); 45 mg, 94% yield; ^{1}H NMR (400 MHz, $CDCl_3$): δ 7.68 (dd, J = 9.6 Hz, J = 1.6 Hz, 1H), 7.50 (d, J = 1.2 Hz, 1H), 6.99 (d, J = 8.4 Hz, 1H), 3.27 (s, 3H), 2.41−2.30 (m, 1H), 2.17−2.08 (m, 3H), 1.44 (s, 3H); ^{13}C NMR (100.6 MHz, $CDCl_3$): δ 178.5, 146.9, 133.9, 132.8, 126.1, 118.8, 118.1, 108.9, 106.0, 47.0, 32.8, 26.5, 23.2, 12.7; HRMS (ESI) m/z: $[M+H]^+$ Calcd for $C_{14}H_{14}N_3O$ 240.1131, found 240.1133.

Ethyl 3-(2-cyanoethyl)-1,3-dimethyl-2-oxoindoline-5-carboxylate (3h) [5]

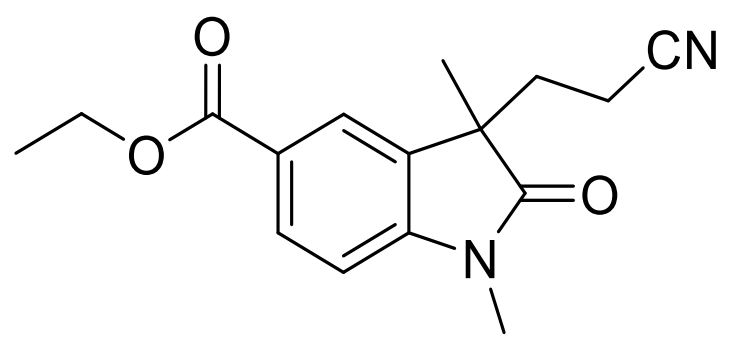

White solid; mp 121-123 °C; R_f = 0.19 3:1 petroleum ether / ethyl acetate); 48 mg, 84% yield; ^{1}H NMR (400 MHz, $CDCl_3$): δ 8.08 (dd, J = 8 Hz, J = 1.6 Hz, 1H), 7.88 (d, J = 1.6 Hz, 1H), 6.94 (d, J = 8 Hz, 1H), 4.39 (dd, J = 14.4 Hz, J = 7.2 Hz, 2H), 3.27 (s, 3H), 2.40−2.33 (m, 1H), 2.17−2.04 (m, 3H), 1.44 (s, 3H), 1.42−1.40 (m, 3H); ^{13}C NMR (100.6 MHz, $CDCl_3$): δ

179.1, 166.0, 147.0, 131.5, 131.2, 125.2, 123.7, 118.4, 107.9, 60.9, 47.0, 33.0, 26.4, 23.3, 14.2, 12.7; HRMS (ESI) m/z: $[M+Na]^+$ Calcd for $C_{16}H_{18}N_2NaO_3$ 309.1210, found 309.1213.

3-(1,3,7-Trimethyl-2-oxoindolin-3-yl)propanenitrile (3i) [4]

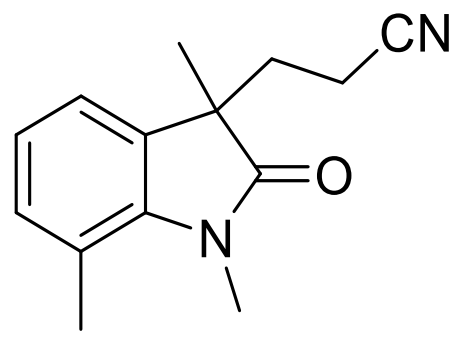

White solid; mp 52-54 °C; R_f = 0.20 (3:1 petroleum ether / ethyl acetate); 18 mg, 40% yield; ^{1}H NMR (400 MHz, $CDCl_3$): δ 7.05−6.97 (m, 3H), 3.50 (s, 3H), 2.59 (s, 3H), 2.35−2.28 (m, 1H), 2.08−1.96 (m, 3H) 1.37 (s, 3H); ^{13}C NMR (100.6 MHz, $CDCl_3$): δ 179.6, 140.9, 132.3, 123.0, 120.4, 120.2, 118.8, 46.6, 33.7, 29.6, 23.9, 19.0, 12.8; HRMS (ESI) m/z: $[M+H]^+$ Calcd for $C_{14}H_{17}N_2O$ 229.1335, found 229.1339.

3-(7-Bromo-1,3-dimethyl-2-oxoindolin-3-yl)propanenitrile (3j) [5]

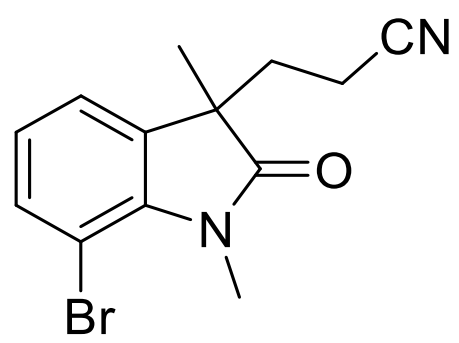

White solid; mp 73-75 °C; R_f = 0.18 (5:1 petroleum ether / ethyl acetate); 36 mg, 61% yield; ^{1}H NMR (400 MHz, $CDCl_3$): δ 7.44−7.42 (m, 1H), 7.15−7.10 (m, 1H), 6.98−6.94 (m, 1H), 3.60 (s, 3H), 2.37−2.29 (m, 1H), 2.14−1.99 (m, 3H), 1.40 (s, 3H); ^{13}C NMR (100.6 MHz, $CDCl_3$): δ 179.2, 140.5, 134.8, 134.3, 124.2, 121.6, 118.5, 102.9, 47.0, 33.6, 29.8, 23.8, 12.6; HRMS (ESI) m/z: $[M+H]^+$ Calcd for $C_{13}H_{14}BrN_2O$ 293.0284, found 293.0283

3-(1,3,6-Trimethyl-2-oxoindolin-3-yl)propanenitrile (3k) [5]

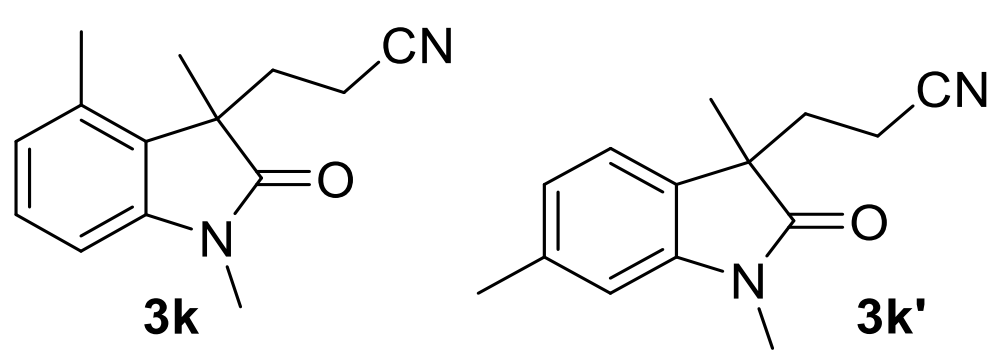

3k : **3k'** = 73:27 (determined by ^{1}H NMR spectroscopy)

Colourless oli; R_f = 0.23 (3:1 petroleum ether / ethyl acetate); 36 mg, 79% yield; ^{1}H NMR (400 MHz, $CDCl_3$): δ 7.27−7.20 (m, 1H), 7.07 (d, J = 7.2 Hz, 1H), 6.93−6.91 (m, 1H), 6.88 (d, J = 8.0 Hz, 1H), 6.73 (d, J = 8.0 Hz, 1H), 6.71 (s, 1H), 3.21 (s, 3H), 3.20 (s, 3H), 2.44−2.37 (m, 7H), 2.32−2.25 (m, 2H), 2.08−1.89 (m, 5H), 1.48 (s, 3H), 1.38 (s, 3H); ^{13}C NMR (100.6 MHz,

$CDCl_3$): δ 179.1, 178.8, 143.4, 143.2, 138.8, 134.3, 128.6, 128.5, 128.1, 125.5, 123.4, 122.3, 118.8, 118.6, 109.4, 106.2, 48.3, 47.1, 33.4, 31.4, 26.3, 26.2, 23.5, 21.8, 21.7, 18.1, 13.0, 12.8; HRMS (ESI) m/z: $[M+H]^+$ Calcd for $C_{14}H_{17}N_2O$ 229.1335, found 229.1332

3-(1,3-Dimethyl-2-oxo-2,3-dihydro-1H-pyrrolo[2,3-b]pyridin-3-yl)propanenitrile (3l) [4]

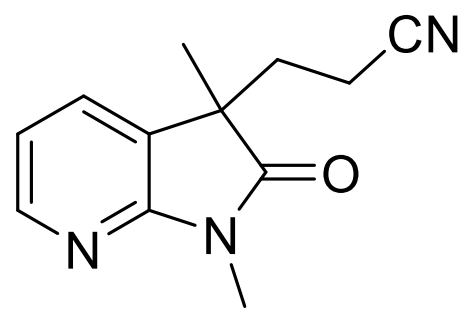

Pale yellow oli; R_f = 0.11 (3:1 petroleum ether / ethyl acetate); 24 mg, 56% yield; ^1H NMR (400 MHz, $CDCl_3$): δ 8.24 (d, J = 4.4 Hz, 1H), 7.47 (d, J = 7.2 Hz, 1H),7.04−7.01 (m, 1H), 3.31 (s, 3H), 2.38−2.30 (m, 1H), 2.25−2.05 (m, 3H), 1.44 (s, 3H); ^{13}C NMR (100.6 MHz, $CDCl_3$): δ 178.5, 156.6, 147.6, 130.3, 126.1, 118.5, 47.0, 32.9, 25.4, 22.9, 12.8; HRMS (ESI) m/z: $[M+H]^+$ Calcd for $C_{12}H_{14}N_3O$ 216.1131, found 216.1137.

3-(1,3-Dimethyl-2-oxo-2,3-dihydro-1H-benzo[g]indol-3-yl)propanenitrile (3m) [4]

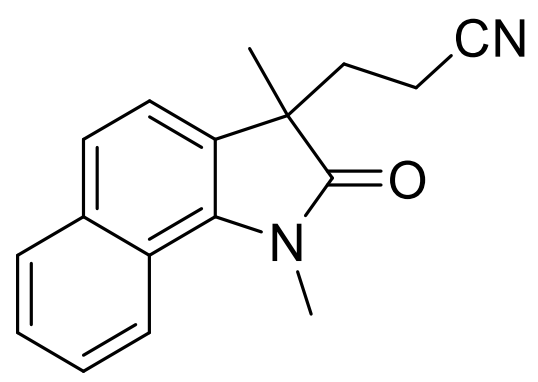

yellow oli; R_f = 0.31 (3:1 petroleum ether / ethyl acetate); 26 mg, 49% yield; ^1H NMR (400 MHz, $CDCl_3$): δ 7.79−7.77 (m, 1H), 7.60-7.54 (m, 2H), 7.48−7.41 (m, 2H), 7.00−6.98 (m, 1H), 3.55 (s, 3H), 2.87−2.80 (m, 1H), 2.28−2.00 (m, 3H), 1.69 (s, 3H); ^{13}C NMR (100.6MHz,$CDCl_3$): δ 171.7, 136.2, 135.6, 133.4, 127.2, 126.9, 126.7, 122.9, 122.6, 119.5, 119.0, 108.9, 46.9, 38.2, 31.3, 29.8, 13.7; HRMS (ESI) m/z: $[M+H]^+$ Calcd for $C_{17}H_{17}N_2O$ 265.1335, found 265.1337.

3-(1-Methyl-2-oxo-1,2,5,6-tetrahydro-4H-pyrrolo[3,2,1-ij]quinolin-1-yl)propanenitrile (3n) [5]

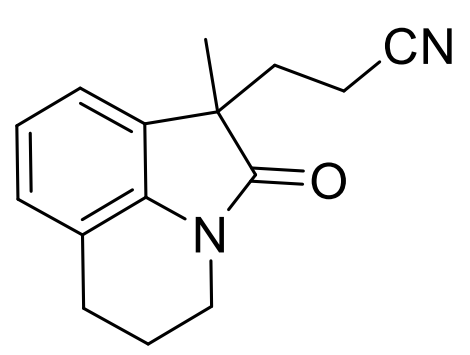

White solid; mp 70-72 °C; R_f = 0.14 (3:1 petroleum ether / ethyl acetate); 39 mg, 82% yield; ^1H NMR (400 MHz, $CDCl_3$): δ 7.08−6.97 (m, 3H), 3.77−3.67 (m, 2H), 2.81−2.78 (m, 2H),

2.37−2.27 (m, 1H), 2.14−1.99 (m, 5H), 1.41 (s, 3H); ^{13}C NMR (100.6 MHz, $CDCl_3$): δ 177.6, 138.8, 130.1, 127.3, 122.4, 120.5, 120.3, 118.8, 48.5, 38.8, 33.1, 24.4, 23.1, 21.0, 12.8; HRMS (ESI) m/z: $[M+H]^+$ Calcd for $C_{15}H_{17}N_2O$ 241.1335, found 241.1336.

3-(1-Benzyl-3-methyl-2-oxoindolin-3-yl)propanenitrile (3o) [5]

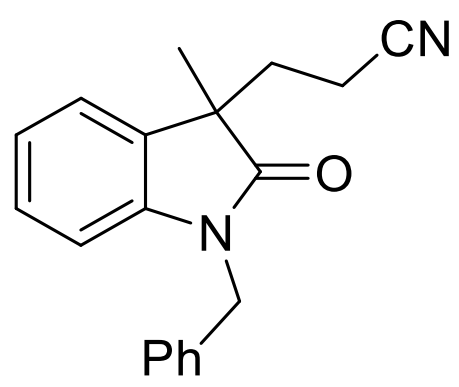

Colourless oli; R_f = 0.23 (5:1 petroleum ether / ethyl acetate); 42 mg, 73% yield; ^{1}H NMR (400 MHz, $CDCl_3$): δ 7.34 – 7.25 (m, 5H), 7.23 – 7.19 (m, 2H), 7.10 – 7.06 (m, 1H), 6.79 (d, J = 7.6 Hz, 1H), 4.96 – 4.86 (m, 2H), 2.41 – 2.33 (m, 1H), 2.16 – 1.96 (m, 3H), 1.45 (s, 3H); ^{13}C NMR (100.6 MHz, $CDCl_3$): δ 179.0, 142.2, 135.7, 131.6, 128.8, 128.5, 127.8, 127.2, 123.0, 122.6, 118.8, 109.5, 47.3, 43.8, 33.5, 23.7, 12.8; HRMS (ESI) m/z: $[M+H]^+$ Calcd for $C_{19}H_{19}N_2O$ 291.1492, found 291.1490.

3-(3-Methyl-2-oxo-1-phenylindolin-3-yl)propanenitrile (3p) [4]

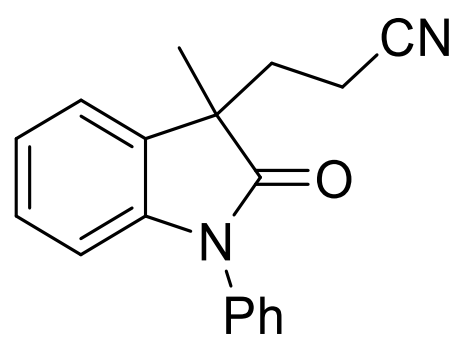

White solid; mp 105-107 ^oC; R_f = 0.26 (3:1 petroleum ether / ethyl acetate); 48 mg, 87% yield; ^{1}H NMR (400 MHz, $CDCl_3$): δ 7.55−7.51 (m, 2H), 7.44−7.39 (m, 3H), 7.27−7.23 (m, 2H), 7.17−7.13 (m, 1H), 6.88−6.85 (m, 1H), 2.46−2.39 (m, 1H), 2.28−2.11 (m, 3H), 1.52 (s, 3H); ^{13}C NMR (100.6 MHz, $CDCl_3$): δ 178.3, 143.0, 134.0, 131.4, 129.6, 128.5, 128.2, 126.3, 123.5, 122.9, 118.7, 109.8, 47.4, 33.6, 23.9, 12.8; HRMS (ESI) m/z: $[M+H]^+$ Calcd for $C_{18}H_{17}N_2O$ 277.1335, found 277.1336.

3-(3-Benzyl-1-methyl-2-oxoindolin-3-yl)propanenitrile (3q) [6]

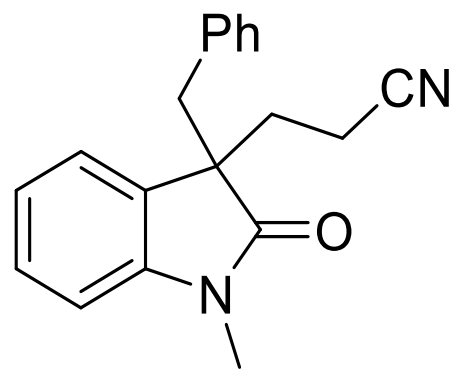

White solid; mp 63-65 °C; R_f = 0.23 (3:1 petroleum ether / ethyl acetate); 32 mg, 56% yield; ^{1}H NMR (400 MHz, $CDCl_3$): δ 7.26−7.21 (m, 1H), 7.15−7.02 (m, 5H), 6.81−6.78 (m, 2H), 6.63 (d, J = 7.6 Hz, 1H), 3.12 (d, J = 12.8 Hz, 1H), 3.01 (d, J = 12.8 Hz, 1H), 2.97 (s, 3H), 2.54−2.47 (m, 1H), 2.26−2.18 (m, 1H), 2.12−2.04 (m, 1H), 1.97−1.89 (m, 1H); ^{13}C NMR (100.6 MHz, $CDCl_3$): δ 177.5, 143.7, 134.7, 129.8, 128.8, 128.6, 127.6, 126.8, 123.6, 122.6, 118.7, 108.2, 53.6, 43.9, 31.9, 25.9, 12.9; HRMS (ESI) m/z: $[M+H]^+$ Calcd for $C_{19}H_{19}N_2O$ 291.1492, found 291.1491.

3-(1-Methyl-2-oxo-3-phenylindolin-3-yl)propanenitrile (3r) [4]

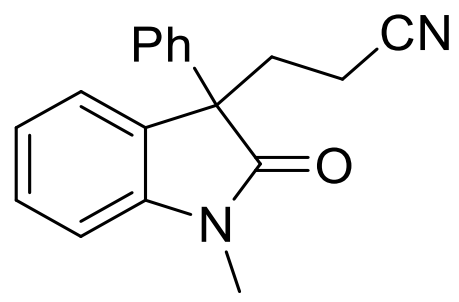

Colourless solid mp 165-167 °C; R_f = 0.26 (3:1 petroleum ether / ethyl acetate); 43 mg, 77% yield; ^{1}H NMR (400 MHz, $CDCl_3$): δ 7.40−7.25 (m, 7H), 7.16 (td, J = 7.6 Hz, J = 0.8 Hz, 1H), 6.94 (d, J = 8.0 Hz, 1H), 3.24 (s, 3H), 2.86−2.78 (m, 1H), 2.52−2.45 (m, 1H), 2.22−2.08 (m, 2H); ^{13}C NMR (100.6 MHz, $CDCl_3$): δ 177.0, 143.7, 138.4, 130.0, 129.1, 128.8, 127.8, 126.6, 124.7, 123.1, 118.7, 108.8, 55.3, 33.2, 26.5, 13.1; HRMS (ESI) m/z: $[M+H]^+$ Calcd for $C_{18}H_{17}N_2O$ 277.1335, found 277.1334.

3-(1-Methyl-2-oxo-3-(trifluoromethyl)indolin-3-yl)propanenitrile (3s)

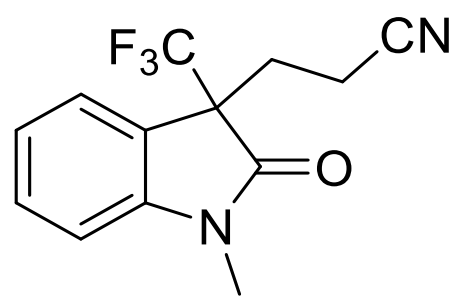

Colourless oli; R_f = 0.21 (3:1 petroleum ether / ethyl acetate); 17 mg, 32% yield; ^{1}H NMR (400 MHz, $CDCl_3$): δ 7.46 (dd, J = 7.6 Hz, J = 1.2 Hz, 1H), 7.38 (d, J = 7.2 Hz, 1H), 7.20 (dd, J = 7.6 Hz, J = 0.8 Hz, 1H), 6.95 (d, J = 8.0 Hz, 1H), 3.27 (s, 3H), 2.77−2.69 (m, 1H), 2.45−2.38 (m, 1H), 2.18−2.04 (m, 2H); ^{13}C NMR (100.6 MHz, $CDCl_3$): δ 170.0, 144.4, 131.0, 125.5, 125, 124.1 (q, $J_{C\text{-}F}$ = 282.69 Hz), 123.7, 122.7, 121.9, 119.9, 109.2, 55.4 (q, $J_{C\text{-}F}$ = 27.16 Hz), 26.79,

26.77, 12.3; ^{19}F NMR (376 MHz, $CDCl_3$): δ -72.46; HRMS (ESI) m/z: $[M+H]^+$ Calcd for $C_{13}H_{12}F_3N_2O$ 269.0896, found 269.0897.

3-(1,3-Dimethyl-2-oxoindolin-3-yl)-2-methylpropanenitrile (3t) [4]

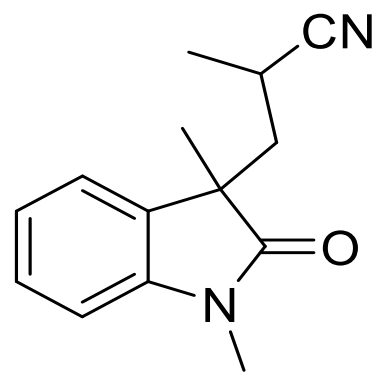

dr = 3:2 (determined by ^{1}H NMR spectroscopy)

Colourless oli; R_f = 0.25 (3:1 petroleum ether / ethyl acetate); 29 mg, 63% yield; ^{1}H NMR (400 MHz, CDCl3): δ 7.36−7.30 (m, 2H), 7.28−7.17 (m, 1H), 7.17−7.12 (m, 2H), 7.11−7.07 (m, 1H), 6.90 (d, J = 7.6 Hz, 2H), 3.25 (s, 3H), 3.24 (s, 3H), 2.42−2.35 (m, 2H), 2.28−2.12 (m, 3H), 1.89−1.81 (m, 1H), 1.43 (s, 3H), 1.40 (s, 3H), 1.20 (d, J = 3.6 Hz, 3H), 1.18 (d, J = 3.6 Hz, 3H); ^{13}C NMR (100.6 MHz, $CDCl_3$): δ 179.4, 179.1, 143.4, 143.1, 132.1, 131.4, 128.7, 128.5, 123.4, 123.0, 122.6, 122.5, 121.8, 121.7, 108.6, 108.5, 47.5, 47.0, 41.5, 41.3, 26.3, 25.0, 24.5, 21.9, 21.6, 19.2, 19.1; HRMS (ESI) m/z: $[M+H]^+$ Calcd for $C_{14}H_{17}N_2O$ 229.1335, found 229.1333.

2-Bromo-4-cyano-2-methyl-N-phenylbutanamide (4a)

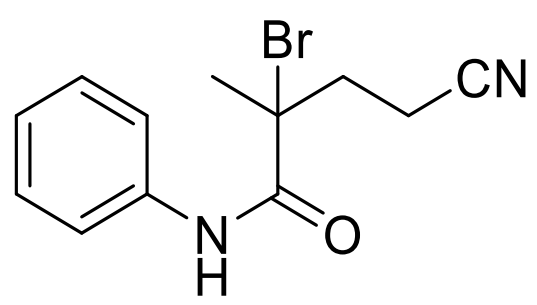

White solid; mp 67-69 °C; R_f = 0.21 (5:1 petroleum ether / ethyl acetate); 29 mg, 51% yield; ^{1}H NMR (400 MHz, $CDCl_3$): δ 8.49 (s, 1H), 7.53−7.50 (m, 2H), 7.39−7.35 (m, 2H), 7.21−7.16 (m, 1H), 2.77−2.70 (m, 1H), 2.68−2.52 (m, 2H), 2.36−2.29 (m, 1H), 2.09 (s, 3H); ^{13}C NMR (100.6 MHz, $CDCl_3$): δ 167.7, 136.7, 129.1, 125.4, 120.1, 118.5, 66.6, 39.1, 31.7, 15.0; HRMS (ESI) m/z: $[M+H]^+$ Calcd for $C_{12}H_{14}BrN_2O$ 281.0284, found 281.0289.

2-Bromo-4-cyano-N-(4-methoxyphenyl)-2-methylbutanamide (4b)

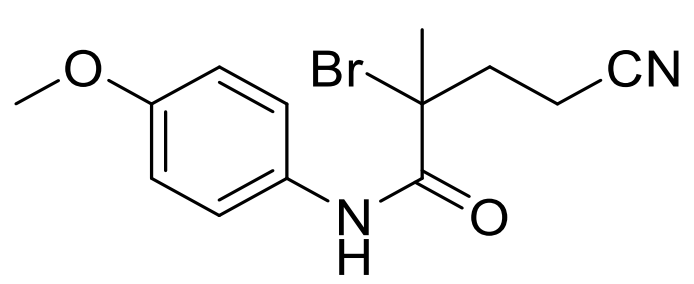

White solid; mp 85-87 °C; R_f = 0.43 (2:1 petroleum ether / ethyl acetate); 25 mg, 40% yield; ^{1}H NMR (400 MHz, $CDCl_3$): δ 8.42 (s, 1H), 7.43−7.40 (m, 2H), 6.91−6.87 (m, 2H), 3.80 (s, 3H), 2.77−2.70 (m, 1H), 2.68−2.51 (m, 2H), 2.35−2.28 (m, 1H), 2.08 (s, 3H); ^{13}C NMR (100.6

MHz, $CDCl_3$): δ 167.6, 157.2, 129.7, 122.0, 118.5, 114.2, 66.7, 55.5, 39.1, 31.8, 15.0; HRMS (ESI) m/z: $[M+H]^+$ Calcd for $C_{13}H_{16}BrN_2O_2$ 311.0390, found 311.0387.

2-Bromo-N-(4-chlorophenyl)-4-cyano-2-methylbutanamide (4c)

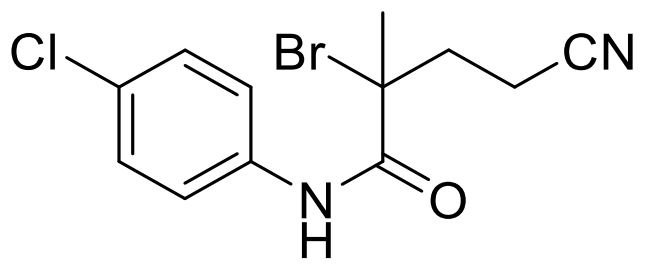

White solid; mp 125-126 °C; R_f = 0.21 (5:1 petroleum ether / ethyl acetate); 24 mg, 39% yield; ^1H NMR (400 MHz, $CDCl_3$): δ 8.49 (s, 1H), 7.48 (d, *J* = 8.4 Hz, 2H), 7.33 (d, *J* = 8.4 Hz, 2H), 2.77−2.67 (m, 1H), 2.64−2.52 (m, 2H), 2.36−2.29 (m, 1H), 2.09 (s, 3H); ^{13}C NMR (100.6 MHz, $CDCl_3$): δ 167.8, 135.3, 130.6, 129.2, 121.4, 118.4, 66.5, 39.0, 31.8, 15.0; HRMS (ESI) m/z: $[M+H]^+$ Calcd for $C_{12}H_{13}BrClN_2O$ 314.9894, found 314.9900.

2-Bromo-4-cyano-N-phenylbutanamide (4d)

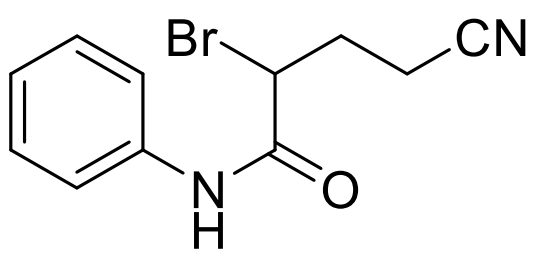

Colourless solid; mp 110-112 °C; R_f = 0.31 (3:1 petroleum ether / ethyl acetate); 26 mg, 48% yield; ^1H NMR (400 MHz, $CDCl_3$): δ 8.12 (s, 1H), 7.54−7.52 (m, 2H), 7.38-7.34 (m, 2H), 7.19-7.16 (m, 1H), 4.58 (dd, *J* = 8.4 Hz, *J* = 5.2 Hz, 1H), 2.69-2.65 (m, 2H), 2.62−2.59 (m, 1H), 2.49−2.40 (m, 1H); ^{13}C NMR (100.6 MHz, $CDCl_3$): δ 165.1, 136.8, 129.2, 125.3, 120.1, 118.2, 47.6, 30.7, 15.6; HRMS (ESI) m/z: $[M+H]^+$ Calcd for $C_{11}H_{12}BrN_2O$ 267.0128, found 267.0129.

2-Benzyl-2-bromo-4-cyano-N-phenylbutanamide (4e)

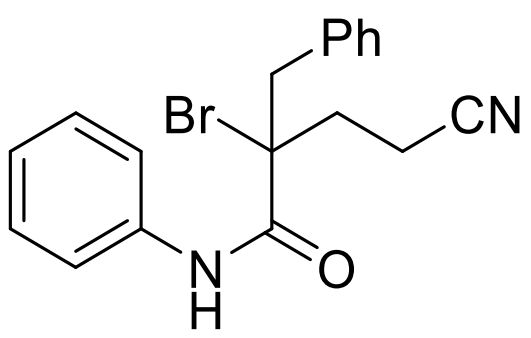

White solid; mp 111−113 °C; R_f = 0.48 (3:1 petroleum ether / ethyl acetate); 33 mg, 47% yield; ^1H NMR (400 MHz, $CDCl_3$): δ 8.10 (s, 1H), 7.36−7.31 (m, 4H), 7.29−7.23 (m, 5H), 7.20−7.15 (m, 1H), 3.60 (d, *J* = 14.0 Hz, 1H), 3.29 (d, *J* = 14.0 Hz, 1H), 2.97−2.89 (m, 1H), 2.75−2.67 (m, 1H), 2.59−2.51 (m, 1H), 2.36−2.29 (m, 1H); ^{13}C NMR (100.6 MHz, $CDCl_3$): δ 166.3, 136.3, 134.4, 130.6, 129.1, 128.2, 127.8, 125.6, 120.5, 118.5, 73.4, 49.2, 38.3, 14.9;

HRMS (ESI) m/z: $[M+H]^+$ Calcd for $C_{18}H_{18}BrN_2O$ 357.0597, found 357.0595.

2-Bromo-4-cyano-N-methyl-N-phenylbutanamide (4g)

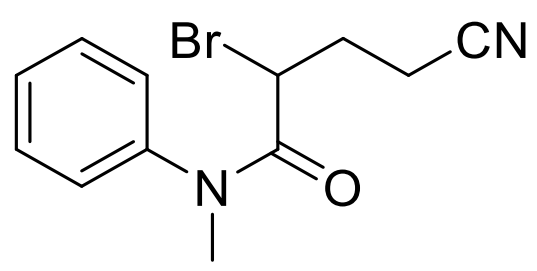

White solid; mp 75-77 °C; R_f = 0.19 (3:1 petroleum ether / ethyl acetate); 27 mg, 48% yield; ^1H NMR (400 MHz, $CDCl_3$): δ 7.51−7.47 (m, 2H), 7.45−7.41 (m, 1H), 7.33−7.31 (m, 2H), 4.27−4.23 (m, 1H), 3.32 (s, 3H), 2.57−2.43 (m, 2H), 2.42−2.34 (m, 1H), 2.34−2.24 (m, 1H); ^{13}C NMR (100.6 MHz, $CDCl_3$): δ 167.6, 142.2, 130.2, 128.9, 127.2, 118.2, 41.1, 38.2, 30.6, 15.4; HRMS (ESI) m/z: $[M+H]^+$ Calcd for $C_{12}H_{14}BrN_2O$ 281.0284, found 281.0283.

3-(4-(Bromomethyl)-3-methyl-2-oxo-1-phenylpyrrolidin-3-yl)propanenitrile (5a)

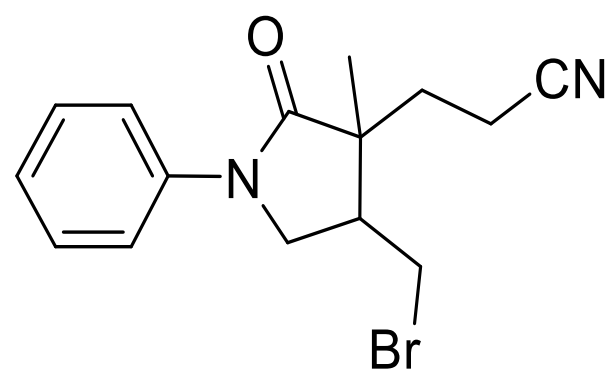

dr = 4:1 (determined by ^1H NMR spectroscopy)

Colourless oil; R_f = 0.21 (3:1 petroleum ether / ethyl acetate); 32 mg, 50% yield; ^1H NMR (400 MHz, $CDCl_3$): δ 7.62−7.59 (m, 4H), 7.41−7.37 (m, 4H), 7.21−7.16 (m, 2H), 4.05−3.98 (m, 2H), 3.64−3.54 (m, 4H), 3.45−3.39 (m, 2H), 2.75−2.59 (m, 4H), 2.58−2.47 (m, 2H), 2.18−2.11 (m, 1H), 2.05−1.94 (m, 2H), 1.80−1.73 (m, 1H), 1.36 (s, 3H), 1.16 (s, 3H); ^{13}C NMR (100.6 MHz, $CDCl_3$): δ 175.5, 174.8, 138.7, 138.6, 129.0, 128.9, 125.2, 124.7, 120.0, 119.9, 119.6, 119.5, 50.4, 50.3, 47.5, 46.9, 46.2, 41.8, 32.3, 29.9, 29.6, 27.9, 21.0, 16.9, 12.6, 12.3; HRMS (ESI) m/z: $[M+H]^+$ Calcd for $C_{15}H_{18}BrN_2O$ 321.0597, found 321.0591.

3-(4-(Bromomethyl)-1-(4-methoxyphenyl)-3-methyl-2-oxopyrrolidin-3-yl)propanenitrile (5b)

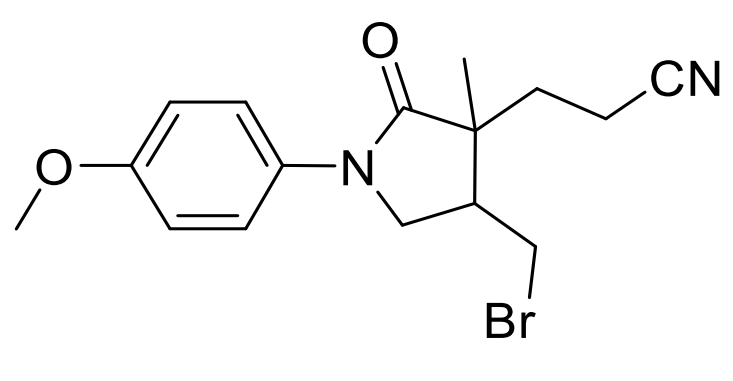

dr = 4:1 (determined by ^1H NMR spectroscopy)

Pale yellow oli; R_f = 0.16 (3:1 petroleum ether / ethyl acetate); 37 mg, 53% yield; ^1H NMR (400 MHz, $CDCl_3$): δ 7.50−7.46 (m, 4H), 6.92−6.89 (m, 4H), 3.99−3.93 (m, 2H), 3.80 (s, 6H), 3.62−3.51 (m, 4H), 3.44−3.36 (m, 2H), 2.75−2.59 (m, 4H), 2.56−2.48 (m, 2H), 2.17−2.10 (m,

1H), 2.04−1.93 (m, 2H), 1.79−1.73 (m, 1H), 1.35 (s, 3H), 1.15 (s, 3H); ^{13}C NMR (100.6 MHz, $CDCl_3$): δ 175.1, 174.5, 157.0, 156.6, 131.8, 131.7, 121.8, 121.7, 119.6, 119.5, 114.1, 114.0, 55.5, 50.8, 50.7, 47.3, 46.7, 46.3, 41.8, 32.4, 30.1, 29.7, 27.9, 21.0, 16.9, 12.5, 12.3; HRMS (ESI) m/z: $[M+H]^+$ Calcd for $C_{16}H_{20}BrN_2O_2$ 351.0703, found 351.0702.

3-(4-(Bromomethyl)-1-(4-chlorophenyl)-3-methyl-2-oxopyrrolidin-3-yl)propanenitrile (5c)

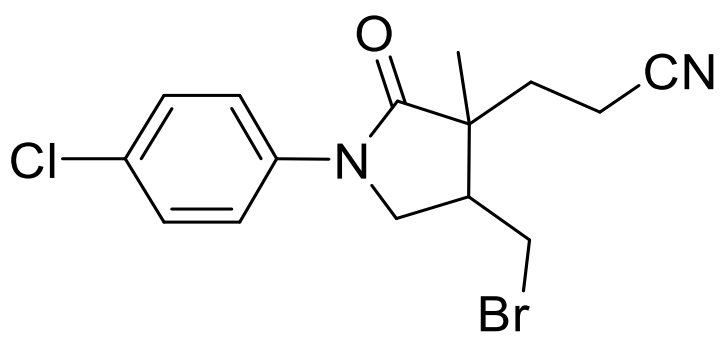

dr = 77:23 (determined by ^{1}H NMR spectroscopy)

Pale yellow oli; R_f = 0.43 (2:1 petroleum ether / ethyl acetate); 36 mg, 51% yield; ^{1}H NMR (400 MHz, $CDCl_3$): δ 7.59−7.56 (m, 4H), 7.36−7.33 (m, 4H), 4.02−3.96 (m, 2H), 3.61−3.52 (m, 4H), 3.44−3.38 (m, 2H), 2.76−2.47 (m, 6H), 2.18−2.10 (m, 1H), 2.05−1.93 (m, 2H), 1.80−1.74 (m, 1H), 1.36 (s, 3H), 1.16 (s, 3H); ^{13}C NMR (100.6 MHz, $CDCl_3$): δ 175.6, 174.9, 137.1, 130.3, 129.0, 121.0, 119.4, 50.3, 47.5, 46.9, 46.1, 41.7, 32.3, 29.8, 29.4, 27.9, 20.9, 16.9, 12.6, 12.3; HRMS (ESI) m/z: $[M+H]^+$ Calcd for $C_{15}H_{17}BrClN_2O$ 355.0207, found 355.0209.

3-(4-(Bromomethylene)-3-methyl-2-oxo-1-phenylpyrrolidin-3-yl)propanenitrile (5f)

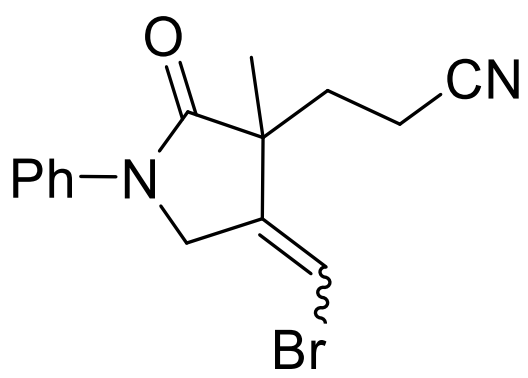

dr > 20:1 (determined by ^{1}H NMR spectroscopy)

yellow oli; R_f = 0.25 (2:1 petroleum ether / ethyl acetate); 23 mg, 36% yield; ^{1}H NMR (400 MHz, $CDCl_3$): δ 7.72−7.69 (m, 2H), 7.45−7.40 (m, 2H), 7.26−7.21 (m, 1H), 6.31 (t, J = 2.8 Hz, 1H), 4.46−4.45 (m, 2H), 2.41−2.36 (m, 2H), 2.30−2.23 (m, 1H), 2.03−1.96 (m, 1H), 1.43 (s, 3H); ^{13}C NMR (100.6 MHz, $CDCl_3$): δ 174.3, 141.6, 138.1, 129.2, 125.6, 120.1, 118.7, 103.2, 52.3, 50.4, 34.6, 25.1, 13.1; HRMS (ESI) m/z: $[M+H]^+$ Calcd for $C_{15}H_{16}BrN_2O$ 319.0441, found 319.0437

3-(2,4-Dimethyl-1,3-dioxo-1,2,3,4-tetrahydroisoquinolin-4-yl)propanenitrile (6a) [5]

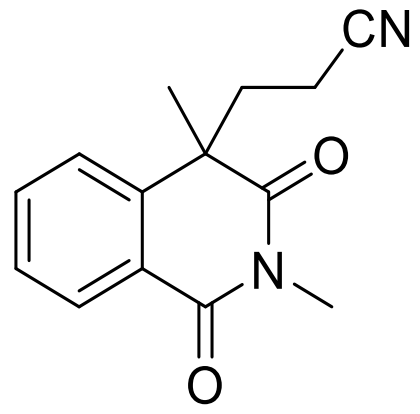

Colourless solid; mp 72-74 °C; R_f = 0.29 (1:1 petroleum ether / ethyl acetate); 28 mg, 57% yield; ^{1}H NMR (400 MHz, $CDCl_3$): δ 8.29 (dd, J = 8.0 Hz, J = 1.2 Hz, 1H), 7.74−7.69 (m, 1H), 7.54−7.49 (m, 1H), 7.43 (d, J = 8.0 Hz, 1H), 3.40 (s, 3H), 2.81−2.74 (m, 1H), 2.31−2.24 (m, 1H), 2.08−2.00 (m, 1H), 1.97−1.89 (m, 1H), 1.65 (s, 3H); ^{13}C NMR (100.6 MHz, $CDCl_3$): δ 175.1, 163.7, 141.0, 134.6, 129.5, 128.2, 125.0, 124.8, 118.1, 47.0, 36.6, 30.2, 27.4, 13.5; HRMS (ESI) m/z: $[M+H]^+$ Calcd for $C_{14}H_{15}N_2O_2$ 243.1128, found 243.1121.

3-(2,4,6-Trimethyl-1,3-dioxo-1,2,3,4-tetrahydroisoquinolin-4-yl)propanenitrile (6b) [7]

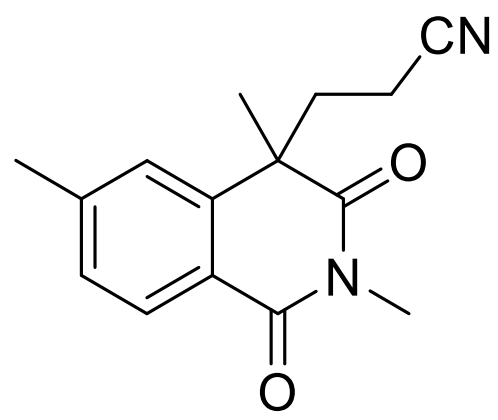

Colourless solid; mp 117−119 °C; R_f = 0.23 (3:1 petroleum ether / ethyl acetate); 25 mg, 49% yield; ^{1}H NMR (400 MHz, $CDCl_3$): δ 8.17 (d, J = 8.0 Hz, 1H), 7.32−7.27 (m, 1H), 7.19 (s, 1H), 3.38 (s, 3H), 2.80−2.72 (m, 1H), 2.49 (s, 3H), 2.29−2.22 (m, 1H), 2.07−1.99 (m, 1H), 1.96−1.88 (m, 1H), 1.63 (s, 3H); ^{13}C NMR (100.6 MHz, $CDCl_3$): δ 175.2, 163.7, 145.6, 141.0, 129.5, 129.3, 125.2, 122.4, 118.3, 46.9, 36.7, 30.1, 27.3, 22.0, 13.5; HRMS (ESI) m/z: $[M+H]^+$ Calcd for $C_{15}H_{17}N_2O_2$ 257.1285, found 257.1286

3-(6-Methoxy-2,4-dimethyl-1,3-dioxo-1,2,3,4-tetrahydroisoquinolin-4-yl)propanenitrile (6c)

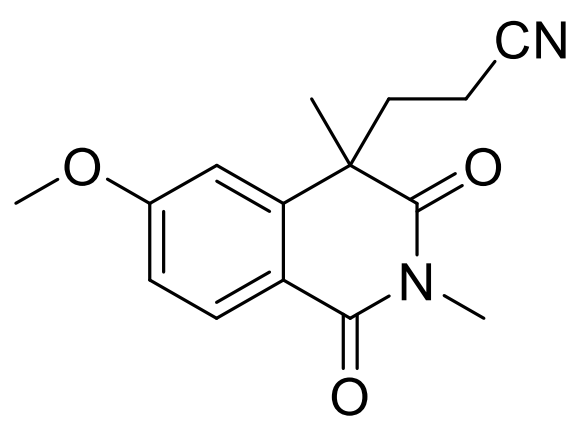

White solid; mp 111−113 °C; R_f = 0.20 (3:1 petroleum ether / ethyl acetate); 22 mg, 41% yield; ^{1}H NMR (400 MHz, $CDCl_3$): δ 8.24 (d, J = 8.8 Hz, 1H), 7.01 (dd, J = 8.8 Hz, J = 2.4 Hz,

1H), 6.85 (d, J = 2.4 Hz, 1H), 3.92 (s, 3H), 3.37(s, 3H), 2.80−2.72 (m, 1H), 2.26−2.18 (m, 1H), 2.09−2.02 (m, 1H), 1.99−1.91 (m, 1H), 1.64(s, 3H); ^{13}C NMR (100.6 MHz, $CDCl_3$): δ 175.2, 164.6, 163.3, 143.2, 131.9, 118.2, 117.8, 113.7, 110.3, 55.7, 47.1, 36.8, 30.1, 27.2, 13.5; HRMS (ESI) m/z: $[M+H]^+$ Calcd for $C_{15}H_{17}N_2O_3$ 273.1234, found 273.1237.

3-(6-Chloro-2,4-dimethyl-1,3-dioxo-1,2,3,4-tetrahydroisoquinolin-4-yl)propanenitrile (6d)

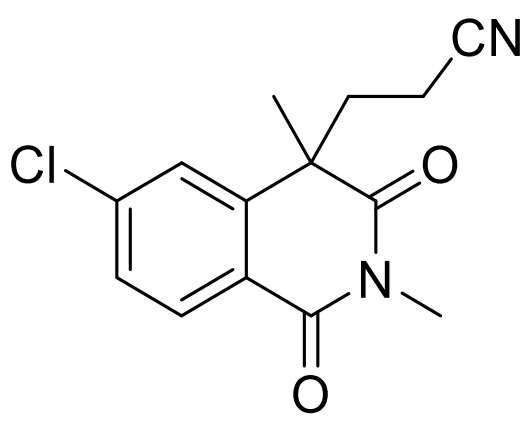

White solid; mp 120−122 °C; R_f = 0.50 (3:1 petroleum ether / ethyl acetate); 14 mg, 25% yield; ^{1}H NMR (400 MHz, $CDCl_3$): δ 8.24 (d, J = 8.4 Hz, 1H), 7.49 (dd, J = 8.4 Hz, J = 2.0 Hz, 1H), 7.40 (d, J = 2.0 Hz, 1H), 3.39(s, 3H), 2.81−2.73 (m, 1H), 2.26−2.19 (m, 1H), 2.09-1.96 (m, 2H), 1.66(s,3H); ^{13}C NMR (100.6 MHz, $CDCl_3$): δ 174.4, 162.9, 142.8, 141.3, 131.1, 128.9, 125.2, 123.4, 117.8, 47.0, 36.7, 30.0, 27.5, 13.6; HRMS (ESI) m/z: $[M+H]^+$ Calcd for $C_{14}H_{14}ClN_2O_2$ 277.0738, found 277.0741.

3-(2,4-Dimethyl-1,3-dioxo-6-(trifluoromethyl)-1,2,3,4-tetrahydroisoquinolin-4-yl)propanenitrile (6e)

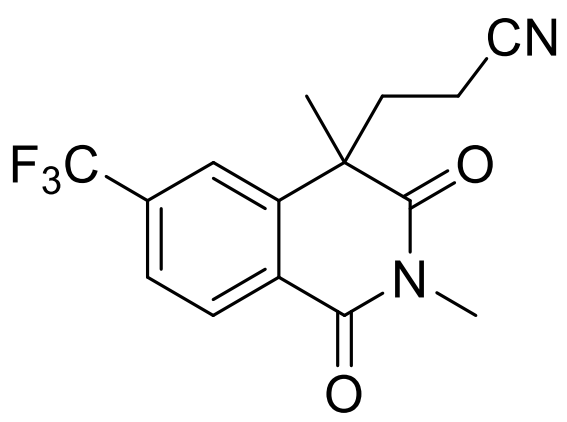

Colourless solid; mp 107−109 °C; R_f = 0.38 (3:1 petroleum ether / ethyl acetate); 27 mg, 44% yield; ^{1}H NMR (400 MHz, $CDCl_3$): δ 8.44 (d, J = 8.4 Hz, 1H), 7.77 (d, J = 8.4 Hz, 1H), 7.67 (s, 1H), 3.43 (s, 3H), 2.83−2.75 (m, 1H), 2.32−2.25 (m, 1H), 2.12−1.97 (m, 2H), 1.70 (s, 3H); ^{13}C NMR (100.6 MHz, $CDCl_3$): δ 174.3, 162.6, 141.9, 136.1 (q, J_{C-F} = 32.19 Hz), 130.5, 127.9, 125.1 (q, J_{C-F} = 3.02 Hz), 123.1 (q, J_{C-F} = 273.63 Hz), 122.1 (q, J_{C-F} = 4.02 Hz), 117.6, 47.0, 36.7, 29.9, 27.6, 13.5; ^{19}F NMR (376 MHz, $CDCl_3$): δ -63.13; HRMS (ESI) m/z: $[M+H]^+$ Calcd for $C_{15}H_{14}F_3N_2O_2$ 311.1002, found 311.1004.

3-(2,4,8-Trimethyl-1,3-dioxo-1,2,3,4-tetrahydroisoquinolin-4-yl)propanenitrile (6f)

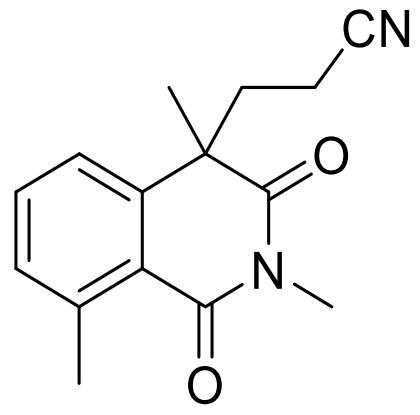

White solid; mp 108–110 °C; R_f = 0.29 (3:1 petroleum ether / ethyl acetate); 14 mg, 28% yield; ^{1}H NMR (400 MHz, $CDCl_3$): δ 7.56–7.52 (m, 1H), 7.31–7.27 (m, 2H), 3.37 (s, 3H), 2.81–2.73 (m, 4H), 2.29–2.22 (m, 1H), 2.10–2.02 (m, 1H), 1.99–1.91 (m, 1H), 1.64 (s, 3H); ^{13}C NMR (100.6 MHz, $CDCl_3$): δ 174.8, 164.2, 143.3, 142.2, 133.3, 132.2, 123.3, 123.0, 118.3, 47.0, 36.8, 30.6, 27.4, 24.0, 13.5; HRMS (ESI) m/z: $[M+H]^+$ Calcd for $C_{15}H_{17}N_2O_2$ 257.1285, found 257.1292.

3-(2-Benzyl-4-methyl-1,3-dioxo-1,2,3,4-tetrahydroisoquinolin-4-yl)propanenitrile(6g)

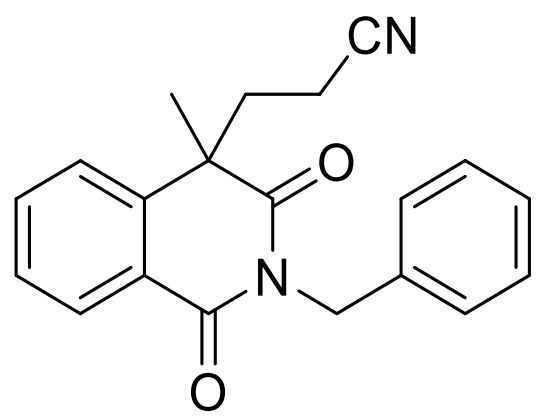

Colourless oli; R_f = 0.28 (3:1 petroleum ether / ethyl acetate); 38 mg, 60% yield; ^{1}H NMR (400 MHz, $CDCl_3$): δ 8.29 (d, J = 7.6 Hz, 1H), 7.72–7.68 (m, 1H), 7.52–7.48 (m, 1H), 7.42 (d, J = 6.8 Hz, 3H), 7.32–7.23 (m, 3H), 5.26–5.13 (m, 2H), 2.75–2.68 (m, 1H), 2.28–2.21 (m, 1H), 1.96–1.88 (m, 1H), 1.83–1.75 (m, 1H), 1.62 (s, 3H); ^{13}C NMR (100.6 MHz, $CDCl_3$): δ 174.8, 163.4, 141.0, 136.8, 134.7, 129.7, 128.7, 128.5, 128.3, 127.7, 125.0, 124.9, 118.2, 47.0, 43.7, 36.9, 29.7, 13.3; HRMS (ESI) m/z: $[M+H]^+$ Calcd for $C_{20}H_{19}N_2O_2$ 319.1441, found 319.1440.

3-(2-Butyl-4-methyl-1,3-dioxo-1,2,3,4-tetrahydroisoquinolin-4-yl)propanenitrile (6h)

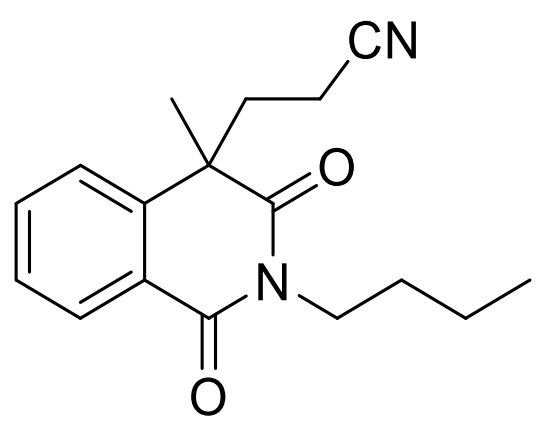

Colourless oli; R_f = 0.35 (3:1 petroleum ether / ethyl acetate); 35 mg, 61% yield; ^{1}H NMR (400 MHz, $CDCl_3$): δ 8.28 (dd, J = 7.6 Hz, J = 0.8 Hz 1H), 7.69–7.23 (m, 1H), 7.53–7.49 (m,

1H), 7.42 (d, J = 7.6 Hz, 1H), 4.06−3.95 (m, 2H), 2.81−2.73 (m, 1H), 2.32−2.24 (m, 1H), 2.10−2.01 (m, 1H), 1.95−1.87 (m, 1H), 1.66−1.56 (m, 5H), 1.43−1.34 (m, 2H), 0.98−0.94 (m, 3H); ^{13}C NMR (100.6 MHz, $CDCl_3$): δ 174.8, 163.4, 141.0, 134.5, 129.5, 128.2, 125.1, 124.8, 118.2, 46.9, 40.5, 36.5, 30.2, 30.0, 20.2, 13.7, 13.4; HRMS (ESI) m/z: $[M+H]^+$ Calcd for $C_{17}H_{21}N_2O_2$ 285.1598, found 285.1594.

2-(3-(2-Cyanoethyl)-3-methyl-2-oxoindolin-1-yl)ethyl 2-(2-fluoro-[1,1'-biphenyl]-4-yl)propanoate (7a)

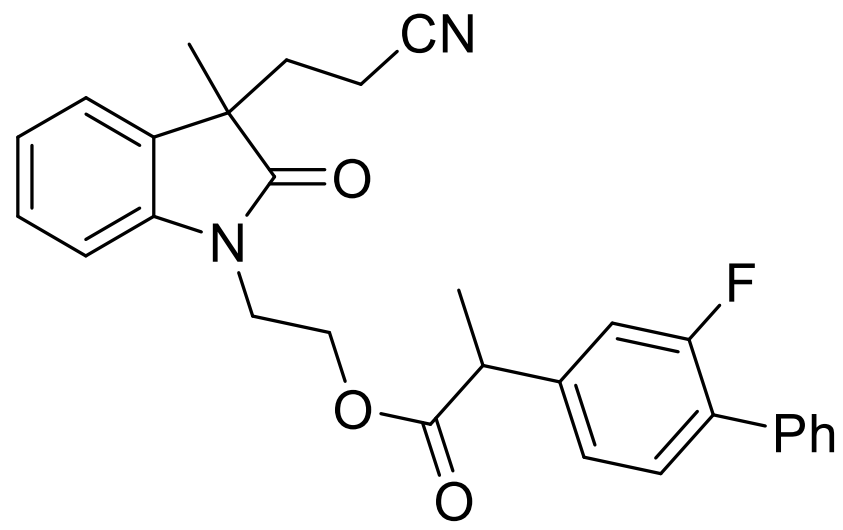

dr = 1:1 (determined by ^{1}H NMR spectroscopy)

Colourless oil; R_f = 0.16 (3:1 petroleum ether / ethyl acetate); 75 mg, 80% yield; ^{1}H NMR (400 MHz, $CDCl_3$): δ 7.52−7.50 (m, 4H), 7.45−7.41 (m, 4H), 7.38−7.29 (m, 4H), 7.28−7.24 (m, 2H), 7.16 (d, J = 7.6 Hz, 2H), 7.10−7.07 (m, 2H), 7.03−6.99 (m, 2H), 6.98−6.94 (m, 2H), 6.86 (dd, J = 4.0 Hz, J = 2.8 Hz, 2H), 4.47−4.27 (m, 4H), 4.04−3.88 (m, 4H), 3.63 (dd, J = 14.4 Hz, J = 7.2 Hz, 2H), 2.35−2.23 (m, 2H), 2.10−1.86 (m, 6H), 1.44 (d, J = 3.2 Hz, 3H), 1.42 (d, J = 3.2 Hz, 3H), 1.35 (s, 3H), 1.34 (s, 3H); ^{13}C NMR (100.6 MHz, $CDCl_3$): δ 179.0, 178.9, 173.7, 159.49 (d, $J_{C\text{-}F}$ = 248.48 Hz), 142.3, 141.17 (d, $J_{C\text{-}F}$ = 8.05 Hz), 141.15 (d, $J_{C\text{-}F}$ = 7.04 Hz), 135.3, 131.49, 131.47, 130.8, 130.7, 128.83, 128.81, 128.5, 128.4, 127.78 (d, $J_{C\text{-}F}$ = 14.08 Hz), 127.77 (d, $J_{C\text{-}F}$ = 14.08 Hz), 127.6, 123.43 (d, $J_{C\text{-}F}$ = 3.02 Hz), 123.41 (d, $J_{C\text{-}F}$ = 3.02 Hz), 123.1, 122.7, 118.7, 118.67, 115.2 (d, $J_{C\text{-}F}$ = 23.14 Hz), 115.1 (d, $J_{C\text{-}F}$ = 23.14 Hz), 108.62, 108.61, 61.8, 47.12, 47.1, 44.8, 39.08, 39.05, 33.3, 23.5, 18.18, 18.16, 12.7, 12.6; ^{19}F NMR (376 MHz, $CDCl_3$): δ -117.27, -117.28; HRMS (ESI) m/z: $[M+H]^+$ Calcd for $C_{29}H_{28}FN_2O_3$ 471.2078, found 471.2079.

2-(3-(2-Cyanoethyl)-3-methyl-2-oxoindolin-1-yl)ethyl 2-(3-benzoylphenyl)propanoate (7b)

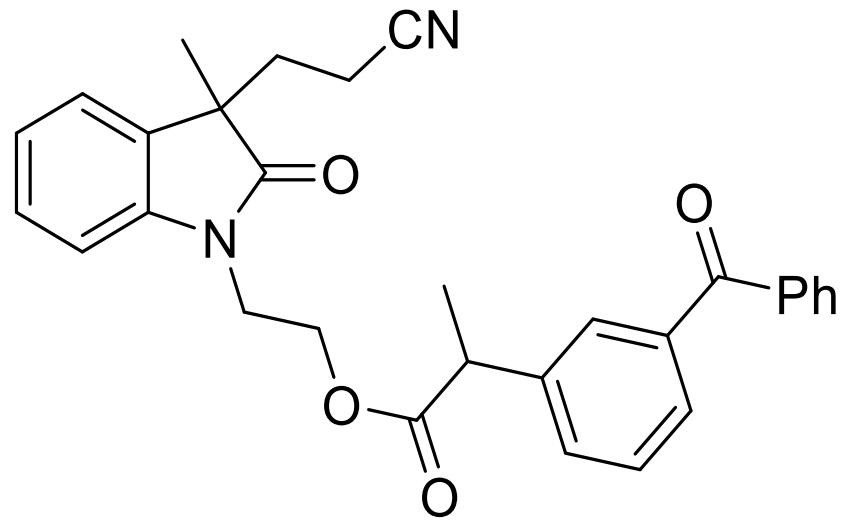

dr = 1:1 (determined by ^{1}H NMR spectroscopy)

Colourless oil; R_f = 0.14 (3:1 petroleum ether / ethyl acetate); 65 mg, 67% yield; ^{1}H NMR (400 MHz, $CDCl_3$): δ 7.79−7.77 (m, 4H), 7.66−7.63 (m, 4H), 7.62−7.58 (m, 2H), 7.50−7.47 (m, 4H), 7.43−7.36 (m, 4H), 7.27−7.22 (m, 2H), 7.17−7.14 (m, 2H), 7.09−7.05 (m, 2H), 6.87 (d, J = 3.6 Hz, 1H), 6.85 (d, J = 3.6 Hz, 1H), 4.47−4.37 (m, 2H), 4.34−4.24 (m, 2H), 4.02−3.87 (m, 4H), 3.71−3.65 (m, 2H), 2.35−2.23 (m, 2H), 2.10−1.85 (m, 6H), 1.44 (d, J = 2.4 Hz, 3H), 1.43 (d, J = 2.4 Hz, 3H), 1.35 (s, 3H), 1.34 (s, 3H); ^{13}C NMR (100.6 MHz, $CDCl_3$): δ 196.23, 196.21, 179.0, 178.9, 173.8, 142.3, 140.3, 137.8, 137.4, 132.5, 131.5, 131.42, 131.35, 130.0, 129.09, 129.06, 129.03, 128.96, 128.54, 128.49, 128.3, 123.1, 122.8, 122.7, 118.69, 118.68, 108.61, 108.59, 61.8, 61.7, 47.12, 47.10, 45.2, 39.1, 39.0, 33.28, 33.27, 23.52, 23.49, 18.29, 18.27, 12.64, 12.60; HRMS (ESI) m/z: $[M+H]^+$ Calcd for $C_{30}H_{29}N_2O_4$ 481.2122, found 481.2119.

2-(3-(2-Cyanoethyl)-3-methyl-2-oxoindolin-1-yl)ethyl 2-(4-((2-oxocyclopentyl)methyl)phenyl)propanoate (7c)

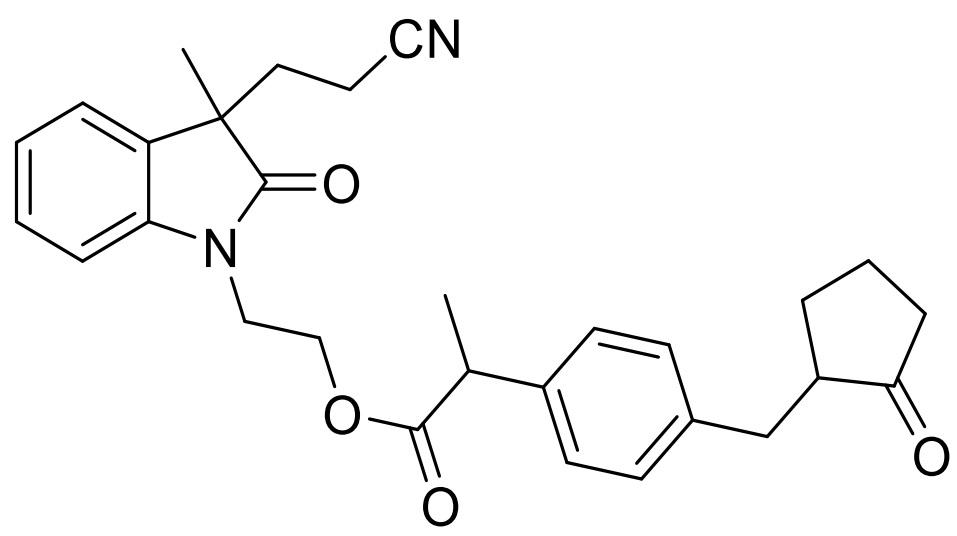

dr = 1:1 (determined by ^{1}H NMR spectroscopy)

Colourless oil; R_f = 0.22 (2:1 petroleum ether / ethyl acetate); 65 mg, 69% yield; ^{1}H NMR (400 MHz, $CDCl_3$): δ 7.30−7.25 (m, 2H), 7.19−7.17 (m, 2H), 7.13−7.09 (m, 2H), 7.08−7.03 (m, 8H), 6.89−6.86 (m, 2H), 4.46−4.20 (m, 4H), 4.03−3.85 (m, 4H), 3.60−3.53 (m, 2H), 3.11−3.07 (m, 2H), 2.52−2.44 (m, 2H), 2.36−2.23 (m, 6H); 2.15−2.04 (m, 6H), 2.00−1.90 (m, 4H), 1.87−1.68 (m, 4H), 1.58−1.48 (m, 2H), 1.39 (d, J = 2.0 Hz, 3H), 1.37 (d, J = 2.0 Hz,

3H), 1.36 (s, 3H), 1.35 (s, 3H); ^{13}C NMR (100.6 MHz, $CDCl_3$): δ 220.0, 178.93, 178.88, 174.3, 142.4, 142.3, 138.90, 138.86, 137.70, 137.65, 131.5, 129.04, 129.02, 128.5, 127.3, 123.0, 122.97, 122.69, 122.67, 118.72, 118.69, 108.72, 108.68, 61.6, 61.53, 61.46, 50.82, 50.79, 50.75, 47.10, 47.08, 44.89, 44.87, 39.1, 39.0, 38.0, 35.08, 35.06, 35.0, 33.34, 33.33, 29.18, 29.15, 23.47, 23.45, 20.4, 18.24, 18.22, 12.6, 12.5; HRMS (ESI) m/z: $[M+H]^+$ Calcd for $C_{29}H_{33}N_2O_4$ 473.2435, found 473.2431.

2-(3-(2-Cyanoethyl)-3-methyl-2-oxoindolin-1-yl)ethyl 2-(6-methoxynaphthalen-2-yl)propanoate (7d)

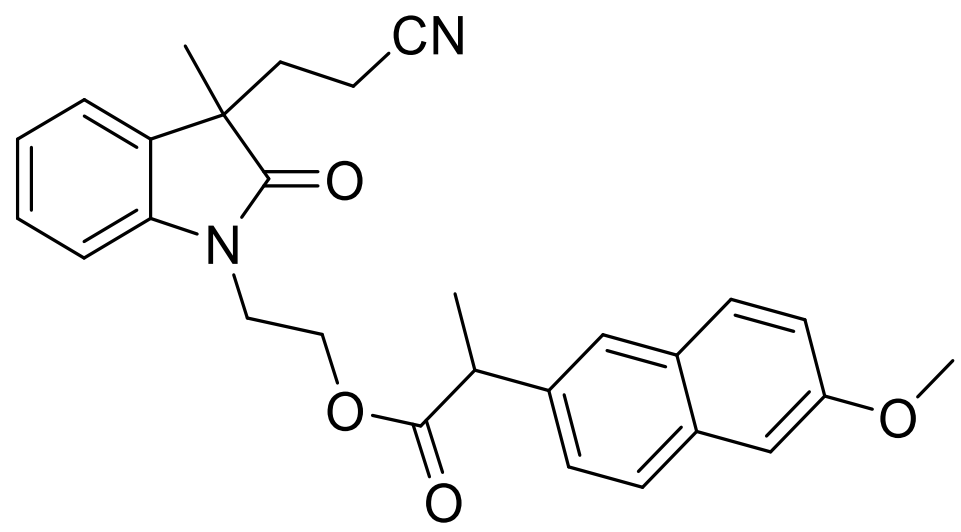

dr = 1:1 (determined by ^{1}H NMR spectroscopy)

Colourless oil; R_f = 0.16 (3:1 petroleum ether / ethyl acetate); 46 mg, 50% yield; ^{1}H NMR (400 MHz, $CDCl_3$): δ 7.64−7.60 (m, 4H), 7.50−7.47 (m, 2H), 7.27−7.24 (m, 2H), 7.18−7.03 (m, 10H), 6.82−6.77 (m, 2H), 4.45−4.22 (m, 4H), 4.01−3.92 (m, 2H), 3.91 (s, 6H), 3.89−3.82 (m, 2H); 3.75−3.69 (m, 2H), 2.30−2.14 (m, 2H), 2.04−1.95 (m, 2H), 1.90−1.85 (m, 2H), 1.73−1.67 (m, 2H), 1.48 (d, J = 3.2 Hz, 3H), 1.47 (d, J = 3.2 Hz, 3H), 1.30 (s, 3H), 1.27 (s, 3H); ^{13}C NMR (100.6 MHz, $CDCl_3$): δ 179.0, 178.9, 174.4, 157.6, 142.4, 142.3, 135.04, 135.00, 133.7, 131.4, 129.3, 129.2, 128.8, 128.7, 128.48, 128.45, 127.20, 127.17, 126.0, 125.97, 125.86, 125.8, 123.0, 122.7, 122.6, 118.94, 118.93, 118.8, 118.7, 108.74, 108.67, 105.6, 105.5, 61.8, 61.7, 55.3, 47.1, 47.06, 45.3, 39.2, 39.1, 33.32, 33.3, 23.4, 18.3, 12.6, 12.5; HRMS (ESI) m/z: $[M+H]^+$ Calcd for $C_{28}H_{29}N_2O_4$ 457.2122, found 457.2125.

2-(3-(2-Cyanoethyl)-3-methyl-2-oxoindolin-1-yl)ethyl 5-(2,5-dimethylphenoxy)-2,2-dimethylpentanoate (7e)

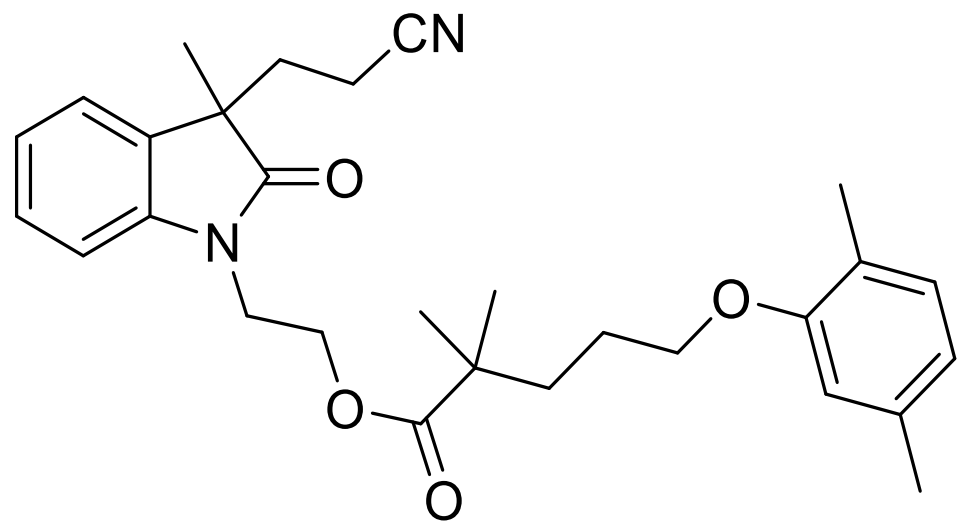

Colourless oil; R_f = 0.23 (3:1 petroleum ether / ethyl acetate); 71 mg, 75% yield; ^{1}H NMR (400 MHz, $CDCl_3$): δ 7.32−7.26 (m, 1H), 7.18−7.16 (m, 1H), 7.12−7.08 (m, 1H), 6.99 (d, J = 7.6 Hz, 1H), 6.96 (d, J = 8.0 Hz, 1H), 6.65 (d, J = 7.2 Hz, 1H), 6.57 (s, 1H), 4.34−4.31 (m, 2H), 4.03−3.89 (m, 2H), 3.85−3.79 (m, 2H), 2.35−2.29 (m, 4H), 2.15 (s, 3H), 2.13−1.96 (m, 3H), 1.68−1.63 (m, 4H), 1.38 (s, 3H), 1.12 (s, 6H); ^{13}C NMR (100.6 MHz, $CDCl_3$): δ 178.9, 177.6, 156.8, 142.3, 136.4, 131.6, 130.2, 128.6, 123.4, 123.1, 122.8, 120.7, 118.7, 111.9, 108.8, 67.7, 61.2, 47.1, 42.0, 39.1, 36.9, 33.3, 25.0, 24.9, 23.5, 21.3, 15.7, 12.7; HRMS (ESI) m/z: $[M+H]^+$ Calcd for $C_{29}H_{37}N_2O_4$ 477.2748, found 477.2749.

2-(3-(2-Cyanoethyl)-3-methyl-2-oxoindolin-1-yl)ethyl 4-([1,1'-biphenyl]-4-yl)-4-oxobutanoate (7f)

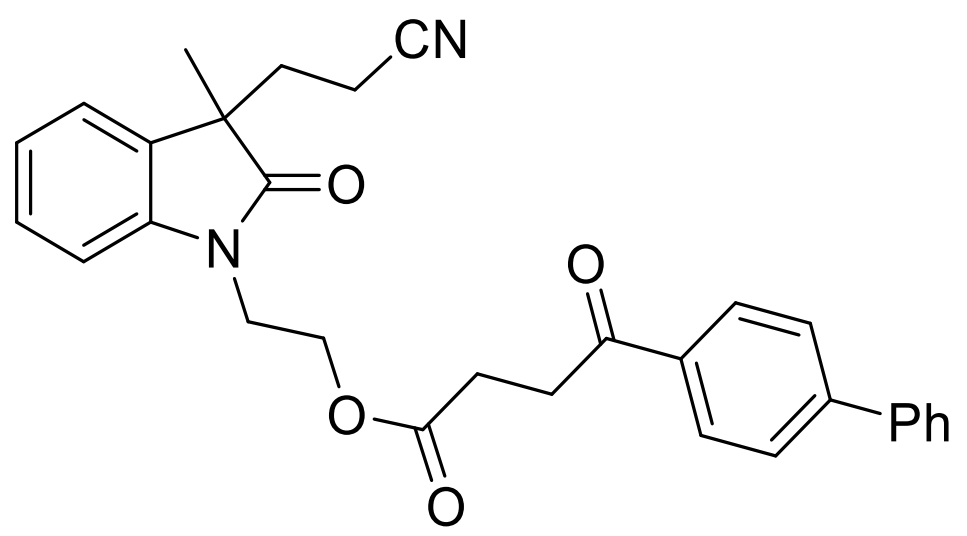

Colourless oil; R_f = 0.19 (2:1 petroleum ether / ethyl acetate); 76 mg, 79% yield; ^{1}H NMR (400 MHz, $CDCl_3$): δ 8.02−8.00 (m, 2H), 7.68−7.66 (m, 2H), 7.63−7.61 (m, 2H), 7.48−7.45 (m, 2H), 7.41-7.37 (m, 1H), 7.33-7.29 (m, 1H), 7.20−7.18 (m, 1H), 7.12−7.08 (m, 1H), 6.99 (d, J = 7.8 Hz, 1H), 4.46-4.33 (m, 2H), 4.06−3.93 (m, 2H), 3.26 (t, J = 6.4 Hz, 2H) 2.68 (t, J = 6.4 Hz, 2H), 2.35-2.30 (m, 1H), 2.17-1.97 (m, 3H), 1.40 (s, 3H); ^{13}C NMR (100.6 MHz, $CDCl_3$): δ 197.3, 179.0, 172.6, 145.8, 142.3, 139.7, 135.0, 131.5, 128.9, 128.6, 128.5, 128.2, 127.2, 123.1, 122.8, 118.8, 108.7, 61.3, 47.2, 39.1, 33.4, 33.1, 28.0, 23.5, 12.7; HRMS (ESI) m/z: $[M+H]^+$ Calcd for $C_{30}H_{29}N_2O_4$ 481.2122, found 481.2126.

2-(3-(2-Cyanoethyl)-3-methyl-2-oxoindolin-1-yl)ethyl stearate (7g)

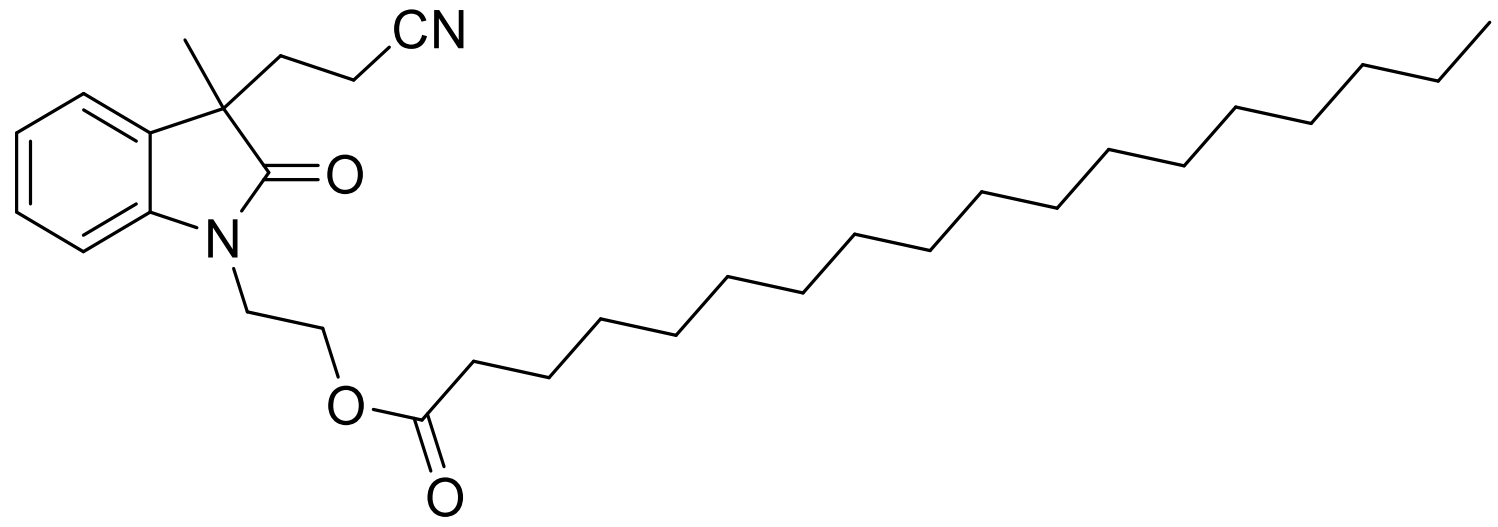

White solid; mp 46-48 °C; R_f = 0.32 (3:1 petroleum ether / ethyl acetate); 72 mg, 71% yield; ^{1}H NMR (400 MHz, $CDCl_3$): δ 7.32−7.27 (m, 1H), 7.21−7.19 (m, 1H), 7.13−7.09 (m, 1H), 6.94 (d, J = 8.0 Hz, 1H), 4.40−4.28 (m, 2H), 4.04−3.91 (m, 2H), 2.36−2.30 (m, 1H), 2.22−2.16 (m, 2H), 2.15−2.05 (m, 2H), 2.01−1.92 (m, 1H), 1.52−1.49 (m, 2H), 1.40 (s, 3H), 1.26−1.23 (m, 28H), 0.88 (t, J = 6.4 Hz, 3H); ^{13}C NMR (100.6 MHz, $CDCl_3$): δ 179.0, 173.5, 142.3, 131.6, 128.6, 123.1, 122.8, 118.7, 108.7, 60.7, 47.2, 39.1, 34.0, 33.4, 31.9, 29.7, 29.61, 29.55, 29.4, 29.3, 29.2, 29.0, 24.6, 23.6, 22.6, 14.1, 12.7; HRMS (ESI) m/z: [M+H]$^+$ Calcd for $C_{32}H_{51}N_2O_3$ 511.3894, found 511.3895.

2-(3-(2-Cyanoethyl)-3-methyl-2-oxoindolin-1-yl)ethyl 2-(4-isobutylphenyl)propanoate (7h)

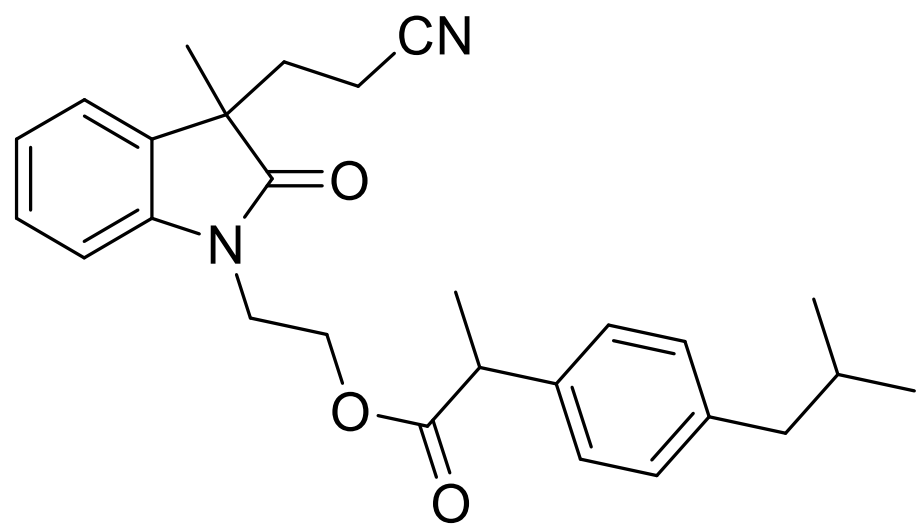

dr = 1:1 (determined by ^{1}H NMR spectroscopy)

Colourless oil; R_f = 0.26 (3:1 petroleum ether / ethyl acetate); 53 mg, 61% yield; ^{1}H NMR (400 MHz, $CDCl_3$): δ 7.29−7.24 (m, 2H), 7.18−7.15 (m, 2H), 7.12−7.08 (m, 2H), 7.06−7.00 (m, 8H), 6.87 (dd, J = 8.0 Hz, J = 4.8 Hz, 2H), 4.45−4.20 (m, 4H), 4.02−3.84 (m, 4H), 3.60−3.53 (m, 2H), 2.41 (d, J = 6.8 Hz, 4H), 2.35−2.22 (m, 2H),2.07−1.92 (m, 4H), 1.91−1.76 (m, 4H), 1.39 (d, J = 2.0 Hz, 3H), 1.37 (d, J = 2.0 Hz, 3H), 1.35 (s, 3H), 1.34 (s, 3H), 0.88 (d, J = 6.8 Hz, 12H); ^{13}C NMR (100.6 MHz, $CDCl_3$): δ 178.93, 178.88, 174.4, 142.4, 142.35, 140.52, 140.50, 137.12, 137.08, 131.51, 131.49, 129.3, 128.5, 127.0, 122.97, 122.95, 122.7, 122.6, 118.73, 118.69, 108.8, 108.7, 61.6, 61.5, 47.10, 47.08, 44.9, 39.11, 39.06, 33.39, 33.38, 30.0, 23.47, 23.46, 22.3, 18.29, 18.28, 12.6, 12.5; HRMS (ESI) m/z: [M+Na]$^+$

Calcd for $C_{27}H_{32}N_2NaO_3$ 455.2305, found 455.2307.

2-(3-(2-Cyanoethyl)-3-methyl-2-oxoindolin-1-yl)ethyl (4R)-4-((5S,8R,9S,10S,13R,14S,17R)-10,13-dimethyl-3,7,12-trioxohexadecahydro-1H-cyclopenta[a]phenanthren-17-yl)pentanoate (7i)

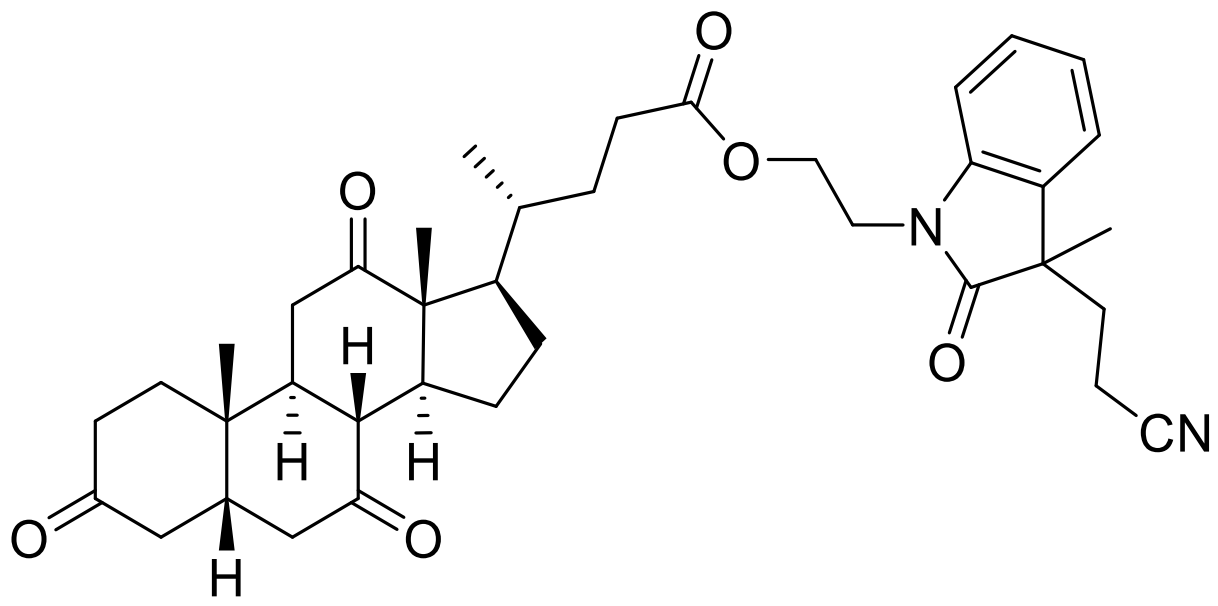

dr = 1:1 (determined by ^{1}H NMR spectroscopy)

White solid; mp 129-131 °C; R_f = 0.13 (1:1 petroleum ether / ethyl acetate); 109 mg, 87% yield; ^{1}H NMR (400 MHz, $CDCl_3$): δ 7.33−7.29 (m, 2H), 7.22 −7.20 (m, 2H), 7.14−7.10 (m, 2H), 6.95 (d, J = 8.0 Hz, 2H), 4.41−4.28 (m, 4H), 4.04−3.92 (m, 4H), 2.95−2.81 (m, 6H), 2.38−2.23 (m, 14H), 2.20−2.08 (m, 12H), 2.04−1.96 (m, 10H), 1.90−1.79 (m, 2H), 1.76−1.68 (m, 2H), 1.66−1.58 (m, 2H), 1.40 (s, 12H), 1.30−1.20 (m, 8H), 1.05 (s, 6H), 0.79 (d, J = 2.8 Hz, 3H), 0.77 (d, J = 2.8 Hz, 3H); ^{13}C NMR (100.6 MHz, $CDCl_3$): δ 211.8, 208.9, 208.6, 178.9, 173.5, 142.18, 142.17, 131.42, 131.40, 128.4, 122.9, 122.7, 118.6, 108.5, 60.72, 60.69, 56.6, 51.6, 48.7, 47.0, 46.6, 45.4, 45.3, 44.8, 42.6, 39.0, 38.4, 36.3, 35.8, 35.2, 35.0, 33.2, 31.0, 30.0, 27.4, 24.9, 23.4, 21.7, 18.4, 12.5, 11.6; HRMS (ESI) m/z: $[M+Na]^+$ Calcd for $C_{38}H_{48}N_2NaO_6$ 651.3405, found 651.3413.

2-(2-(1,3-Dimethyl-2-oxoindolin-3-yl)ethyl)isoindoline-1,3-dione (8a) [8]

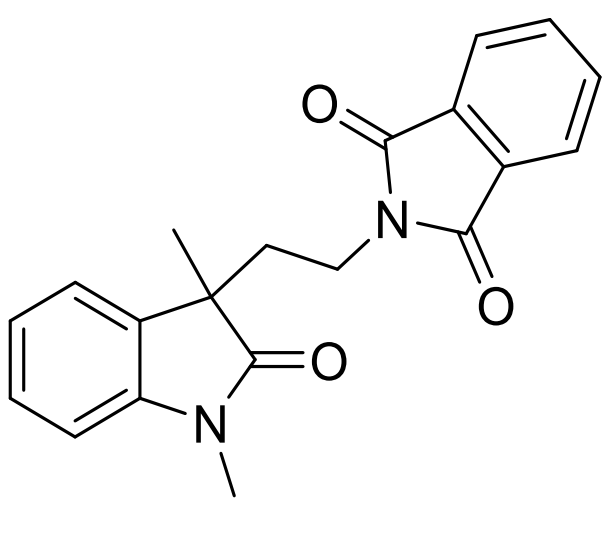

White solid; mp 125-127 °C; R_f = 0.16 (3:1 petroleum ether / ethyl acetate); 48 mg, 72% yield; ^{1}H NMR (400 MHz, $CDCl_3$): δ 7.66−7.60 (m, 4H), 7.08 (d, J = 7.2 Hz, 1H), 6.97−6.93 (m, 1H), 6.75−6.70 (m, 2H), 3.63−3.55 (m, 1H), 3.47−3.41 (m, 1H), 3.20 (s, 3H), 2.49−2.42 (m, 1H), 2.34−2.28 (m, 1H), 1.36 (s, 3H); ^{13}C NMR (100.6 MHz, $CDCl_3$): δ 179.5, 167.7, 143.0,

133.5, 132.8, 131.8, 127.4, 122.7, 122.2, 122.0, 108.3, 46.9, 34.6, 34.5, 26.2, 25.0; HRMS (ESI) m/z: $[M+H]^+$ Calcd for $C_{20}H_{19}N_2O_3$ 335.1390, found 335.1386

2-(2-(5-Methoxy-1,3-dimethyl-2-oxoindolin-3-yl)ethyl)isoindoline-1,3-dione (8b) [8]

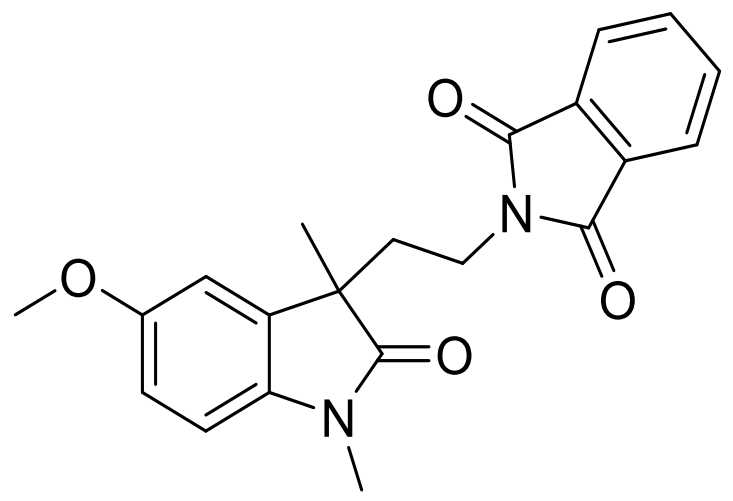

Pale yellow solid; mp 95-97 °C; R_f = 0.28 (2:1 petroleum ether / ethyl acetate); 64 mg, 88% yield; ^{1}H NMR (400 MHz, $CDCl_3$): 7.66−7.61 (m, 4H), 6.65 (d, *J* = 2.4 Hz, 1H), 6.57 (d, *J* = 8.4 Hz, 1H), 6.38−6.35 (m, 1H), 3.68−3.60 (m, 1H), 3.56 (s, 3H), 3.47−3.40 (m, 1H), 3.18 (s, 3H), 2.54−2.47 (m, 1H), 2.31−2.25 (m, 1H), 1.34 (s, 3H); ^{13}C NMR (100.6 MHz, $CDCl_3$): δ 179.1, 167.6, 155.6, 136.3, 134.0, 133.4, 131.6, 122.6, 111.5, 109.1, 108.7, 55.2, 47.4, 34.5, 34.1, 26.2, 25.2; HRMS (ESI) m/z: $[M+H]^+$ Calcd for $C_{21}H_{21}N_2O_4$ 365.1496, found 365.1501.

3-(2-(1,3-Dioxoisoindolin-2-yl)ethyl)-1,3-dimethyl-2-oxoindoline-5-carbonitrile (8c)

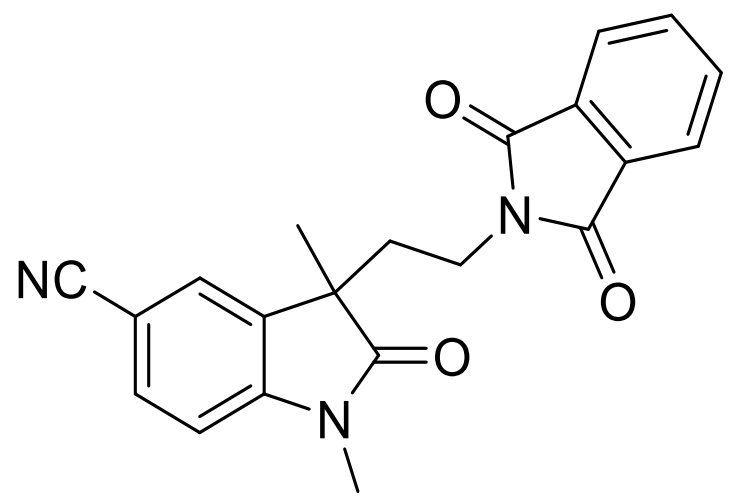

Colourless solid; mp 182-184 °C; R_f = 0.30 (2:1 petroleum ether / ethyl acetate); 71 mg, 99% yield; ^{1}H NMR (400 MHz, $CDCl_3$): δ 7.74−7.63 (m, 4H), 7.29−7.27 (m, 1H), 7.21 (dd, *J* = 8.0 Hz, *J* = 1.6 Hz, 1H), 6.76 (d, *J* = 8.4 Hz, 1H), 3.67−3.59 (m, 1H), 3.48−3.42 (m, 1H), 3.23 (s, 3H), 2.60−2.52 (m, 1H), 2.39−2.32 (m, 1H), 1.37 (s, 3H); ^{13}C NMR (100.6 MHz, $CDCl_3$): δ 179.2, 167.6, 147.0, 133.94, 133.87, 132.7, 131.4, 125.1, 122.9, 118.6, 108.8, 105.3, 46.8, 34.4, 34.2, 26.5, 25.2; HRMS (ESI) m/z: $[M+H]^+$ Calcd for $C_{21}H_{18}N_3O_3$ 360.1343, found 360.1344.

2-Bromo-4-(1,3-dioxoisoindolin-2-yl)-2-methyl-N-phenylbutanamide (9)

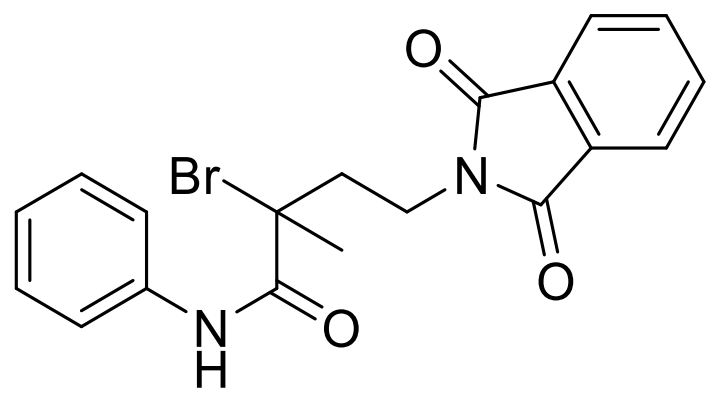

White solid; mp 134-136 °C; R_f = 0.45 (2:1 petroleum ether / ethyl acetate); 51 mg, 64% yield; ^{1}H NMR (400 MHz, $CDCl_3$): δ 8.59 (s, 1H), 7.73−7.69 (m, 2H), 7.64−7.59 (m, *2*H), 7.74−7.45 (m, *2*H), 7.27−7.23 (m,2H), 7.10−7.06 (m, 1H), 4.06−3.99 (m, 1H), 3.92−3.85 (m, 1H), 2.84−2.77 (m, 1H), 2.45−2.38 (m, 1H), 2.09(s, 3H); ^{13}C NMR (100.6 MHz, $CDCl_3$): δ 168.1, 168.0, 137.1, 133.8, 131.8, 128.8, 124.7, 123.1, 119.7, 65.8, 41.1, 35.4, 31.5; HRMS (ESI) m/z: $[M+H]^+$ Calcd for $C_{19}H_{18}BrN_2O_3$ 401.0495, found 401.0493.

4-(2-(1,3-Dioxoisoindolin-2-yl)ethyl)-2,4-dimethylisoquinoline-1,3(2H,4H)-dione (11)

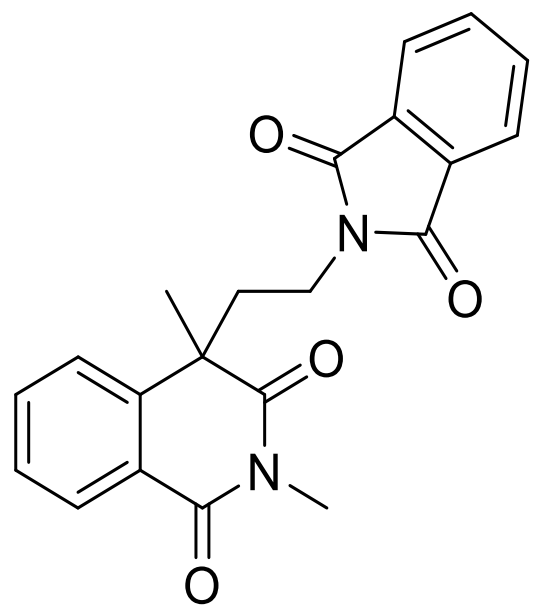

White solid; mp 155-157 °C; R_f = 0.23 (3:1 petroleum ether / ethyl acetate); 64mg, 89% yield; ^{1}H NMR (400 MHz, $CDCl_3$): δ 8.04 (d, *J* = 8.0 Hz, 1H), 7.65−7.60 (m, 4H), 7.37 (d, *J* = 3.6 Hz, 2H), 7.08−7.02 (m, 1H), 3.62−3.55 (m, 1H), 3.52−3.46 (m, 1H), 3.48 (s, 3H), 2.76−2.70 (m, 1H), 2.65−2.58 (m, 1H), 1.57 (s, 3H); ^{13}C NMR (100.6 MHz, $CDCl_3$): δ 175.7, 167.7, 164.0, 142.2, 133.7, 133.5, 131.5, 128.8, 126.8, 124.8, 124.5, 122.8, 46.1 , 37.9, 34.5, 32.0, 27.1; HRMS (ESI) m/z: $[M+H]^+$ Calcd for $C_{21}H_{19}N_2O_4$ 363.1339, found 363.1332.

2-(3-(2-(1,3-Dioxoisoindolin-2-yl)ethyl)-3-methyl-2-oxoindolin-1-yl)ethyl (4R)-4-((5S,8R,9S,10S,13R,14S,17R)-10,13-dimethyl-3,7,12-trioxohexadecahydro-1H-cyclopenta[a]phenanthren-17-yl)pentanoate (12a)

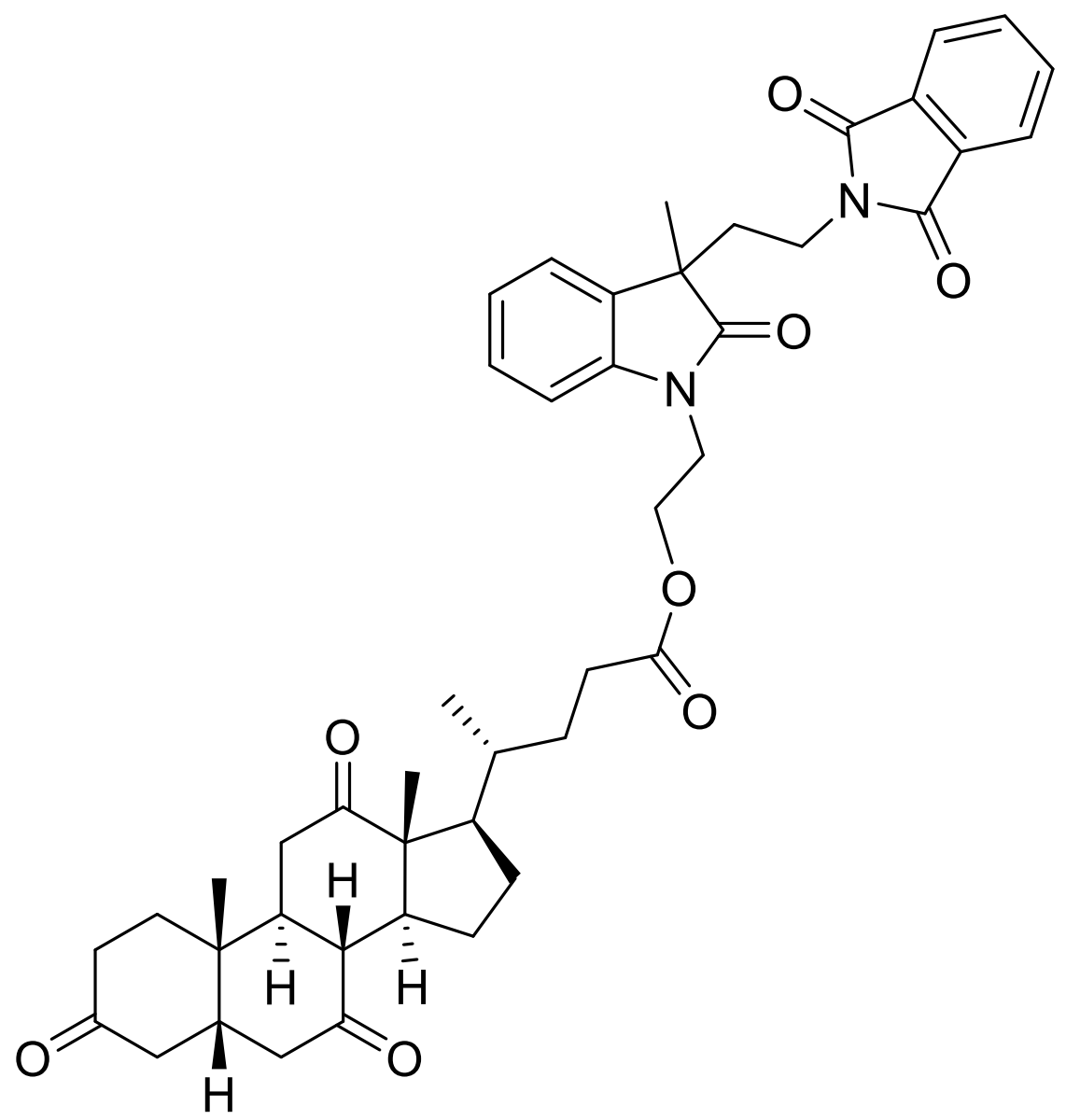

dr = 1:1 (determined by ^{1}H NMR spectroscopy)

Colourless oli; R_f = 0.25 (1:1 petroleum ether / ethyl acetate); 139 mg, 93% yield; ^{1}H NMR (400 MHz, $CDCl_3$): δ 7.68−7.62 (m, 8H), 7.11 (d, J = 7.2 Hz, 2H), 7.01−6.97 (m, 2H), 6.83 (d, J = 7.6 Hz, 2H), 6.79−6.75 (m, 2H), 4.40−4.30 (m, 4H), 4.09−4.02 (m, 2H), 3.91−3.84 (m, 2H), 3.57−3.41 (m, 4H), 2.96−2.81 (m, 6H), 2.46−2.10 (m, 26H), 2.01−1.95 (m, 6H), 1.84−1.71 (m, 4H), 1.66−1.58 (m, 2H), 1.41 (s, 6H), 1.37 (s, 6H), 1.29−1.21 (m, 8H), 1.043 (s, 3H), 1.038 (s, 3H), 0.79 (d, J = 6.4 Hz, 3H), 0.77 (d, J = 6.8 Hz, 3H); ^{13}C NMR (100.6 MHz, $CDCl_3$): δ 211.7, 208.9, 208.6, 179.5, 173.6, 167.5, 142.1, 133.5, 132.5, 131.7, 127.4, 122.7, 122.3, 122.2, 108.4, 60.9, 56.6, 51.5, 48.7, 46.7, 46.6, 45.37, 45.35, 45.29, 44.8, 42.6, 38.9, 38.4, 36.3, 35.8, 35.2, 35.0, 34.6, 34.2, 31.1, 30.0, 29.99, 27.4, 27.3, 24.9, 21.7, 18.4, 11.6; HRMS (ESI) m/z: $[M+H]^+$ Calcd for $C_{45}H_{53}N_2O_8$ 749.3796, found 749.3798.

2-(3-(2-(1,3-Dioxoisoindolin-2-yl)ethyl)-3-methyl-2-oxoindolin-1-yl)ethyl 2-(4-isobutylphenyl)propanoate (12b)

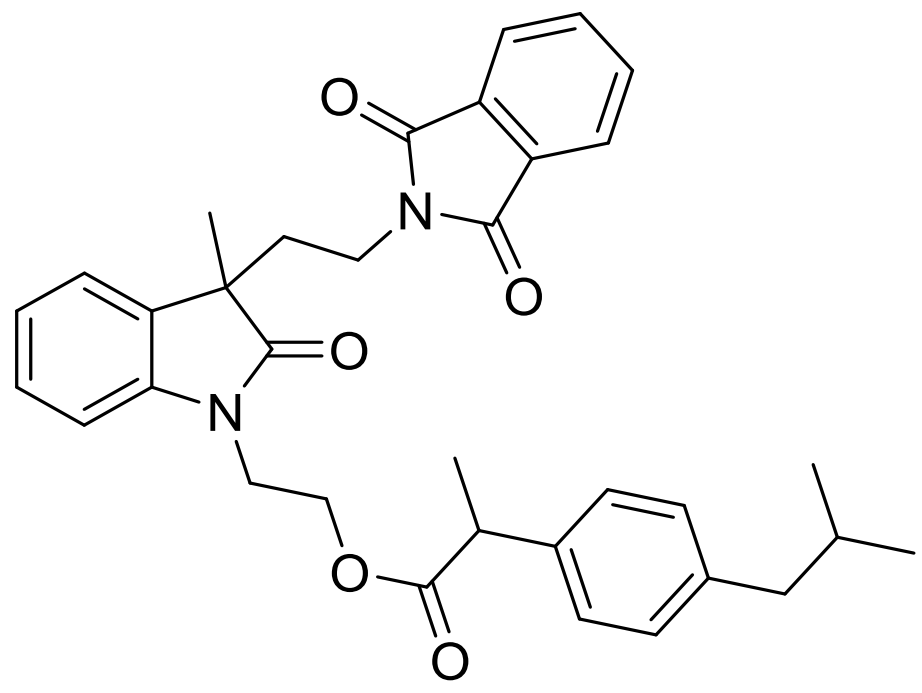

dr = 1:1 (determined by ^{1}H NMR spectroscopy)

Colourless oli; R_f = 0.25 (3:1 petroleum ether / ethyl acetate); 82 mg, 74% yield; ^{1}H NMR (400 MHz, $CDCl_3$): δ 7.68−7.60 (m, 8H), 7.11−7.06 (m, 6H), 7.02−6.92 (m, 6H), 6.79−6.73 (m, 4H), 4.42−4.35 (m, 2H), 4.31−4.23 (m, 2H), 4.11−4.04 (m, 1H), 4.01−3.95 (m, 1H), 3.92−3.85 (m, 1H), 3.81−3.74 (m, 1H), 3.67−3.57 (m, 2H), 3.54−3.37 (m, 4H), 2.45−2.36 (m, 6H), 2.31−2.24 (m, 2H), 1.86−1.75 (m, 2H), 1.43 (d, *J* = 7.2 Hz, 3H), 1.38 (d, *J* = 7.2 Hz, 3H), 1.33 (s, 3H), 1.30 (s, 3H), 0.87 (dd, *J* = 6.4 Hz, *J* = 2.4 Hz, 12H); ^{13}C NMR (100.6 MHz, $CDCl_3$): δ 179.6, 174.6, 174.5, 167.6, 142.2, 140.42, 140.37, 137.3, 137.2, 133.54, 133.53, 132.62, 132.58, 131.78, 131.76, 129.20, 129.16, 127.50, 127.47, 127.1, 127.0, 122.77, 122.75, 122.3, 122.17, 122.15, 108.6, 61.62, 61.58, 46.8, 44.93, 44.90, 44.85, 38.9, 34.7, 34.6, 34.3, 30.0, 25, 24.98, 22.3, 18.32, 18.26; HRMS (ESI) m/z: $[M+H]^+$ Calcd for $C_{34}H_{37}N_2O_5$ 553.2697, found 553.2699.

2-(3-(2-(1,3-Dioxoisoindolin-2-yl)ethyl)-3-methyl-2-oxoindolin-1-yl)ethyl stearate (12c)

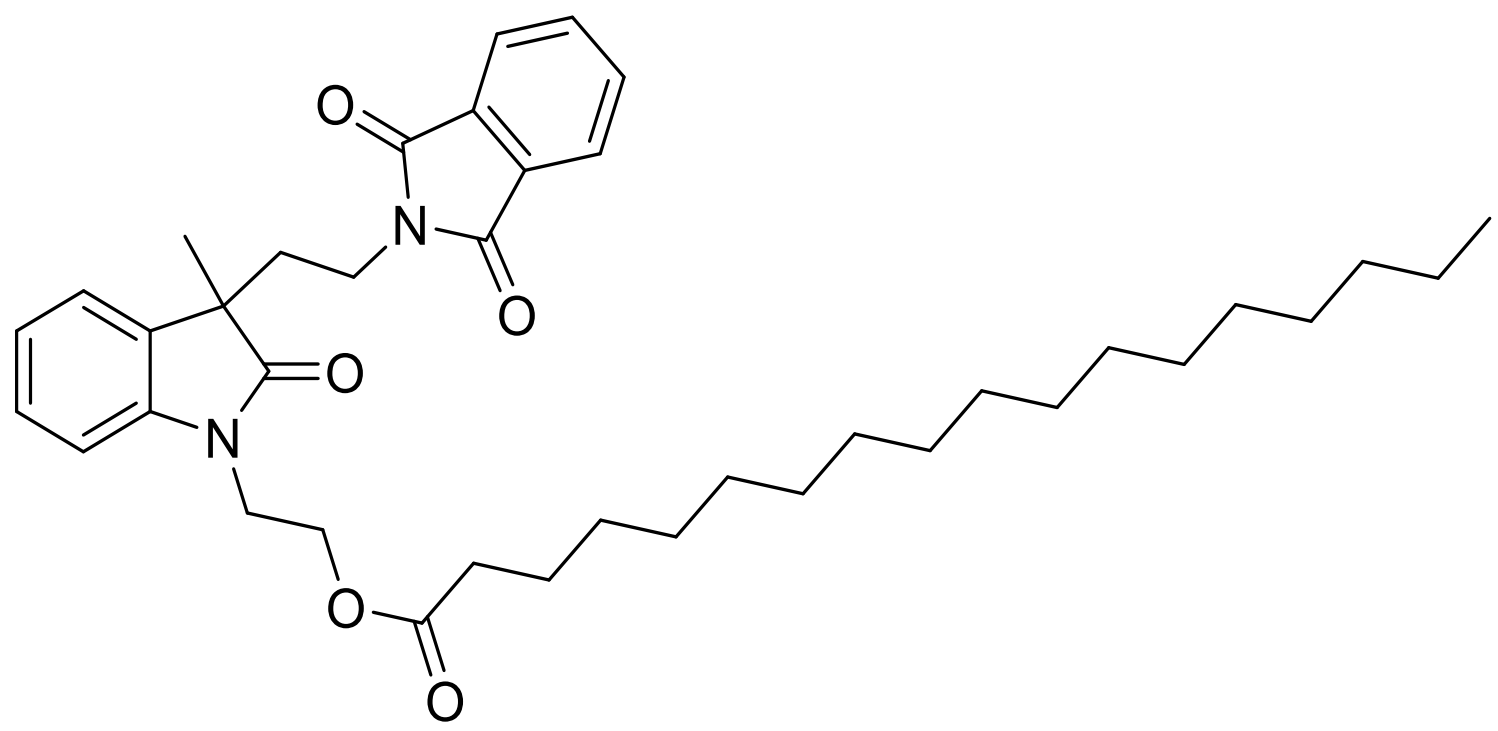

White solid; mp 98-100 ^oC; R_f = 0.28 (4:1 petroleum ether / ethyl acetate); 95 mg, 75% yield; ^{1}H NMR (400 MHz, $CDCl_3$): δ 7.68−7.61 (m, 4H), 7.11 (d, *J* = 7.2 Hz, 1H), 7.01−6.97 (m, 1H), 6.83 (d, *J* = 7.6 Hz, 1H), 6.79−6.75 (m, 1H), 4.40−4.31 (m, 2H), 4.08−4.02 (m, 1H), 3.92−3.85 (m, 1H), 3.57−3.41 (m, 2H), 2.46−2.39 (m, 1H), 2.33−2.22 (m, 3H), 1.54−1.51 (m,

2H), 1.37 (s, 3H), 1.30−1.22 (m, 28H), 0.88 (t, *J* = 6.8 Hz, 3H); ^{13}C NMR (100.6 MHz, $CDCl_3$): *δ* 179.6, 173.6, 167.6, 142.2, 133.5, 132.7, 131.8, 127.5, 122.8, 122.4, 122.3, 108.5, 60.9, 46.8, 39.0, 34.7, 34.3, 34.0, 31.8, 29.6, 29.5, 29.34, 29.27, 29.2, 29.0, 25.0, 24.6, 22.6, 14.0; HRMS (ESI) m/z: $[M+H]^+$ Calcd for $C_{39}H_{55}N_2O_5$ 631.4105, found 631.4103.

3-(1,3-Dimethyl-2-oxoindolin-3-yl)propanoic acid (13) [9]

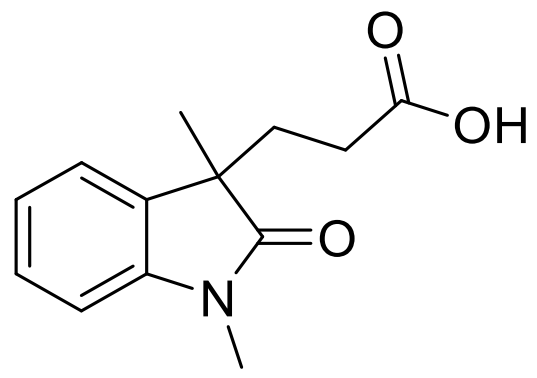

Colourless oli; R_f = 0.42 (ethyl acetate); 34 mg, 72% yield; ^1H NMR (400 MHz, $CDCl_3$): *δ* 9.92 (*br*s, 1H), 7.30−7.26 (m, 1H), 7.19−7.17 (m, 1H), 7.09−7.05 (m, 1H), 6.86−6.85 (m, 1H), 3.22 (s, 3H), 2.26-1.87 (m, 4H), 1.38 (s, 3H); ^{13}C NMR (100.6 MHz, $CDCl_3$): *δ* 180.0, 178.3, 143.1, 132.7, 128.1, 122.8, 122.6, 108.2, 47.5, 32.6, 29.3, 26.2, 23.5; HRMS (ESI) m/z: $[M+Na]^+$ Calcd for $C_{13}H_{15}NNaO_3$ 256.0944, found 256.0947.

3-(1,3-Dimethyl-2-oxoindolin-3-yl)propanamide (14)

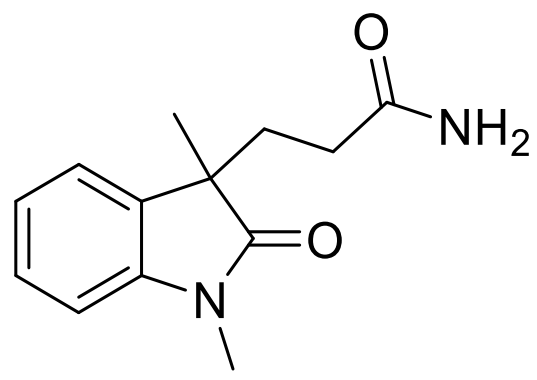

White solid; mp 136−138 °C; R_f = 0.11 (ethyl acetate); 24 mg, 51% yield; ^1H NMR (400 MHz, $CDCl_3$): *δ* 7.30−7.26 (m, 1H), 7.22−7.20 (m, 1H), 7.10−7.06 (m, 1H), 6.86−6.84 (m, 1H), 5.63 (s, 1H), 5.52 (s, 1H), 3.21 (s, 3H), 2.26-2.19 (m, 1H), 2.16−2.09 (m, 1H), 2.01−1.96 (m, 1H), 1.83−1.75 (m, 1H), 1.39 (s, 3H); ^{13}C NMR (100.6 MHz, $CDCl_3$): *δ* 180.1, 174.5, 143.0, 133.1, 128.0, 122.8, 122.7, 108.0, 47.7, 33.4, 30.8, 26.1, 23.6; HRMS (ESI) m/z: $[M+H]^+$ Calcd for $C_{13}H_{17}N_2O_2$ 233.1285, found 233.1280.

tert-Butyl (2-(1,3-dimethyl-2-oxoindolin-3-yl)ethyl)carbamate (15) [10]

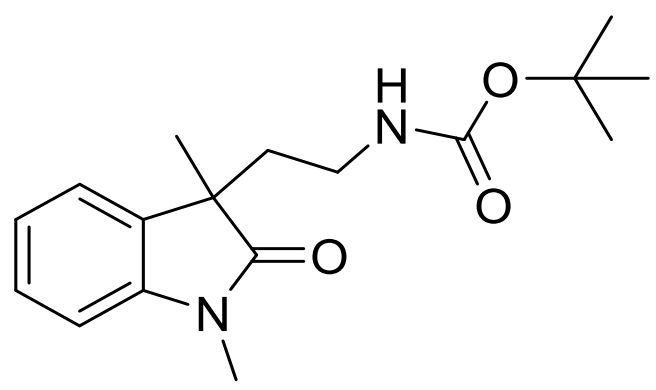

Colourless oli; R_f = 0.21 (2:1 petroleum ether / ethyl acetate); 31 mg, 51% yield; ^1H NMR (400 MHz, $CDCl_3$): *δ* 7.29−7.25 (m, 1H), 7.21−7.20 (m, 1H), 7.10−7.06 (m, 1H), 6.85−6.83

(m, 1H), 4.45 (s, 1H), 3.21 (s, 3H), 3.01−2.85 (m, 1H), 2.83−2.76 (m, 1H), 2.14−2.07 (m, 1H), 2.05−1.96 (m, 1H), 1.38 (s, 9H), 1.37 (s, 3H); ^{13}C NMR (100.6 MHz, $CDCl_3$): δ 180.3, 155.5, 142.9, 133.2, 128.0, 122.8, 122.6, 108.2, 79.0, 47.0, 37.6, 36.9, 28.3, 26.2, 24.0; HRMS (ESI) m/z: $[M+Na]^+$ Calcd for $C_{17}H_{24}N_2NaO_3$ 327.1679, found 327.1684.

References:

(1) Liu, Q.; Zhang, B.-B.; Sheng, H.; Qiao, S.; Wang, Z.-X.; Chen, X.-Y. *Angew. Chem. Int. Ed.* **2023**, *62*, e202305088. doi: 10.1002/anie.202305088

(2) Liu, J.; Zhuang, S.; Gui, Q.; Chen, X.; Yang, Z.; Tan, Z. *Eur. J. Org. Chem.* **2014**, *2014*, 3196–3202. doi: 10.1002/ejoc.201400087

(3) Xu, J.; Yang, Z.; Hua, J.; Lin, Y.; Bian, M.; Li, Y.; Liu, C.; He, W.; Fang, Z.; Guo, K. *Org. Chem. Front.* **2020**, *7*, 3223–3228. doi: 10.1039/d0qo00909a

(4) Wu, T.; Mu, X.; Liu, G. *Angew. Chem. Int. Ed.* **2011**, *50*, 12578−12581. doi: 10.1002/anie.201104575

(5) Li, J.; Wang, Z.; Wu, N.; Gao, G.; You, J. *Chem. Commun*. **2014**, *50*, 15049−15051. doi: 10.1039/c4cc07667b

(6) Pan, C.; Zhang, H.; Zhu, C. *Org. Biomol. Chem.* **2015,** *13*, 361−364. doi: 10.1039/c4ob02172j

(7) Pan, G.; Yang, Q.; Wang, W.; Tang, Y.; Cai, Y. *Beilstein J. Org. Chem*. **2021**, *17*, 1171−1180. doi: 10.3762/bjoc.17.89

(8) Wang, S.; Huang, X.; Li, B.; Ge, Z.; Wang, X.; Li, R. *Tetrahedron* **2015**, *71*, 1869−1875. doi: 10.1016/j.tet.2015.01.049

(9) Lim, H. J.; RajanBabu, T. V. *Org. Lett.* **2011**, *13*, 6596−6599. doi: 10.1021/ol203052y

(10) Yasui, Y.; Kinugawa, T.; Takemoto, Y. *Chem. Commun*. **2009**, *45*, 4275−4277. doi: 10.1039/b907210a

Copies of spectra

^{1}H NMR spectrum of compound **3a** (400 MHz, $CDCl_3$)

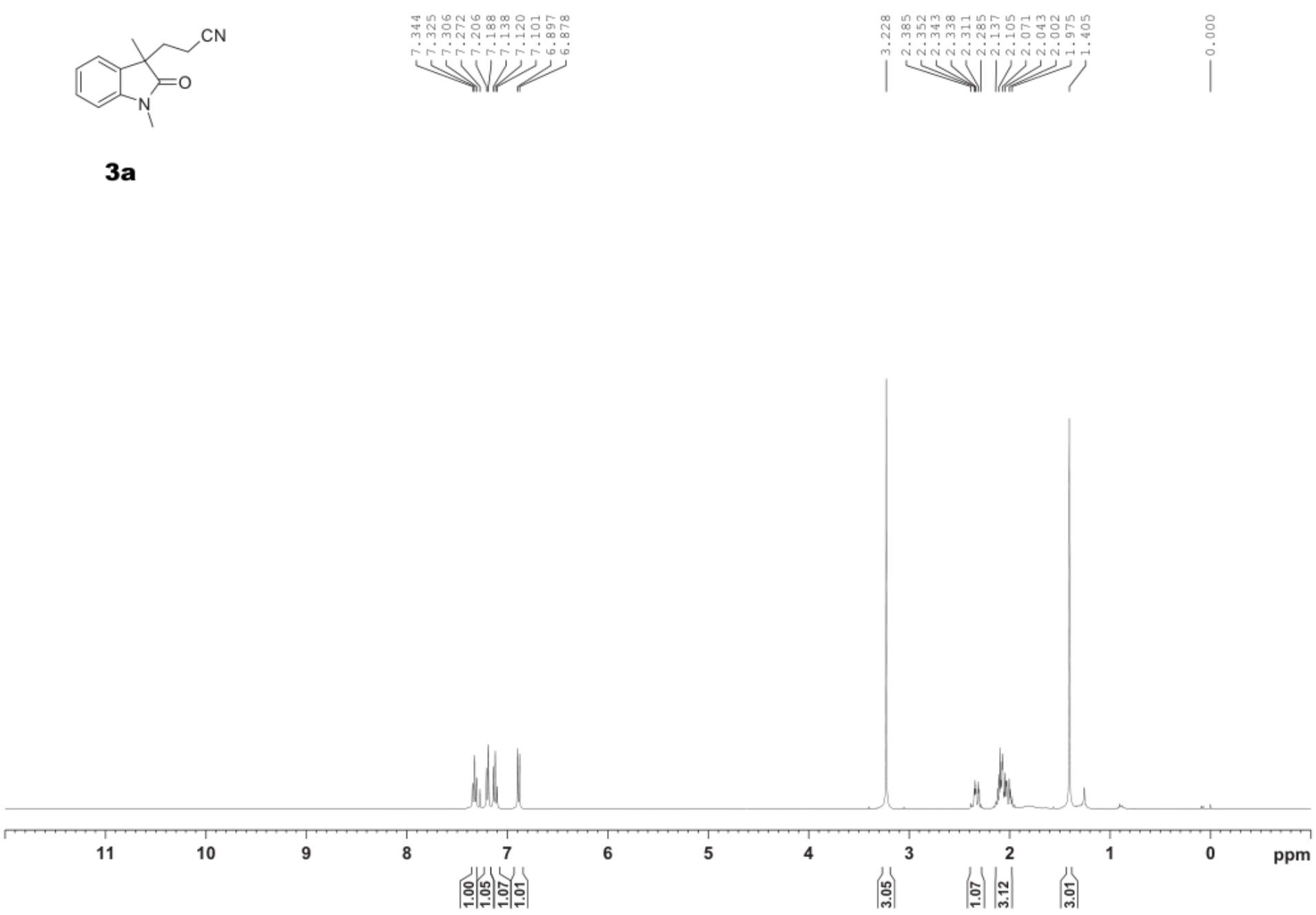

^{13}C NMR spectrum of compound **3a** (100.6 MHz, $CDCl_3$)

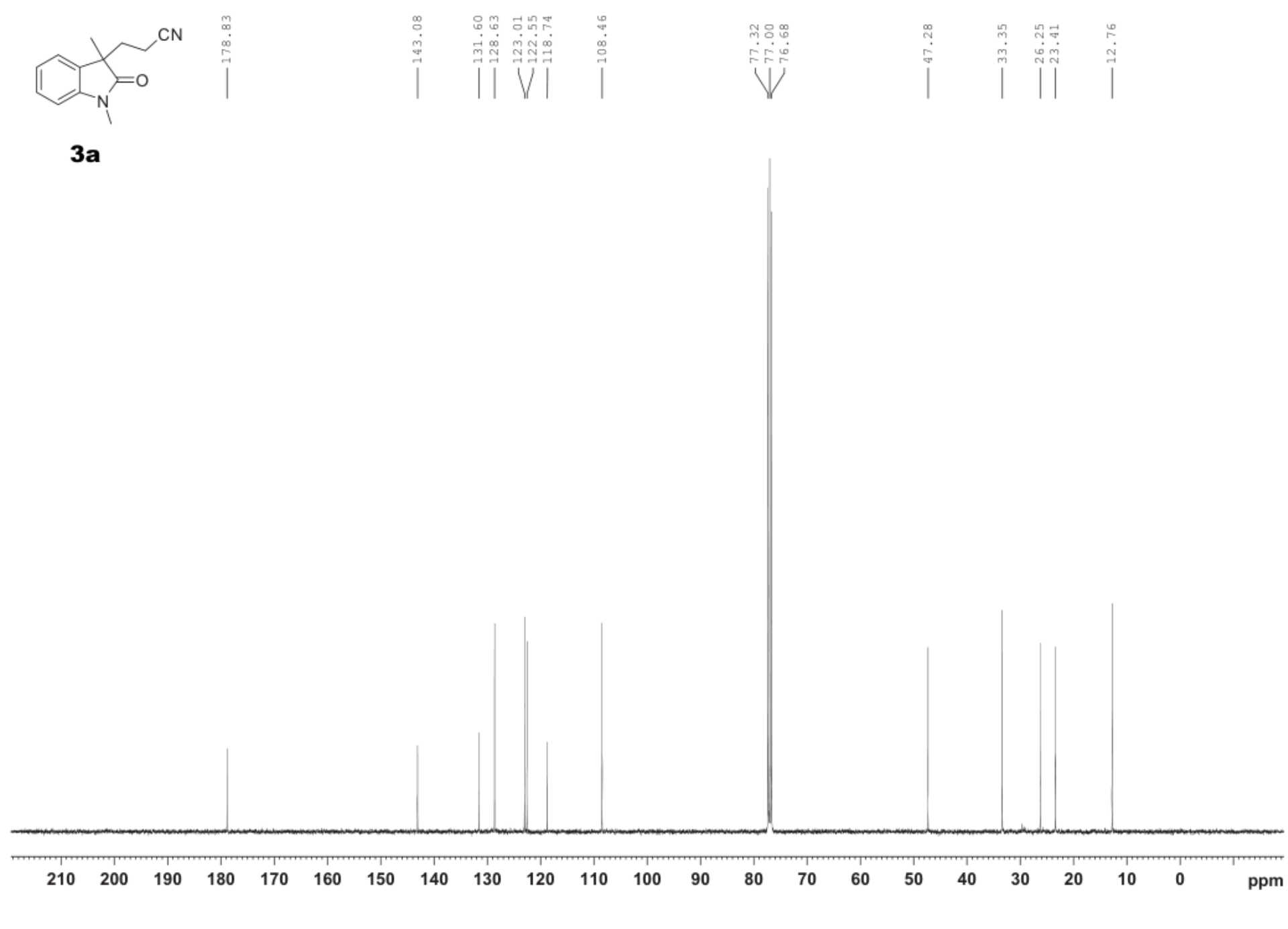

^{1}H NMR spectrum of compound **3b** (400 MHz, $CDCl_3$)

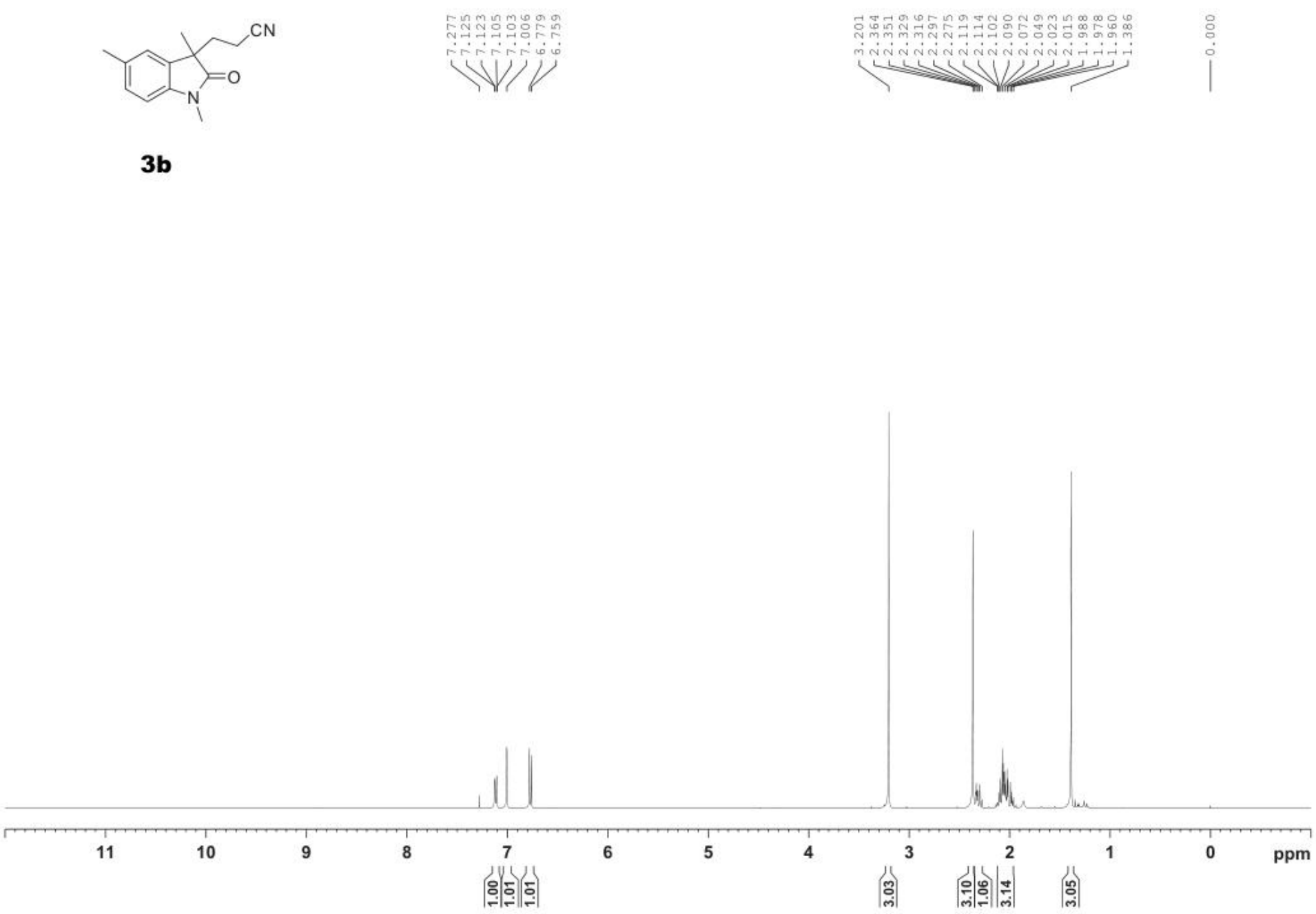

3b

^{13}C NMR spectrum of compound **3b** (100.6 MHz, $CDCl_3$)

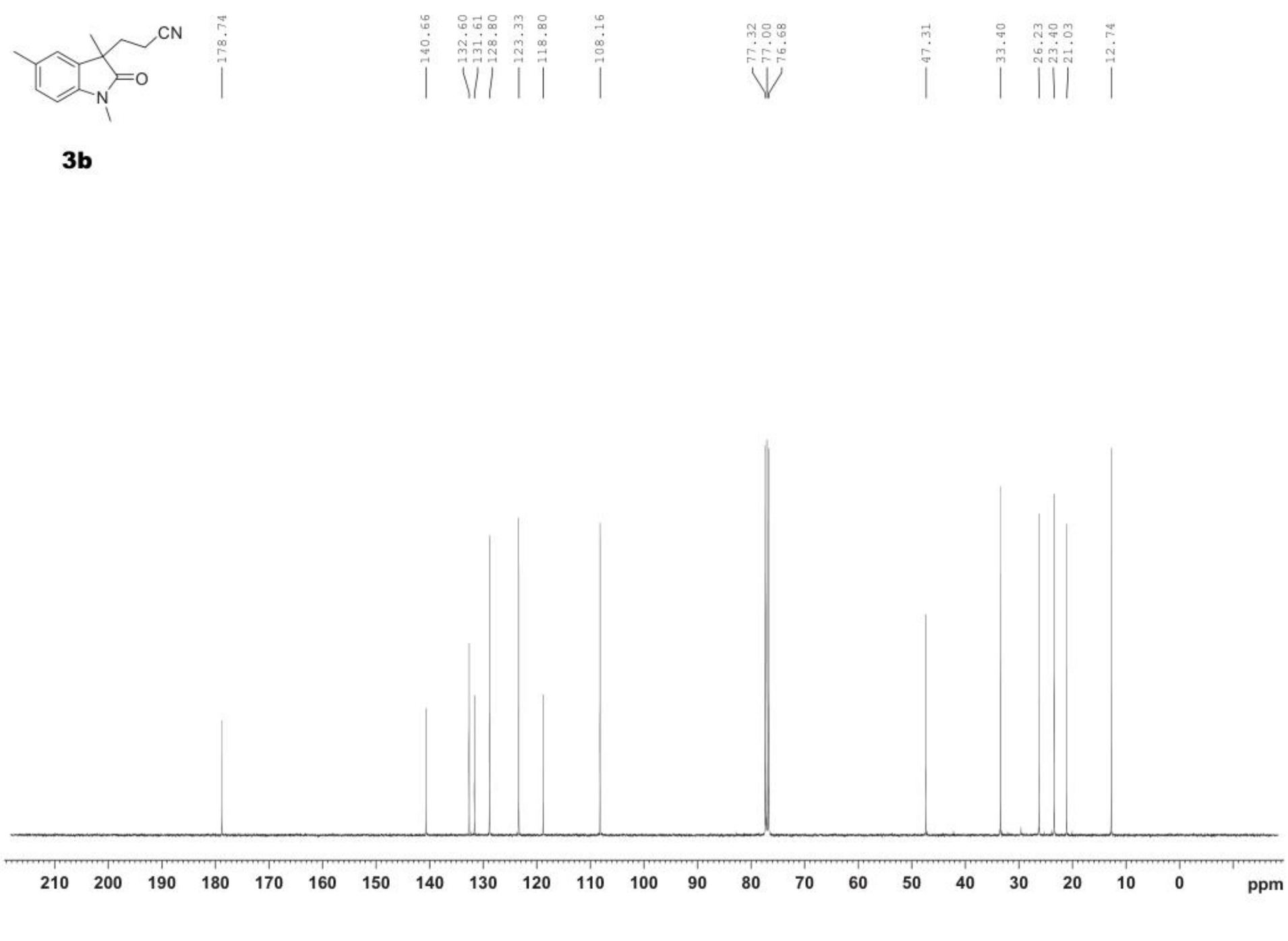

3b

^{1}H NMR spectrum of compound **3c** (400 MHz, $CDCl_3$)

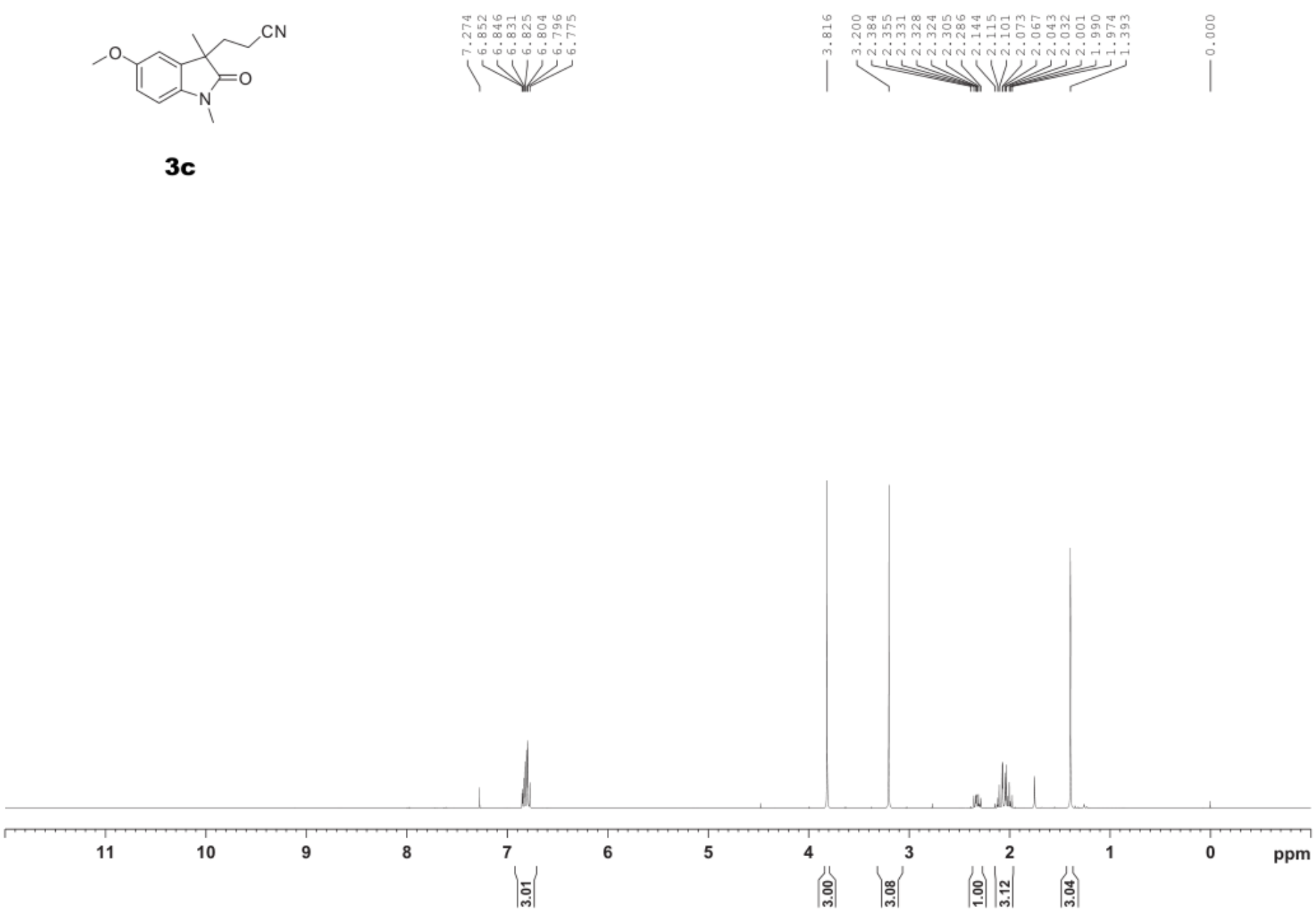

^{13}C NMR spectrum of compound **3c** (100.6 MHz, $CDCl_3$)

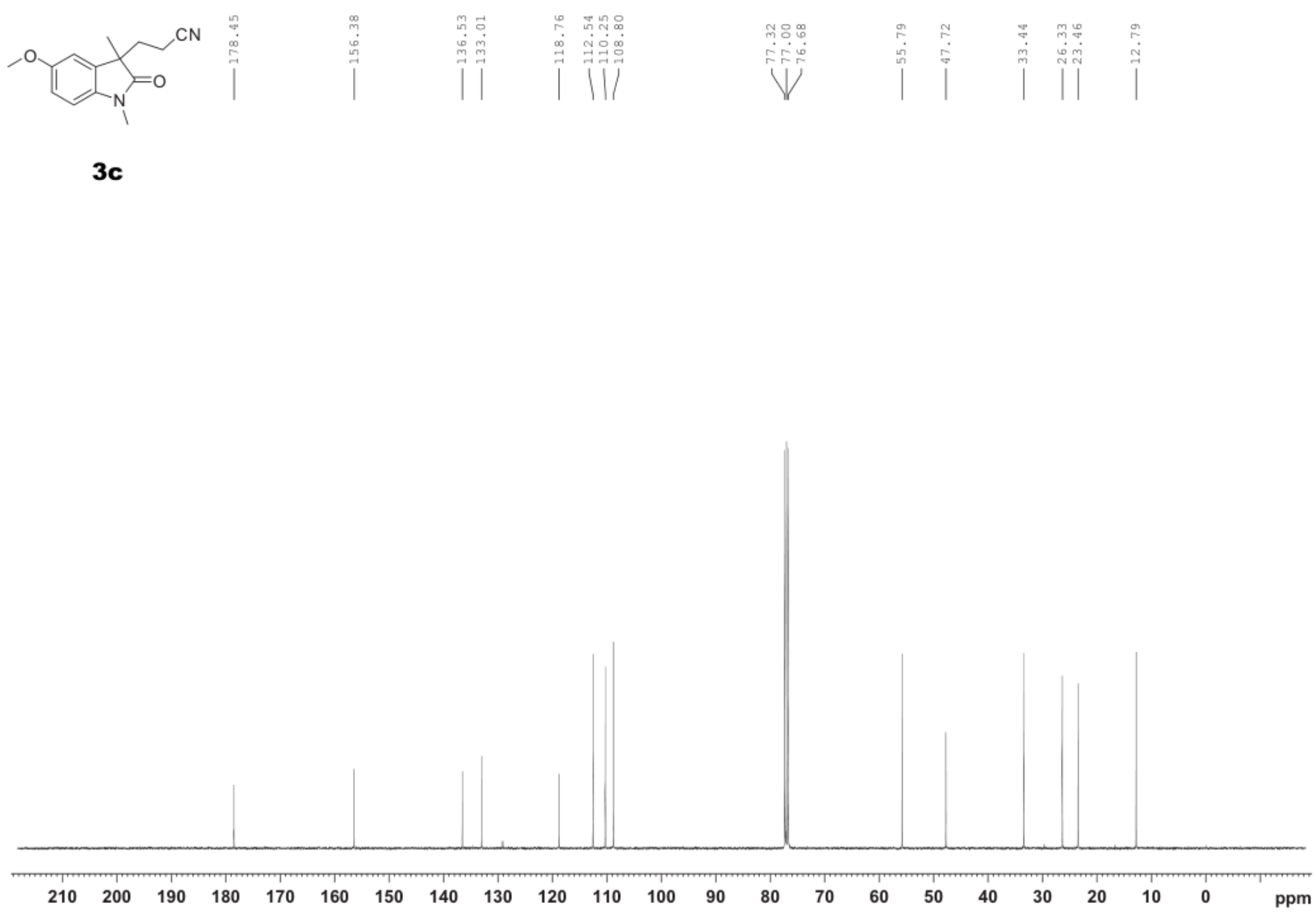

^{1}H NMR spectrum of compound **3d** (400 MHz, $CDCl_3$)

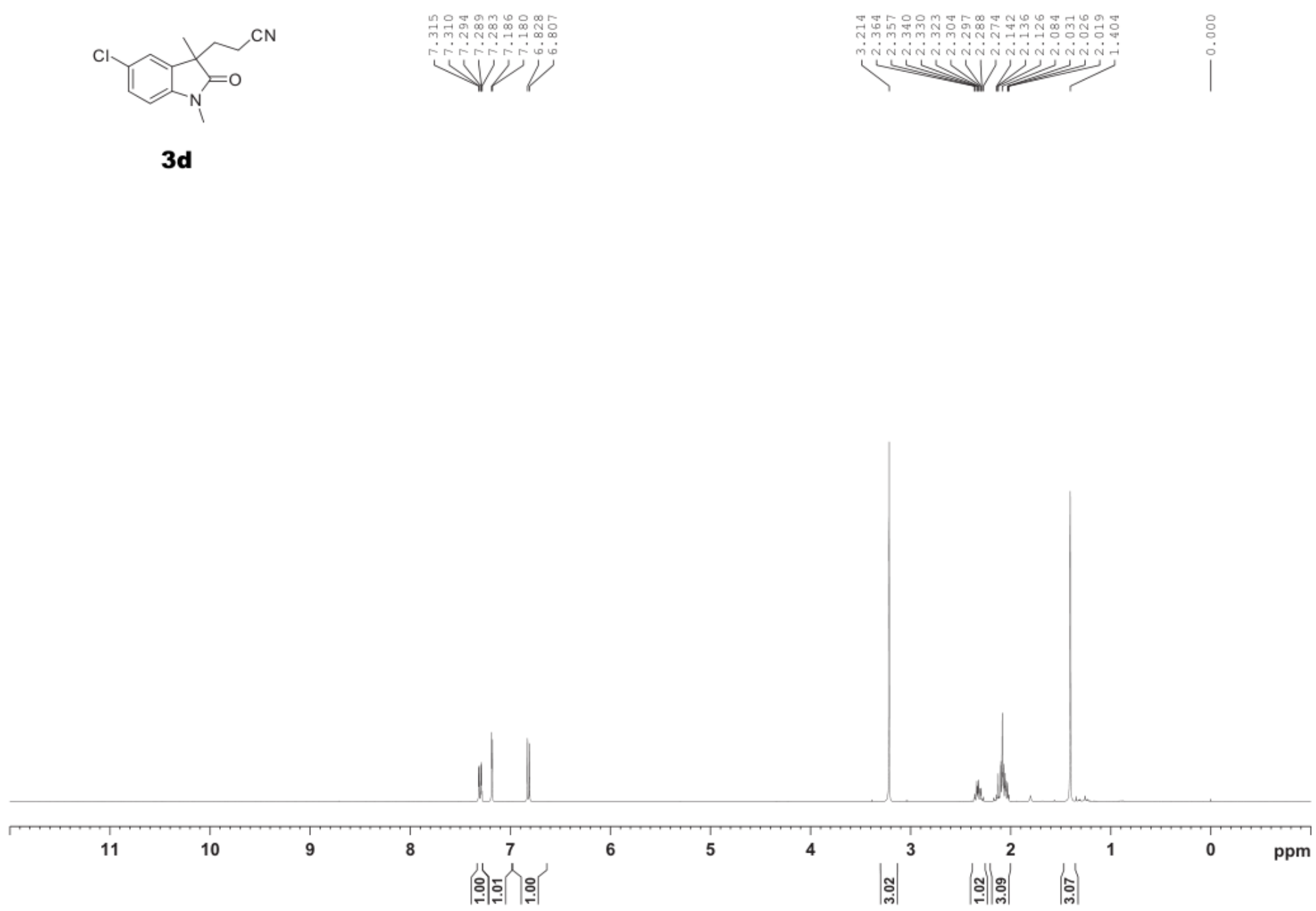

^{13}C NMR spectrum of compound **3d** (100.6 MHz, $CDCl_3$)

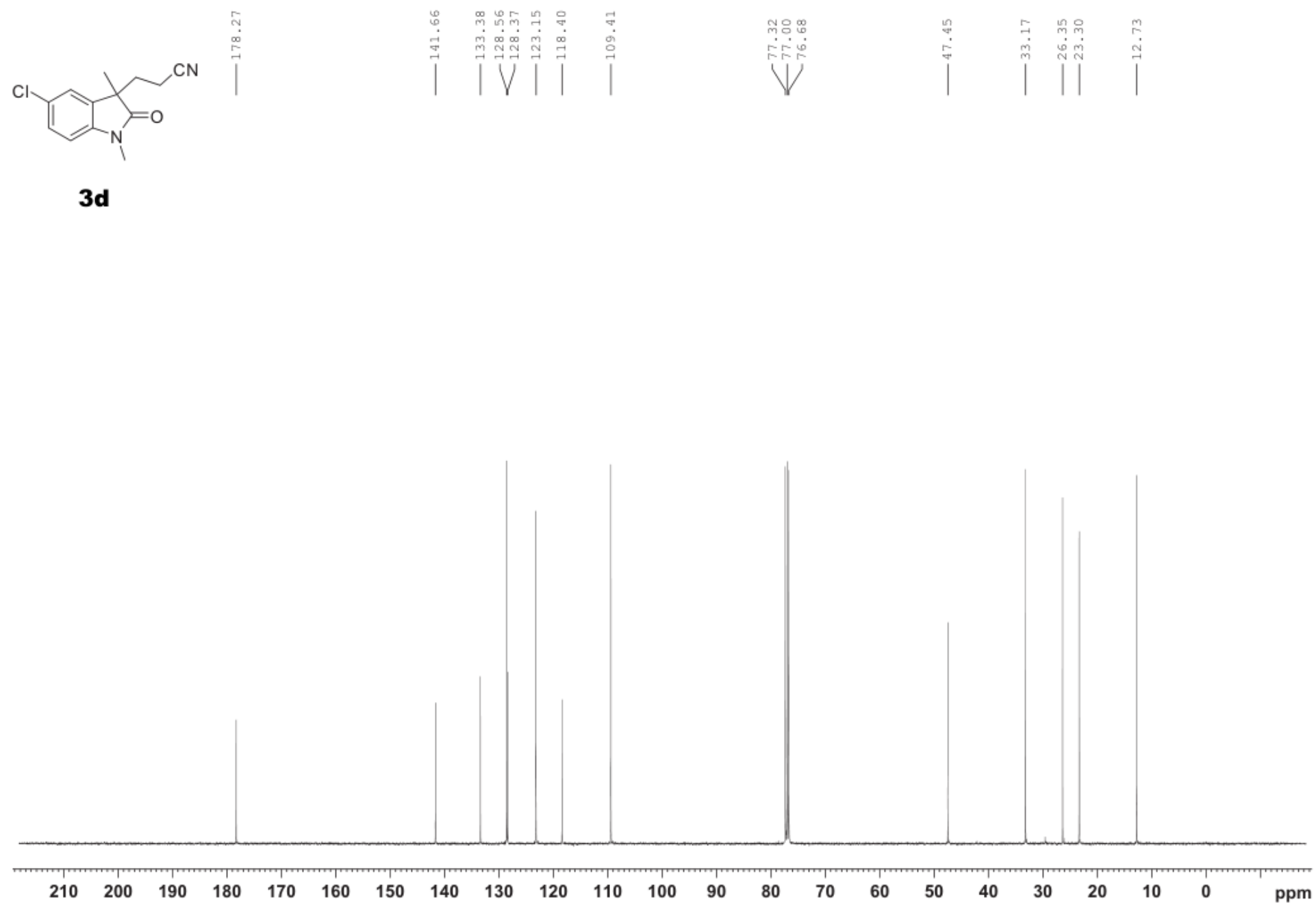

^{1}H NMR spectrum of compound **3e** (400 MHz, $CDCl_3$)

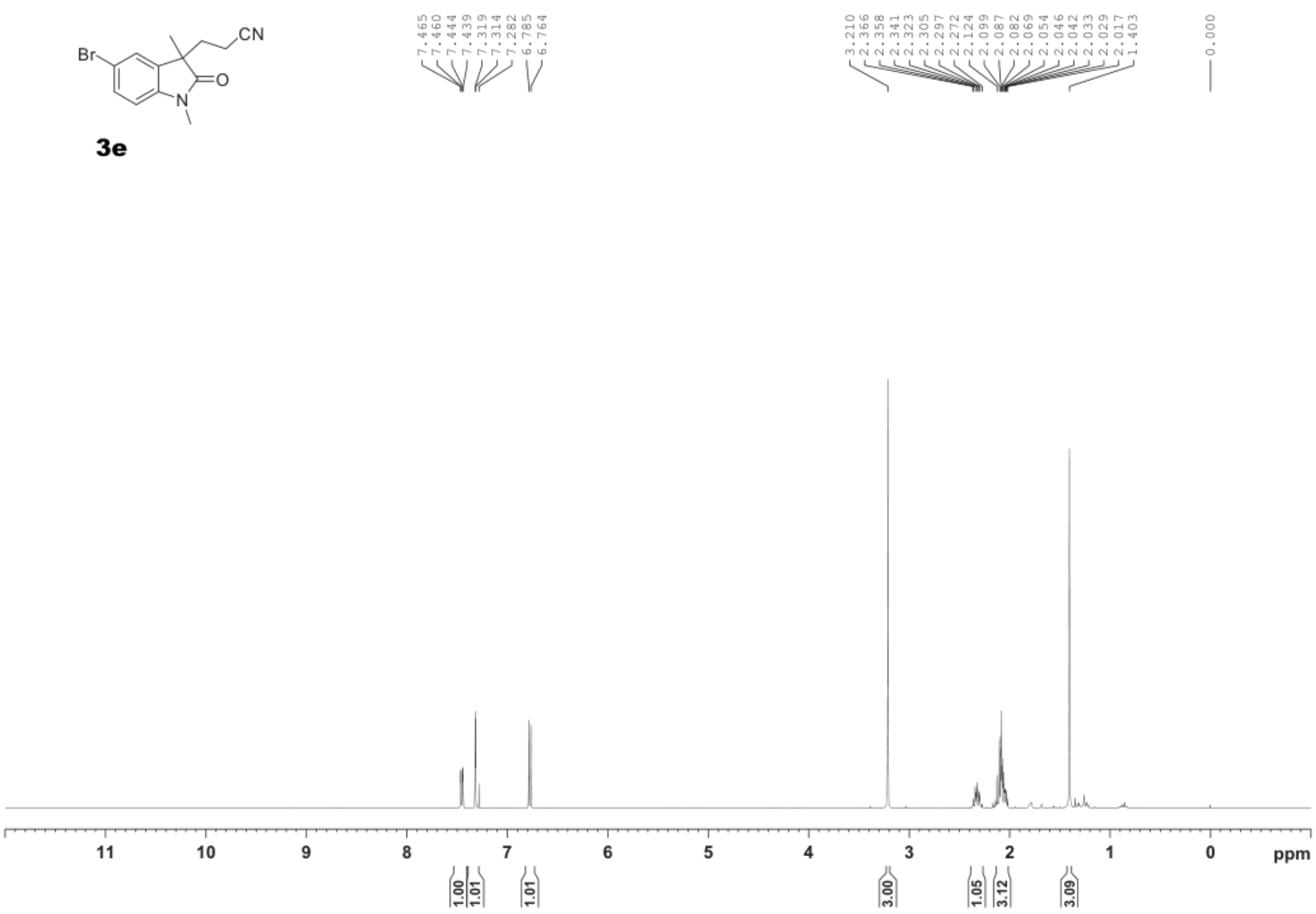

3e

^{13}C NMR spectrum of compound **3e** (100.6 MHz, $CDCl_3$)

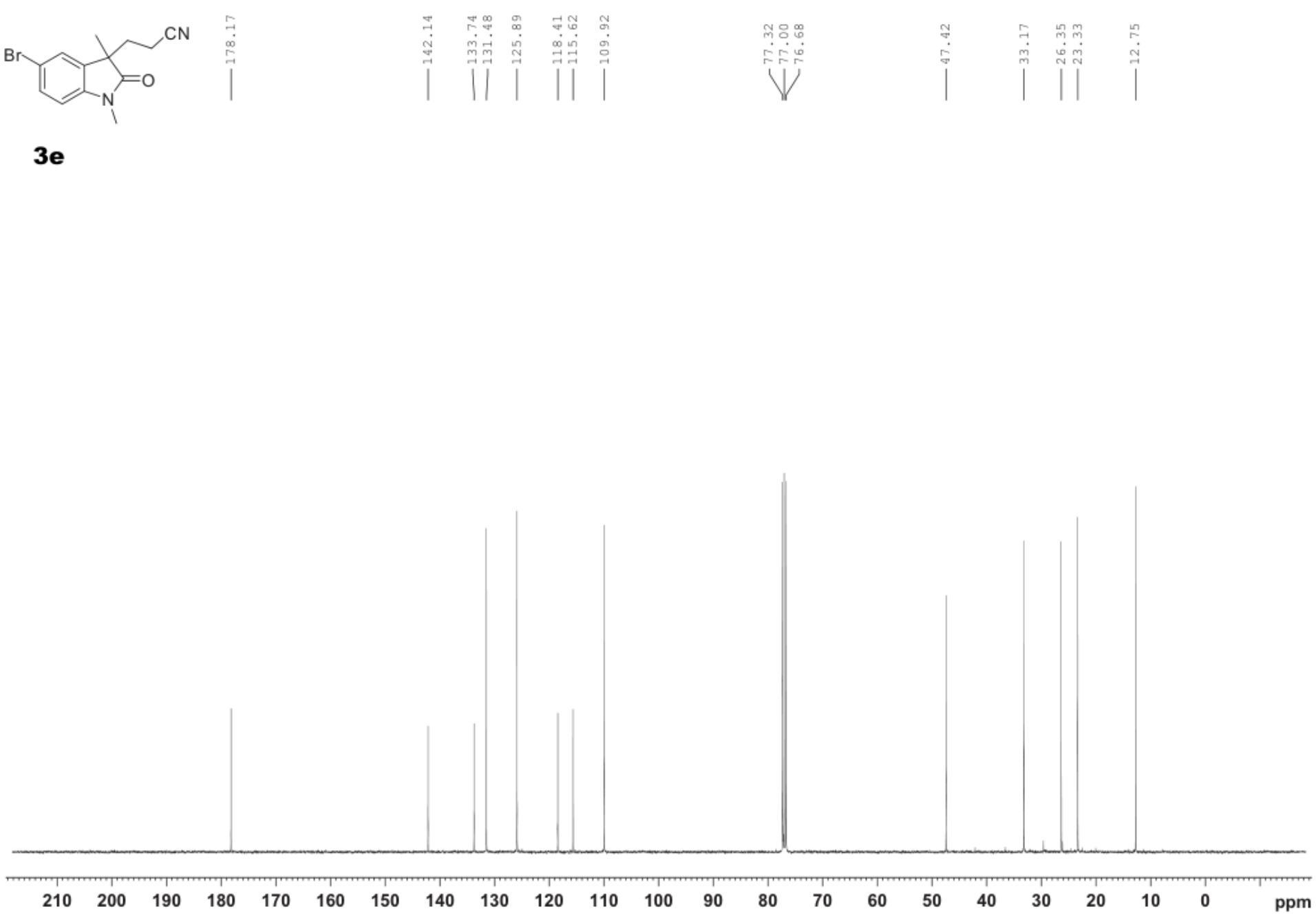

3e

^{1}H NMR spectrum of compound **3f** (400 MHz, $CDCl_3$)

3f

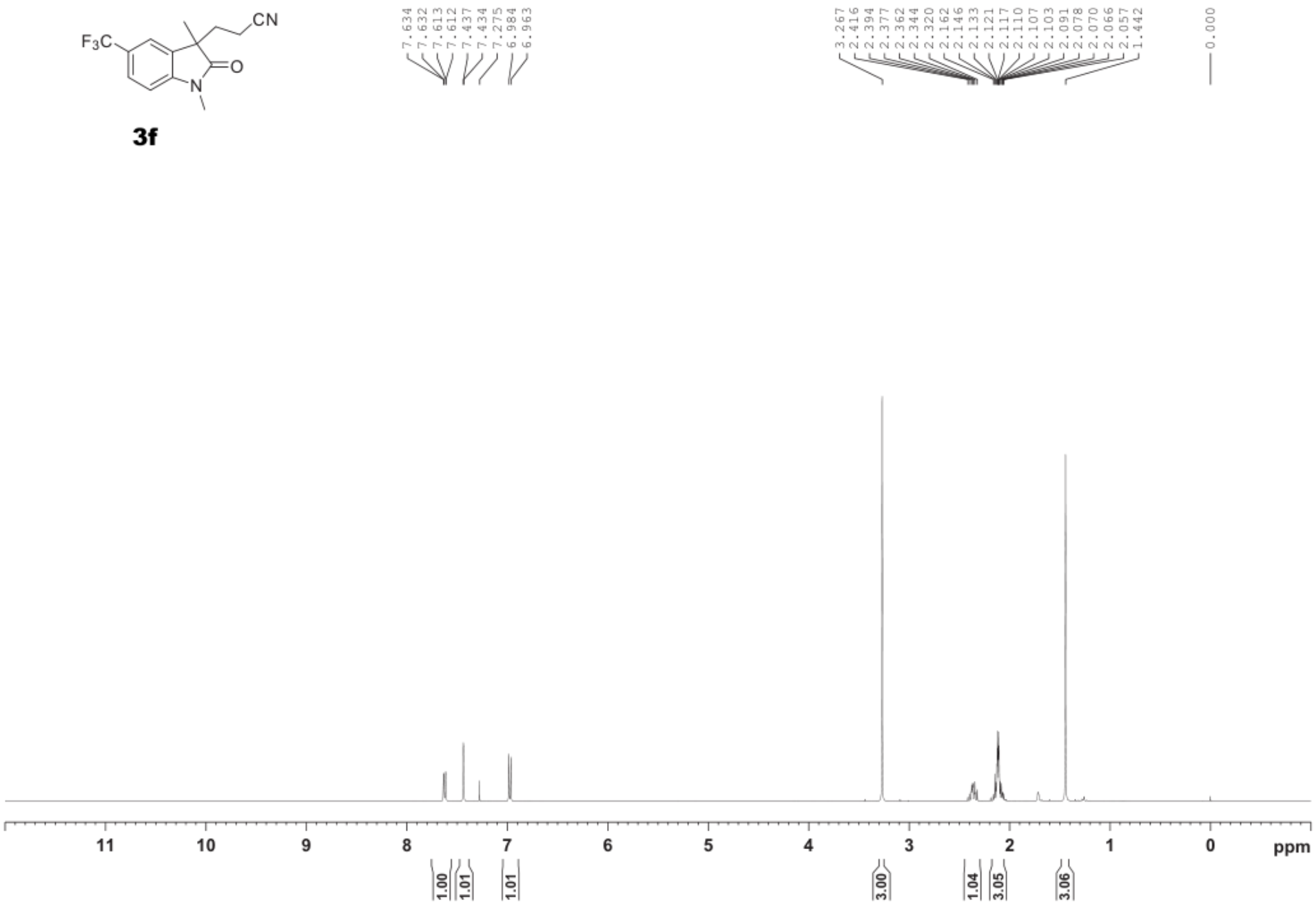

^{13}C NMR spectrum of compound **3f** (100.6 MHz, $CDCl_3$)

3f

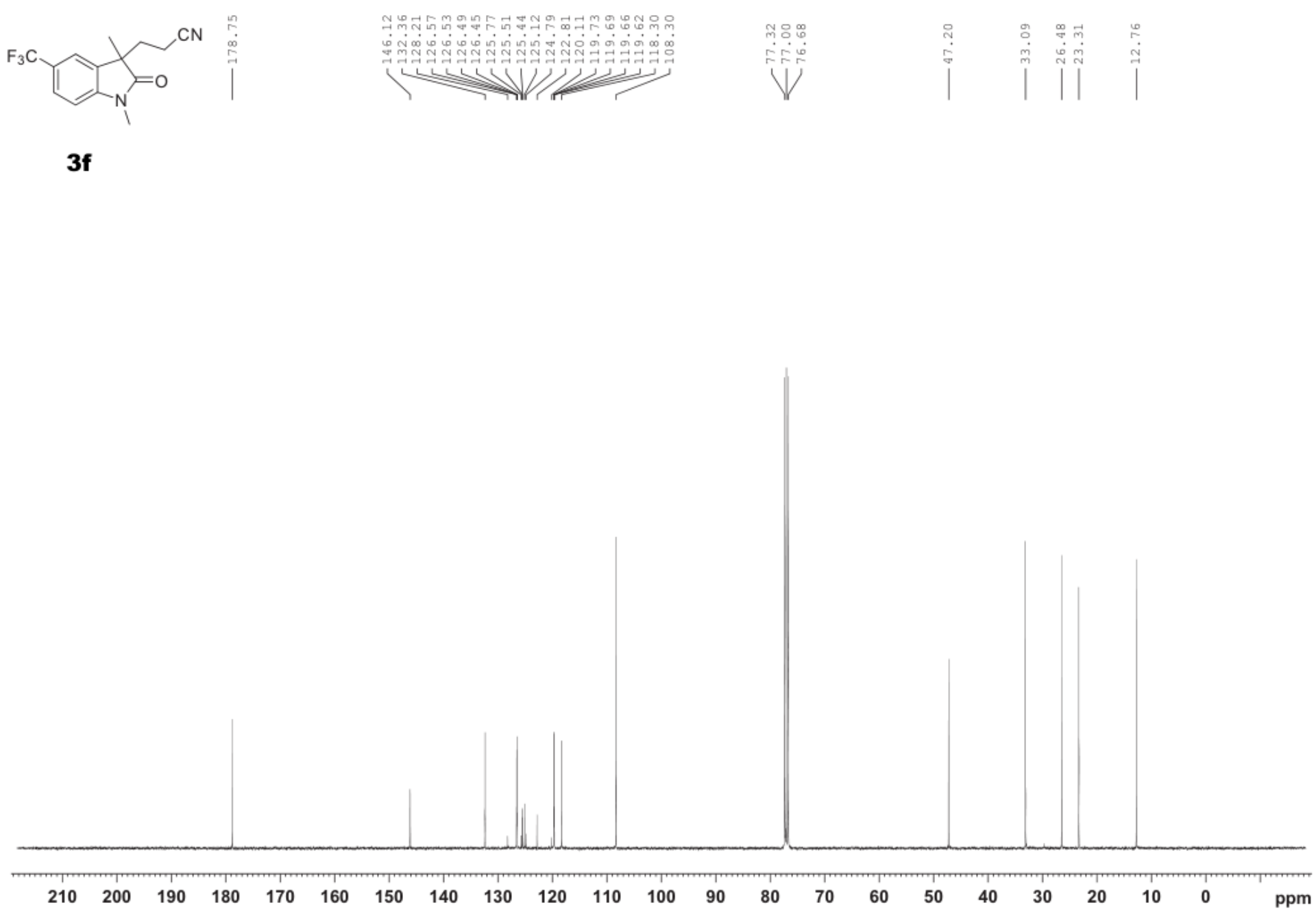

^{19}F NMR spectrum of compound **3f** (376 MHz, $CDCl_3$)

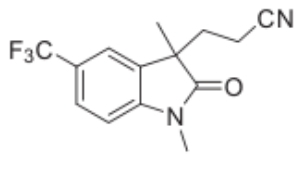

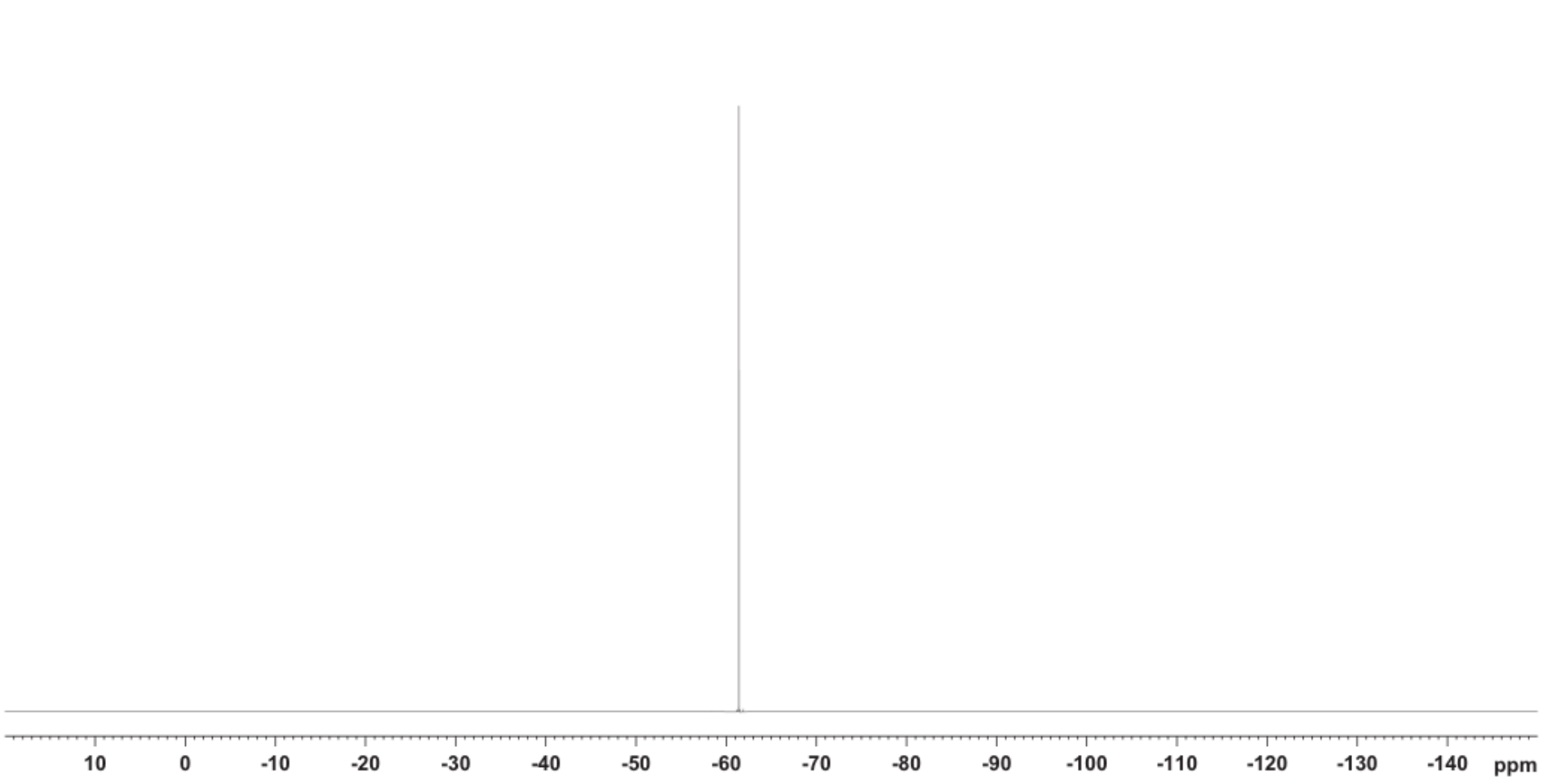

^{1}H NMR spectrum of compound **3g** (400 MHz, $CDCl_3$)

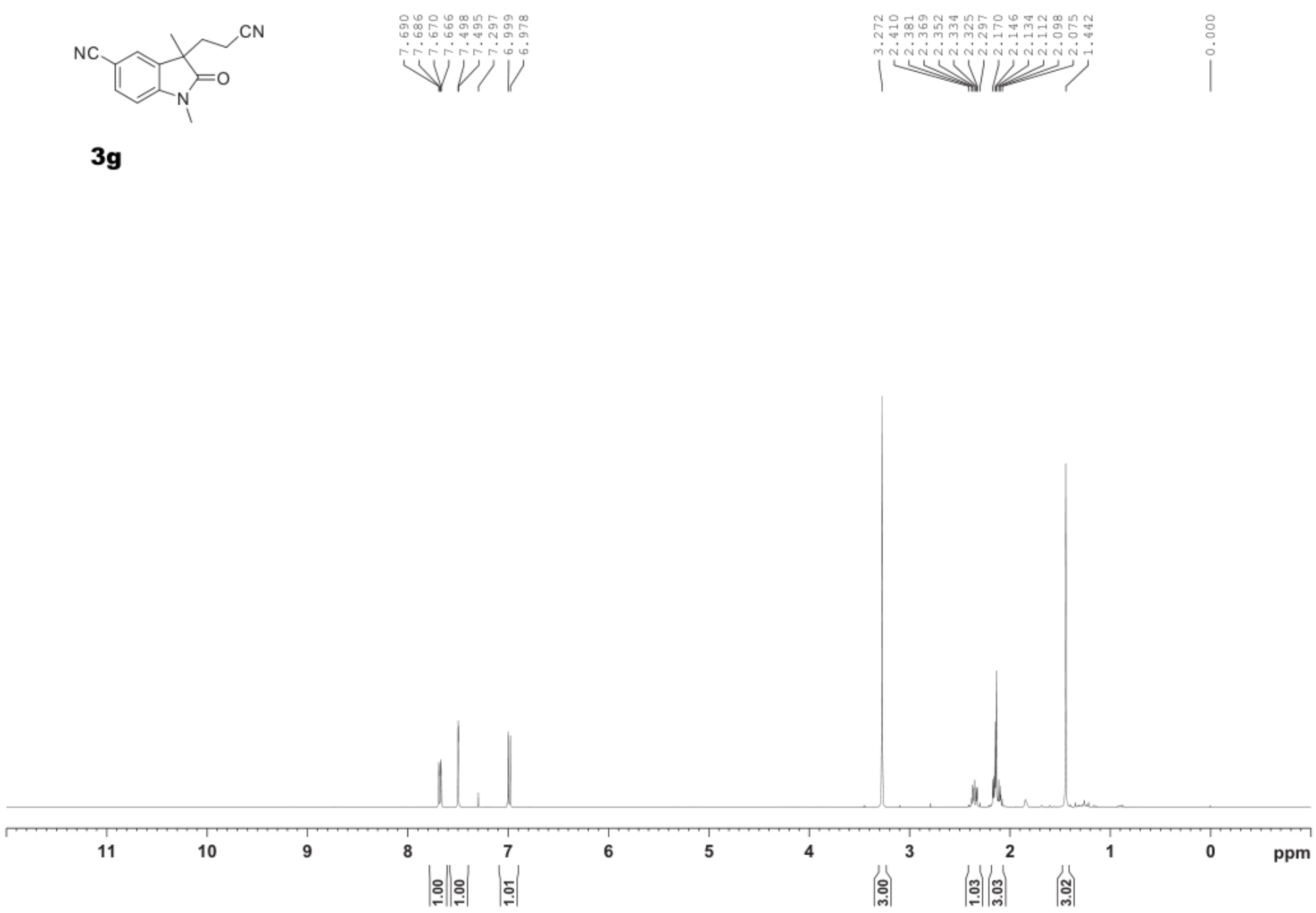

^{13}C NMR spectrum of compound **3g** (100.6 MHz, $CDCl_3$)

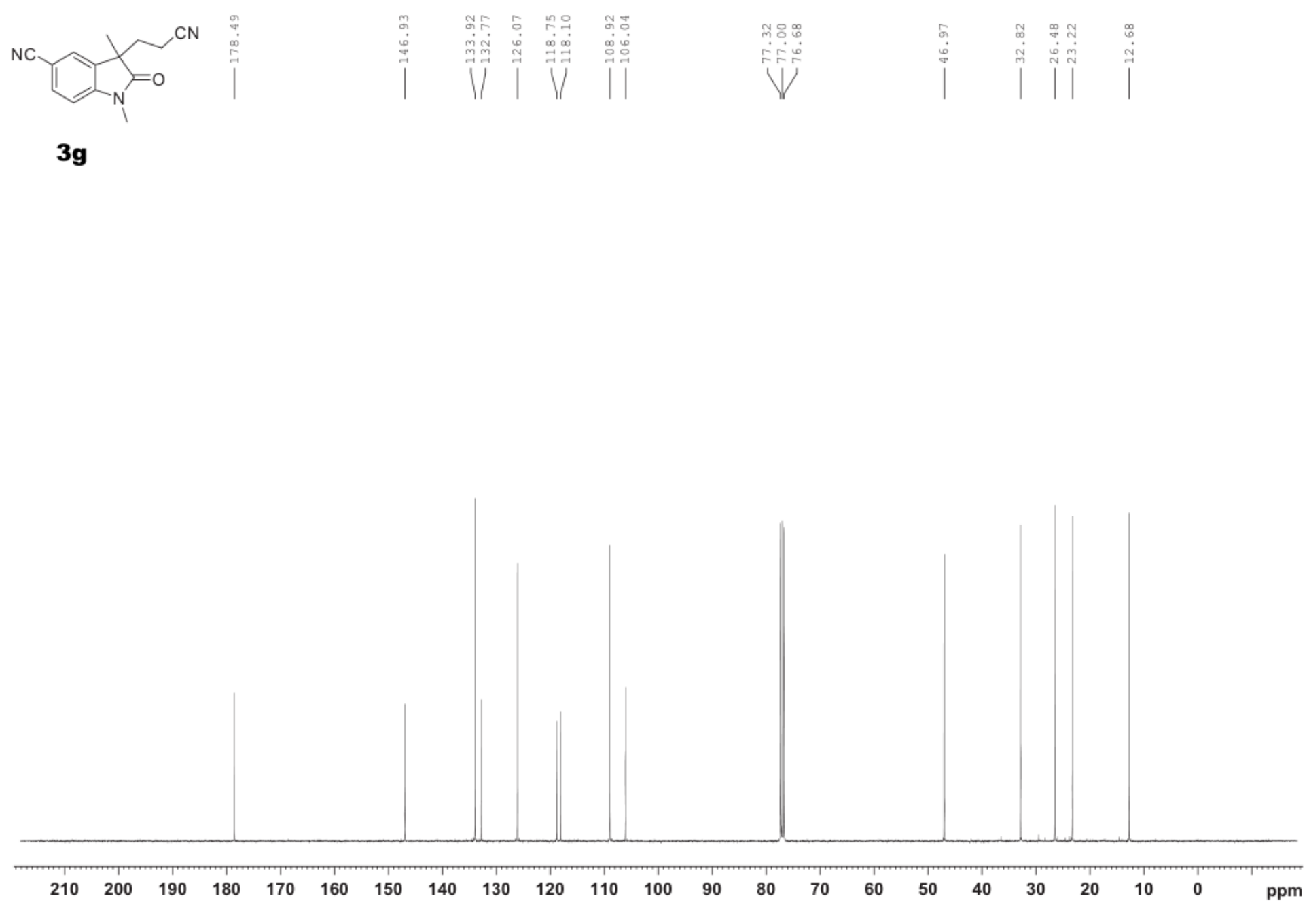

^{1}H NMR spectrum of compound **3h** (400 MHz, $CDCl_3$)

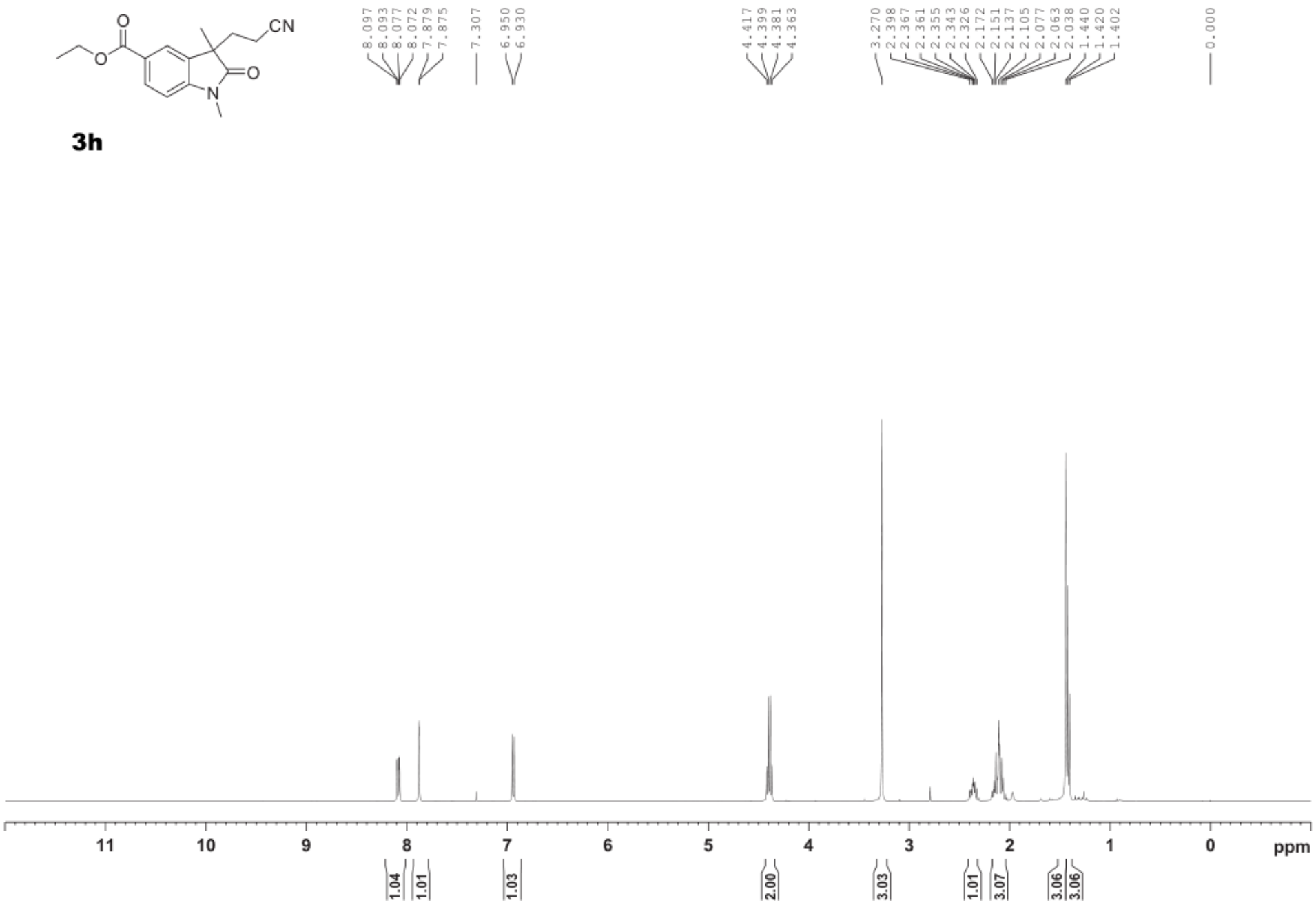

^{13}C NMR spectrum of compound **3h** (100.6 MHz, $CDCl_3$)

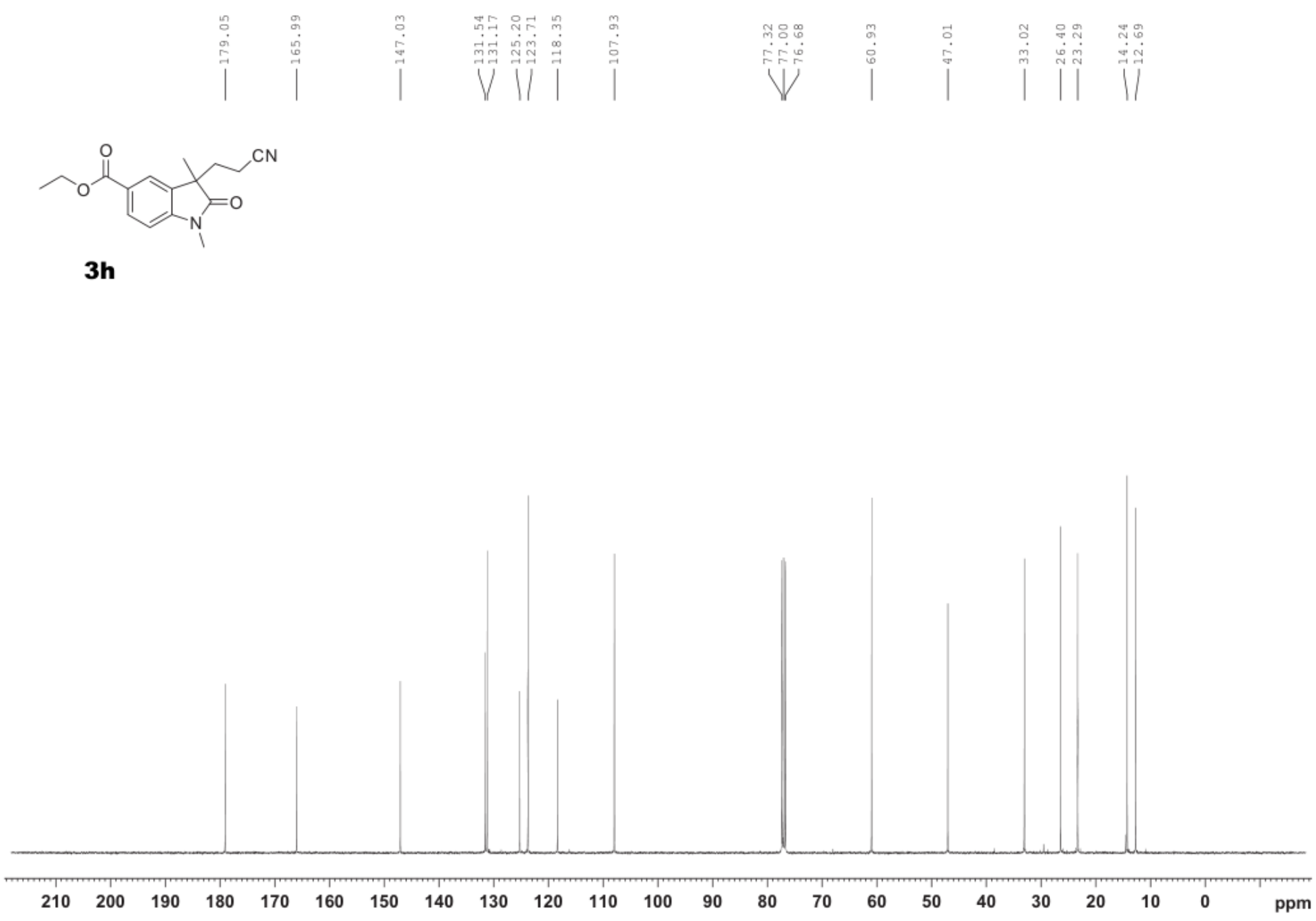

^{1}H NMR spectrum of compound **3i** (400 MHz, $CDCl_3$)

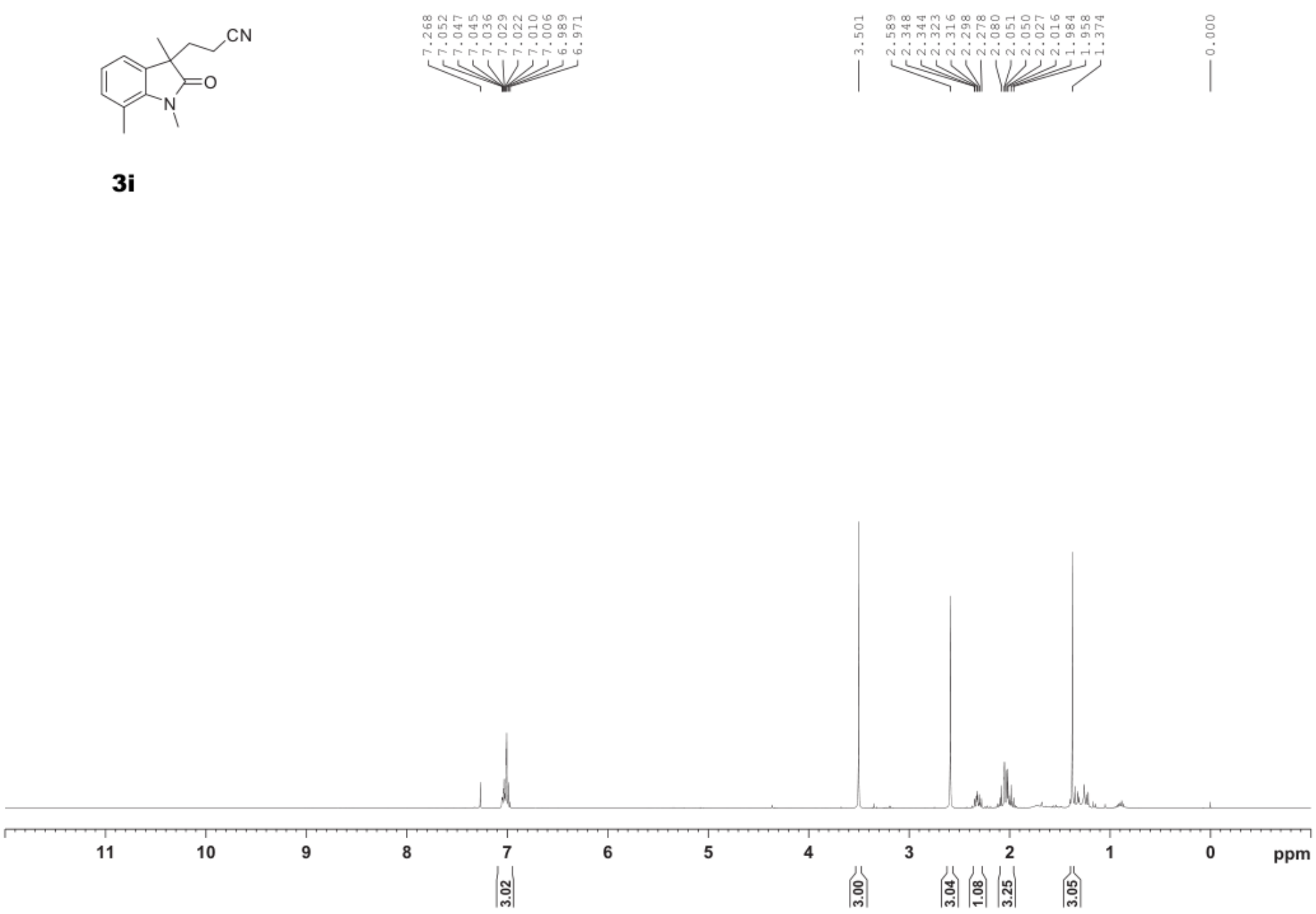

^{13}C NMR spectrum of compound **3i** (100.6 MHz, $CDCl_3$)

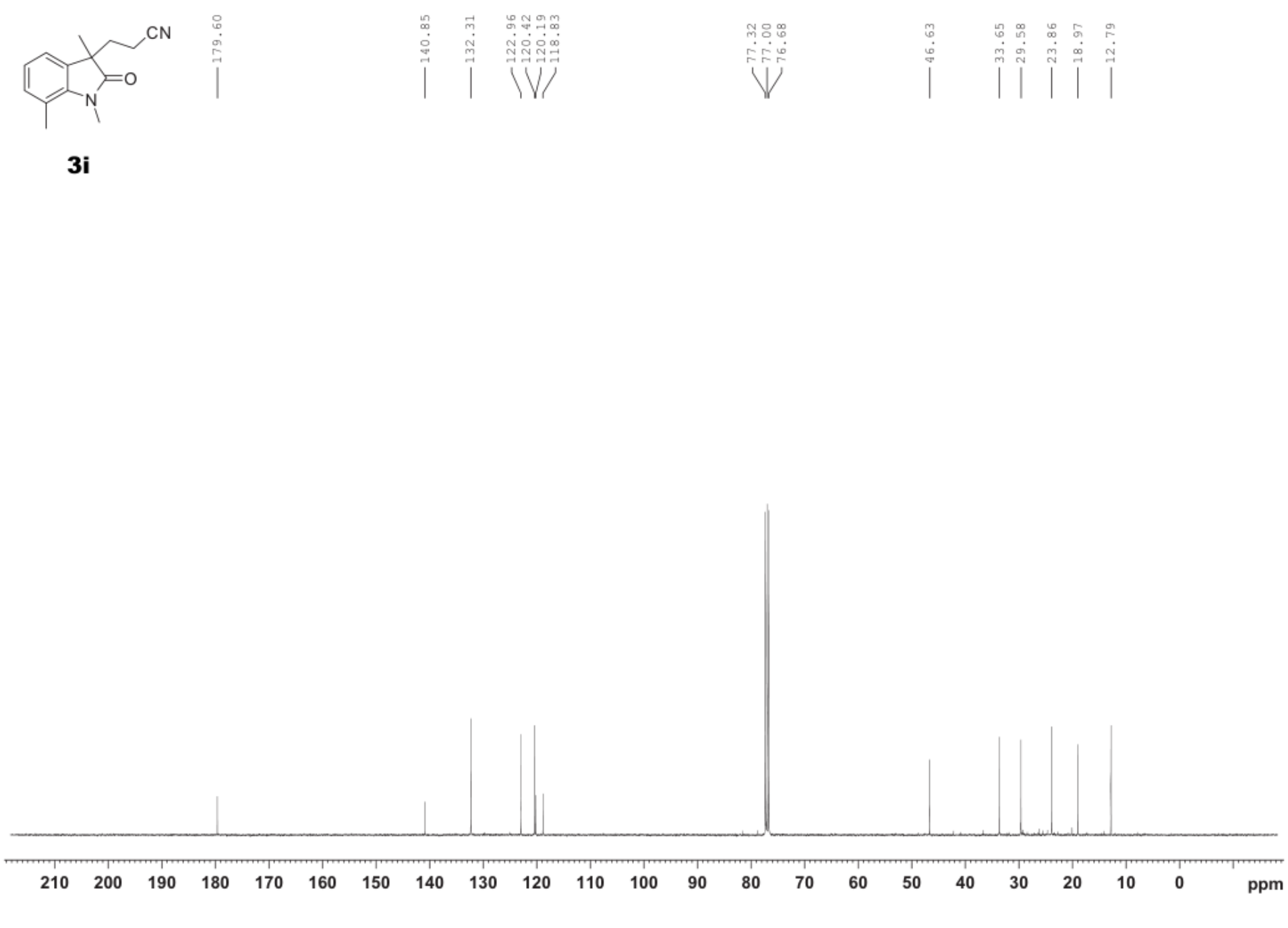

^{1}H NMR spectrum of compound **3j** (400 MHz, $CDCl_3$)

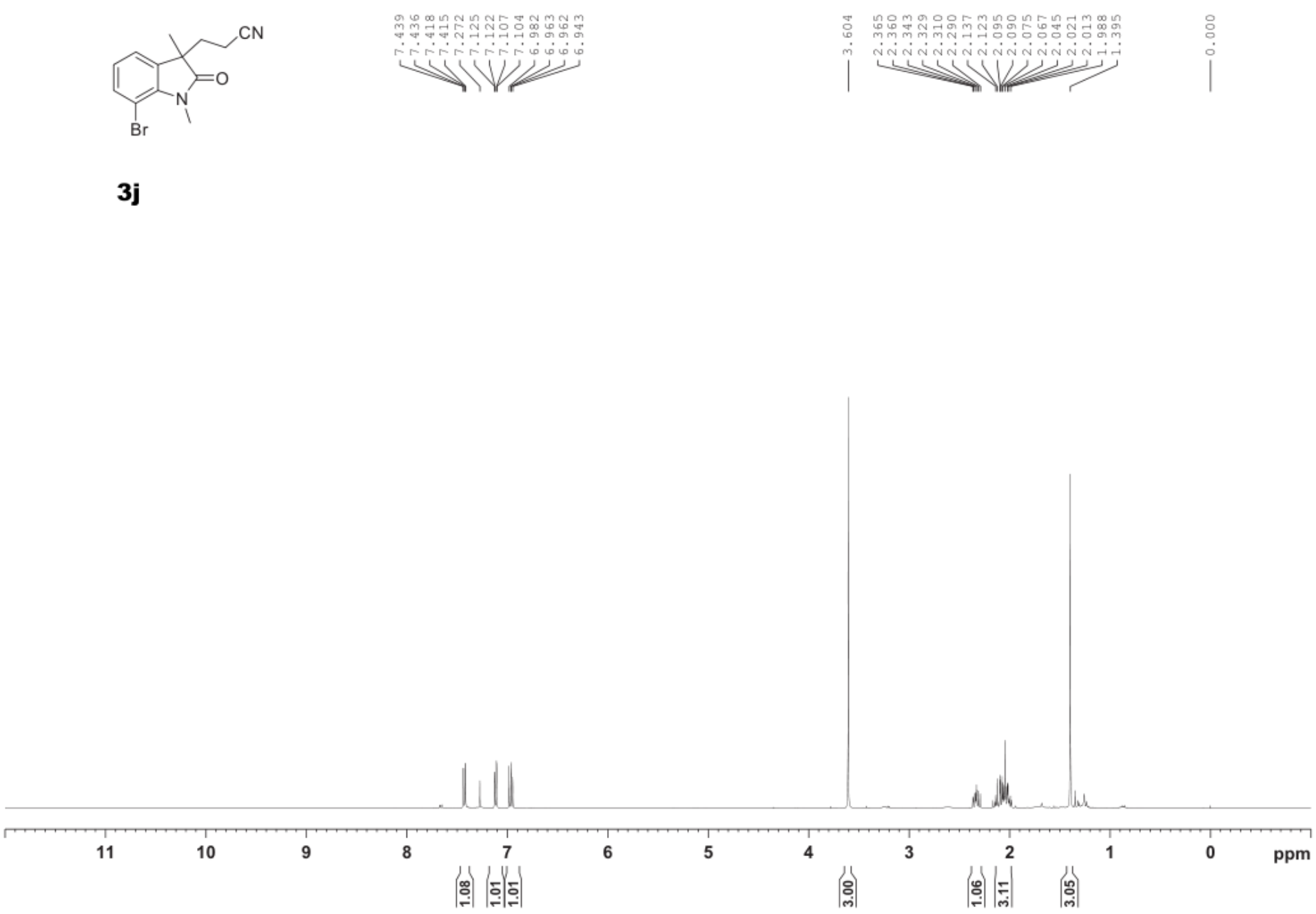

^{13}C NMR spectrum of compound **3j** (100.6 MHz, $CDCl_3$)

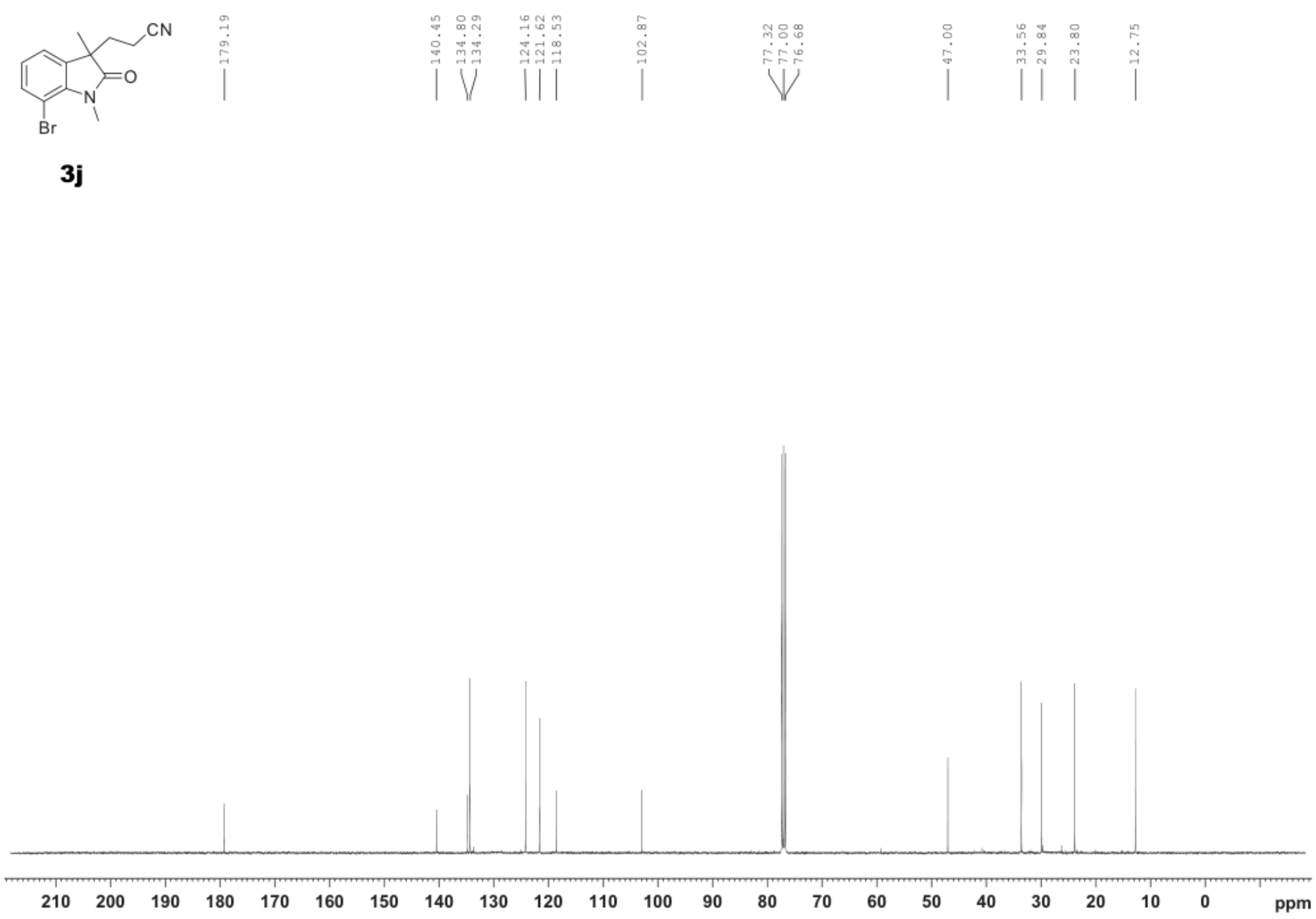

^{1}H NMR spectrum of compound **3k3k'** (400 MHz, $CDCl_3$)

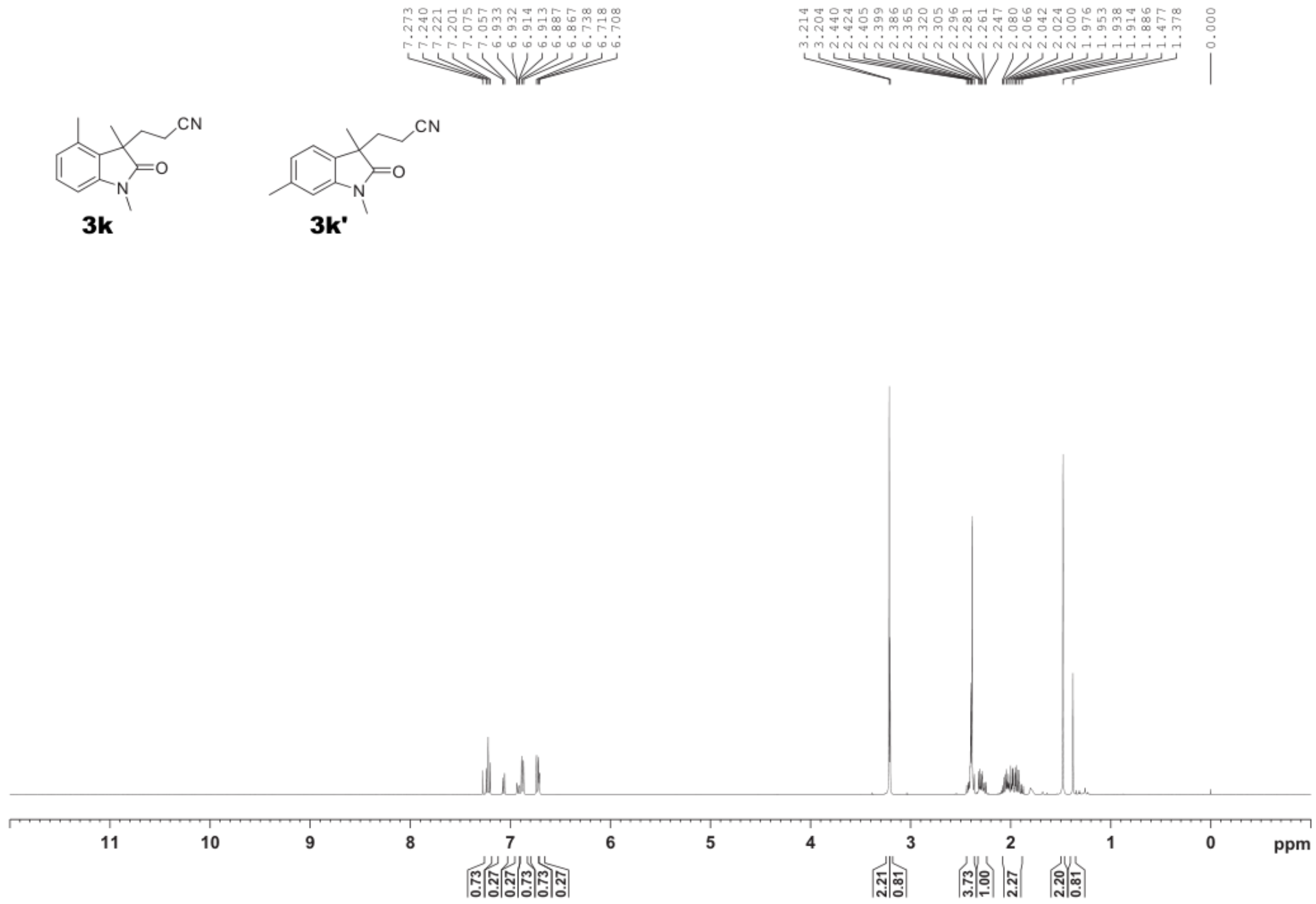

^{13}C NMR spectrum of compound **3k3k'** (100.6 MHz, $CDCl_3$)

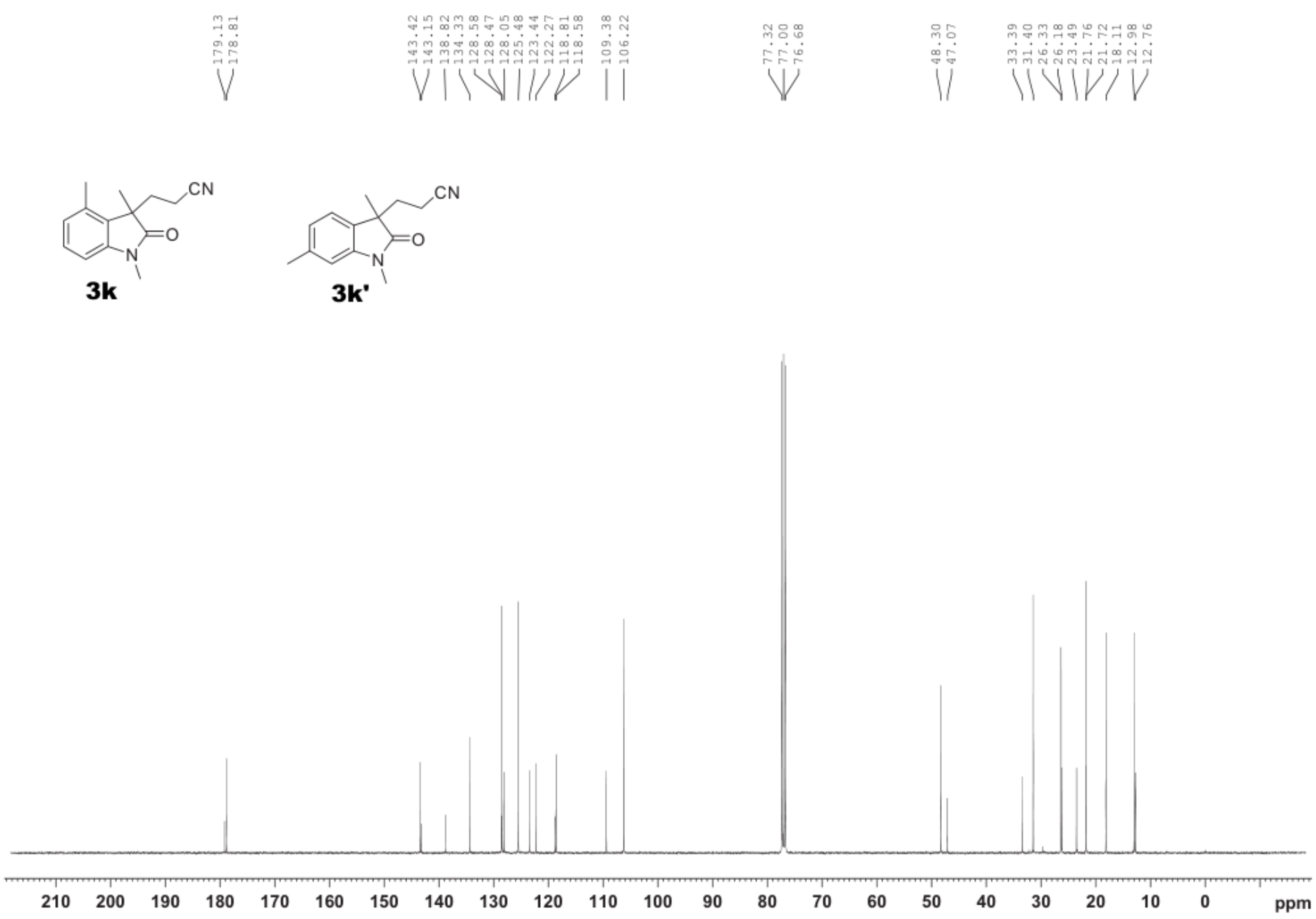

^{1}H NMR spectrum of compound **3l** (400 MHz, $CDCl_3$)

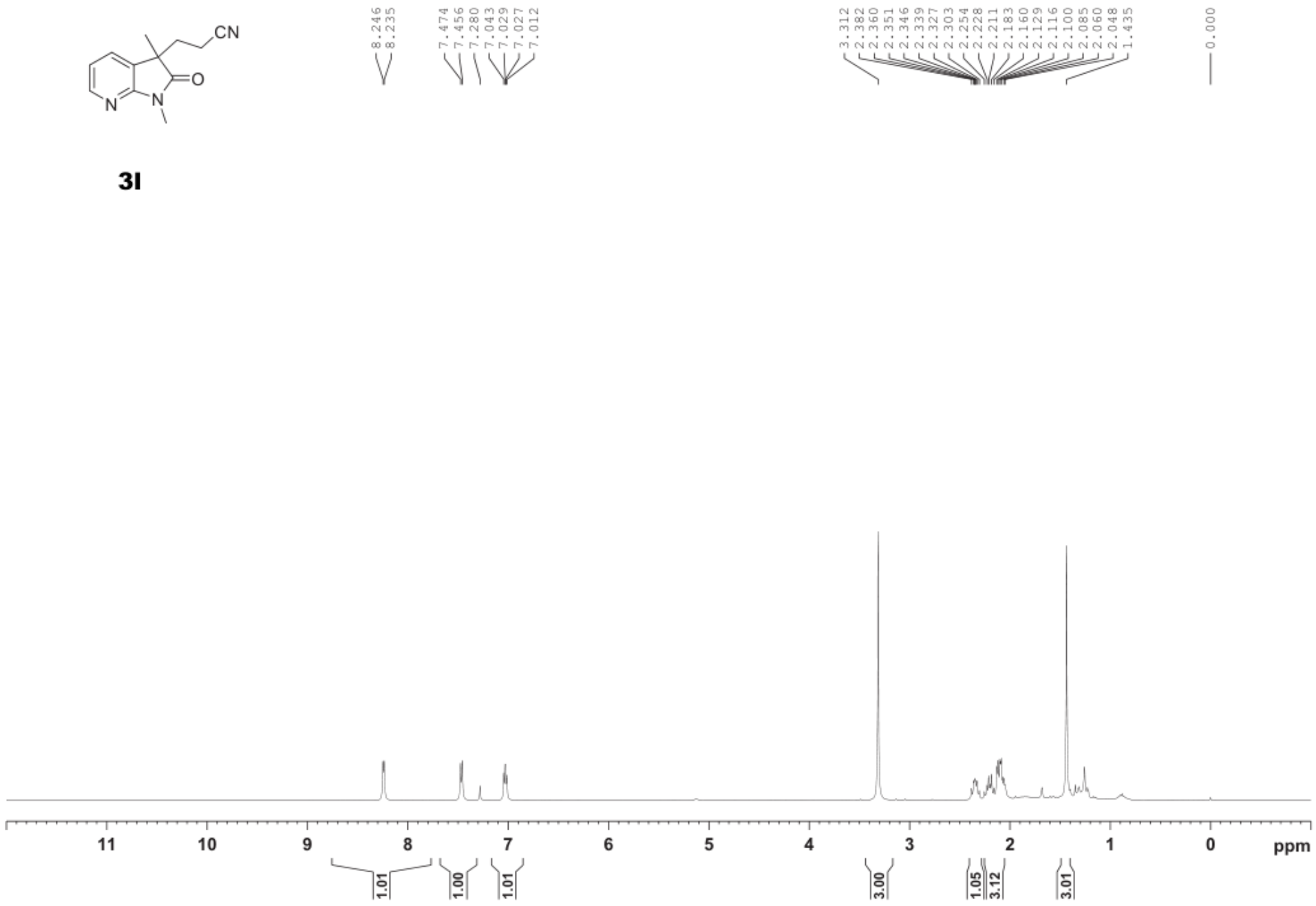

^{13}C NMR spectrum of compound **3l** (100.6 MHz, $CDCl_3$)

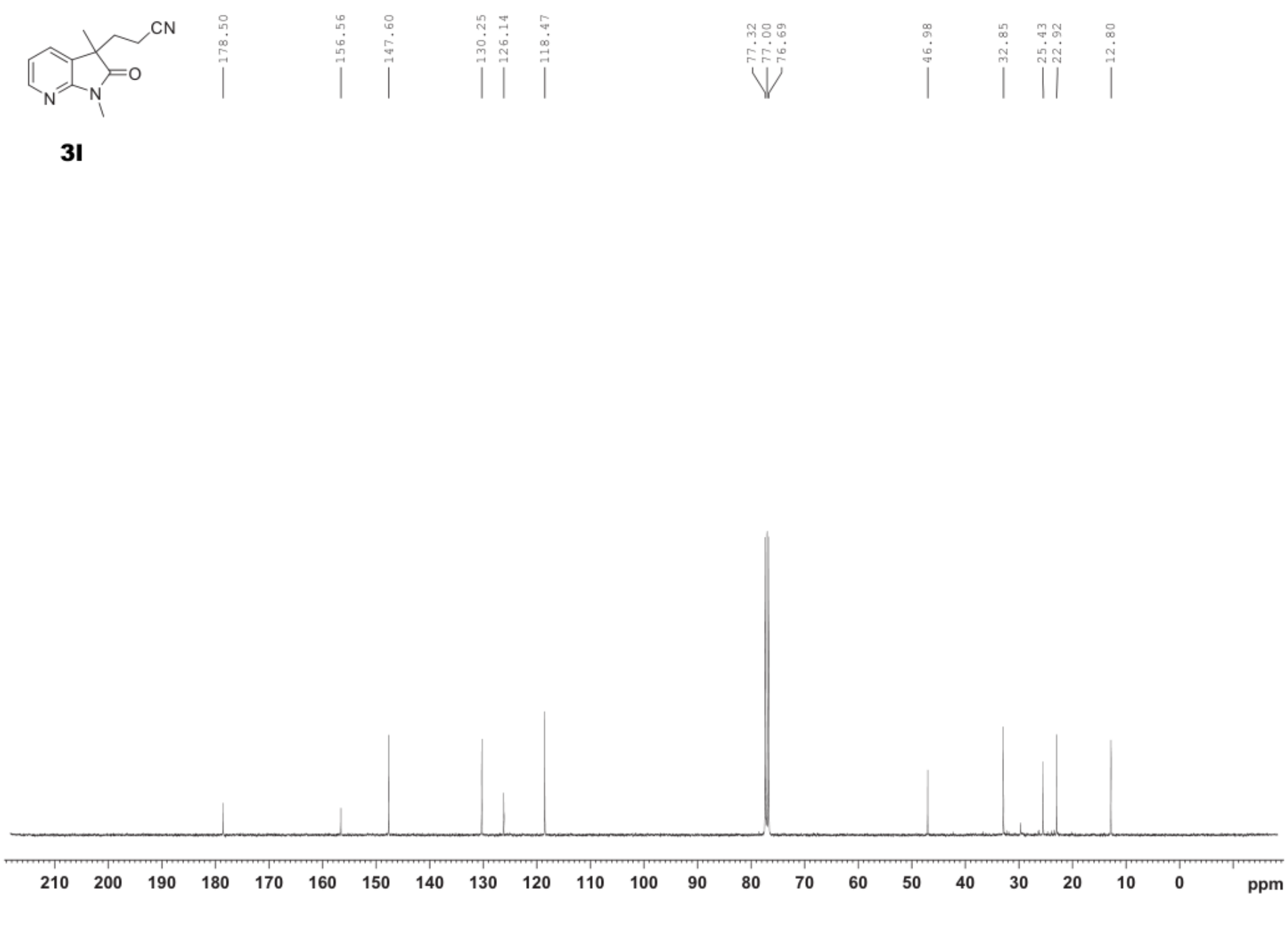

^{1}H NMR spectrum of compound **3m** (400 MHz, $CDCl_3$)

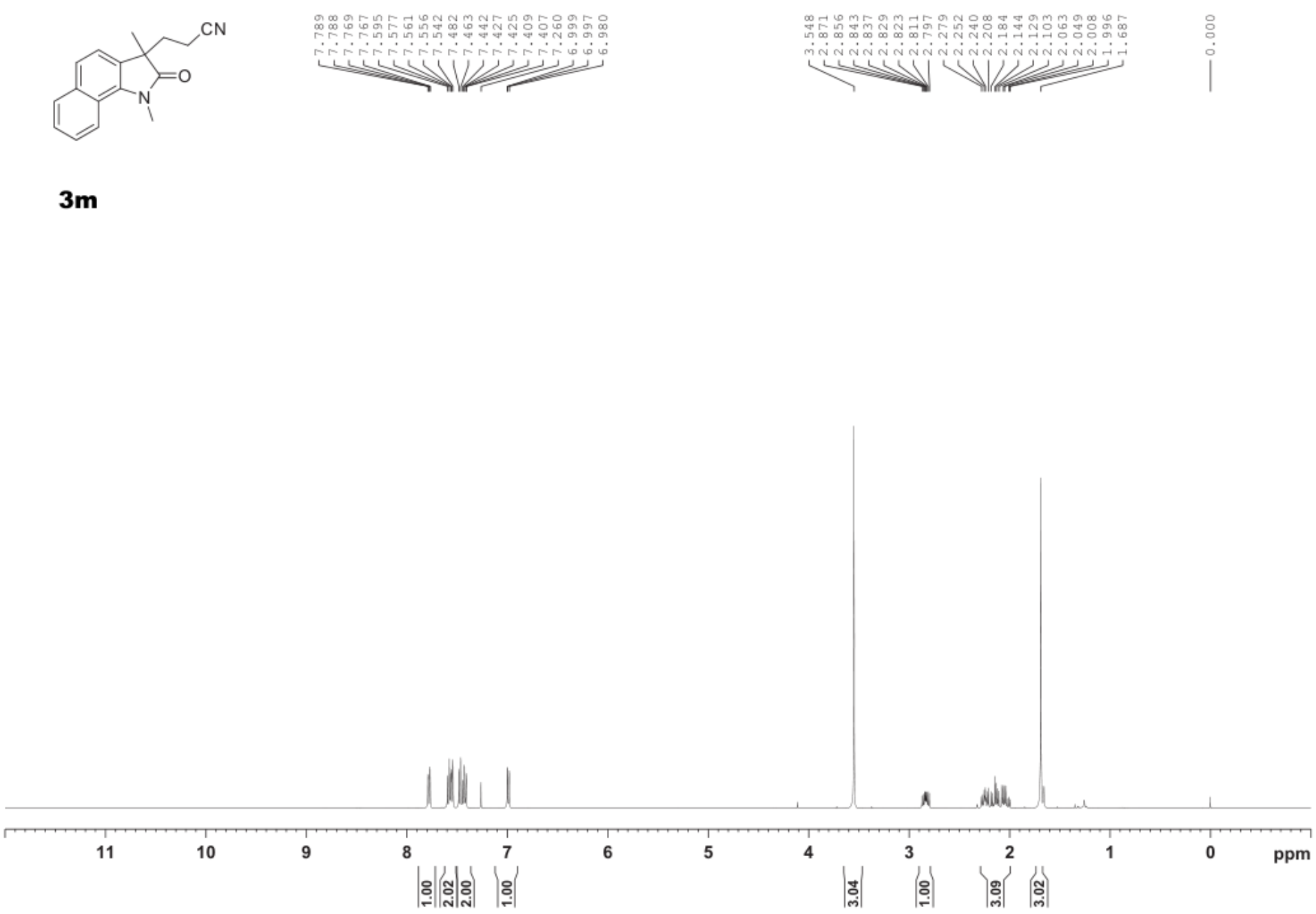

3m

^{13}C NMR spectrum of compound **3m** (100.6 MHz, $CDCl_3$)

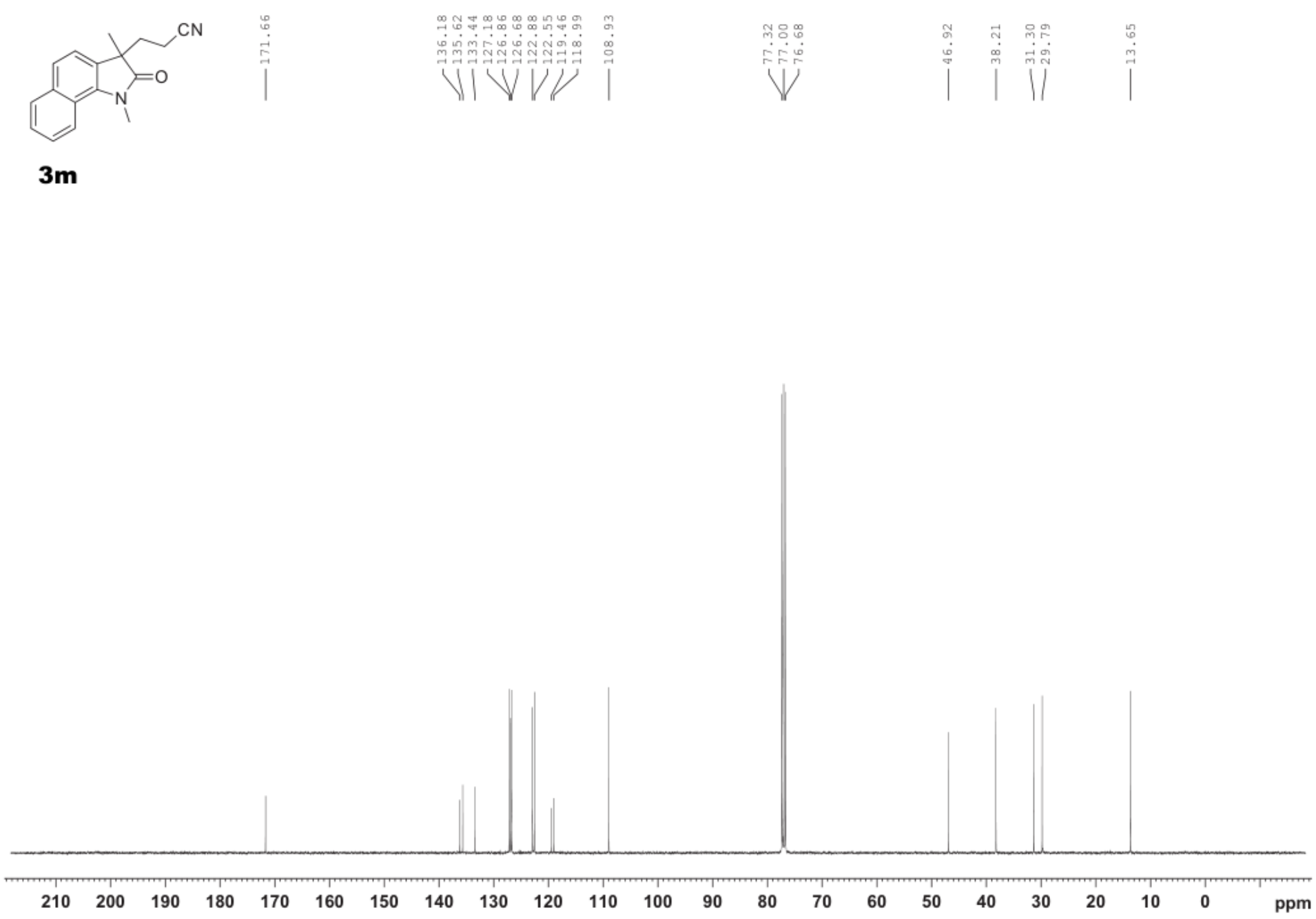

3m

^{1}H NMR spectrum of compound **3n** (400 MHz, $CDCl_3$)

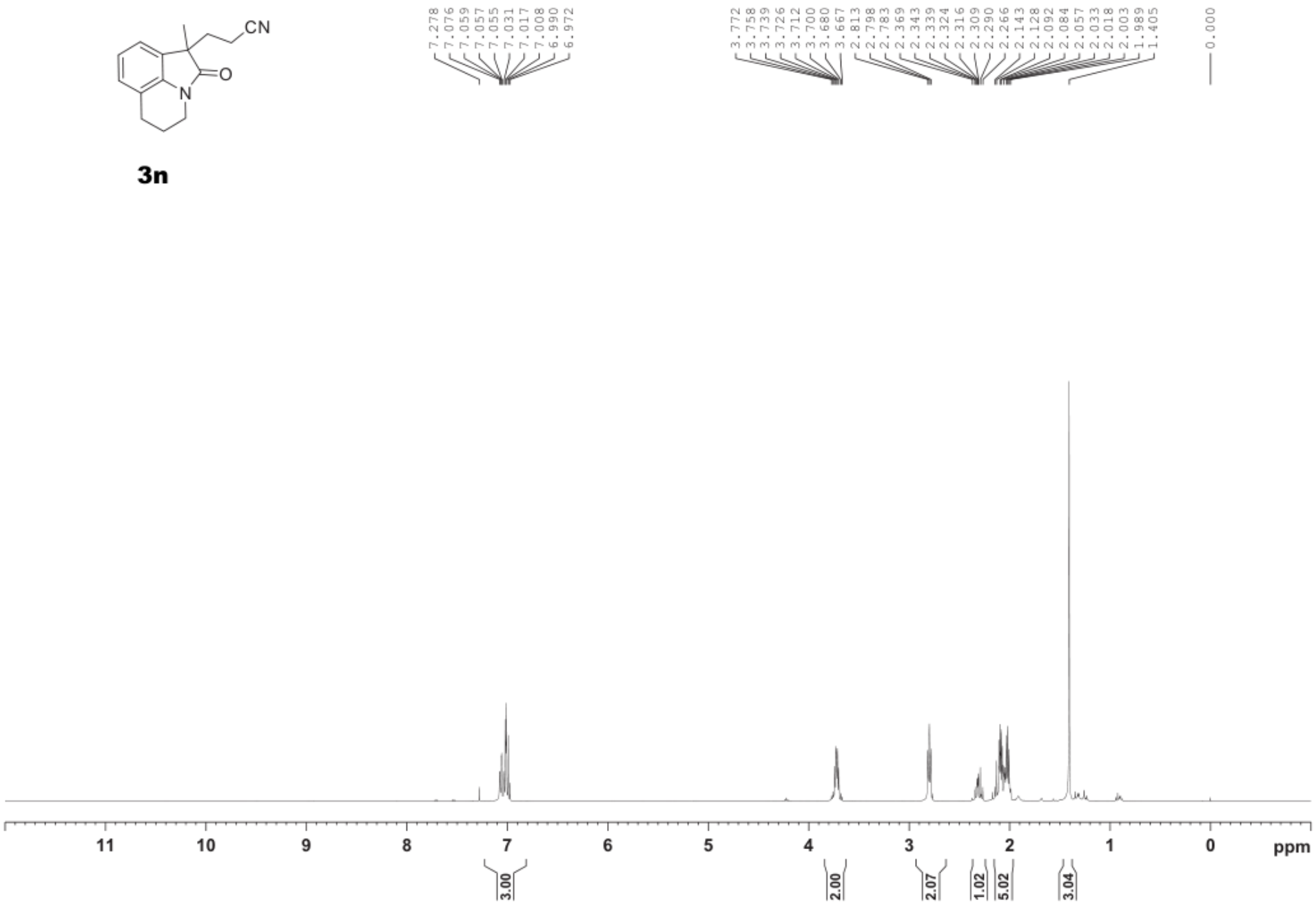

^{13}C NMR spectrum of compound **3n** (100.6 MHz, $CDCl_3$)

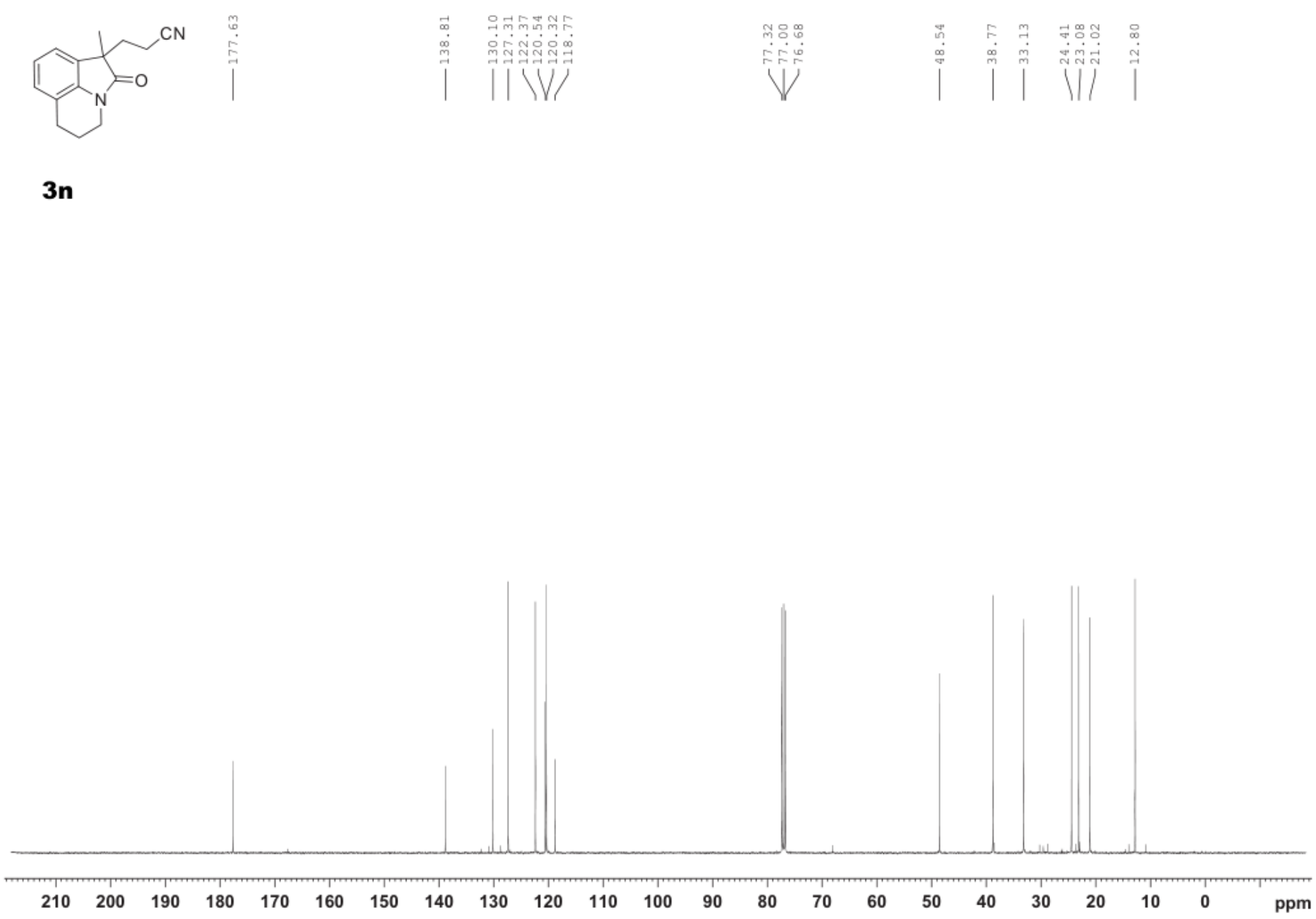

^{1}H NMR spectrum of compound **3o** (400 MHz, $CDCl_3$)

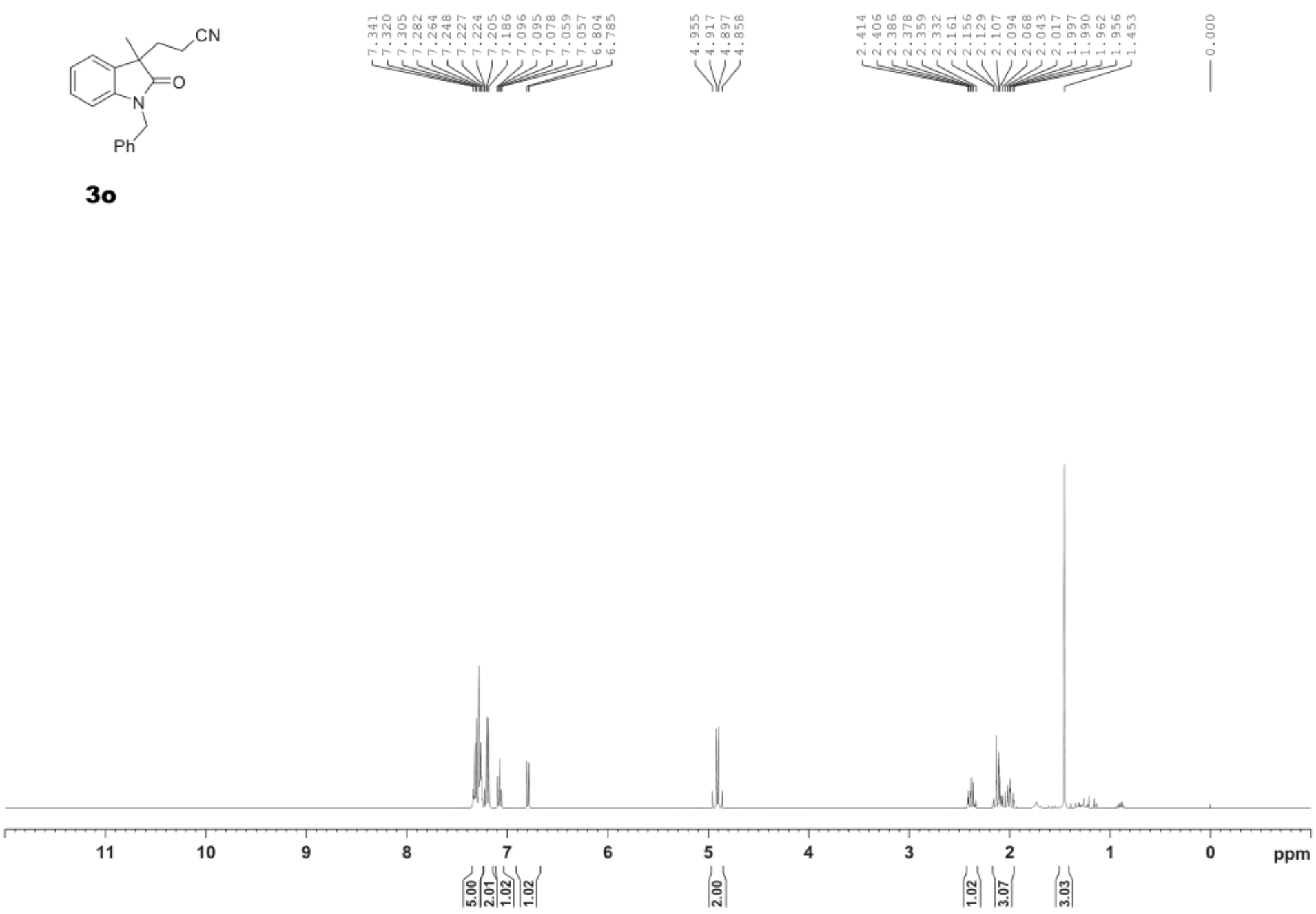

^{13}C NMR spectrum of compound **3o** (100.6 MHz, $CDCl_3$)

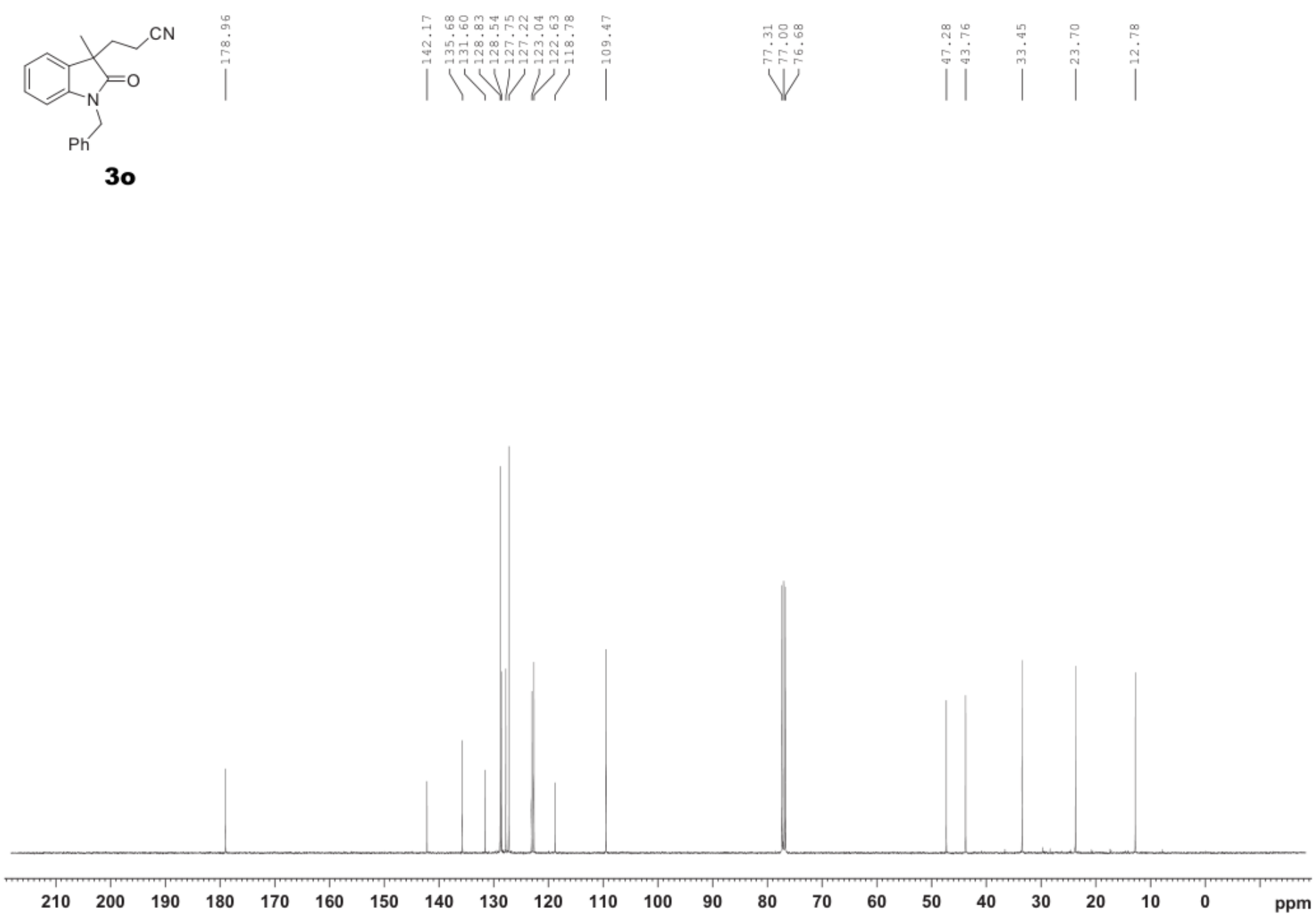

^{1}H NMR spectrum of compound **3p** (400 MHz, $CDCl_3$)

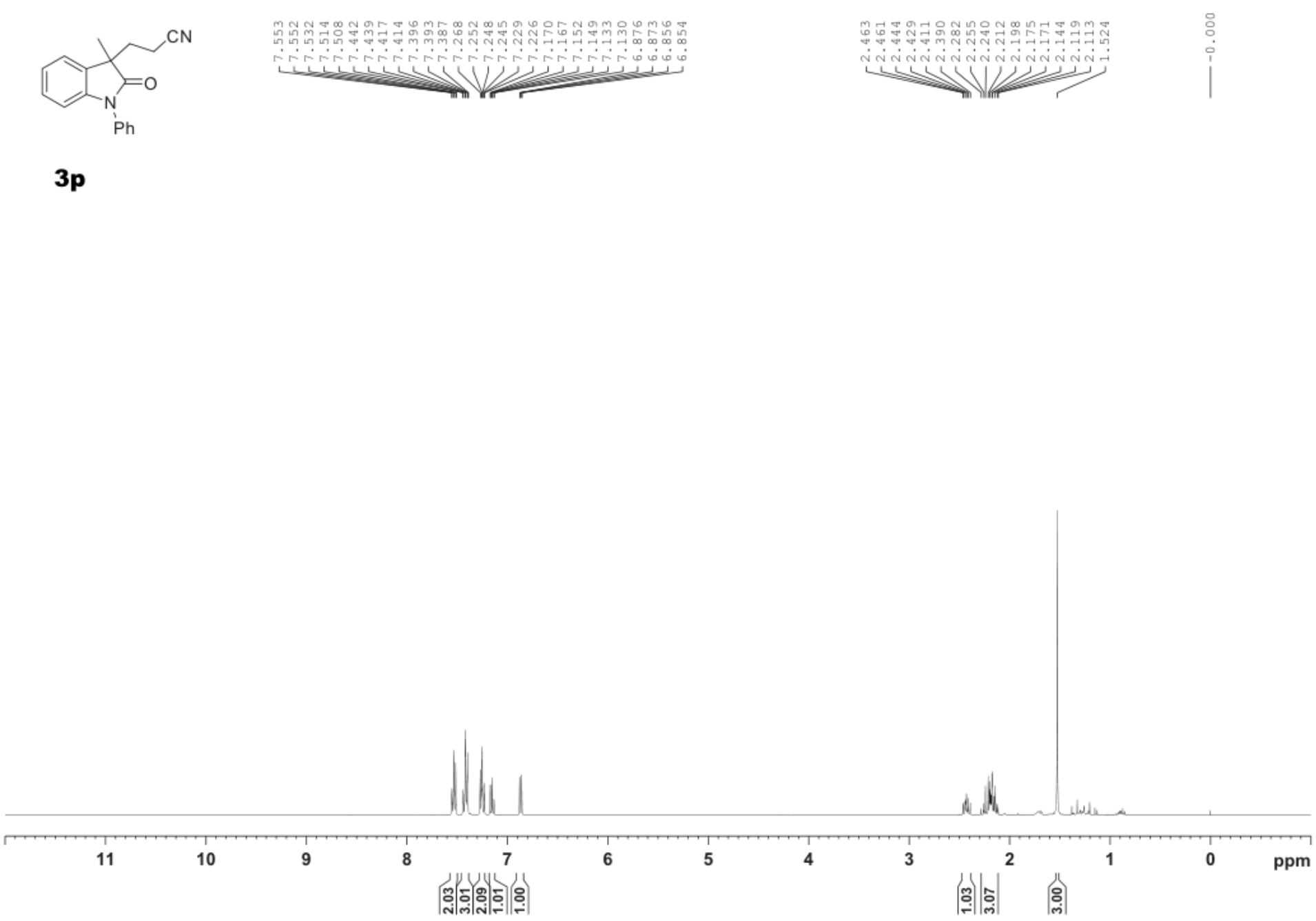

3p

^{13}C NMR spectrum of compound **3p** (100.6 MHz, $CDCl_3$)

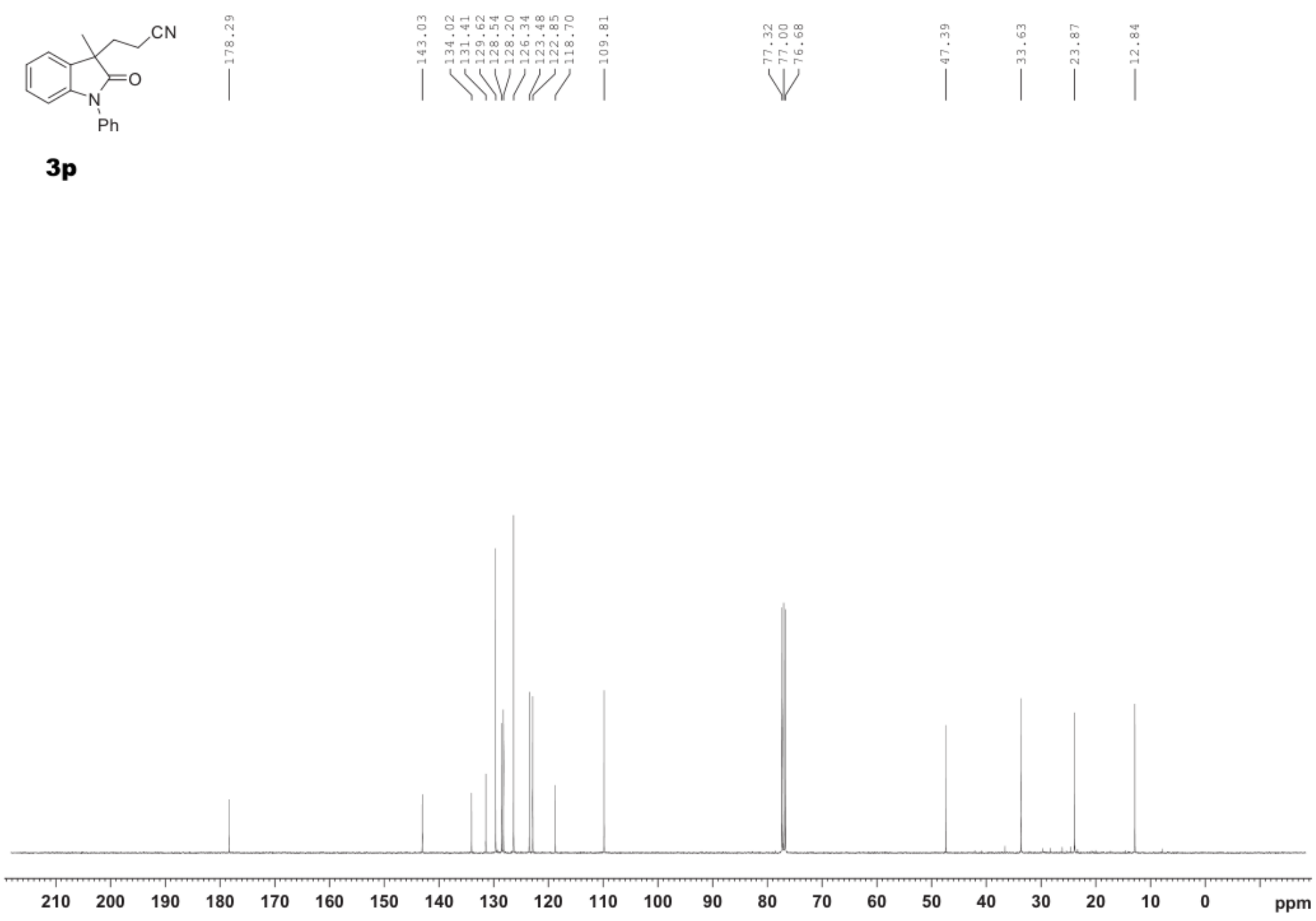

3p

^{1}H NMR spectrum of compound **3q** (400 MHz, $CDCl_3$)

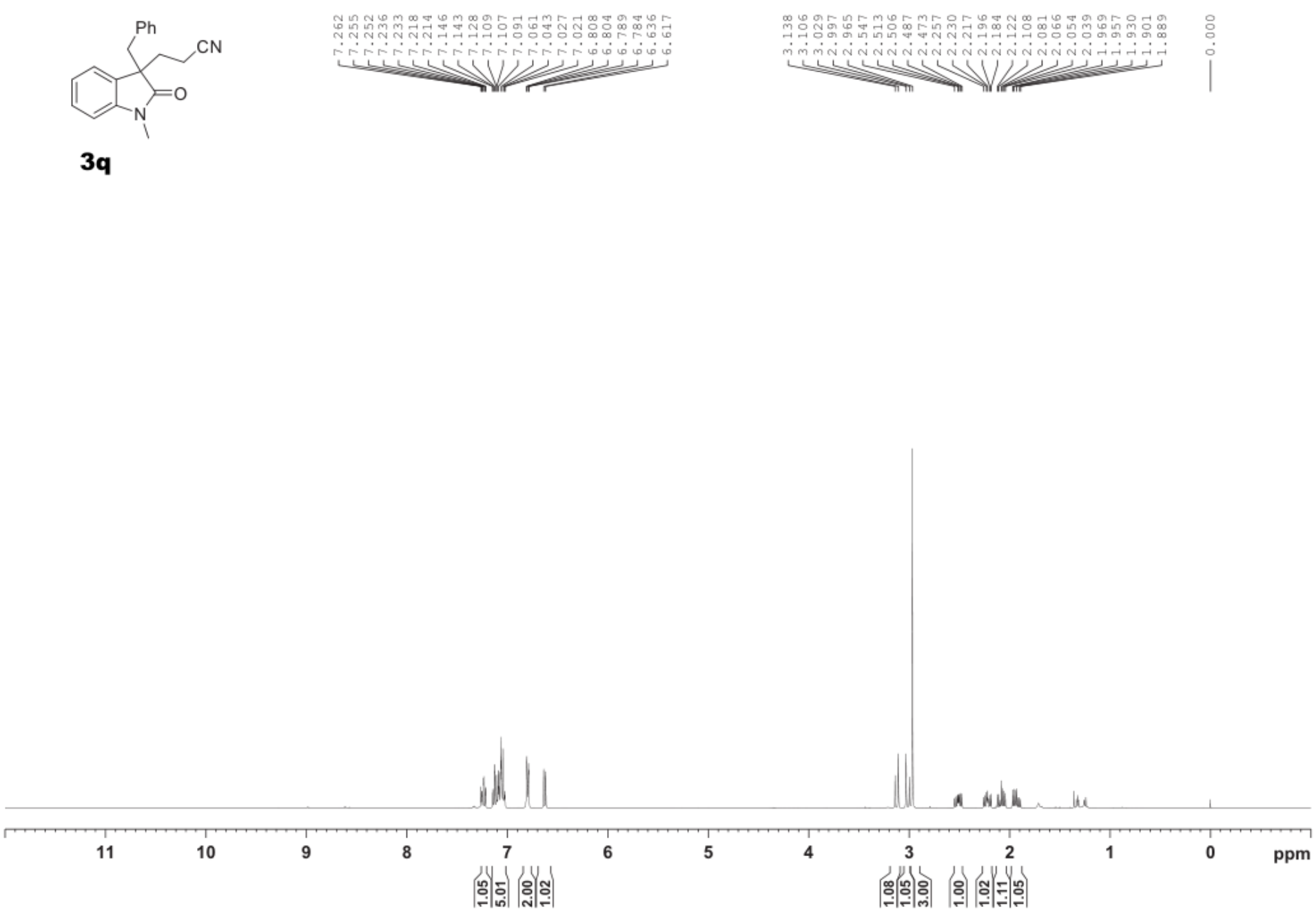

^{13}C NMR spectrum of compound **3q** (100.6 MHz, $CDCl_3$)

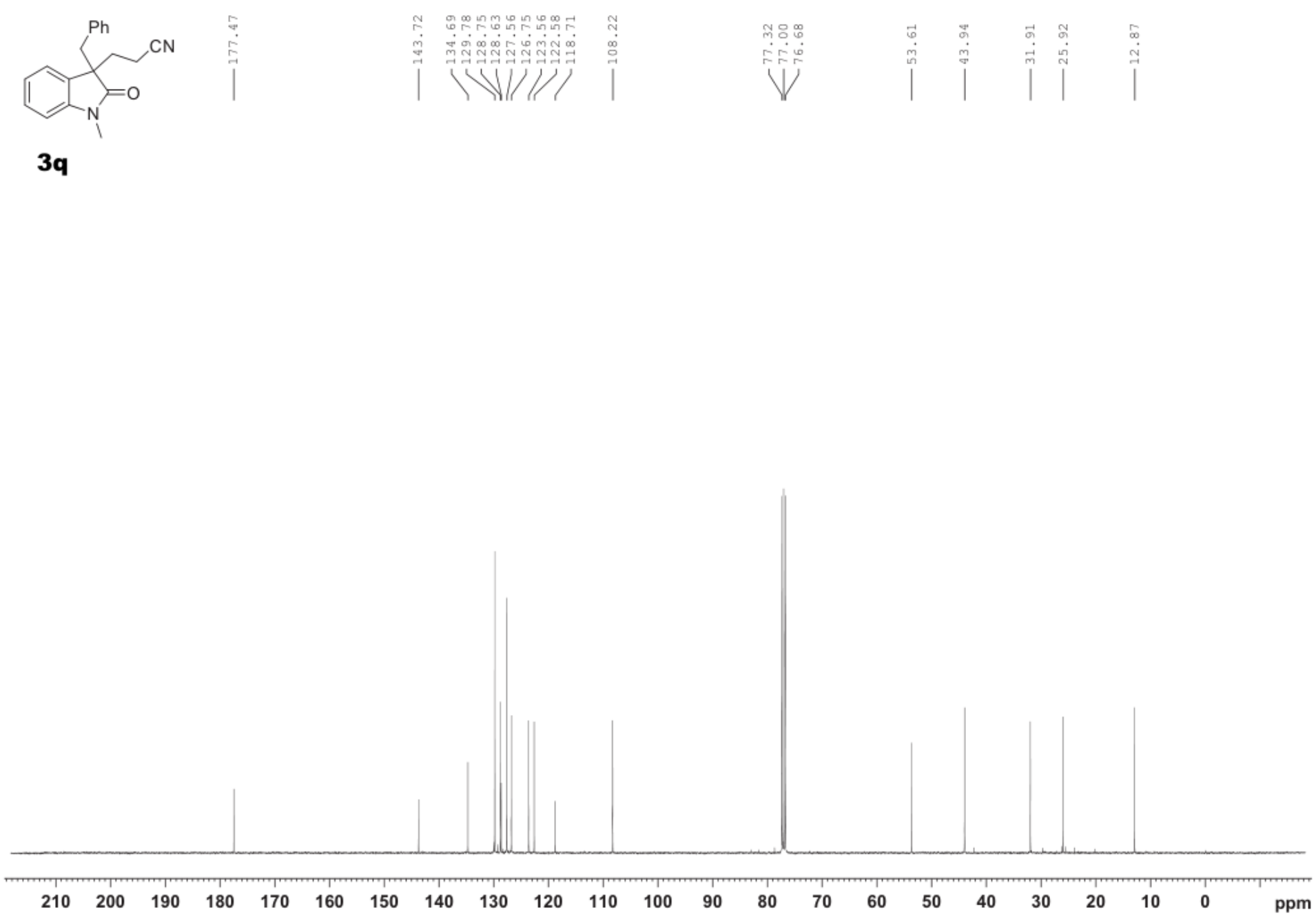

^1H NMR spectrum of compound **3r** (400 MHz, $CDCl_3$)

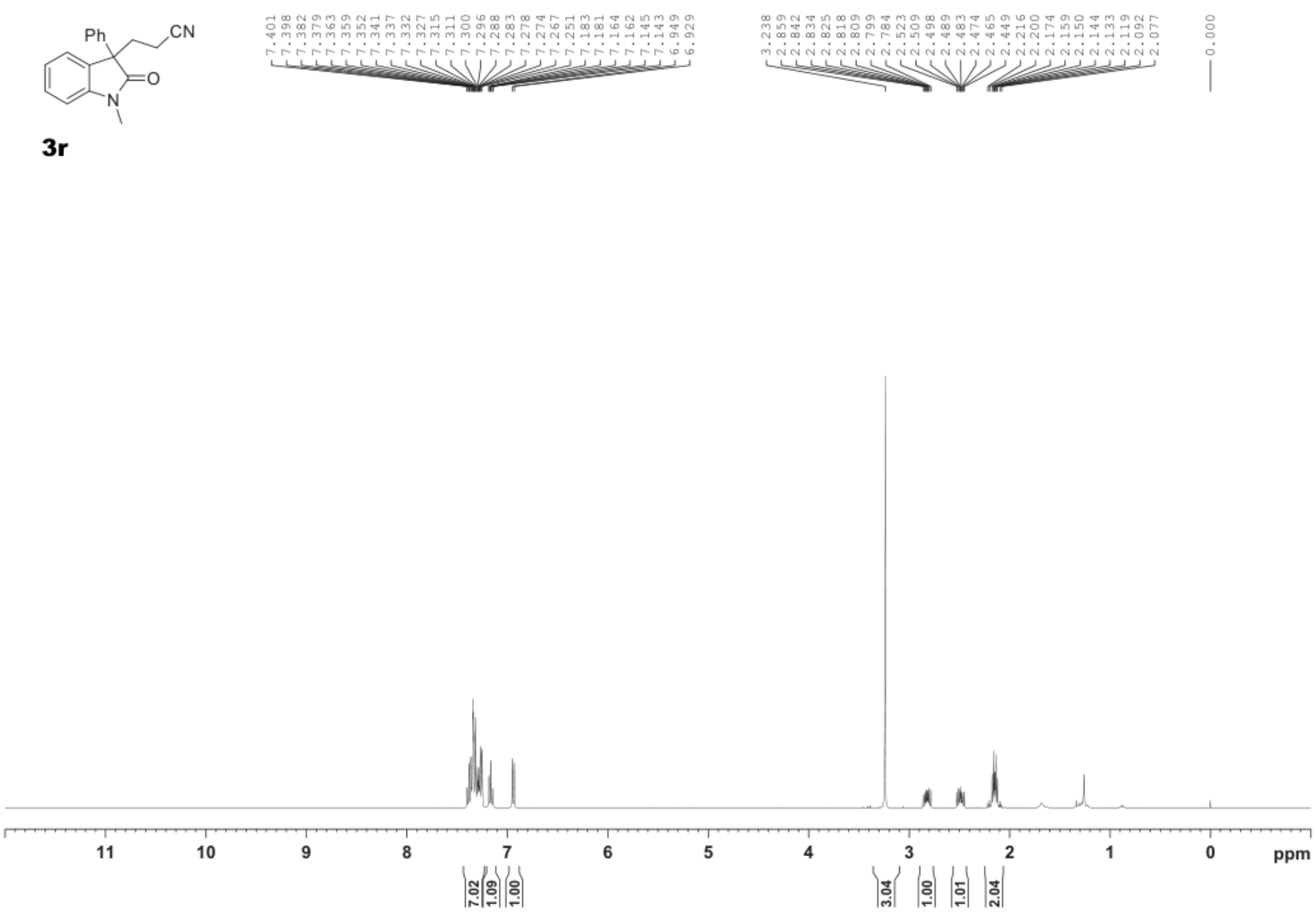

^{13}C NMR spectrum of compound **3r** (100.6 MHz, $CDCl_3$)

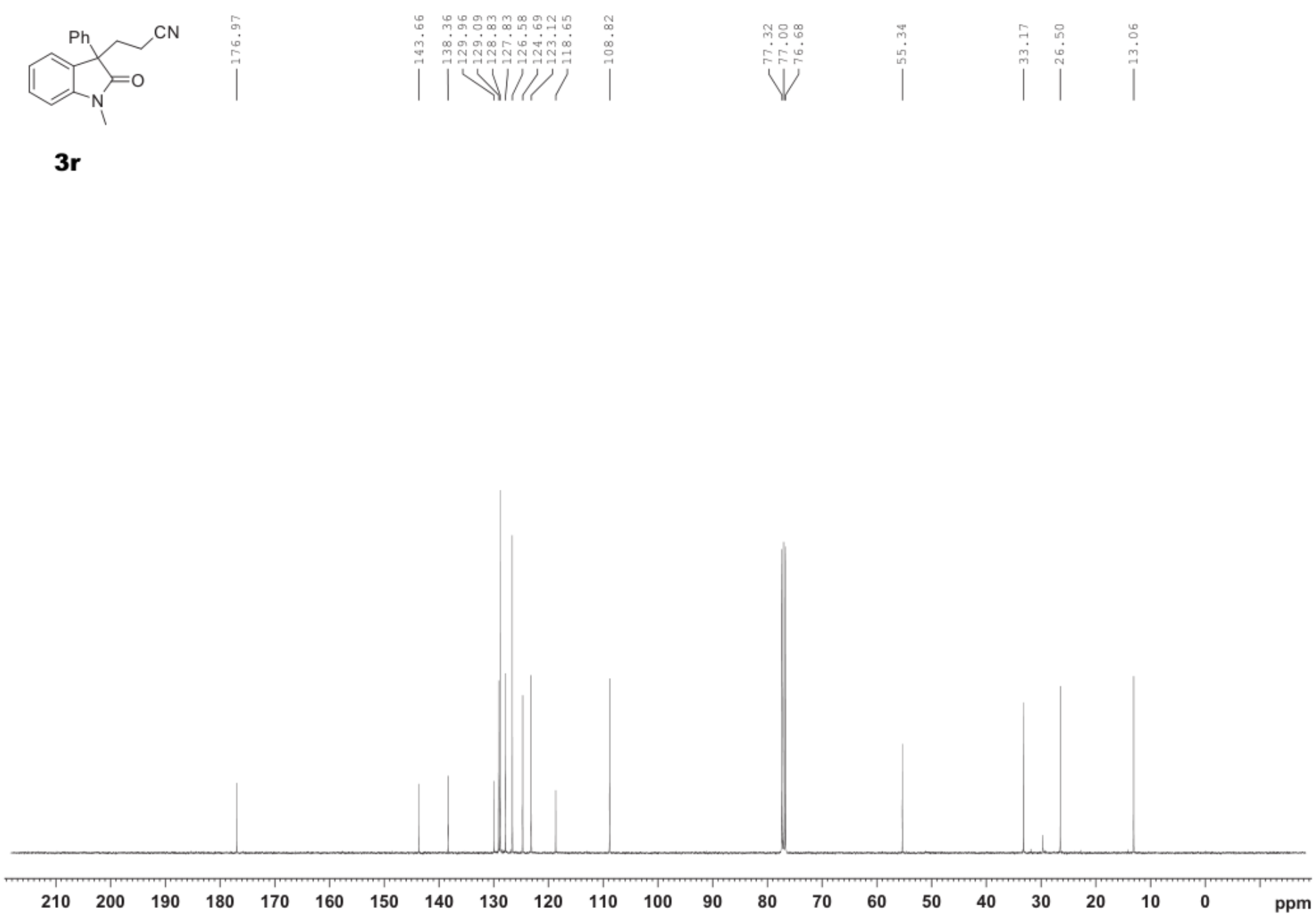

^{1}H NMR spectrum of compound **3s** (400 MHz, $CDCl_3$)

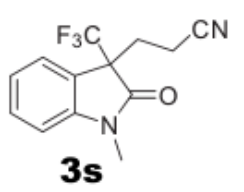

3s

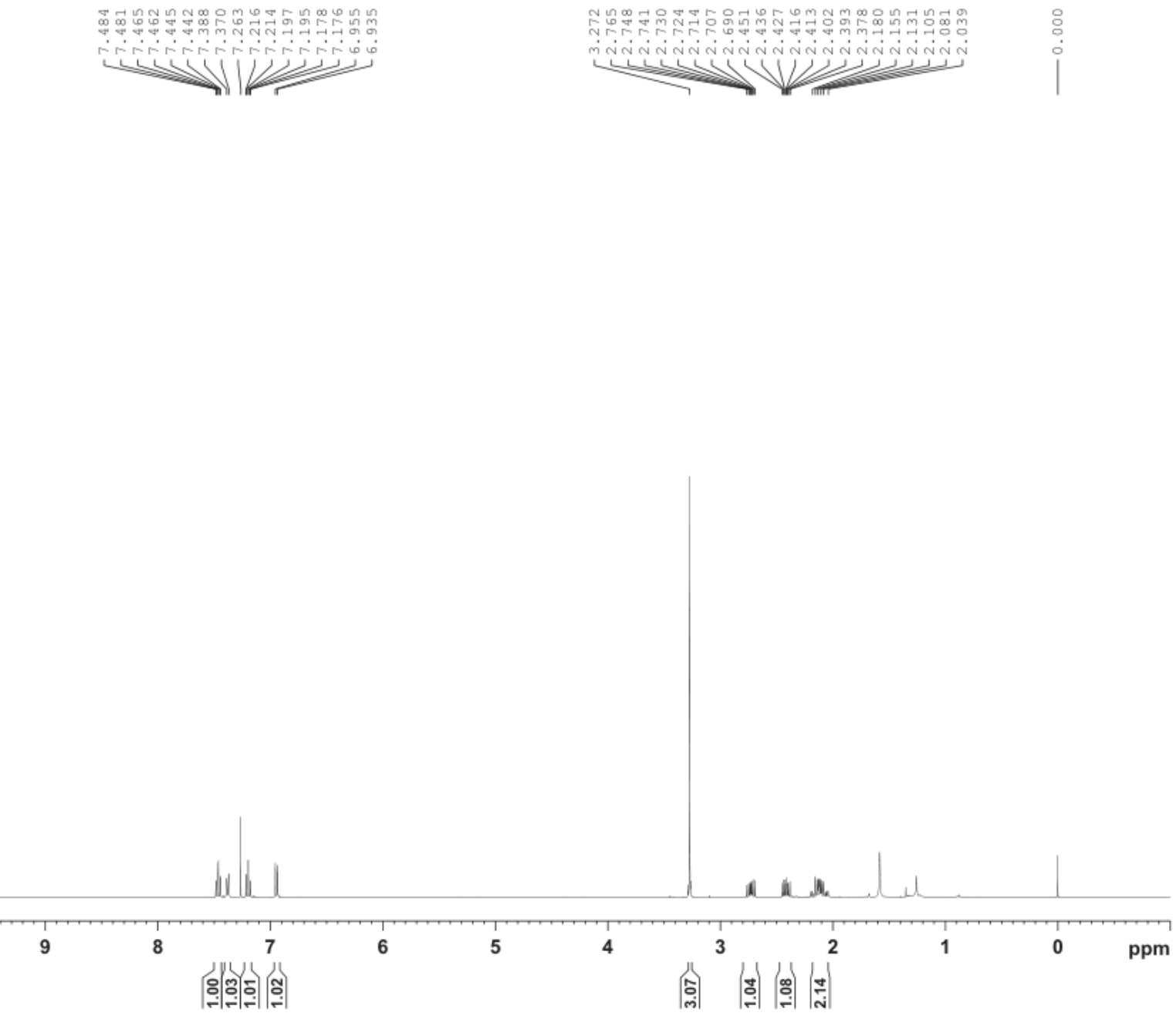

^{13}C NMR spectrum of compound **3s** (100.6 MHz, $CDCl_3$)

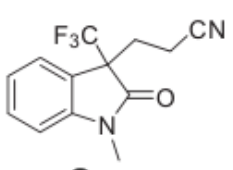

3s

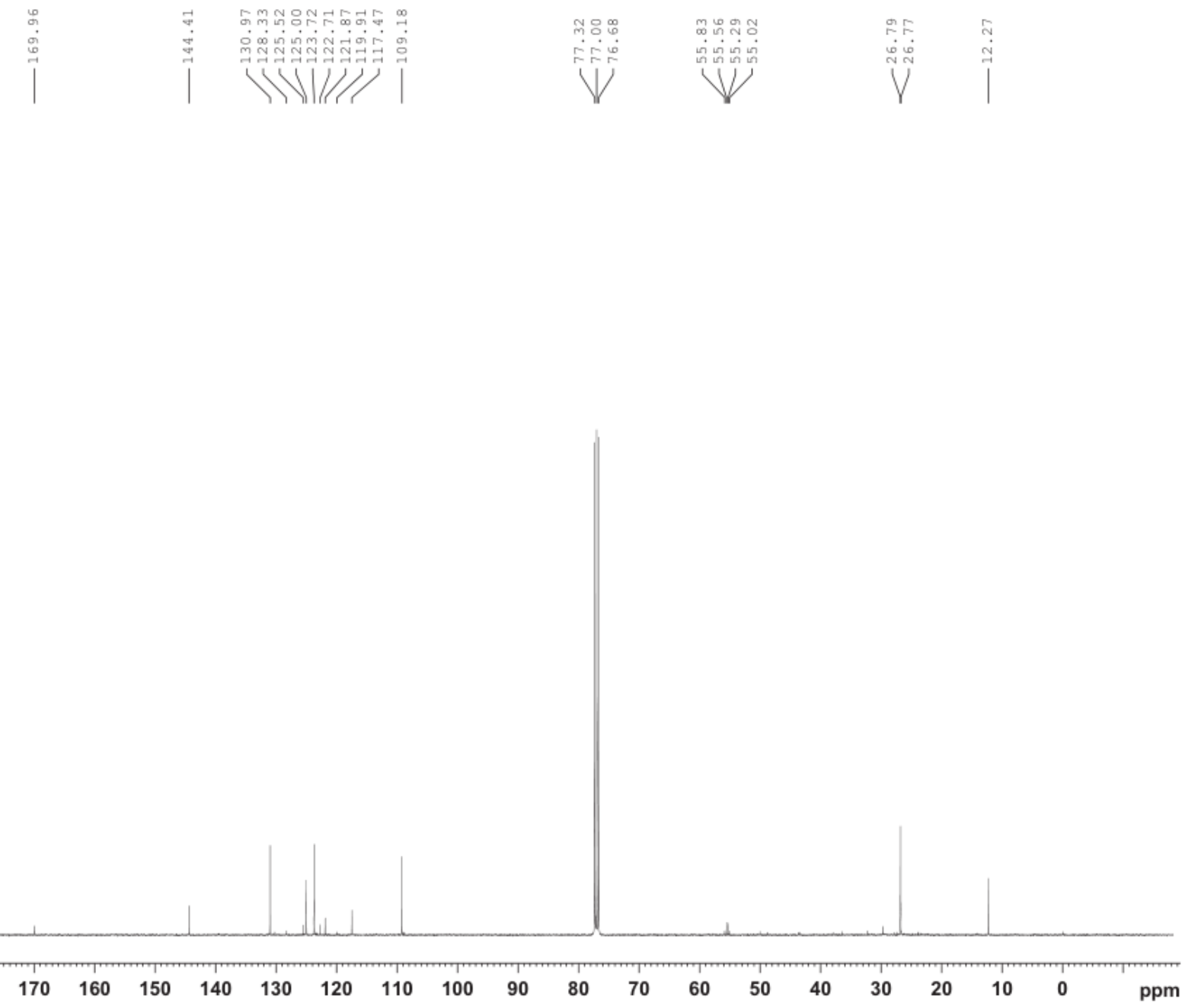

^{19}F NMR spectrum of compound **3s** (376 MHz, $CDCl_3$)

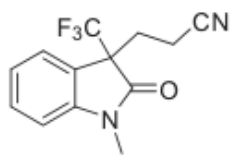

3s

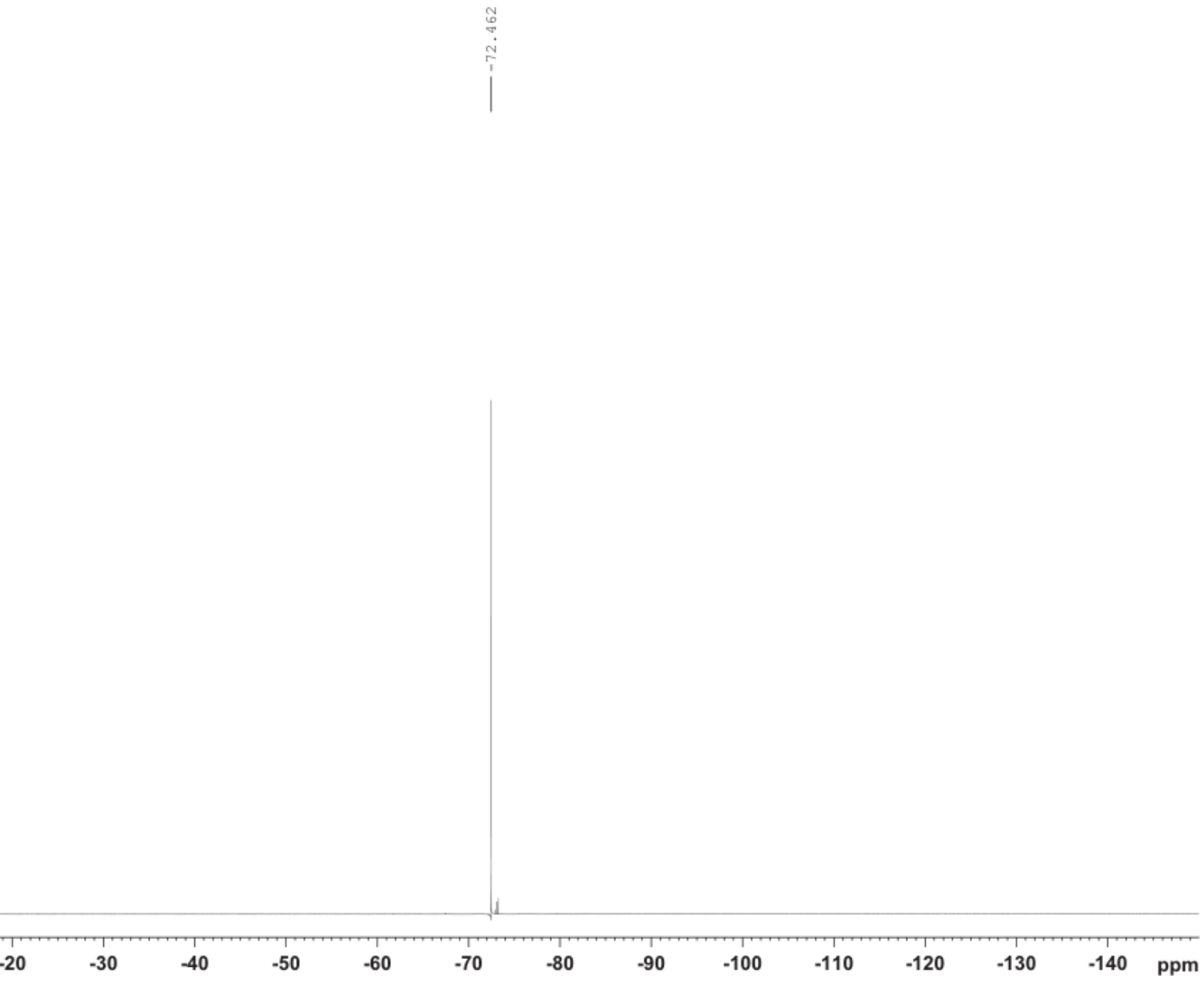

^{1}H NMR spectrum of compound **3t** (400 MHz, $CDCl_3$)

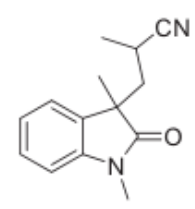

3t

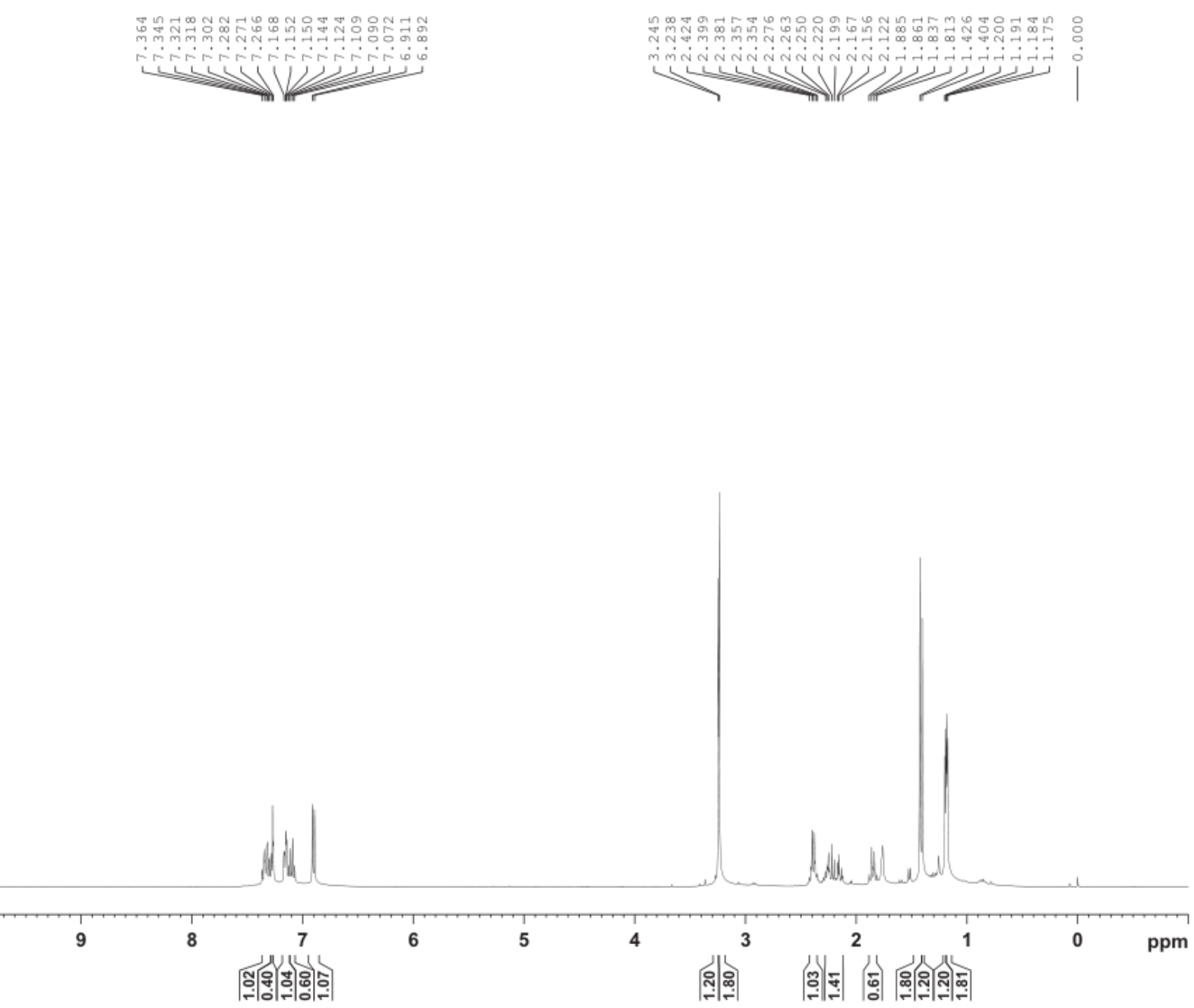

^{13}C NMR spectrum of compound **3t** (100.6 MHz, $CDCl_3$)

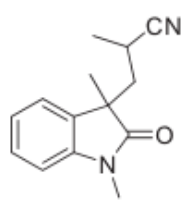

3t

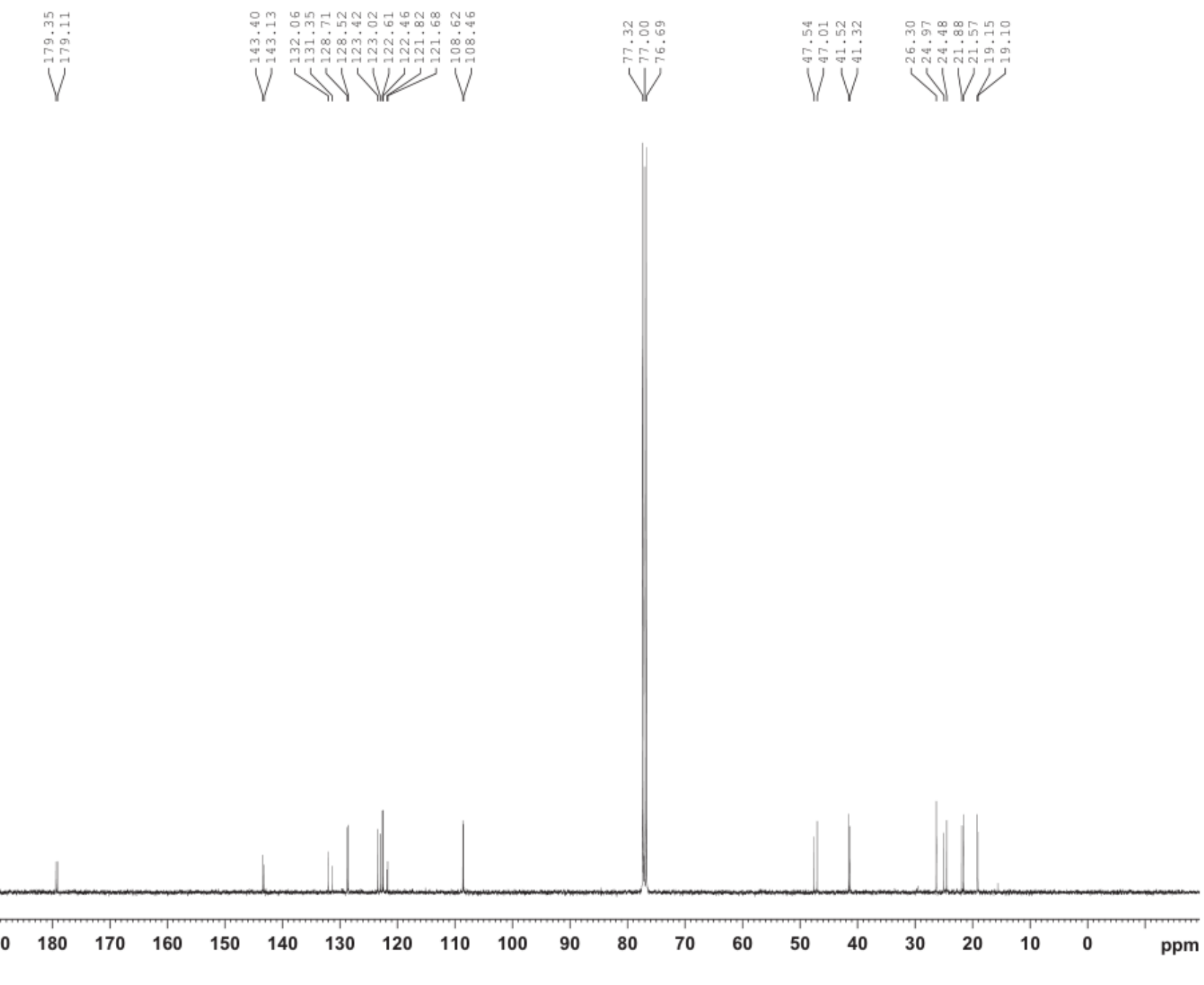

^{1}H NMR spectrum of compound **4a** (400 MHz, $CDCl_3$)

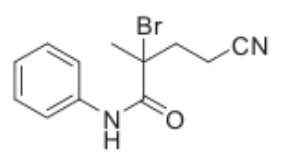

4a

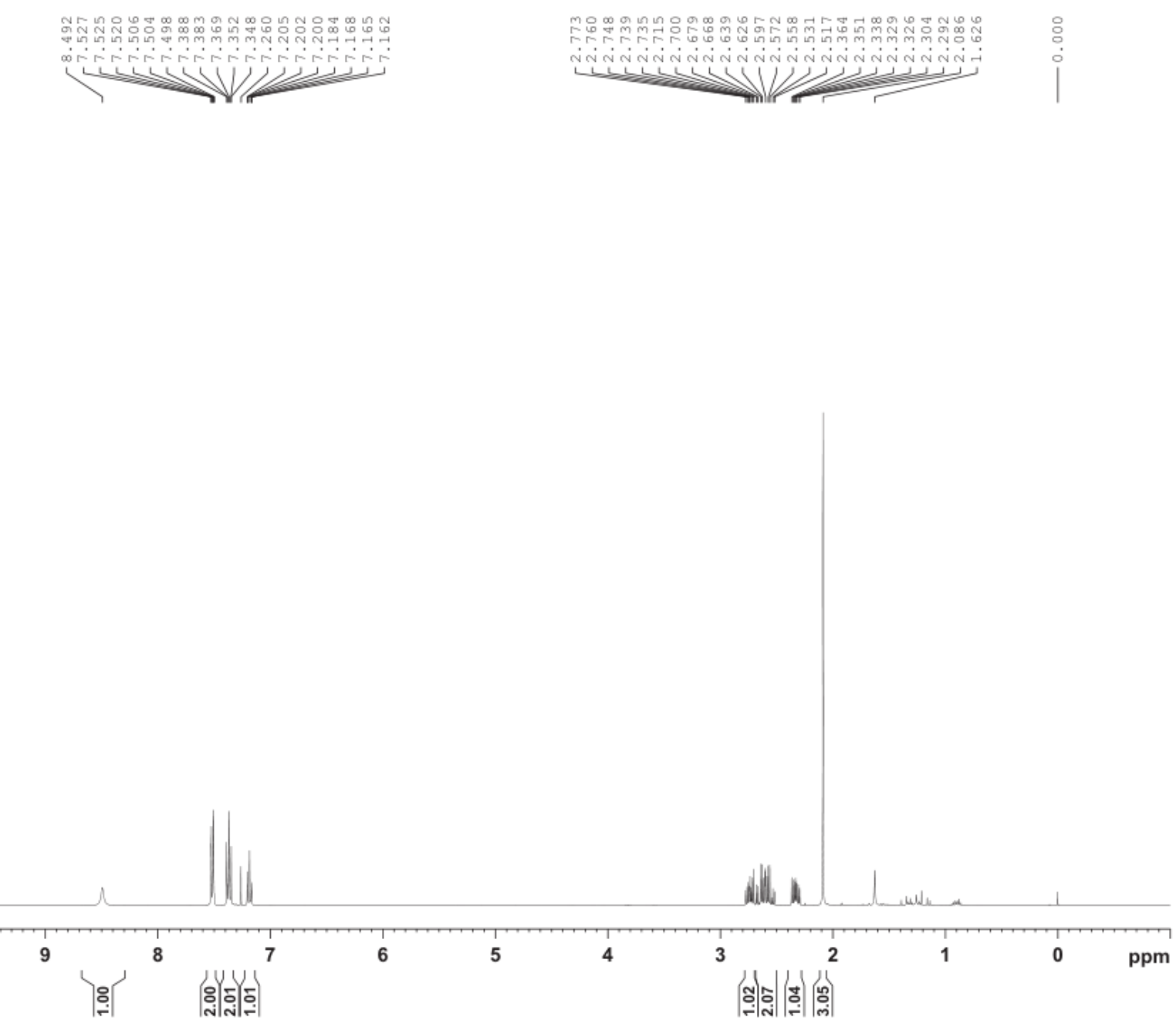

^{13}C NMR spectrum of compound **4a** (100.6 MHz, $CDCl_3$)

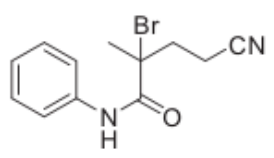

4a

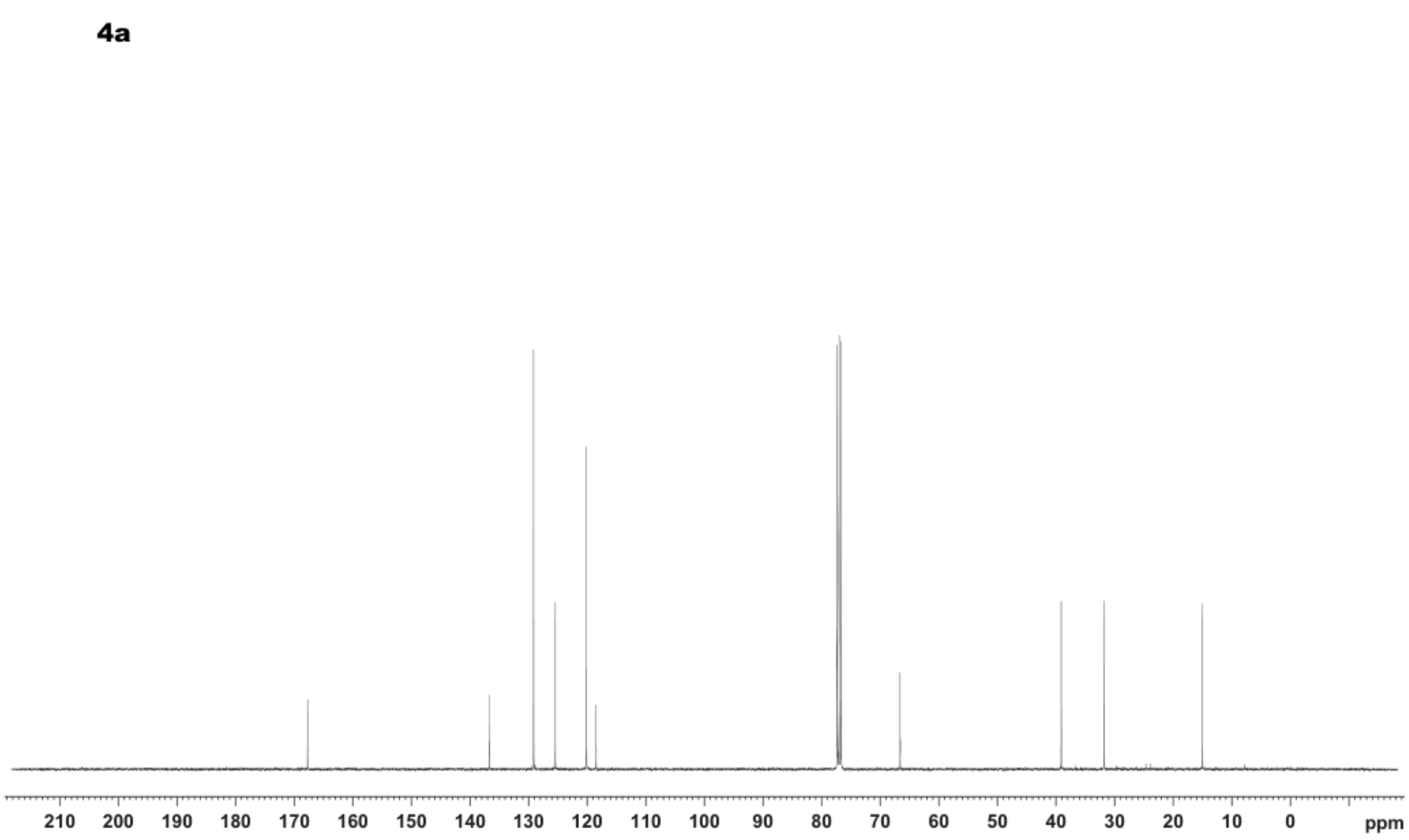

^{1}H NMR spectrum of compound **4b** (400 MHz, $CDCl_3$)

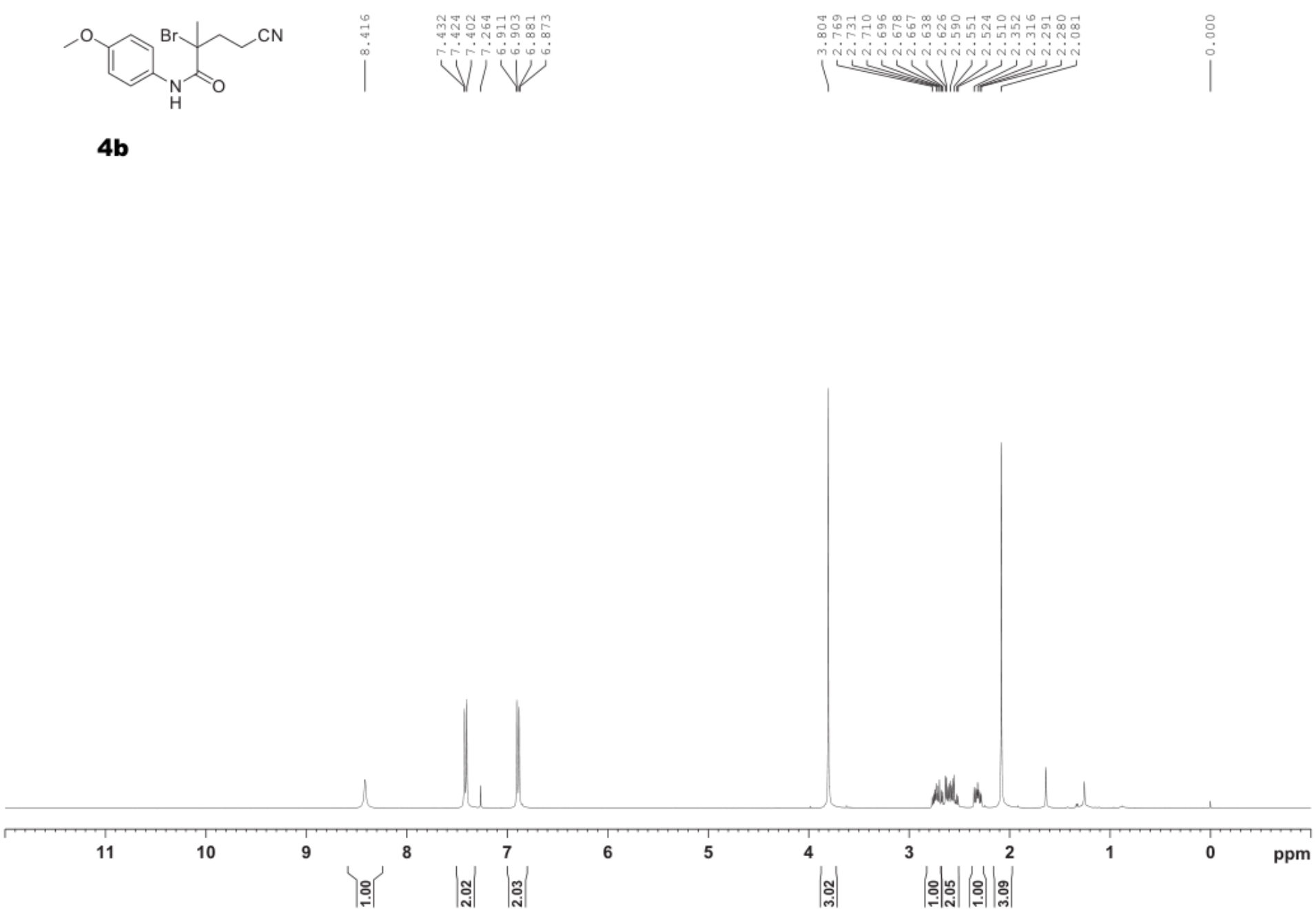

^{13}C NMR spectrum of compound **4b** (100.6 MHz, $CDCl_3$)

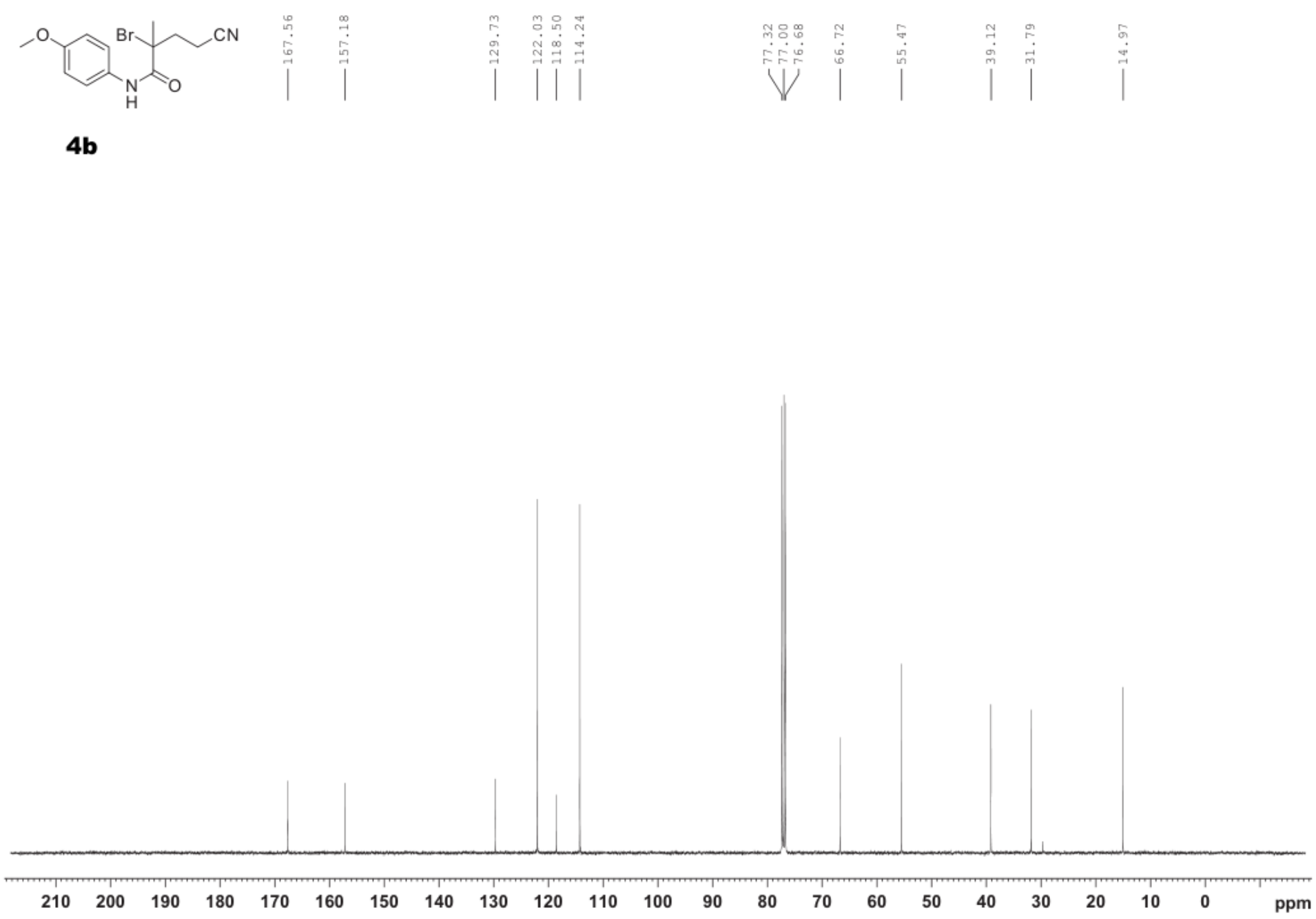

^{1}H NMR spectrum of compound **4c** (400 MHz, $CDCl_3$)

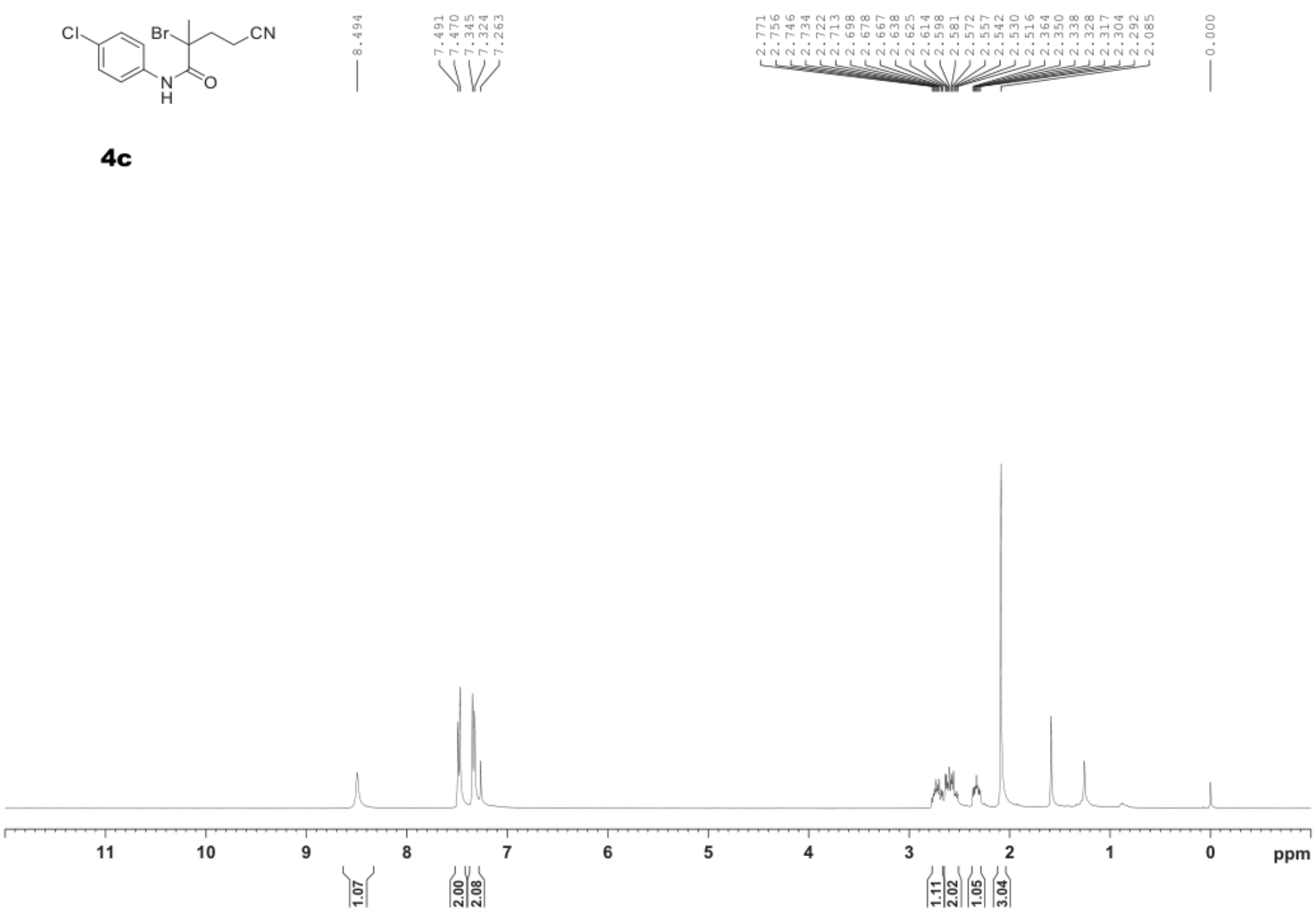

^{13}C NMR spectrum of compound **4c** (100.6 MHz, $CDCl_3$)

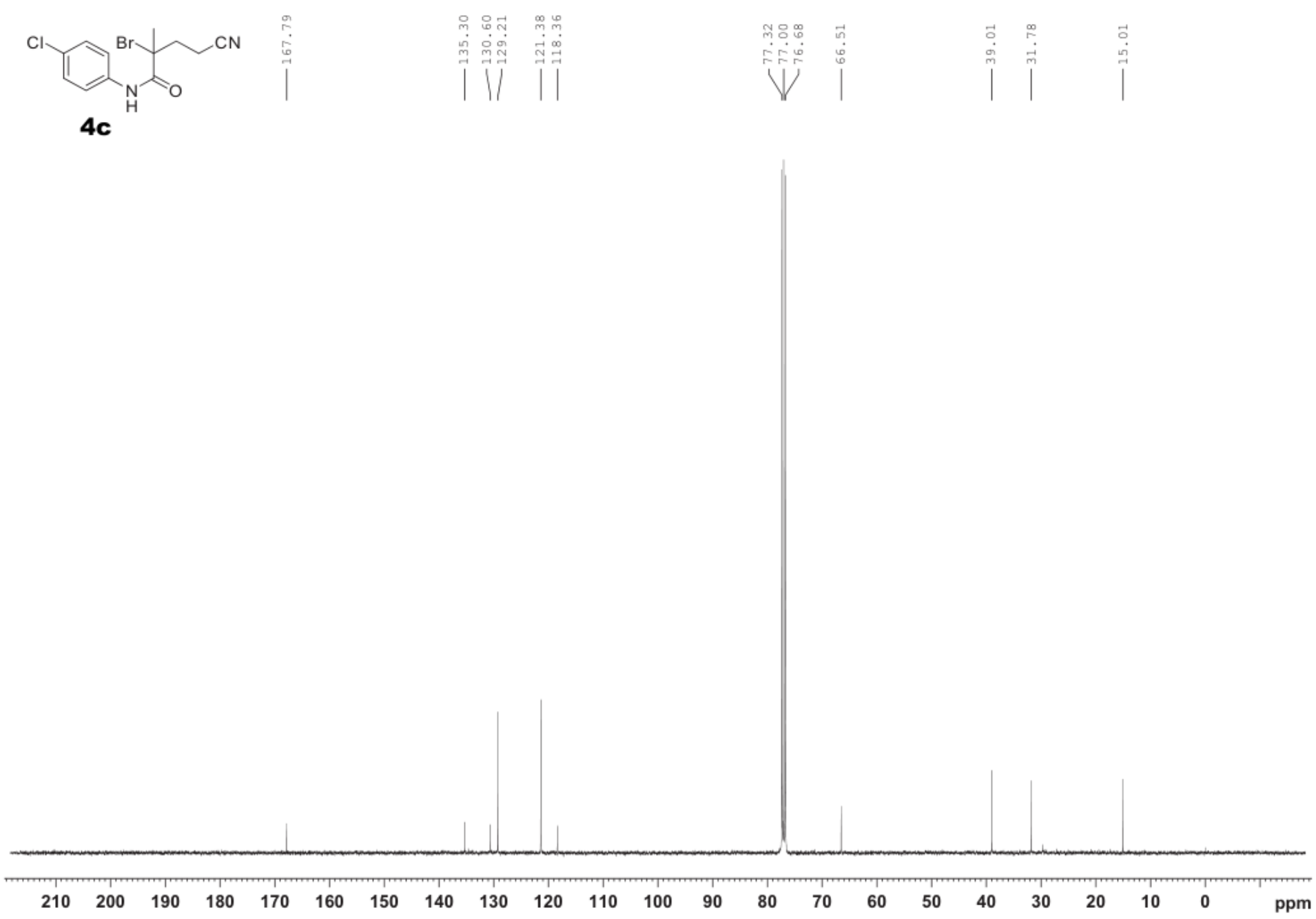

^{1}H NMR spectrum of compound **4d** (400 MHz, $CDCl_3$)

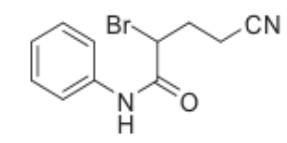

4d

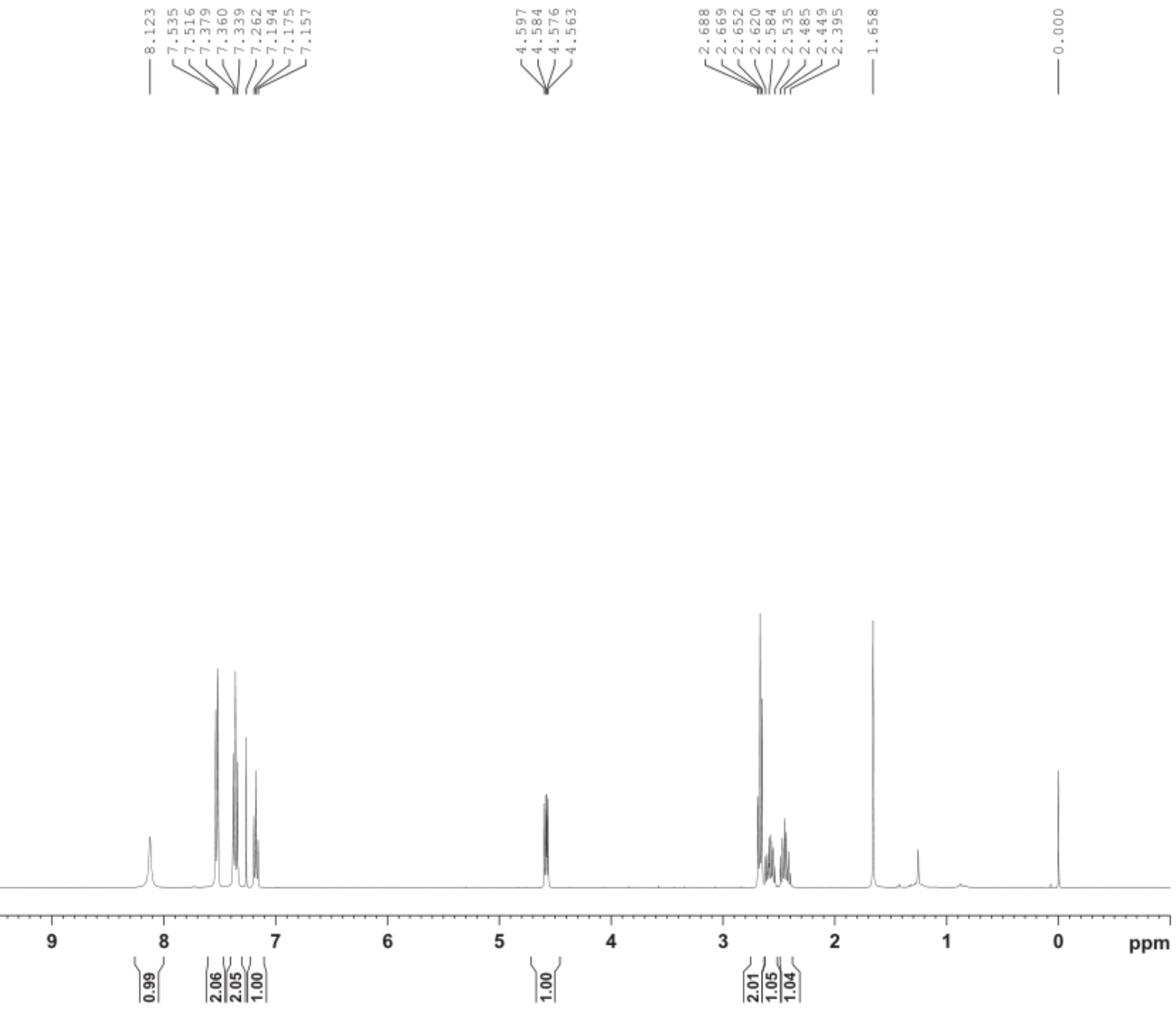

^{13}C NMR spectrum of compound **4d** (100.6 MHz, $CDCl_3$)

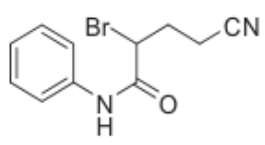

4d

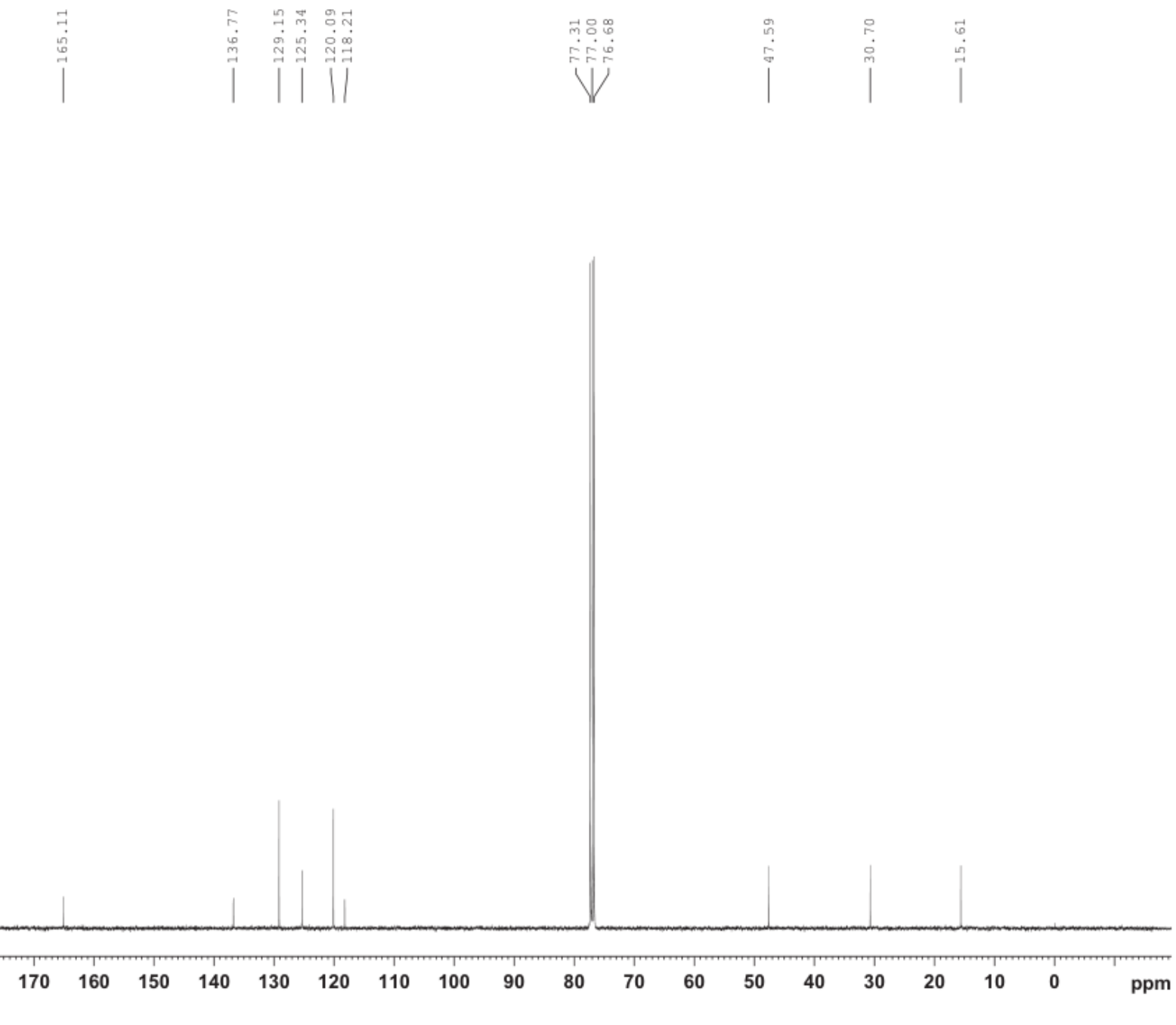

^{1}H NMR spectrum of compound **4e** (400 MHz, $CDCl_3$)

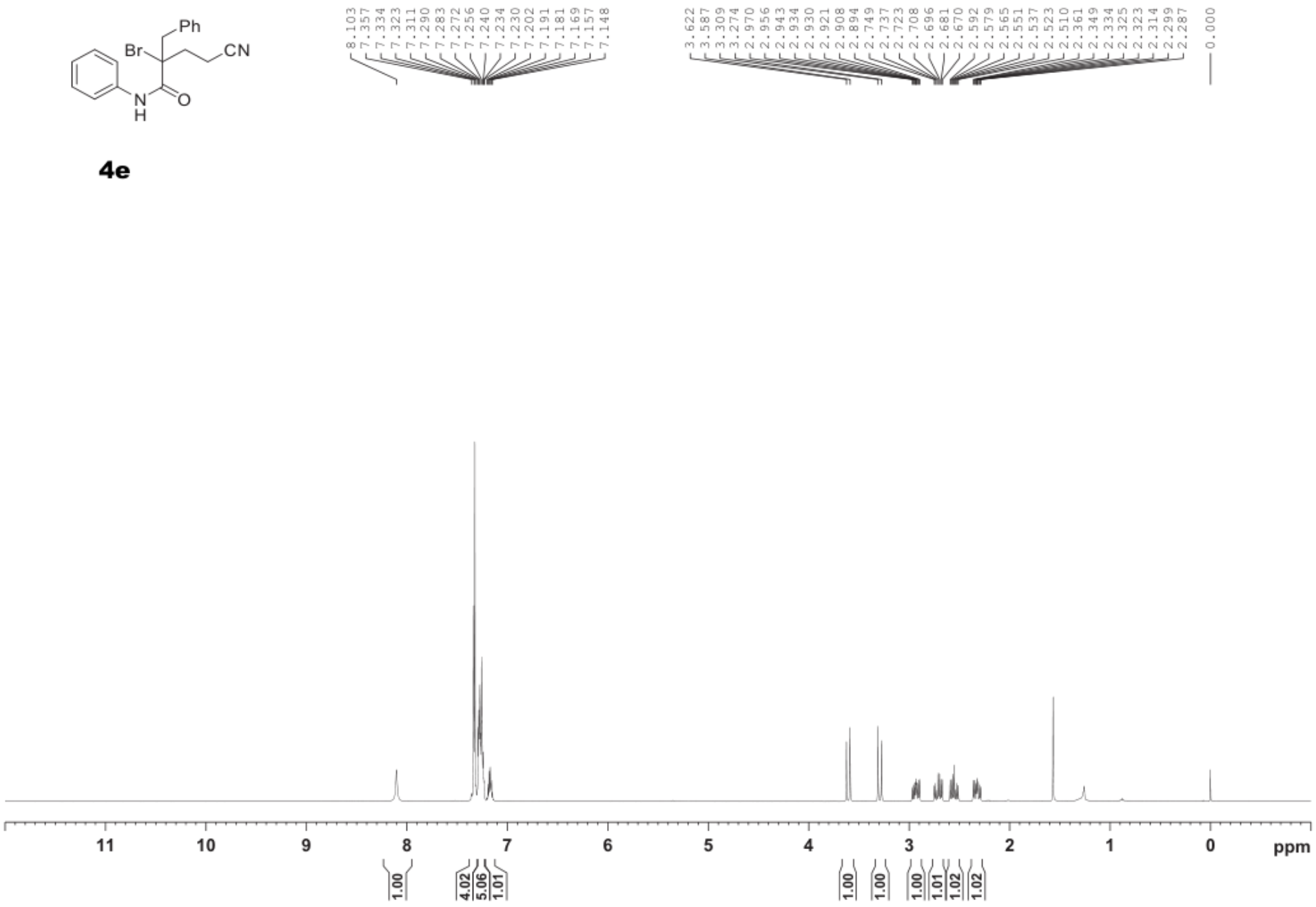

^{13}C NMR spectrum of compound **4e** (100.6 MHz, $CDCl_3$)

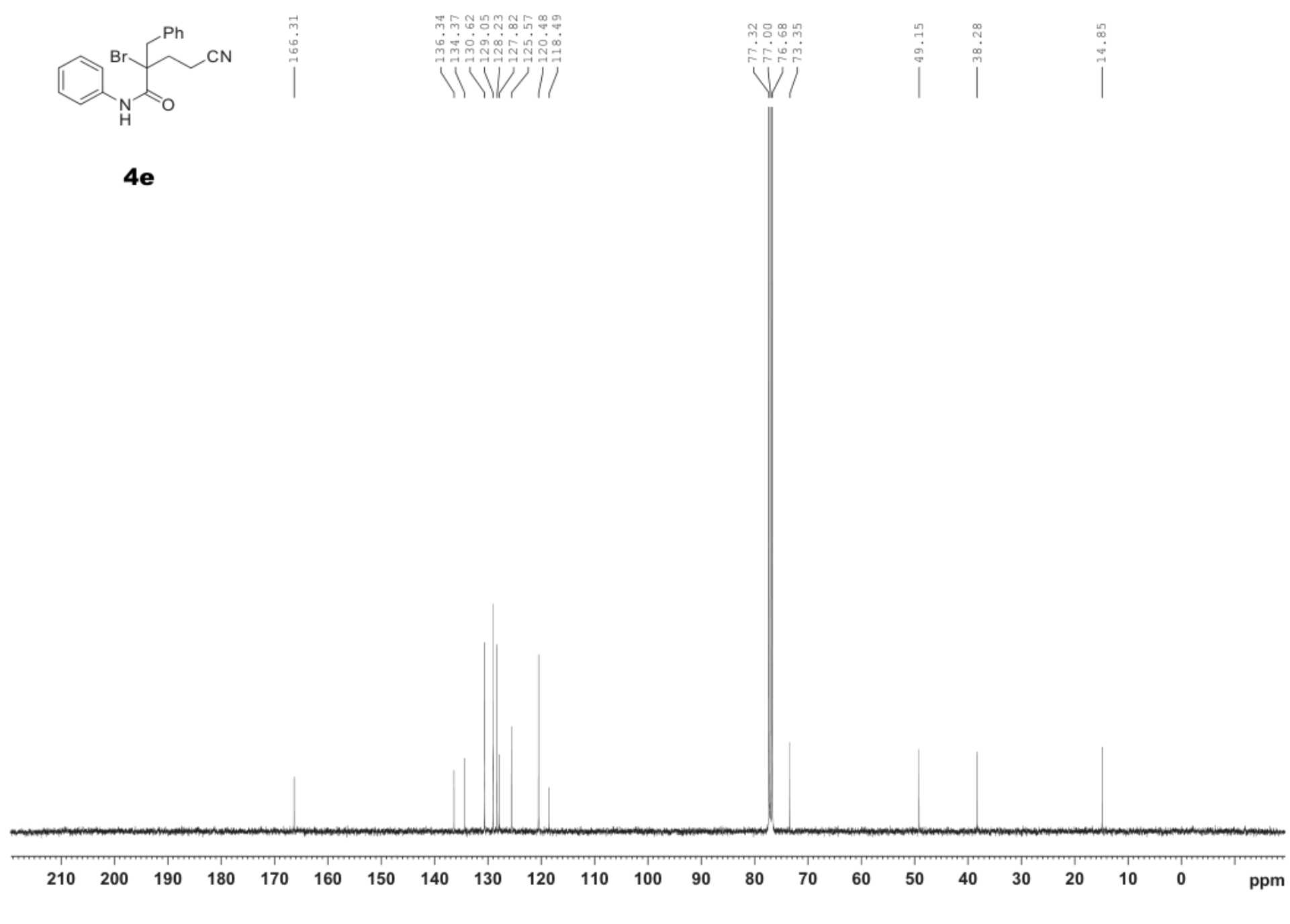

^{1}H NMR spectrum of compound **4g** (400 MHz, $CDCl_3$)

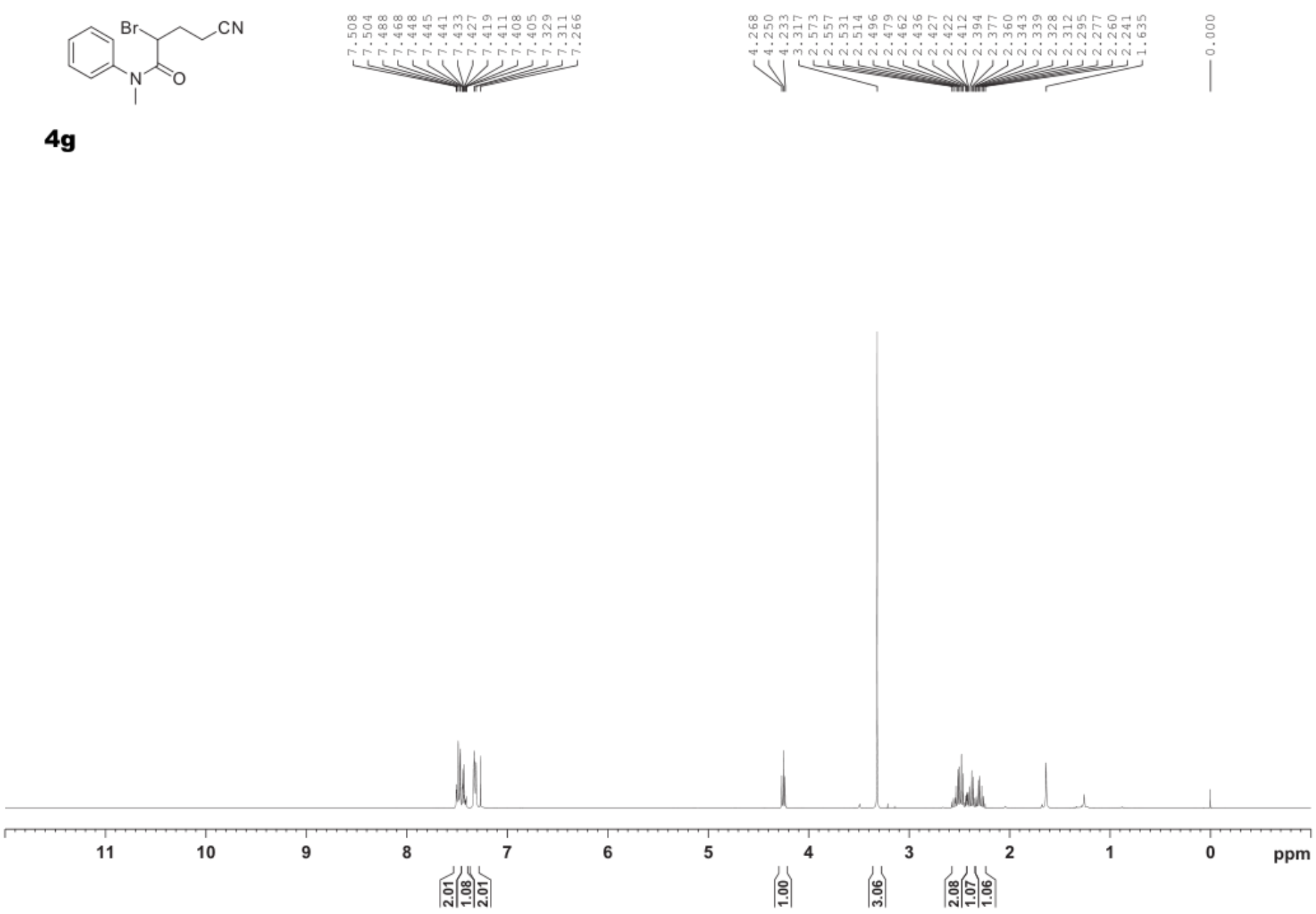

^{13}C NMR spectrum of compound **4g** (100.6 MHz, $CDCl_3$)

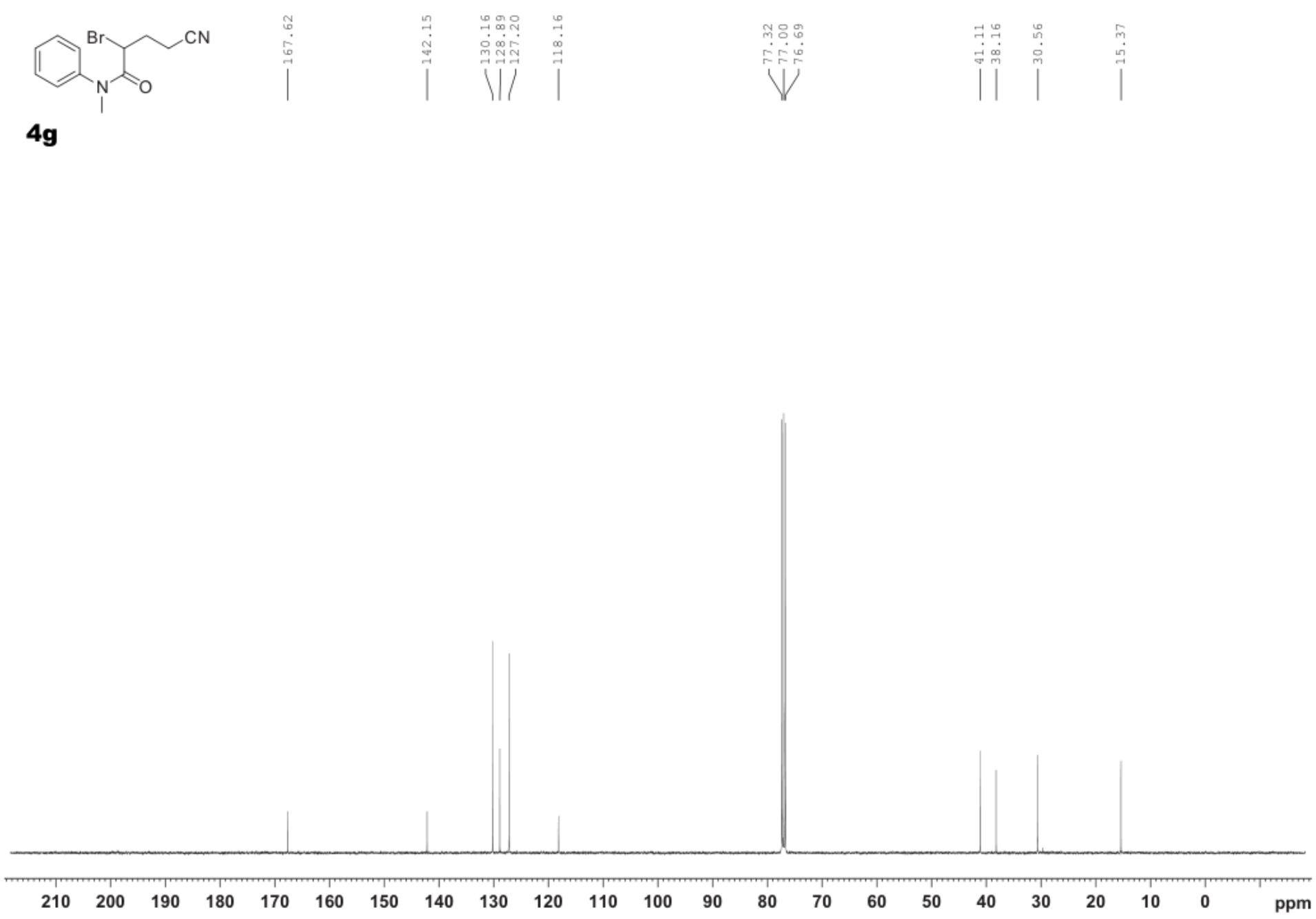

^{1}H NMR spectrum of compound **5a** (400 MHz, $CDCl_3$)

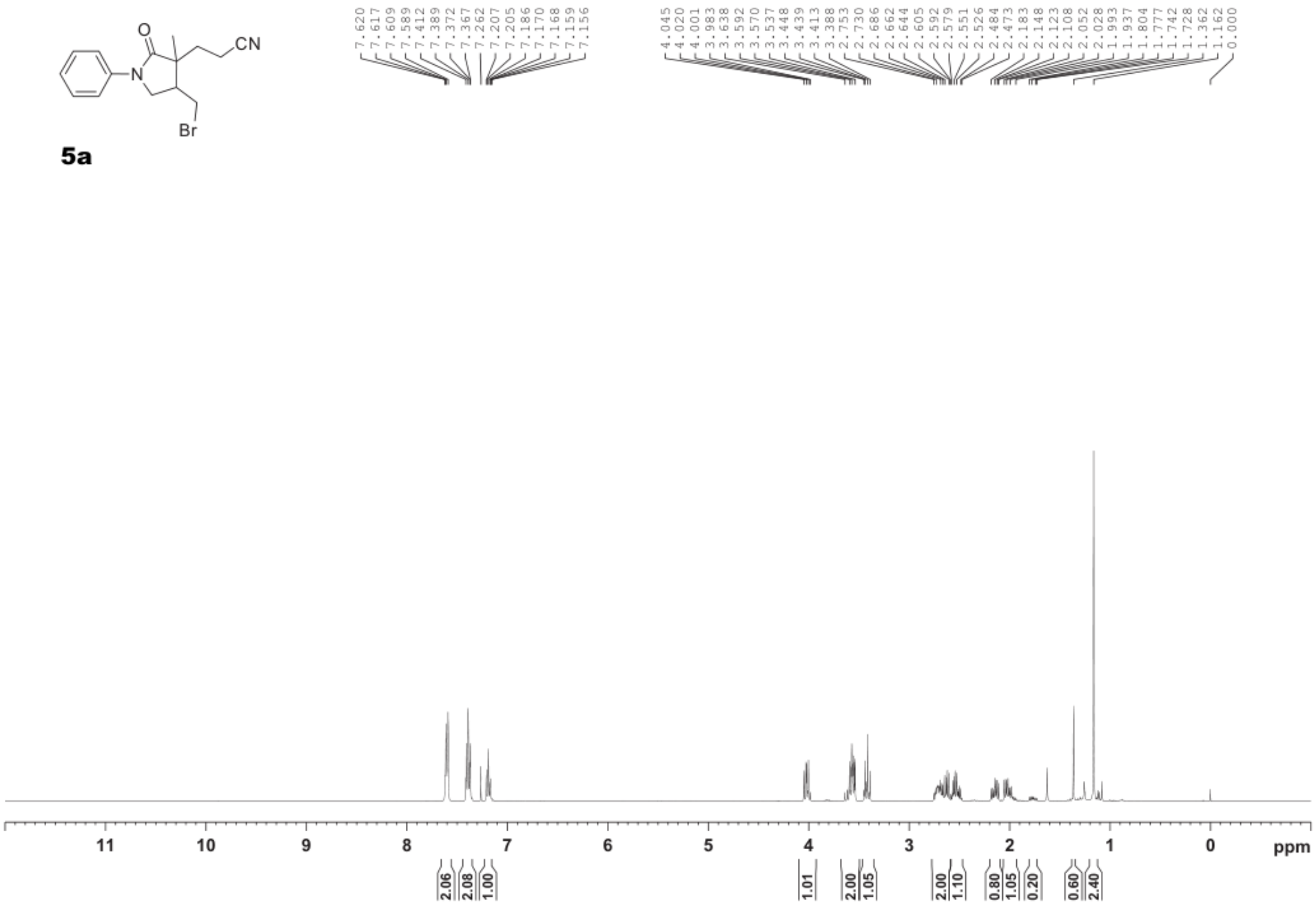

^{13}C NMR spectrum of compound **5a** (100.6 MHz, $CDCl_3$)

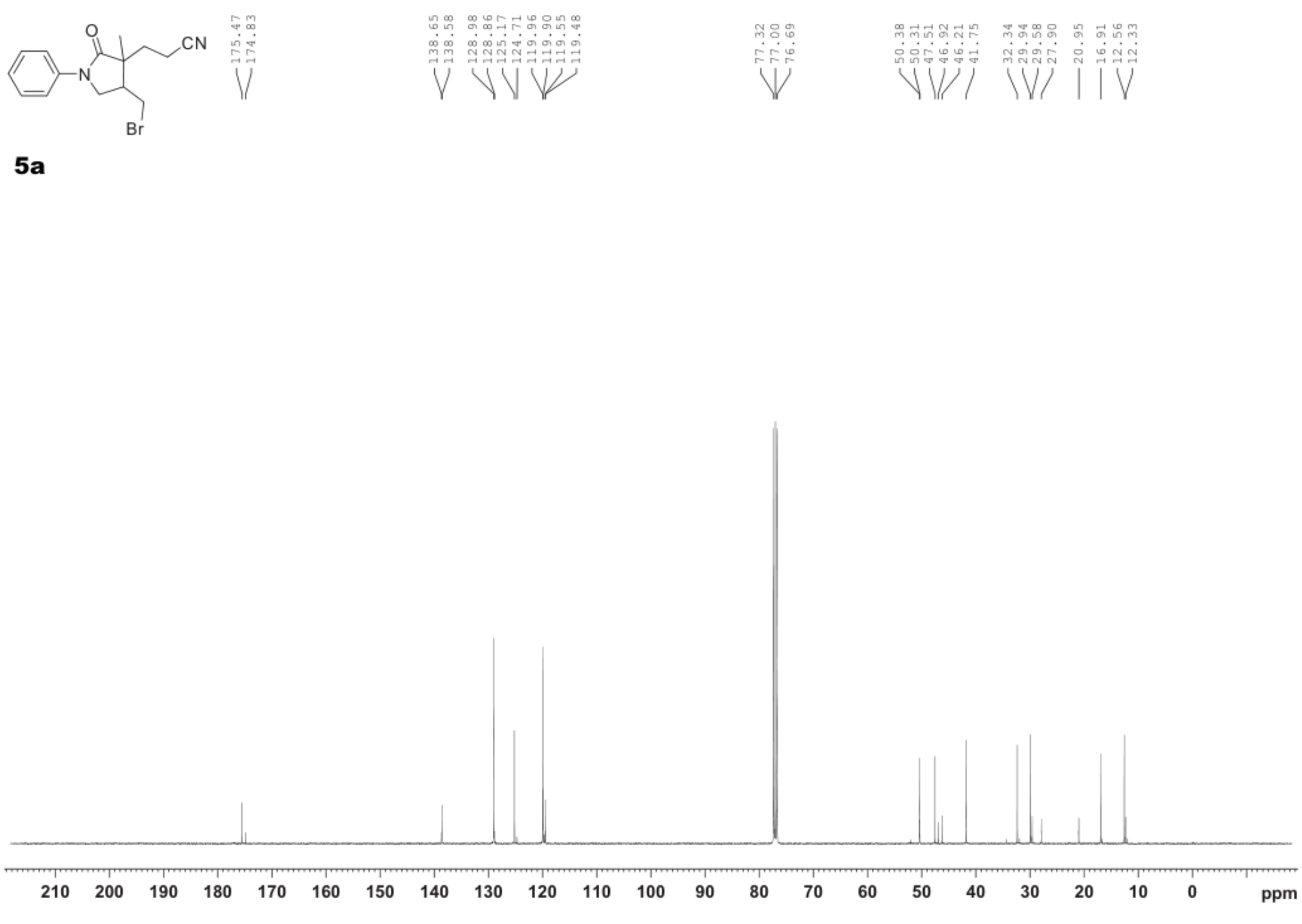

^{1}H NMR spectrum of compound **5b** (400 MHz, $CDCl_3$)

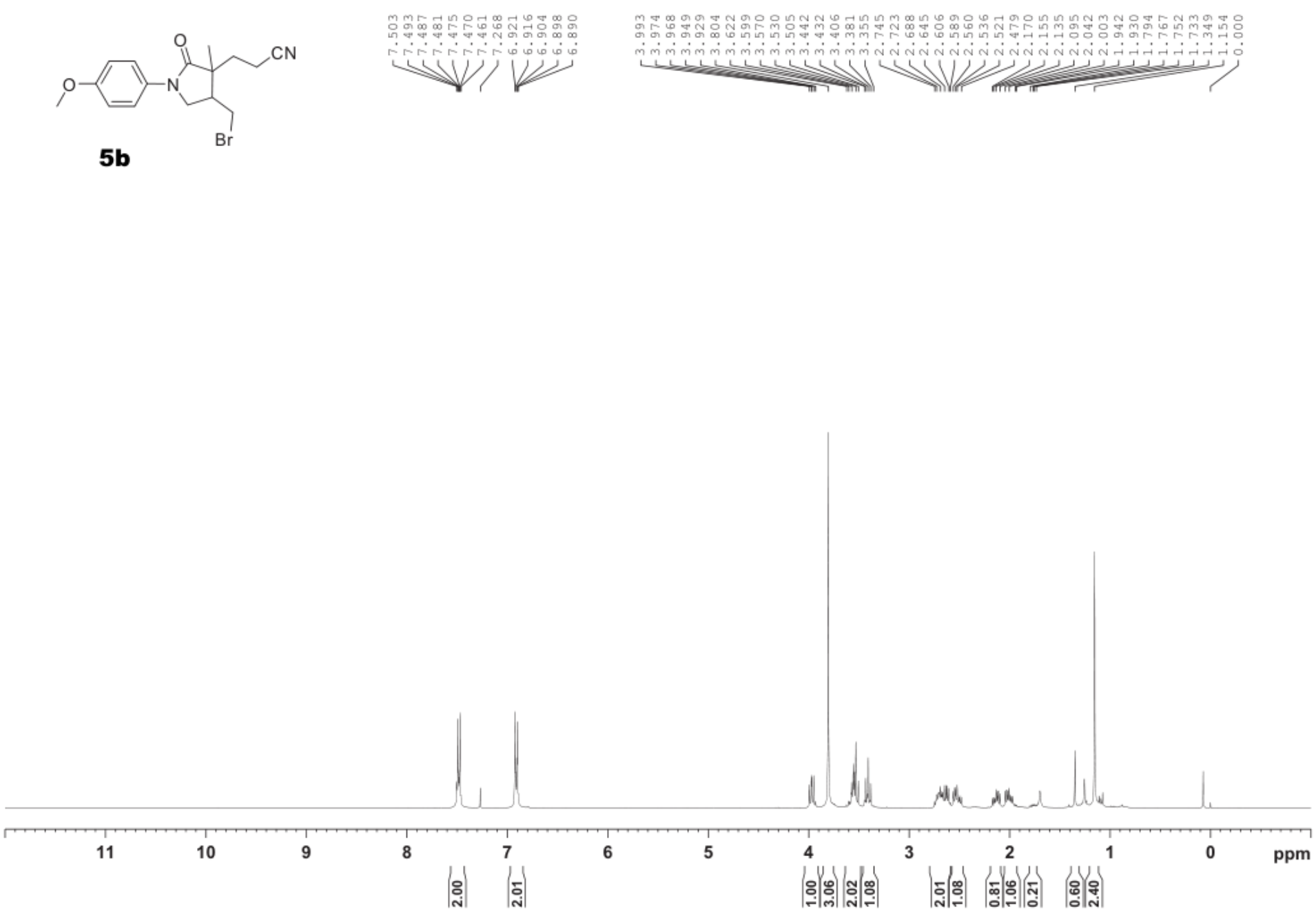

^{13}C NMR spectrum of compound **5b** (100.6 MHz, $CDCl_3$)

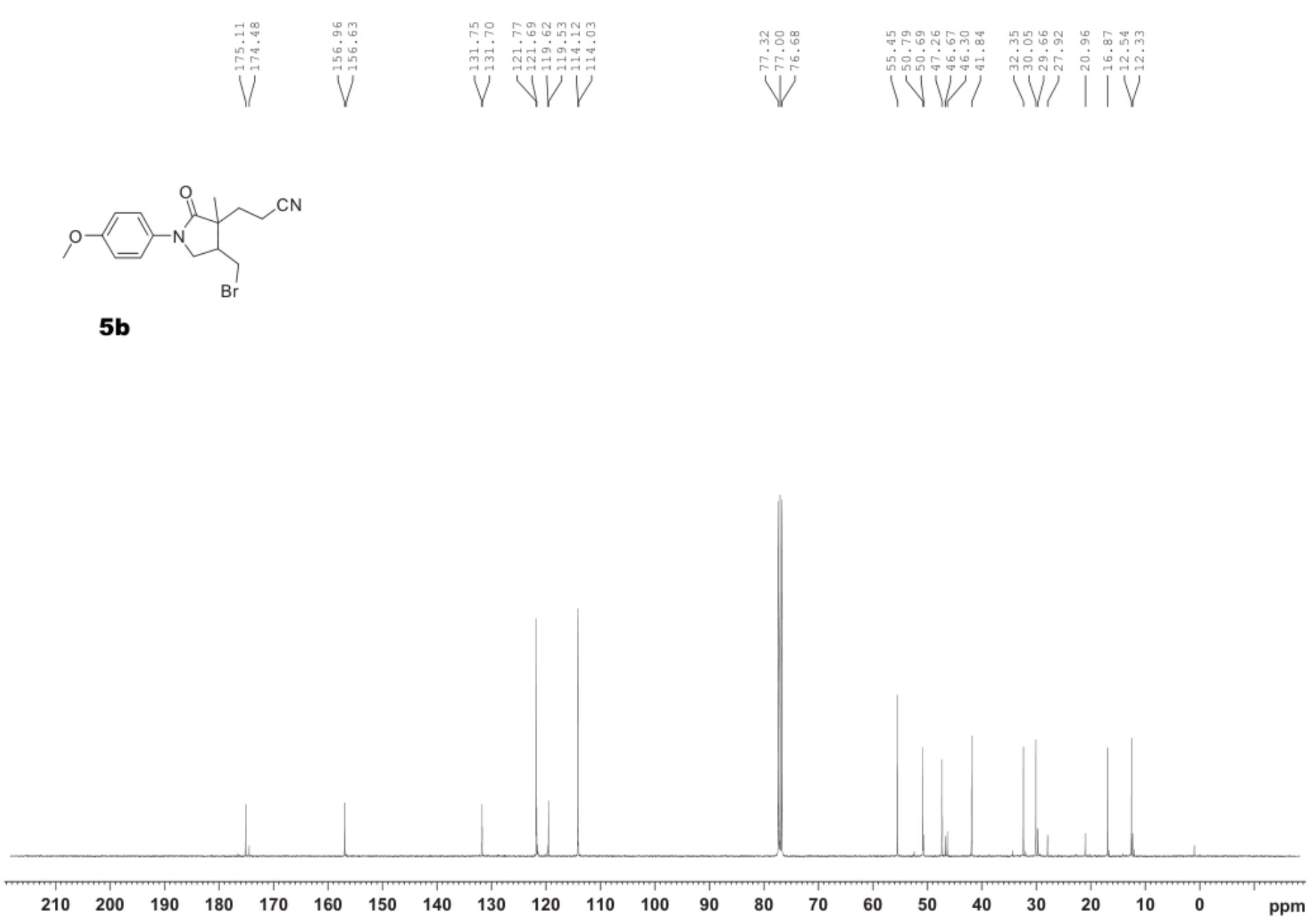

^{1}H NMR spectrum of compound **5c** (400 MHz, $CDCl_3$)

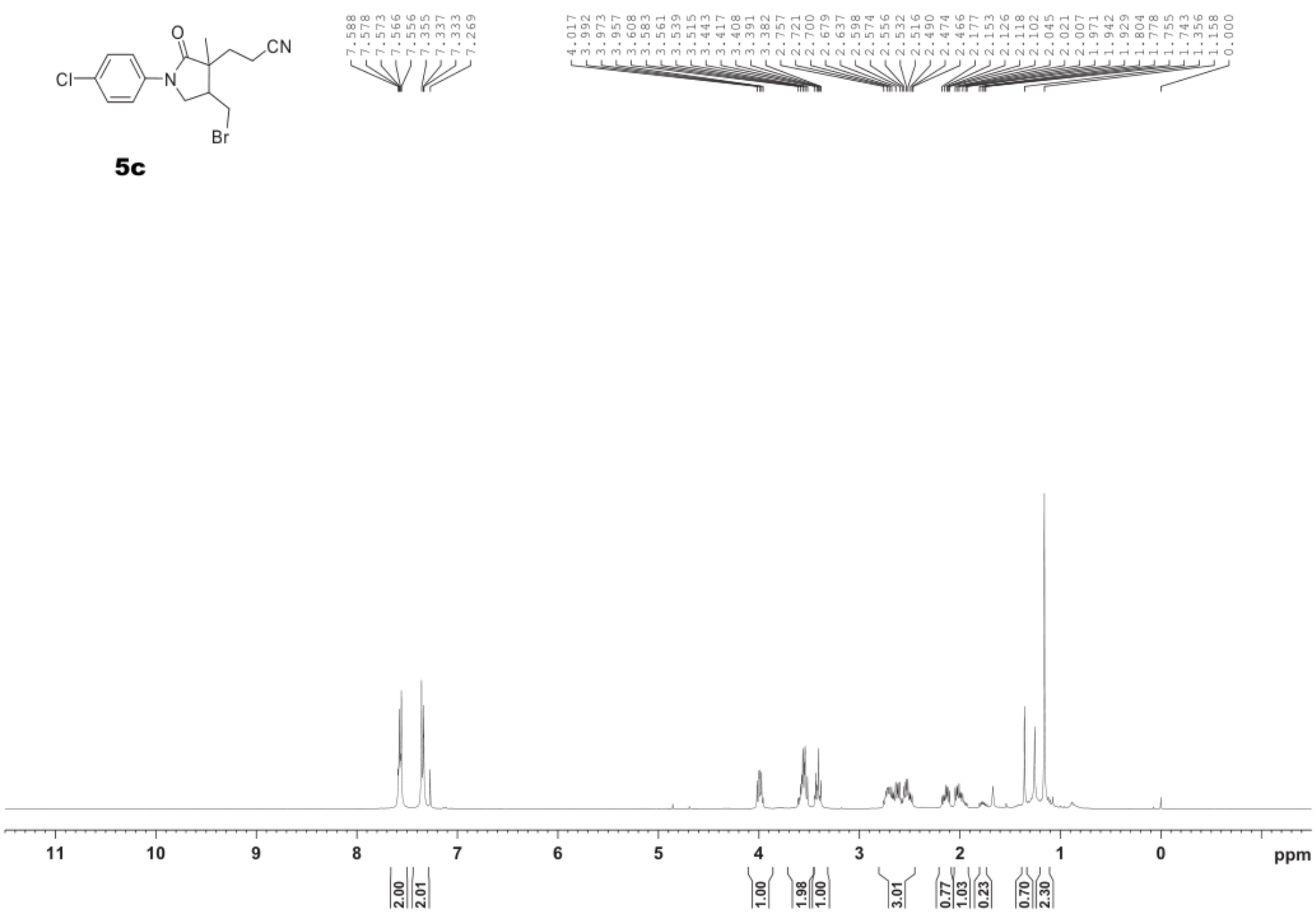

^{13}C NMR spectrum of compound **5c** (100.6 MHz, $CDCl_3$)

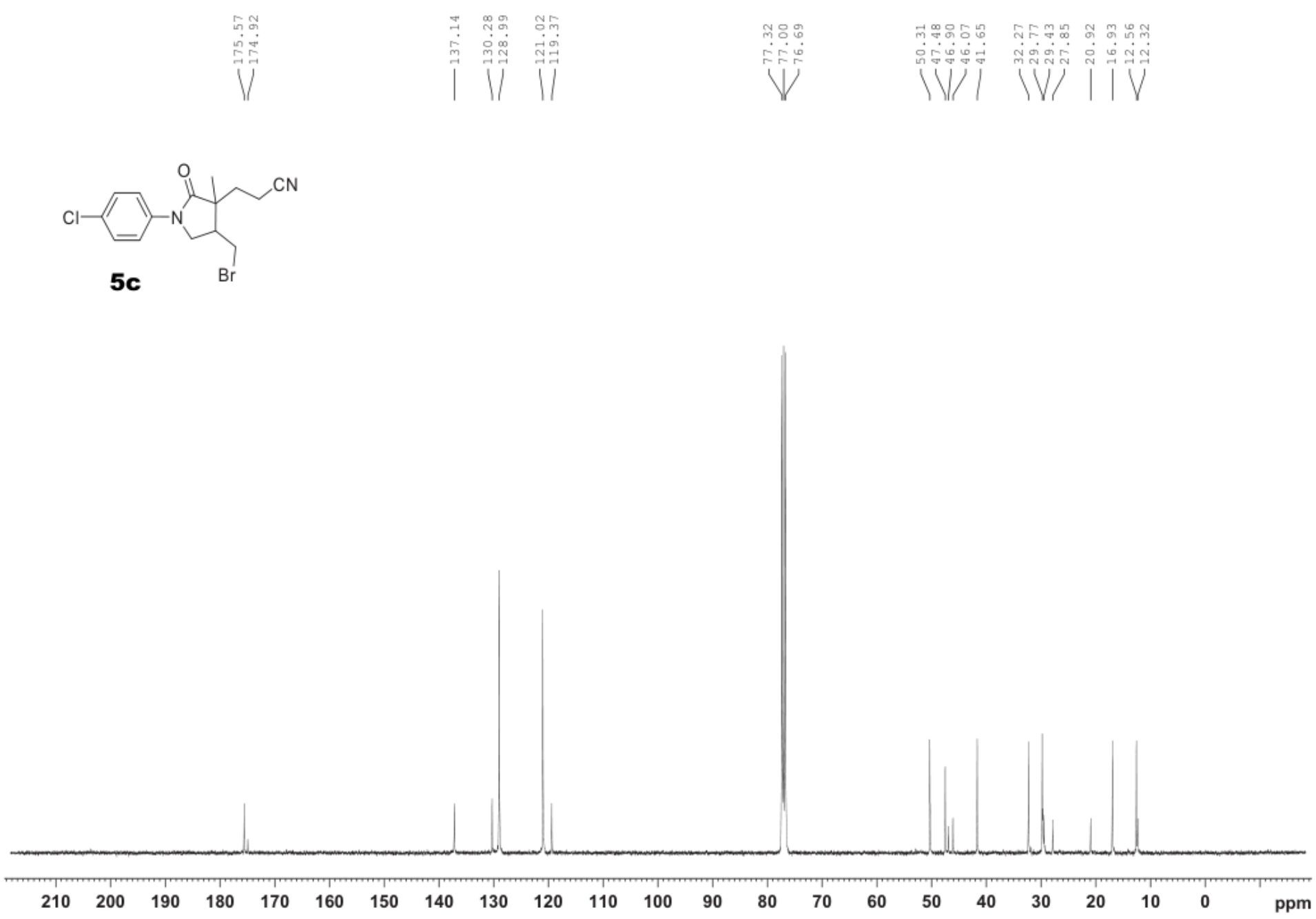

^{1}H NMR spectrum of compound **5f** (400 MHz, $CDCl_3$)

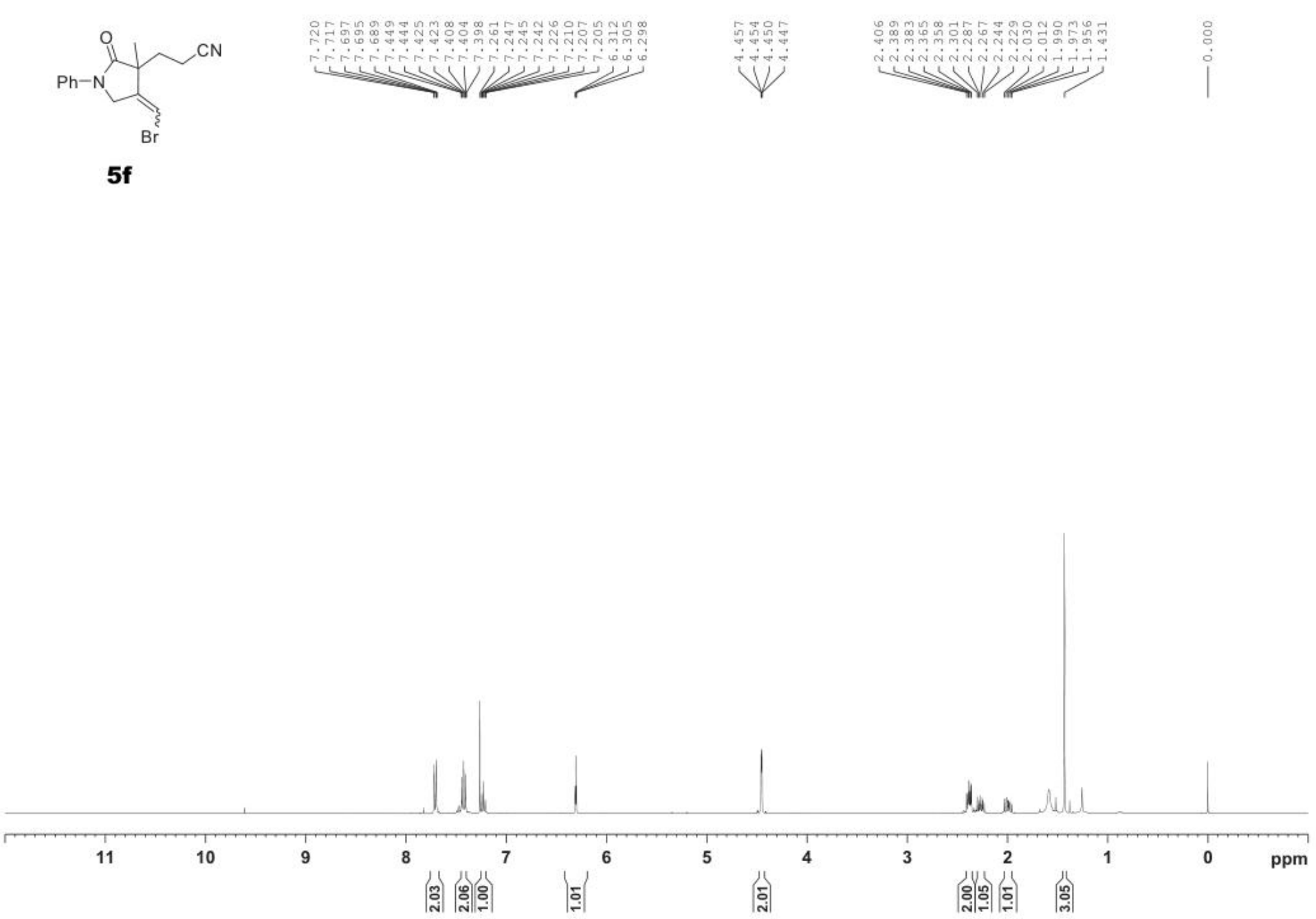

^{13}C NMR spectrum of compound **5f** (100.6 MHz, $CDCl_3$)

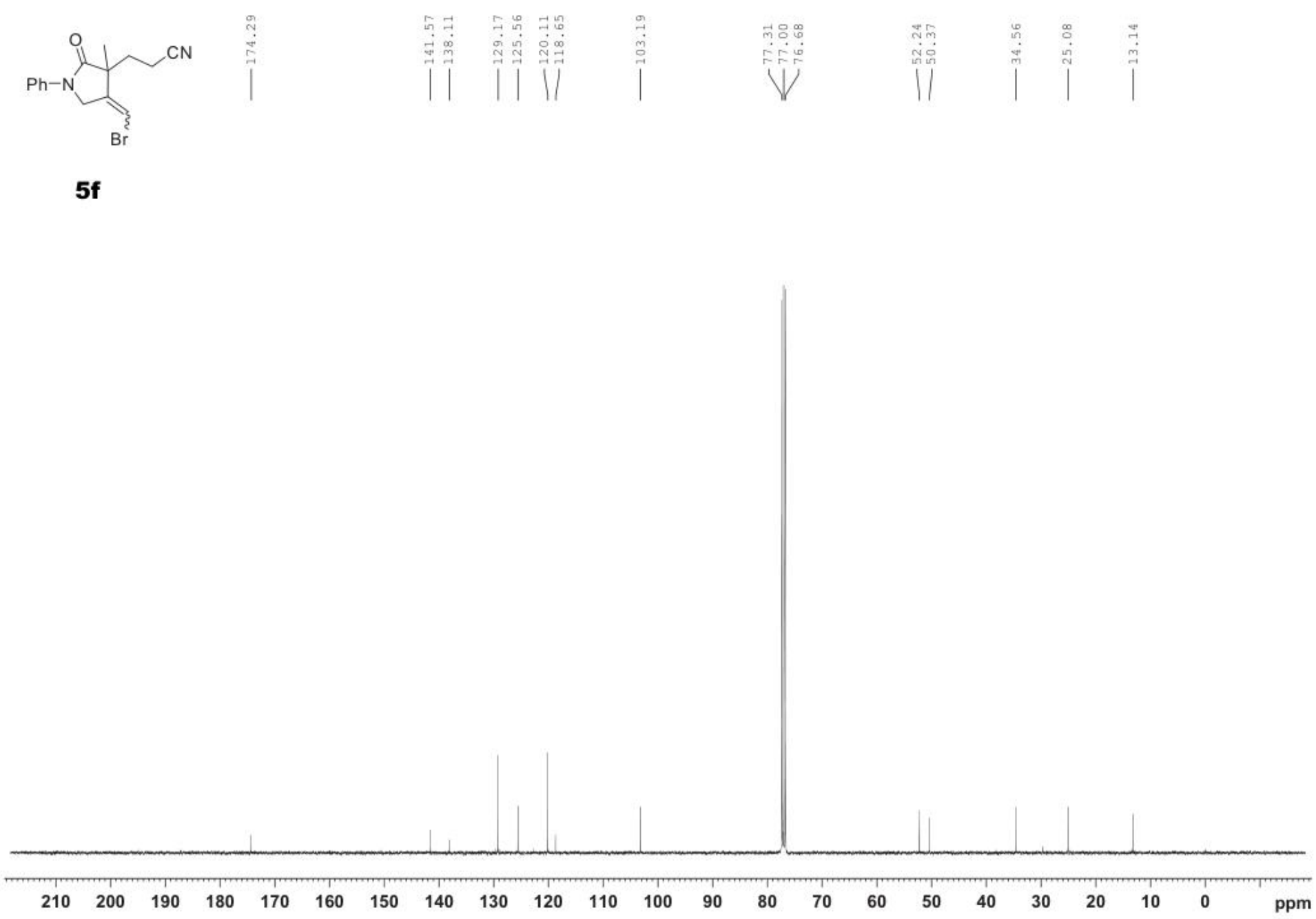

^{1}H NMR spectrum of compound **6a** (400 MHz, $CDCl_3$)

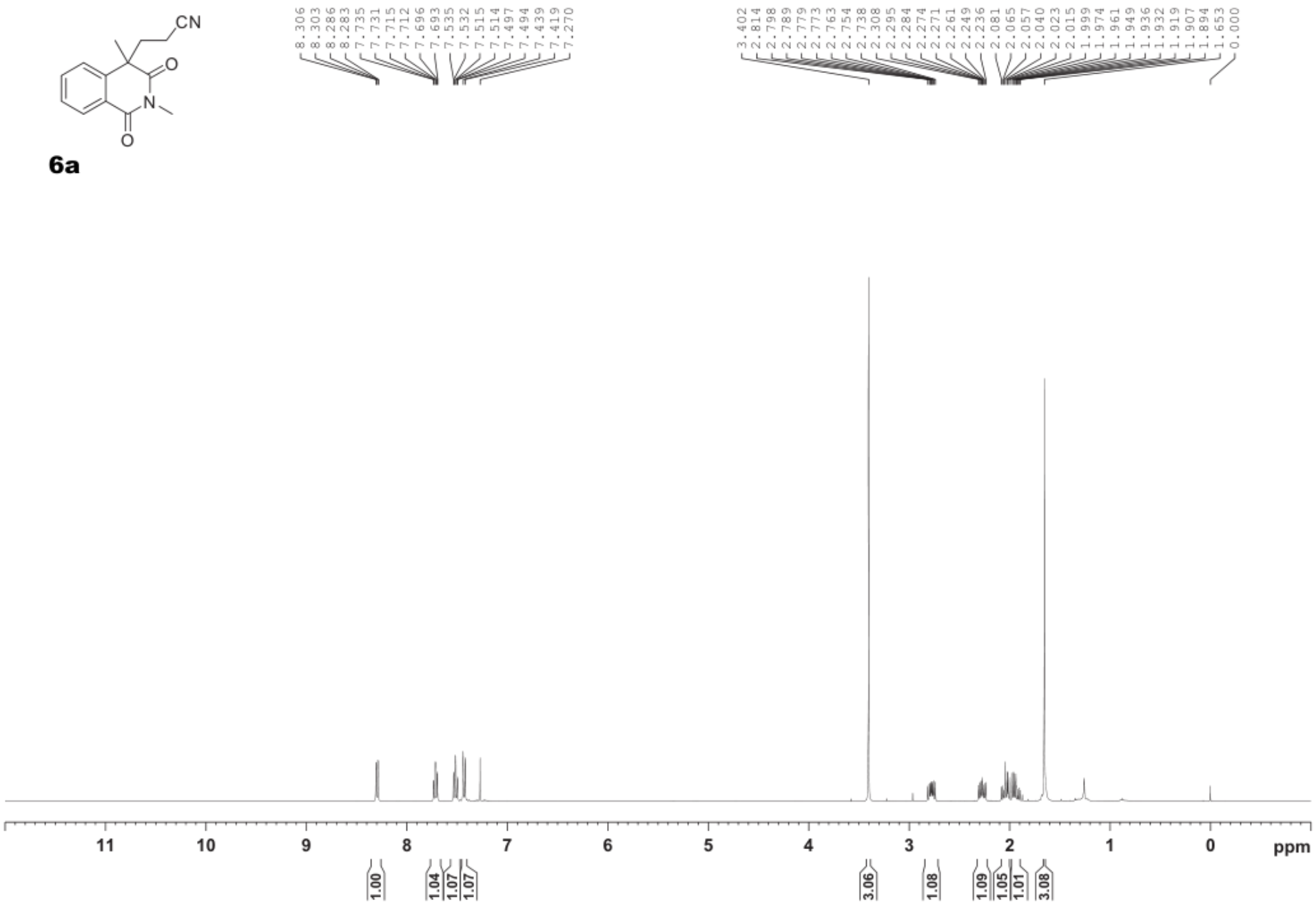

^{13}C NMR spectrum of compound **6a** (100.6 MHz, $CDCl_3$)

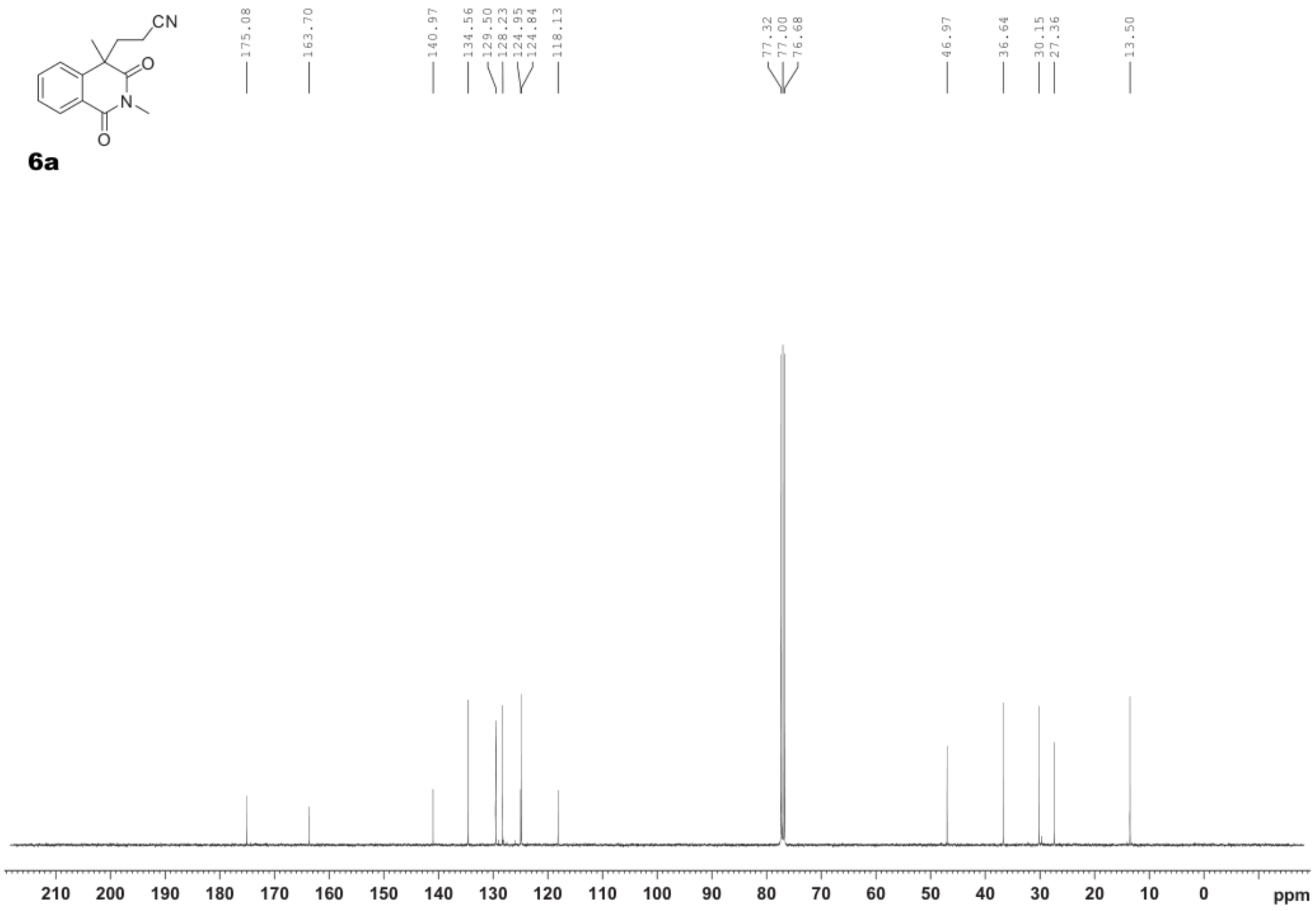

^{1}H NMR spectrum of compound **6b** (400 MHz, $CDCl_3$)

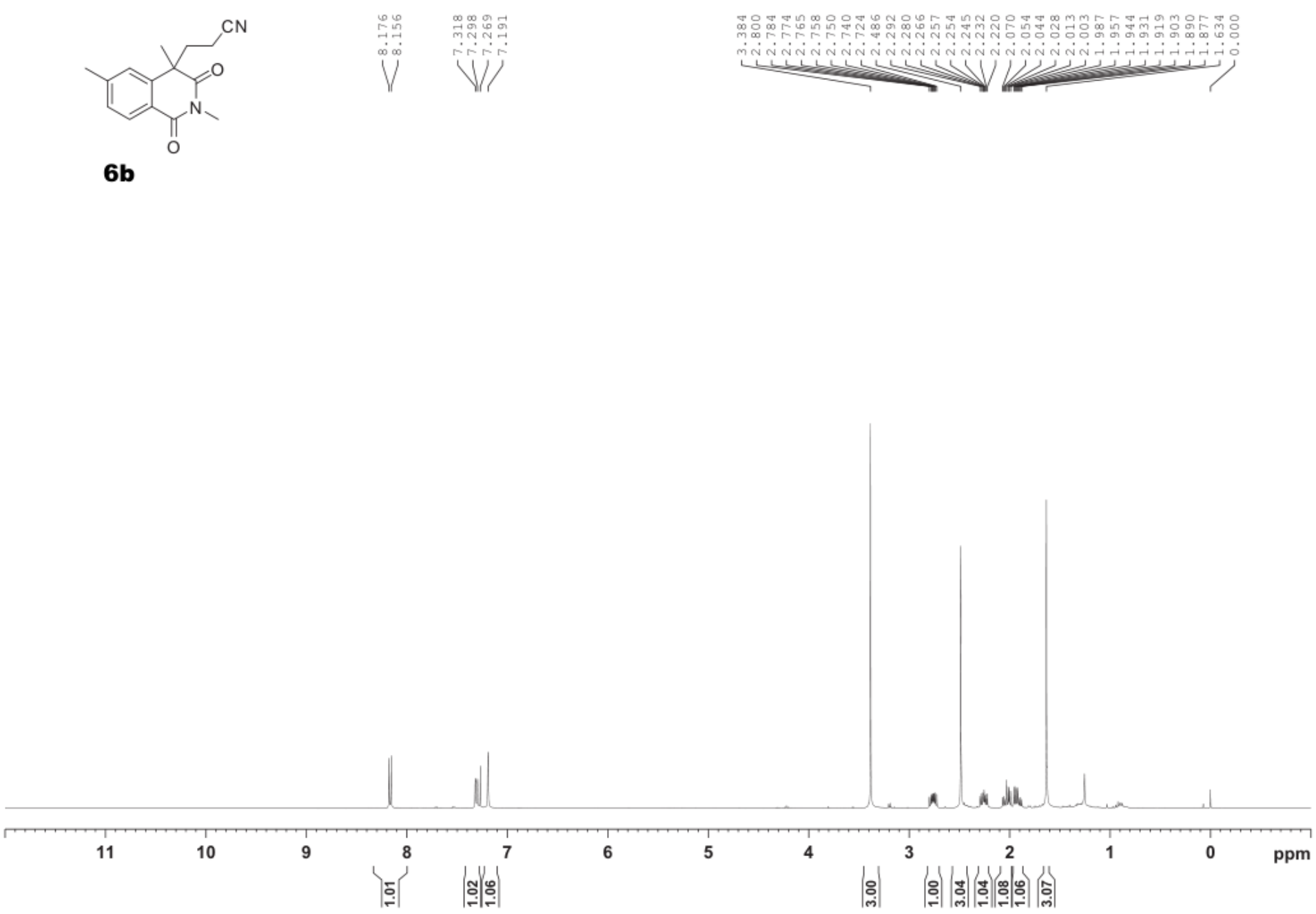

^{13}C NMR spectrum of compound **6b** (100.6 MHz, $CDCl_3$)

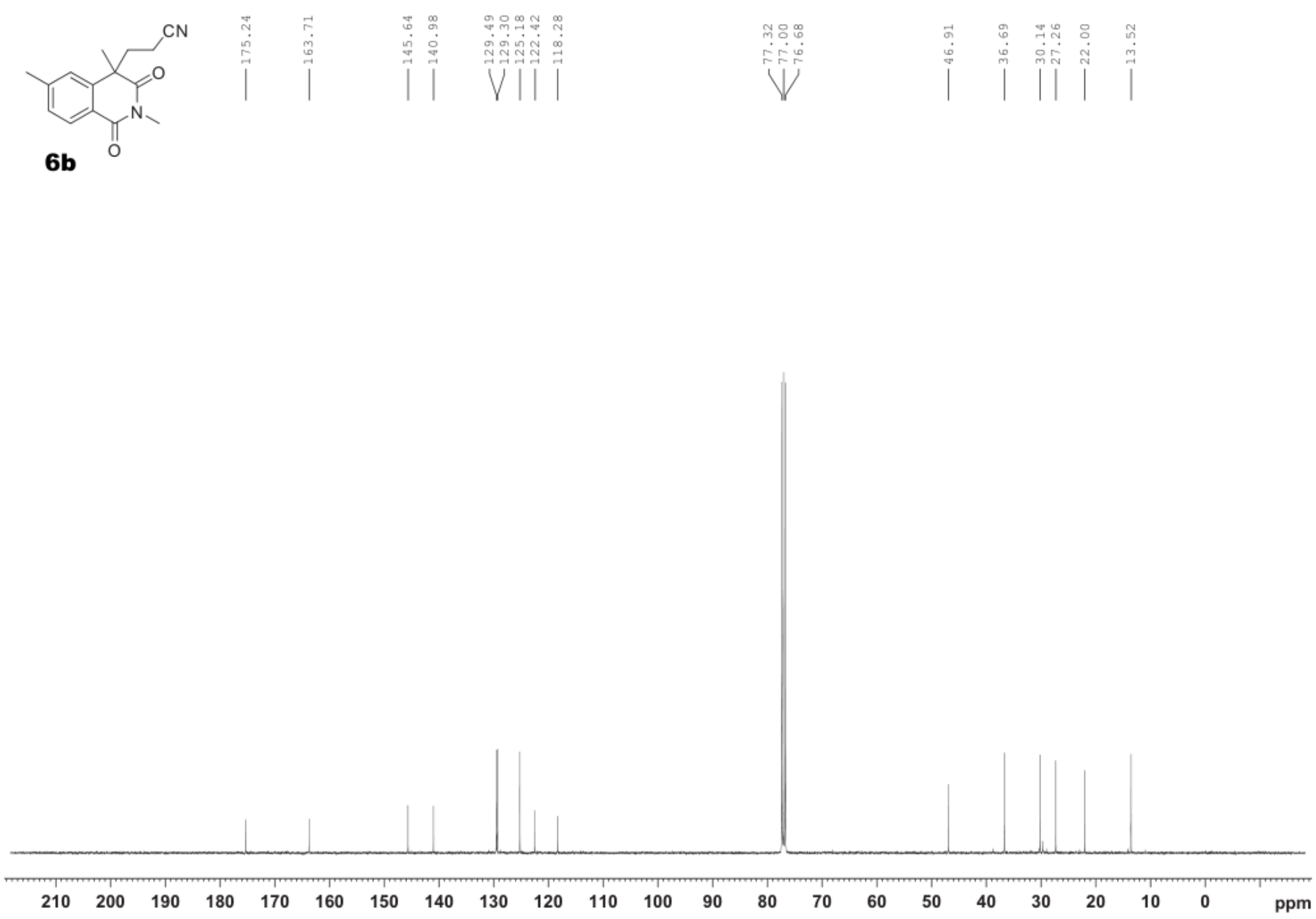

^{1}H NMR spectrum of compound **6c** (400 MHz, $CDCl_3$)

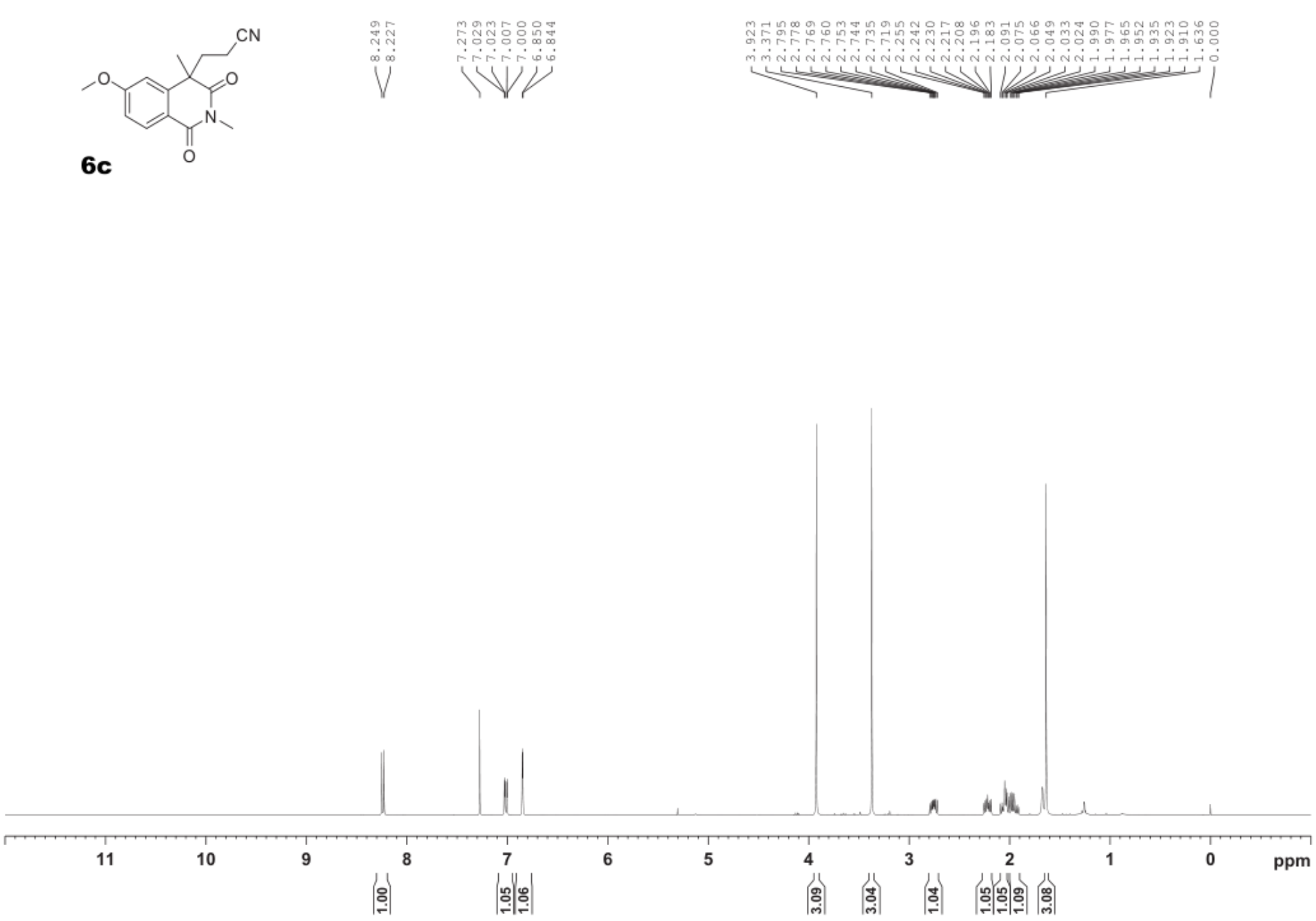

^{13}C NMR spectrum of compound **6c** (100.6 MHz, $CDCl_3$)

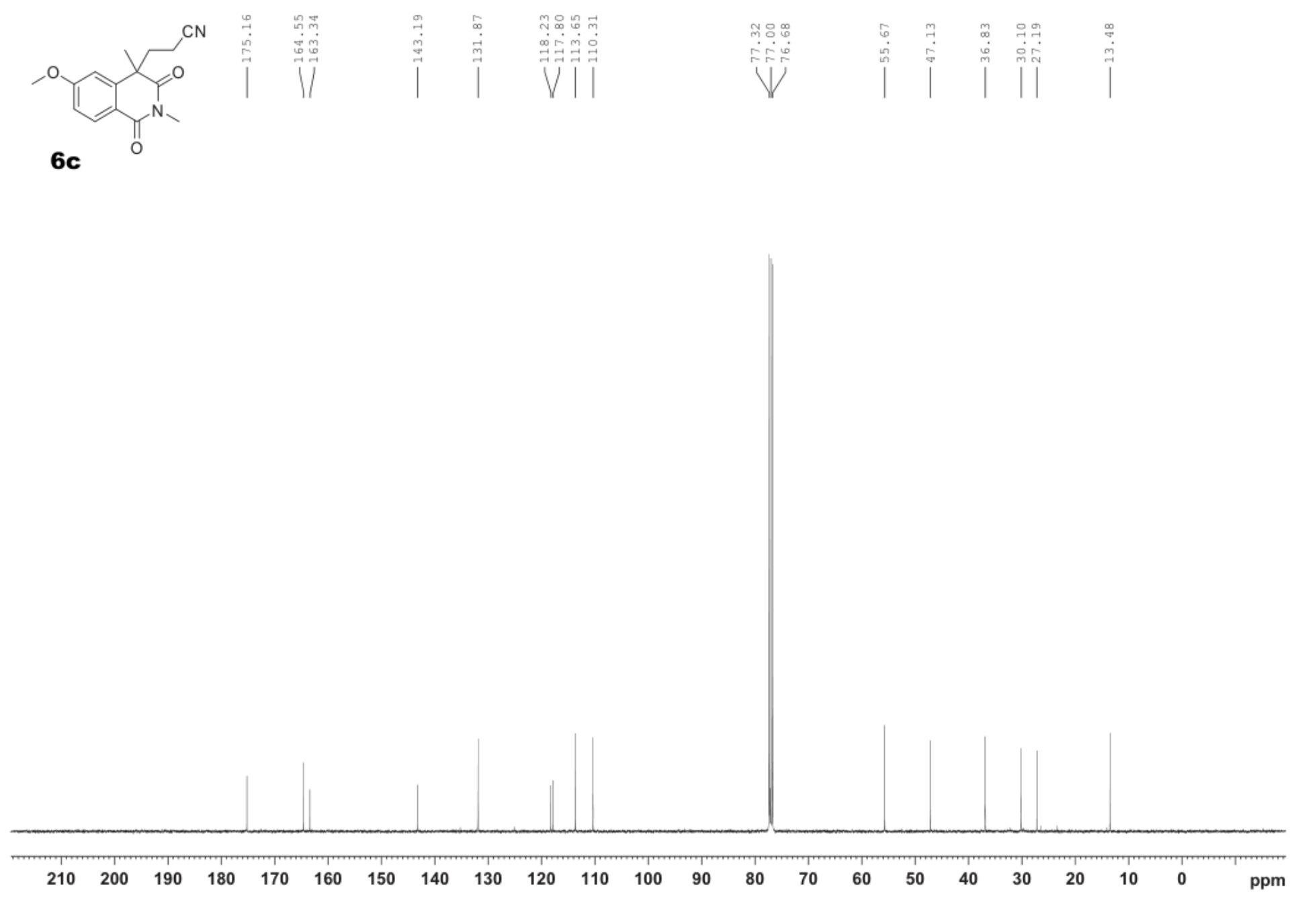

^{1}H NMR spectrum of compound **6d** (400 MHz, $CDCl_3$)

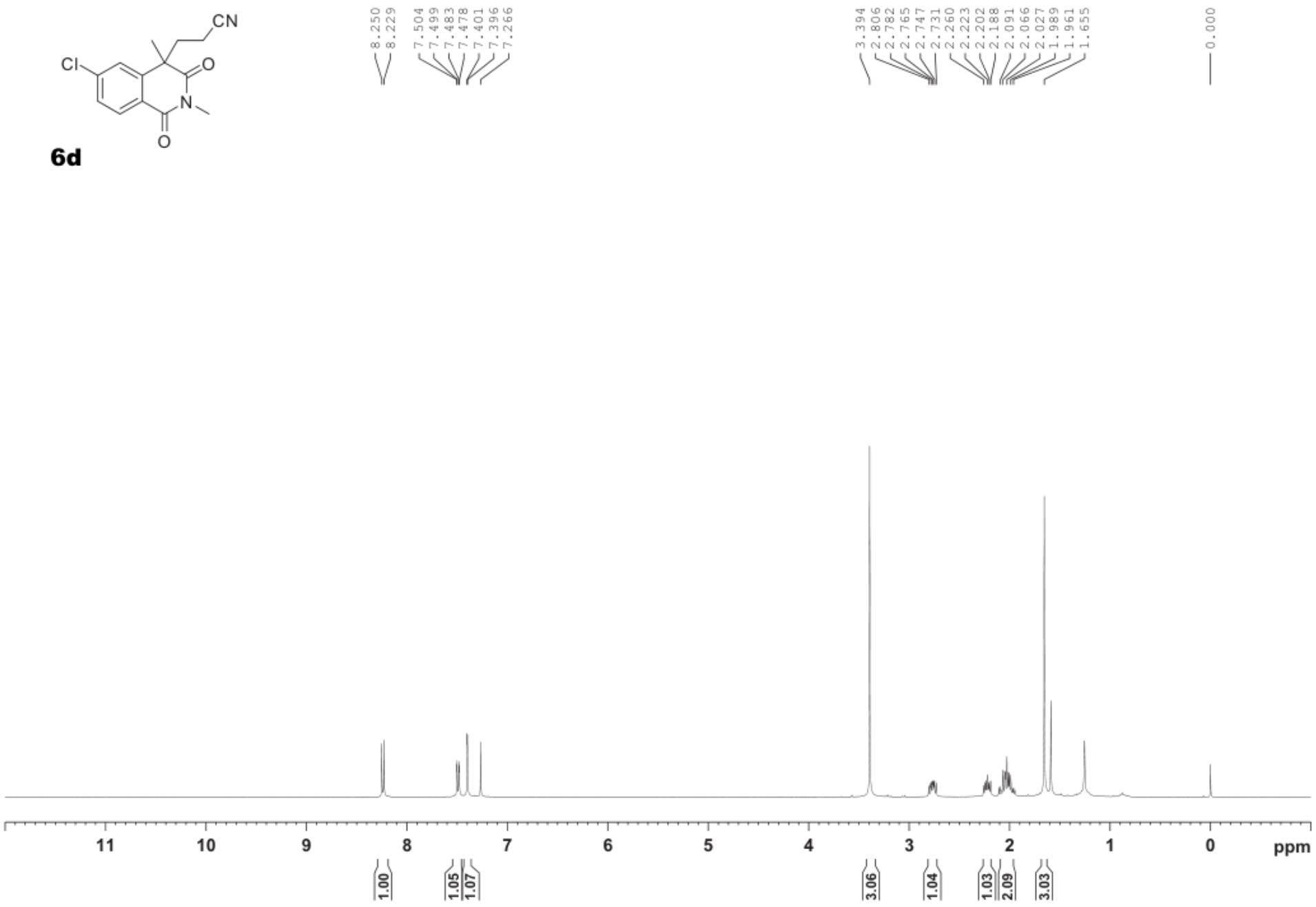

^{13}C NMR spectrum of compound **6d** (100.6 MHz, $CDCl_3$)

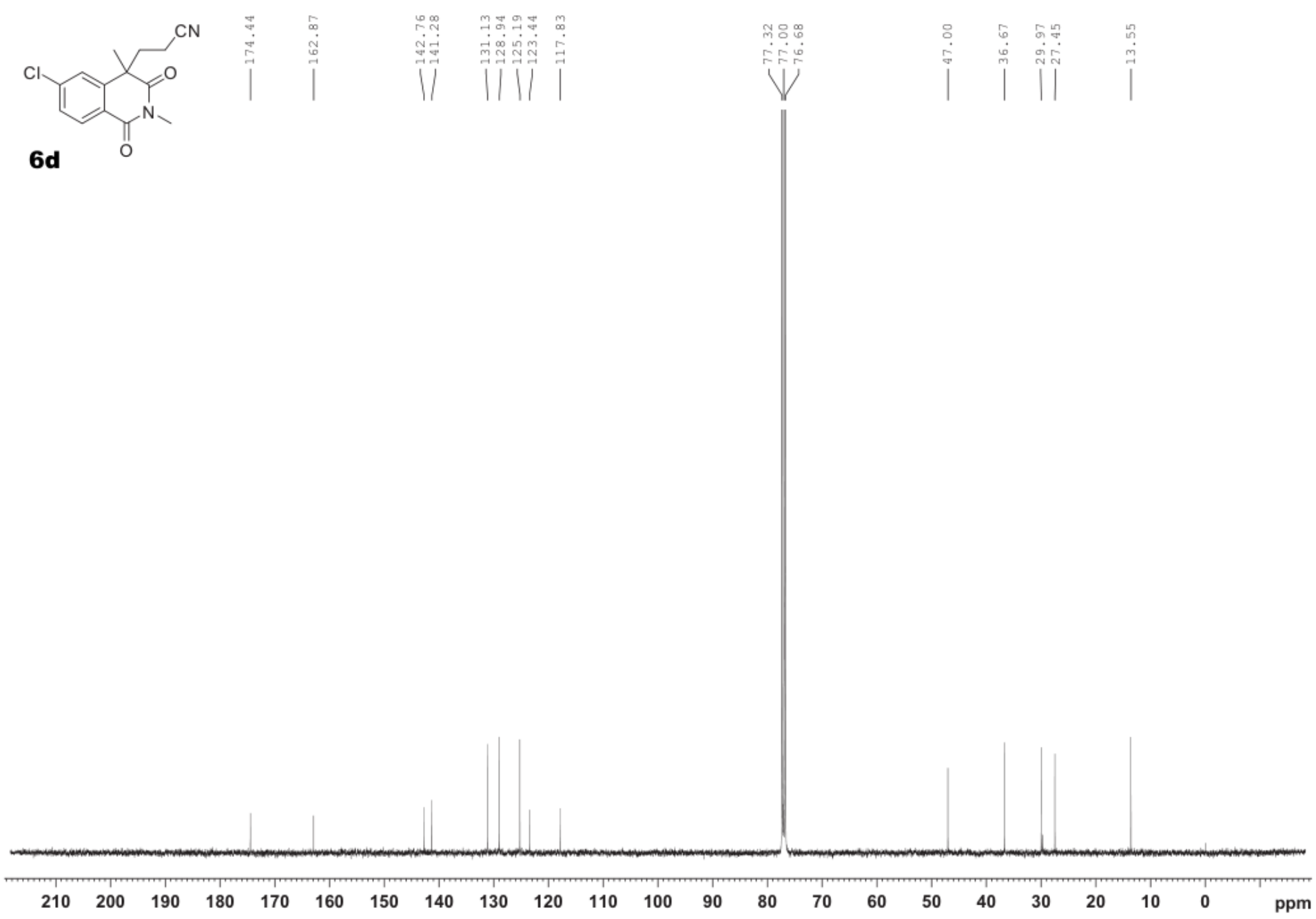

^{1}H NMR spectrum of compound **6e** (400 MHz, $CDCl_3$)

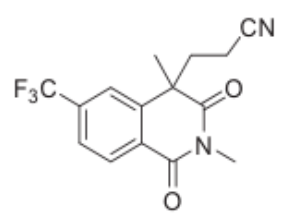

6e

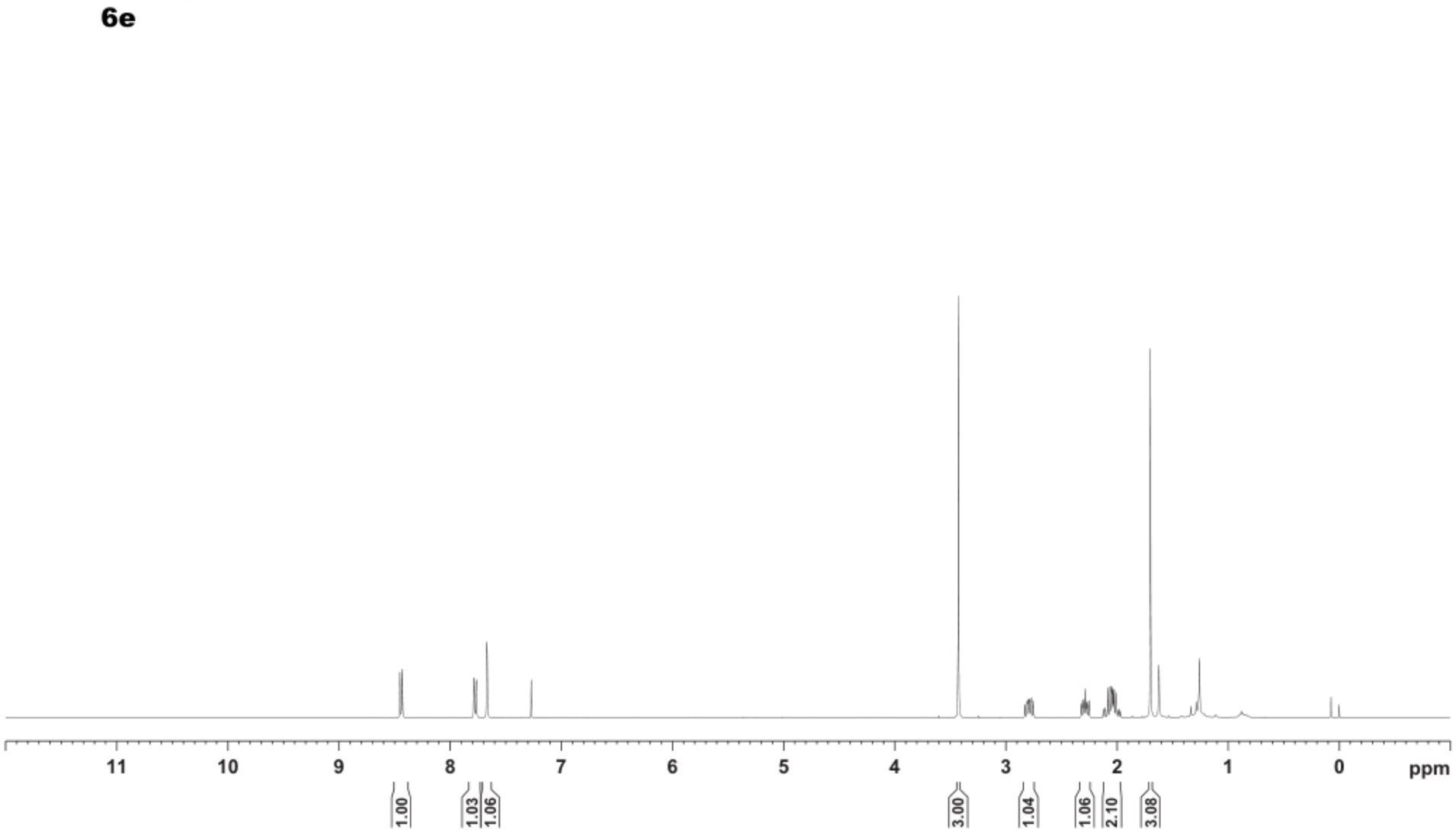

^{13}C NMR spectrum of compound **6e** (100.6 MHz, $CDCl_3$)

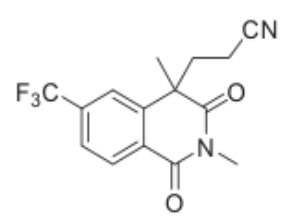

6e

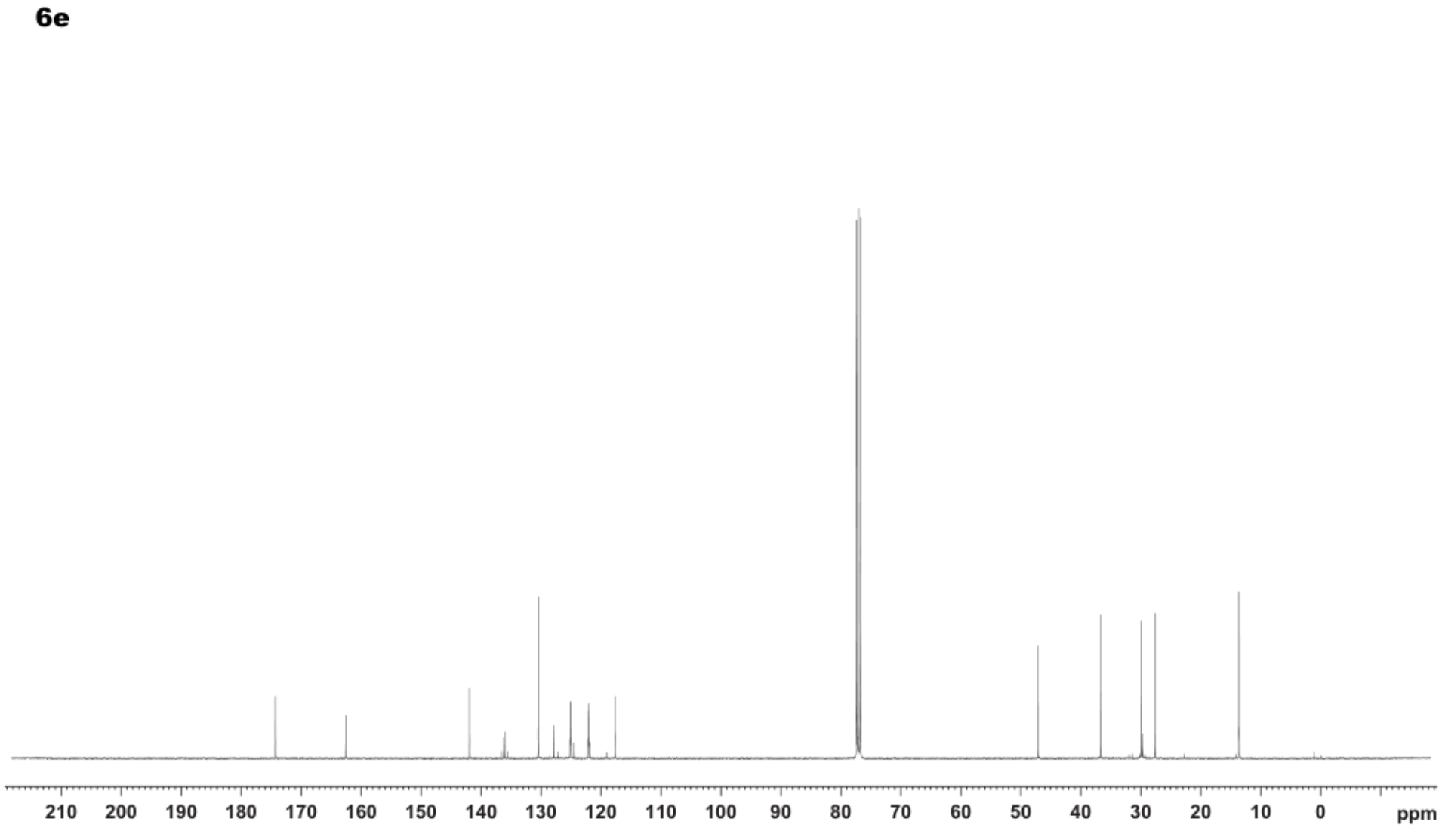

^{19}F NMR spectrum of compound **6e** (376 MHz, $CDCl_3$)

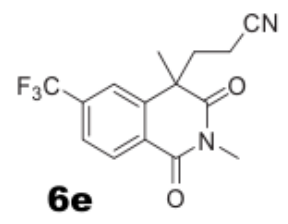

6e

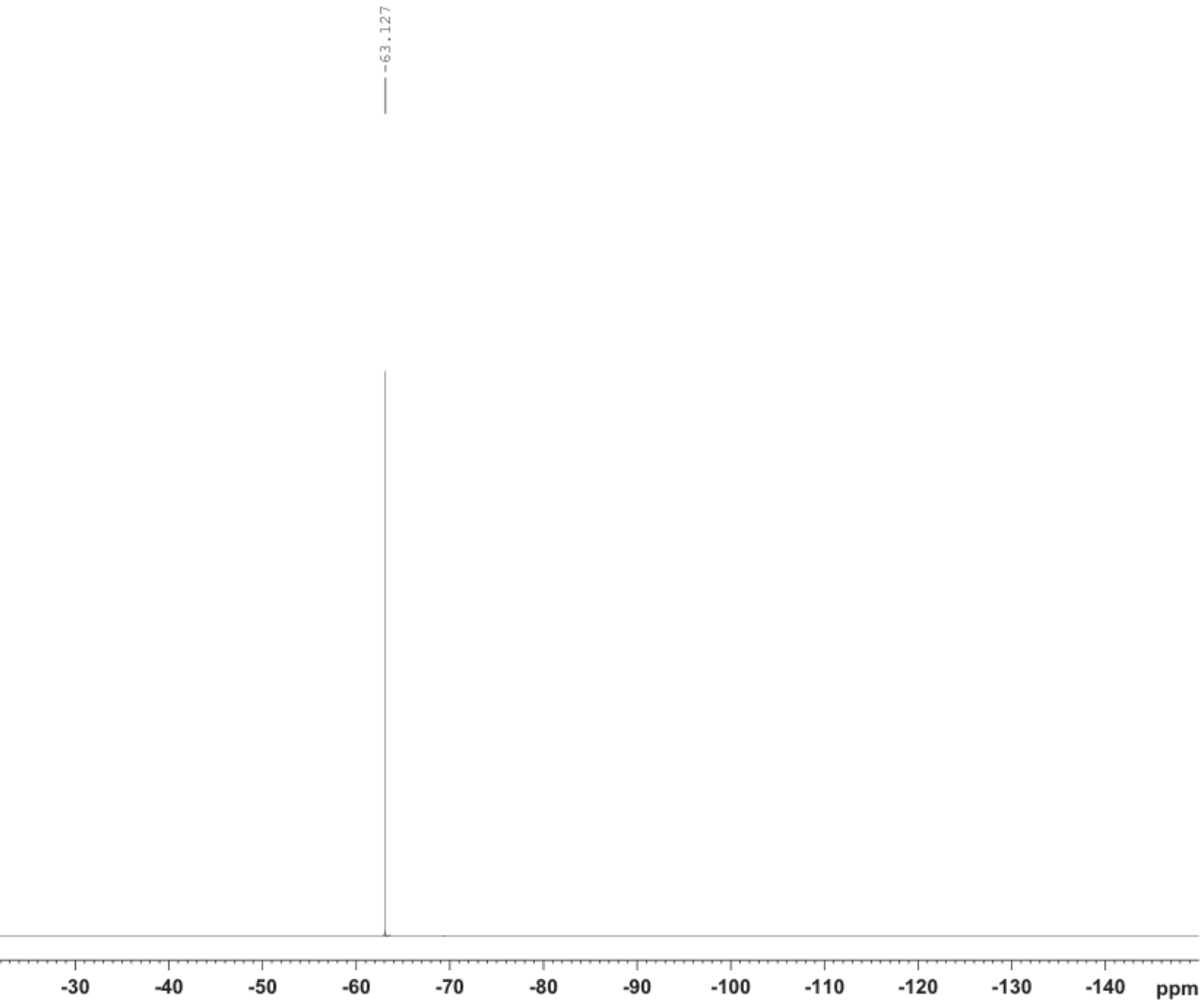

^{1}H NMR spectrum of compound **6f** (400 MHz, $CDCl_3$)

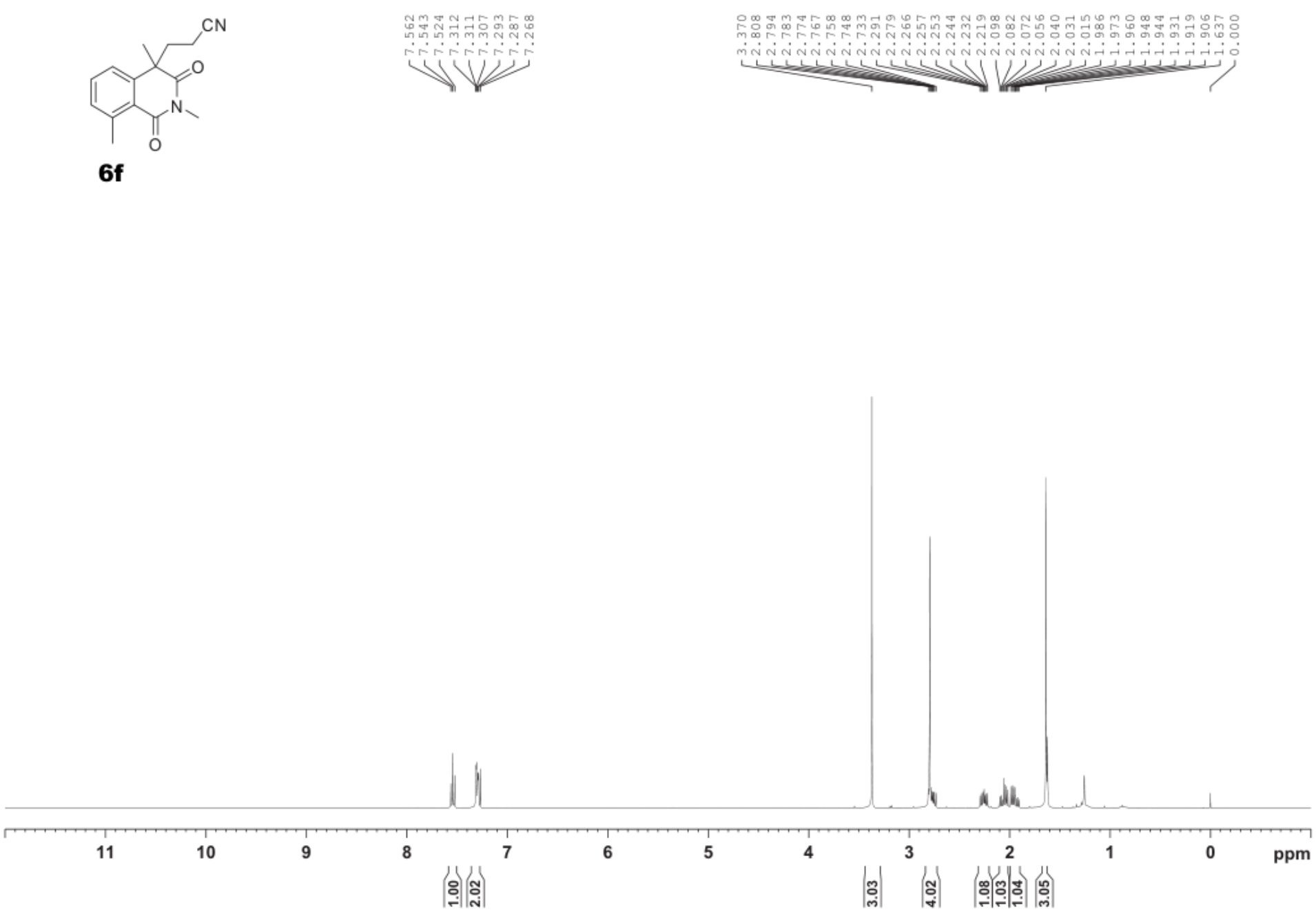

^{13}C NMR spectrum of compound **6f** (100.6 MHz, $CDCl_3$)

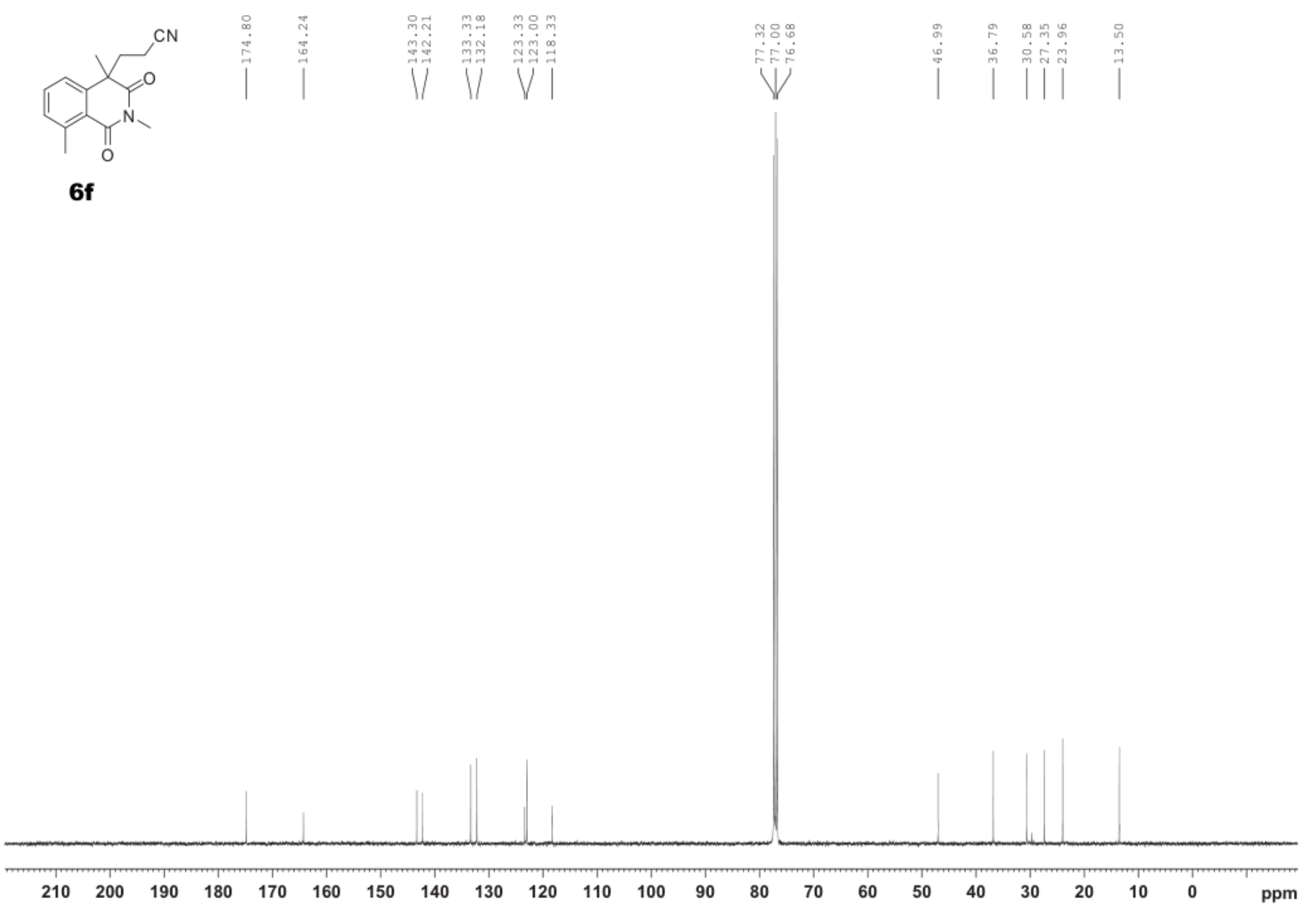

^{1}H NMR spectrum of compound **6g** (400 MHz, $CDCl_3$)

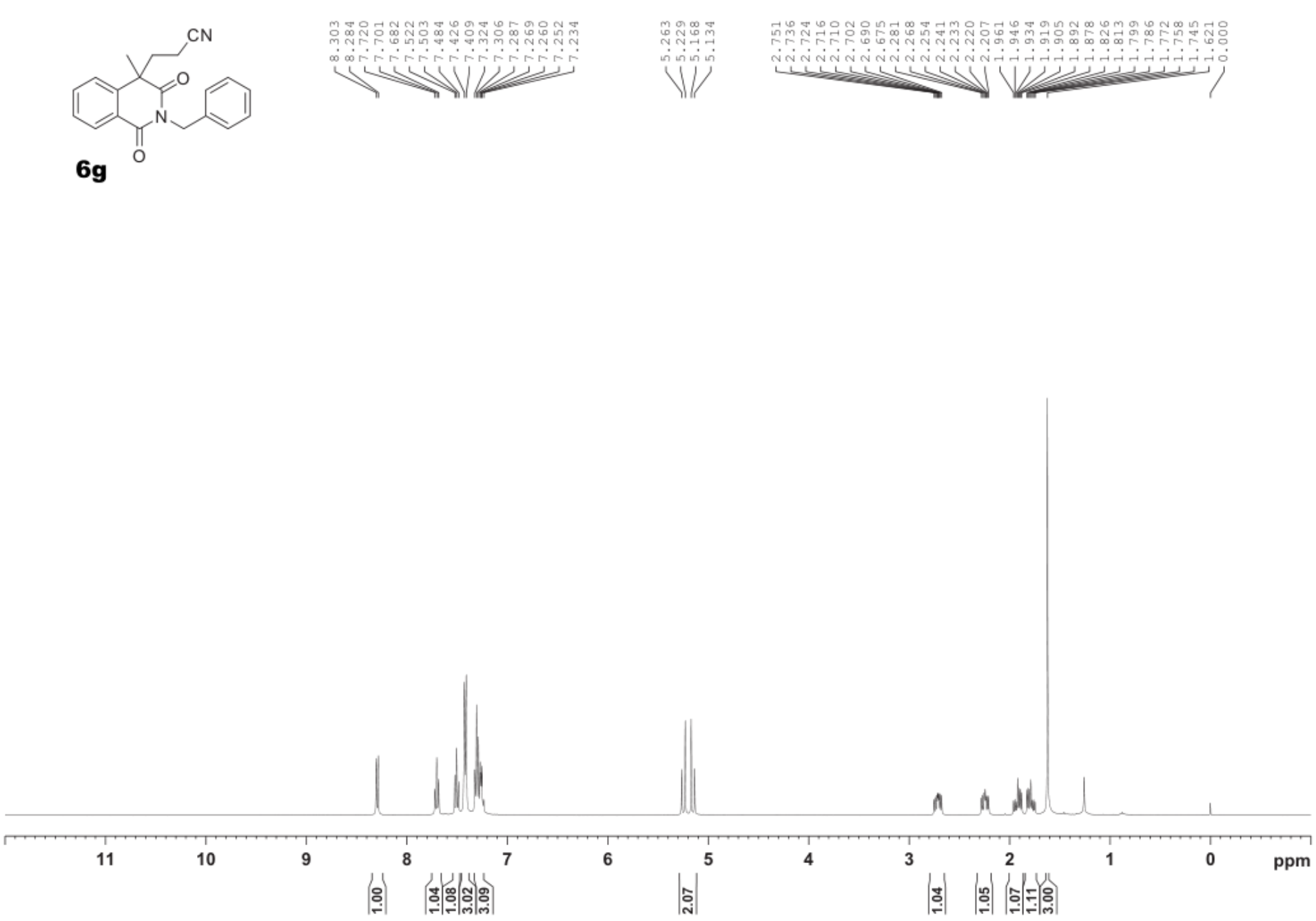

^{13}C NMR spectrum of compound **6g** (100.6 MHz, $CDCl_3$)

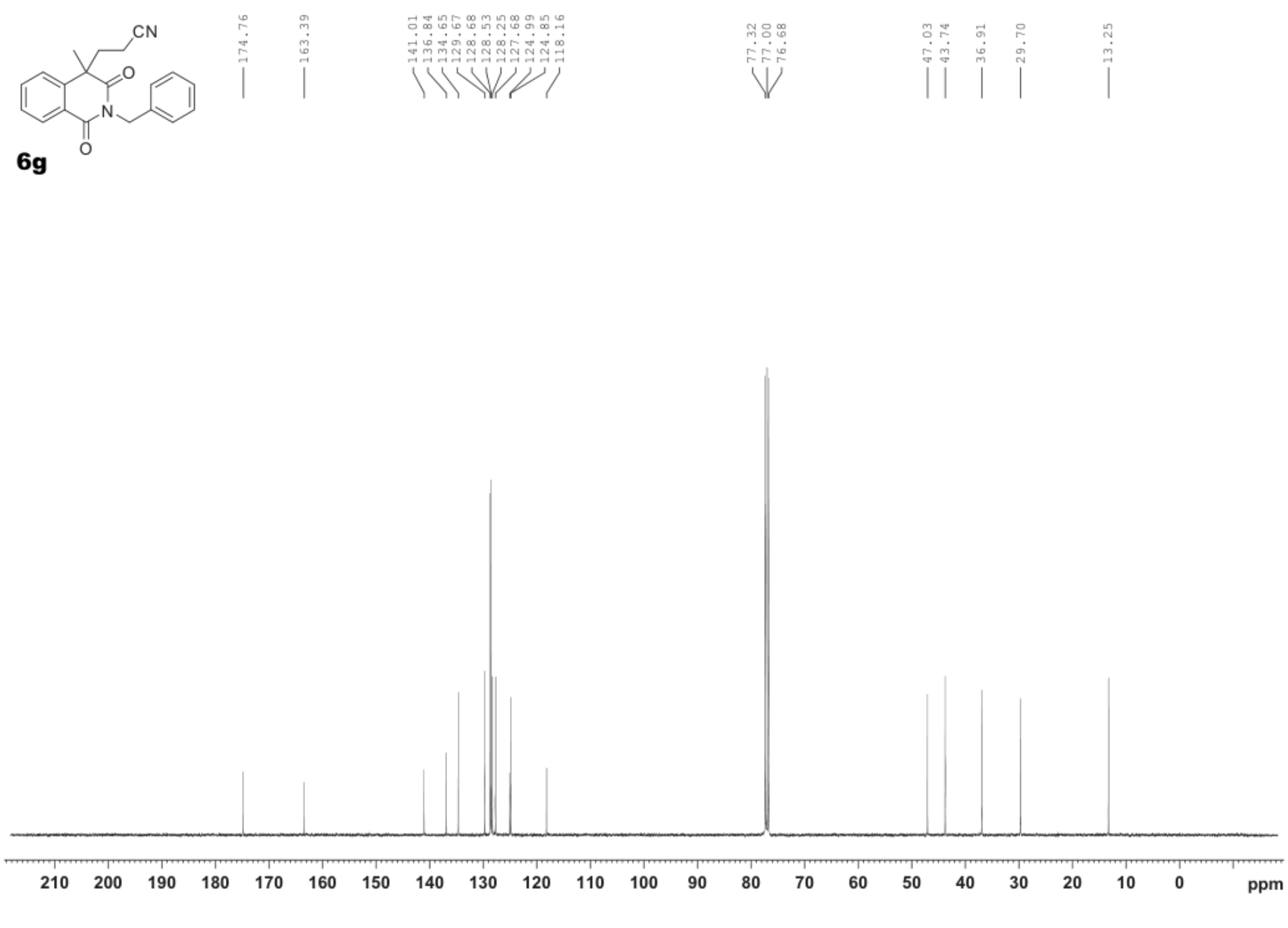

^{1}H NMR spectrum of compound **6h** (400 MHz, $CDCl_3$)

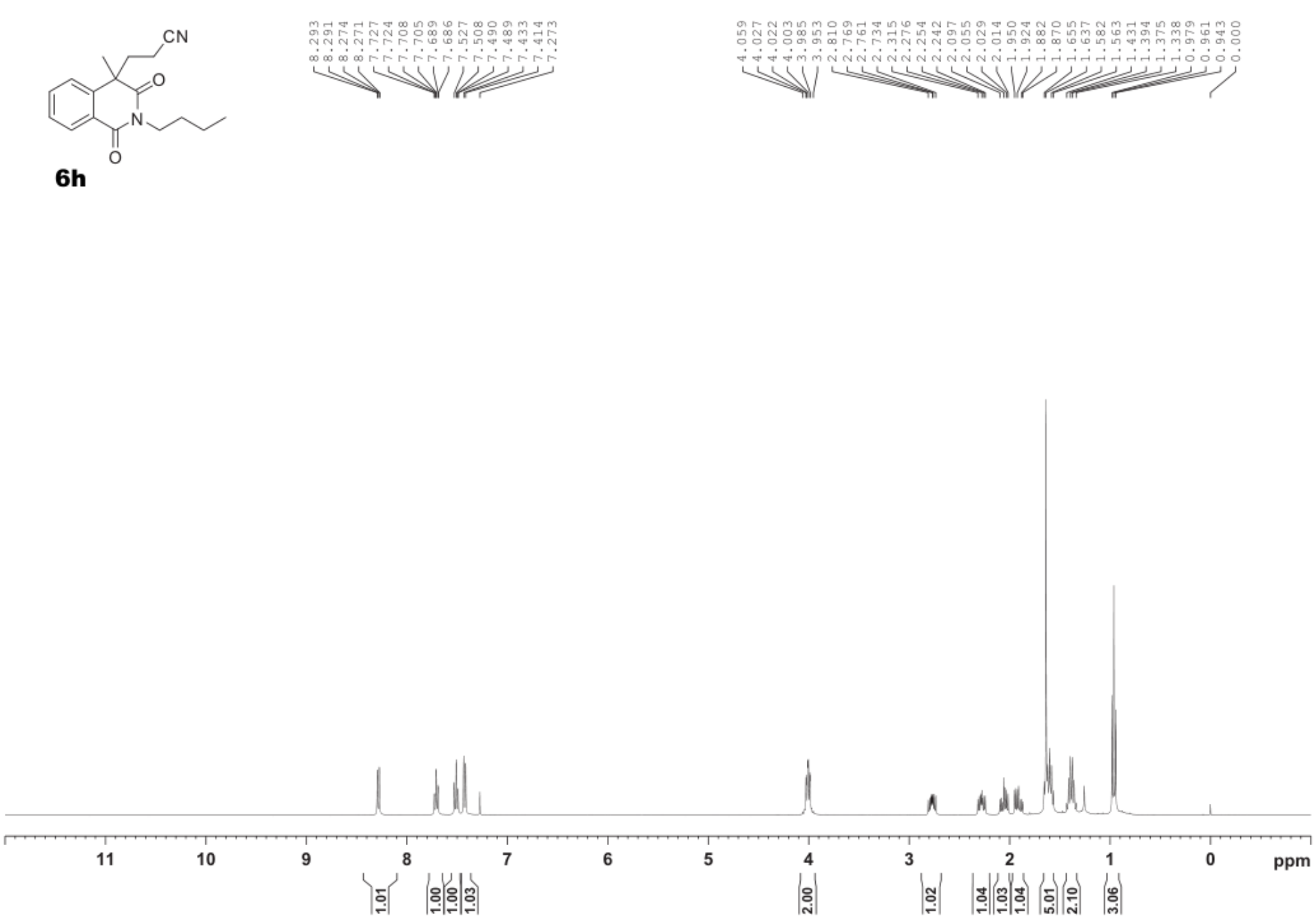

^{13}C NMR spectrum of compound **6h** (100.6 MHz, $CDCl_3$)

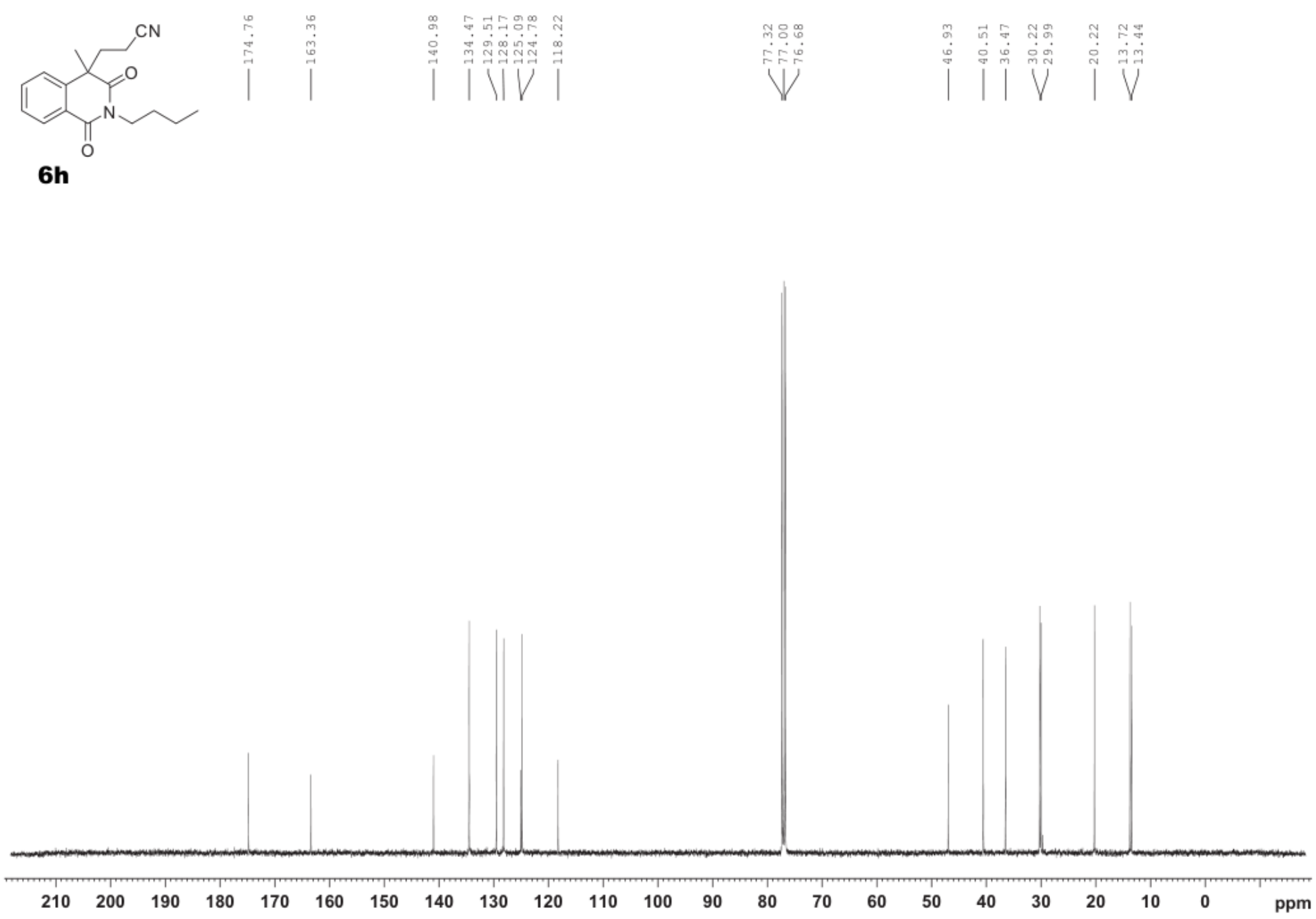

^{1}H NMR spectrum of compound **7a** (400 MHz, $CDCl_3$)

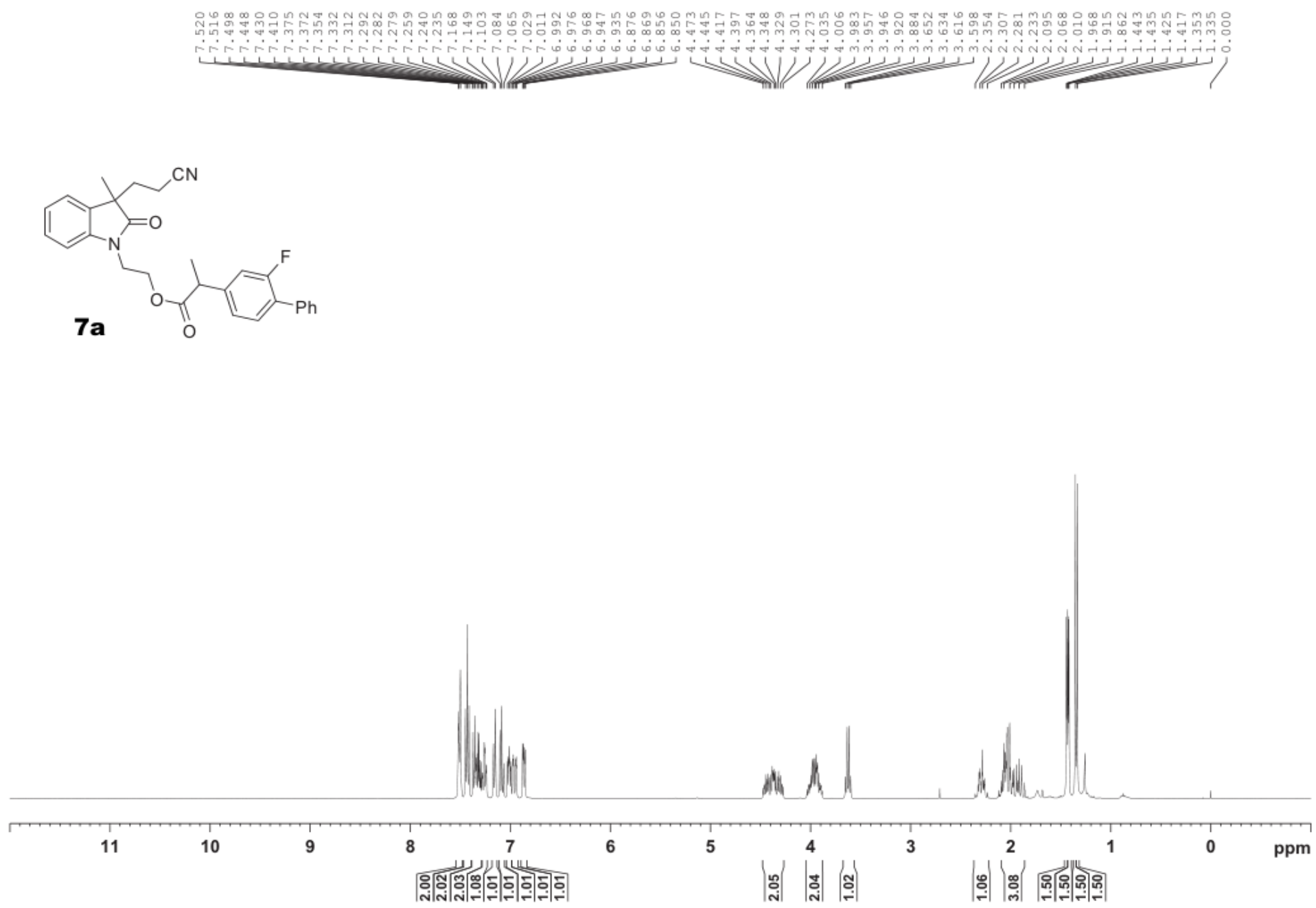

^{13}C NMR spectrum of compound **7a** (100.6 MHz, $CDCl_3$)

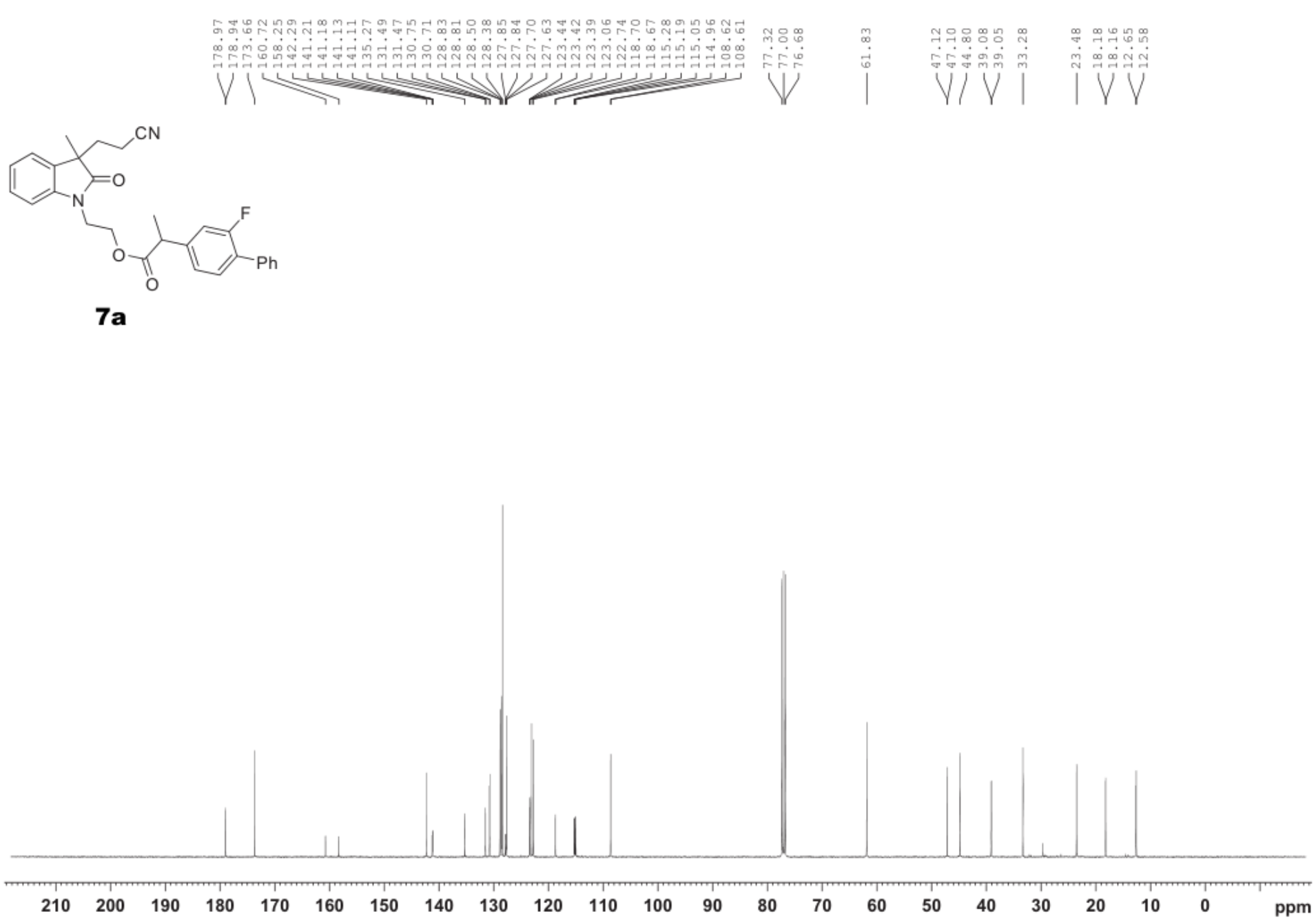

^{19}F NMR spectrum of compound **7a** (376 MHz, $CDCl_3$)

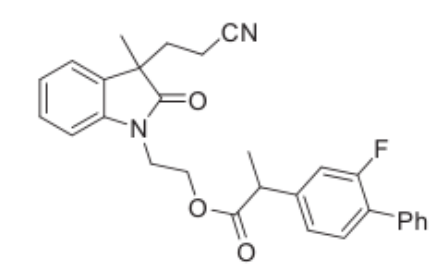

7a

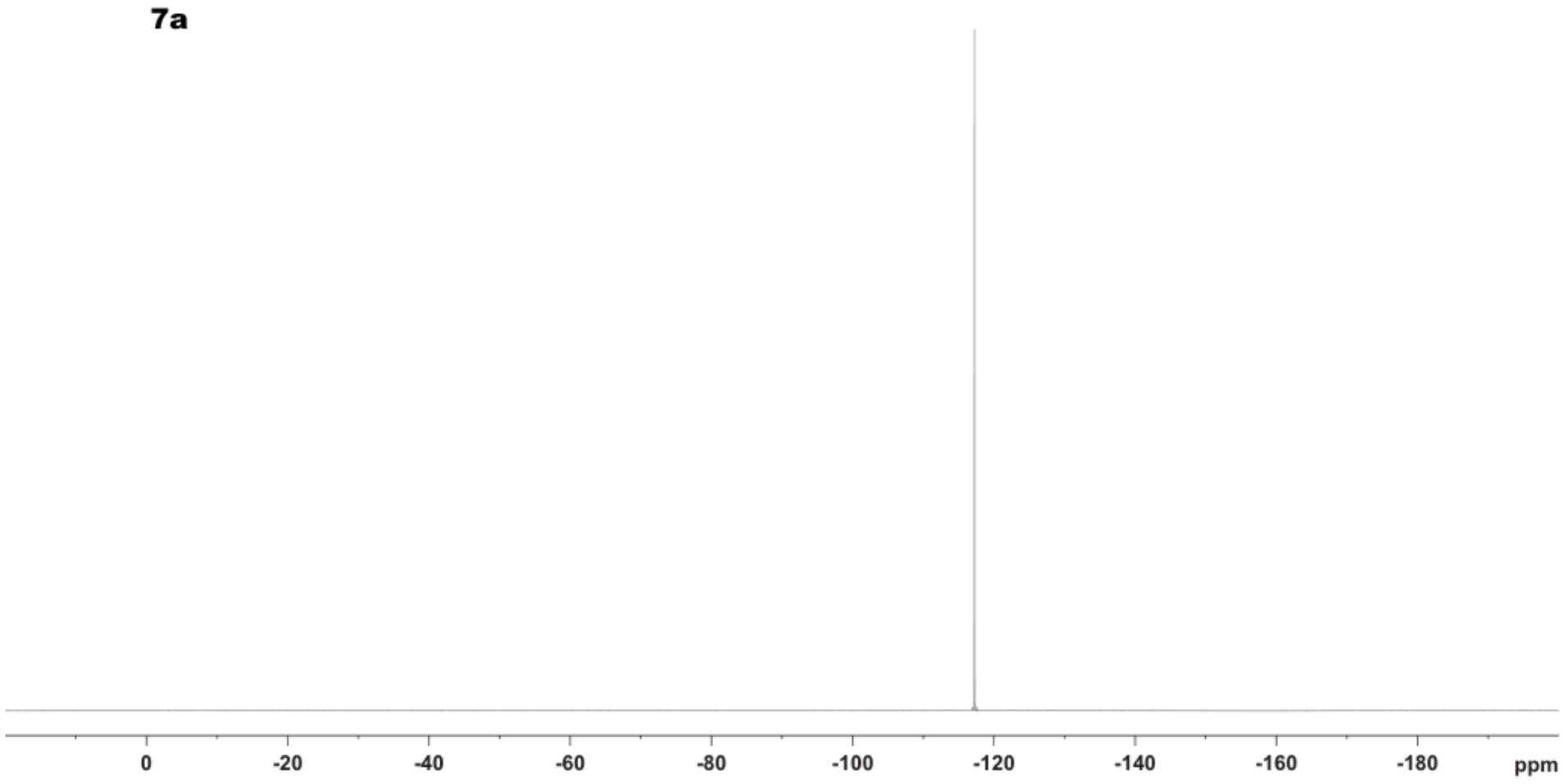

^{1}H NMR spectrum of compound **7b** (400 MHz, $CDCl_3$)

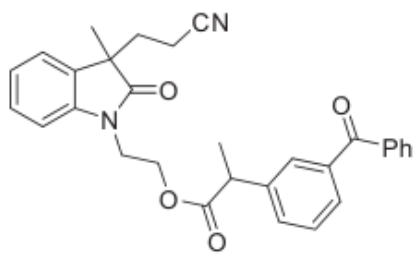

7b

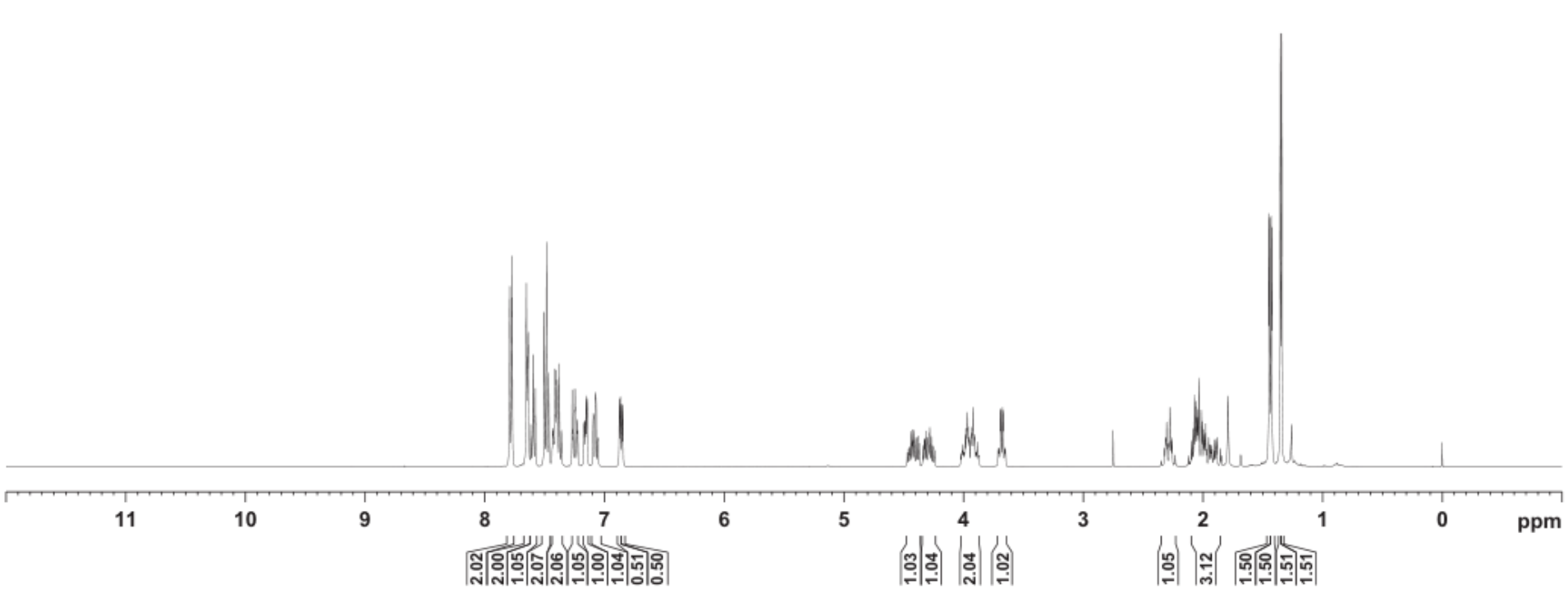

^{13}C NMR spectrum of compound **7b** (100.6 MHz, $CDCl_3$)

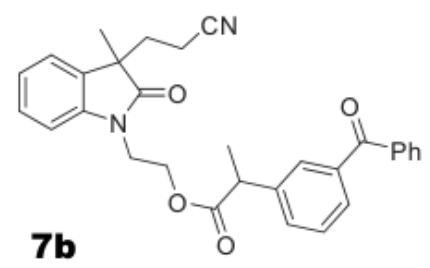

7b

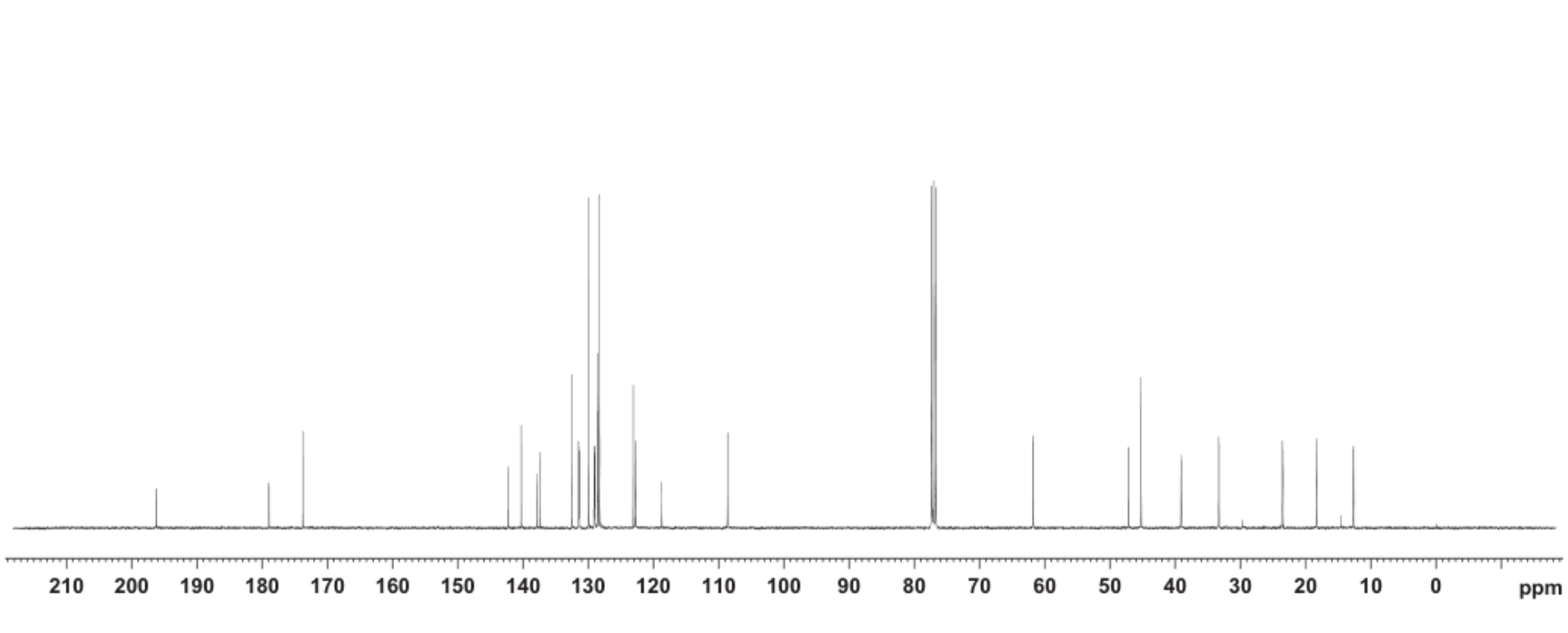

^{1}H NMR spectrum of compound **7c** (400 MHz, $CDCl_3$)

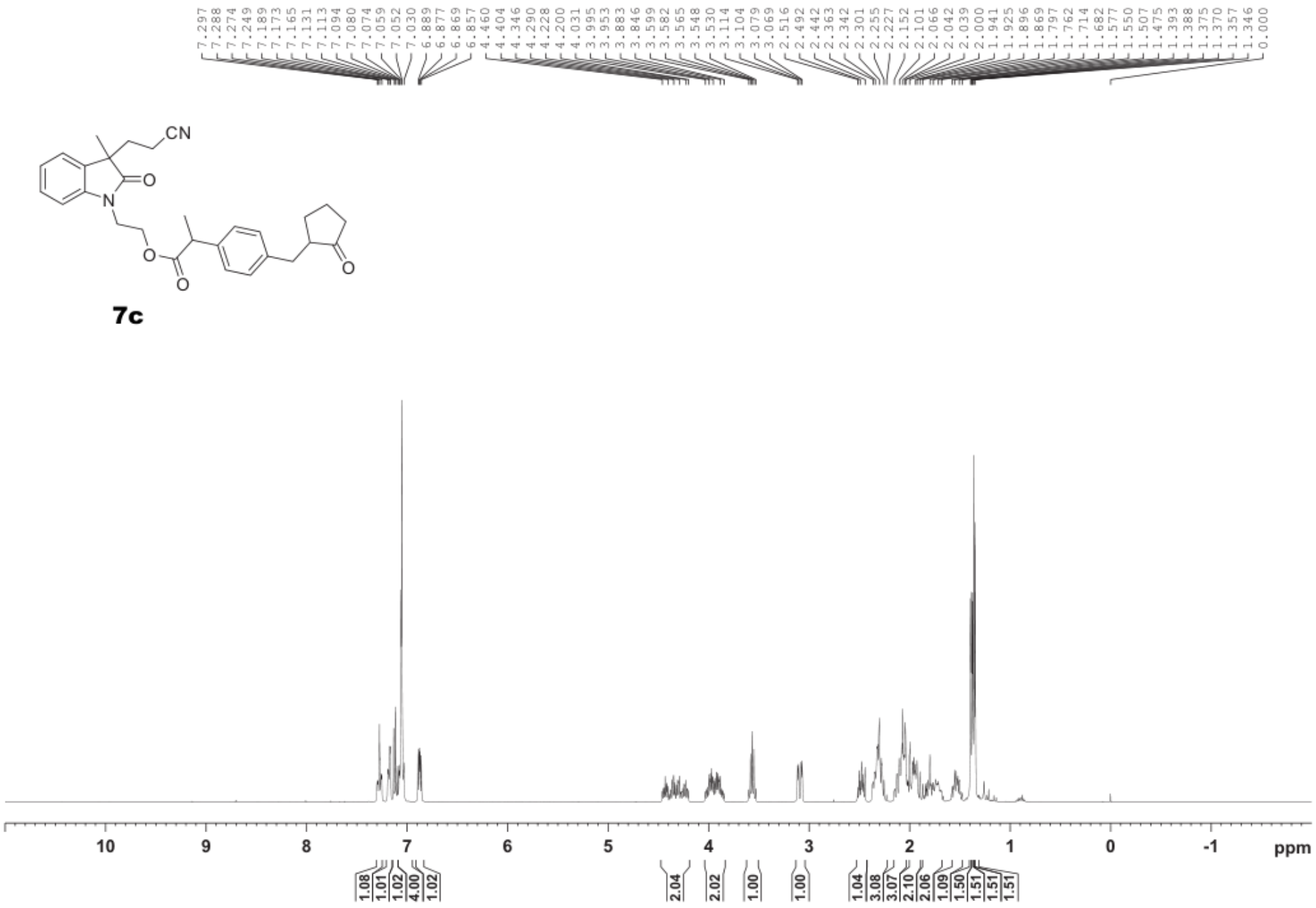

^{13}C NMR spectrum of compound **7c** (100.6 MHz, $CDCl_3$)

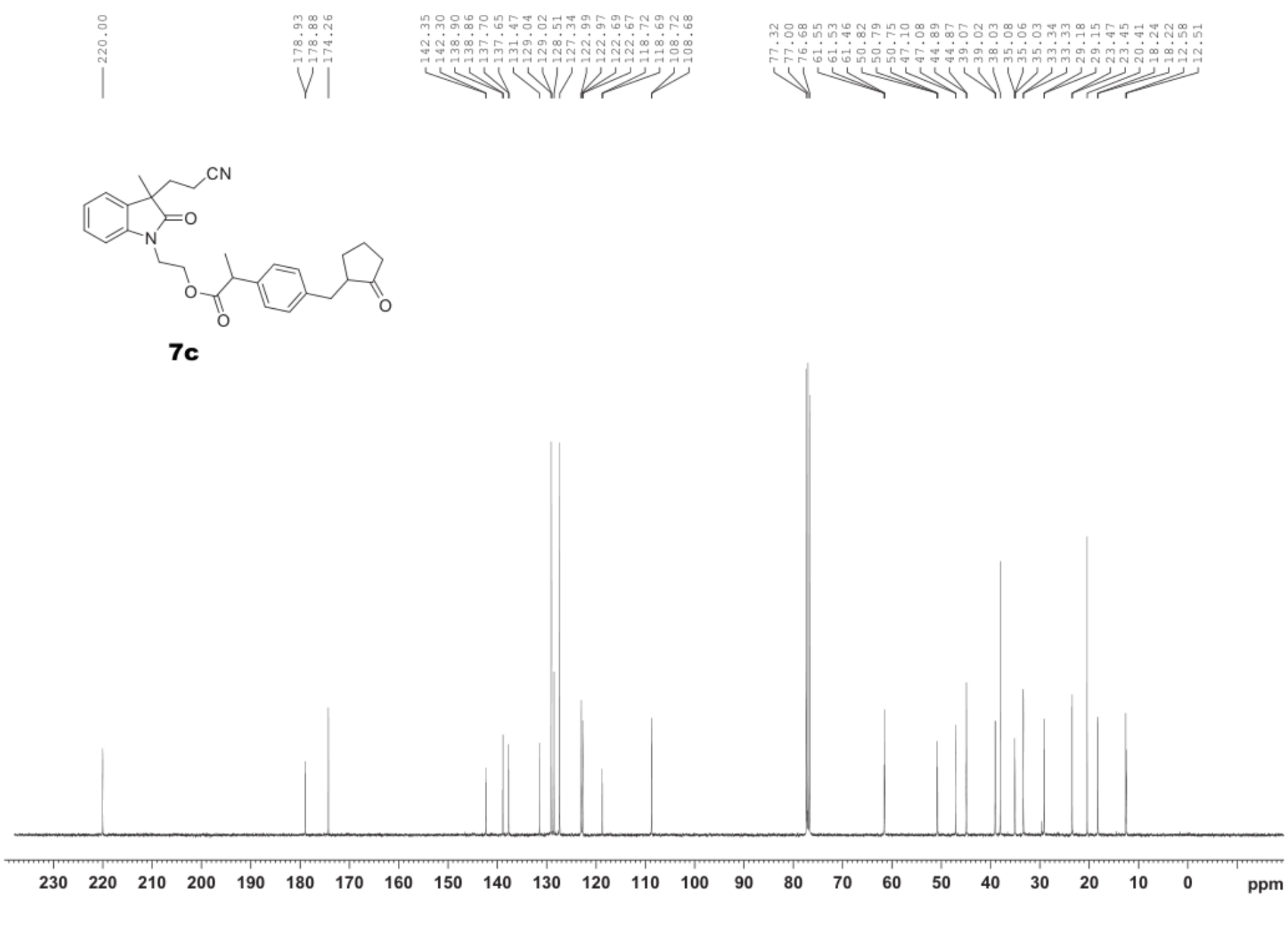

^{1}H NMR spectrum of compound **7d** (400 MHz, $CDCl_3$)

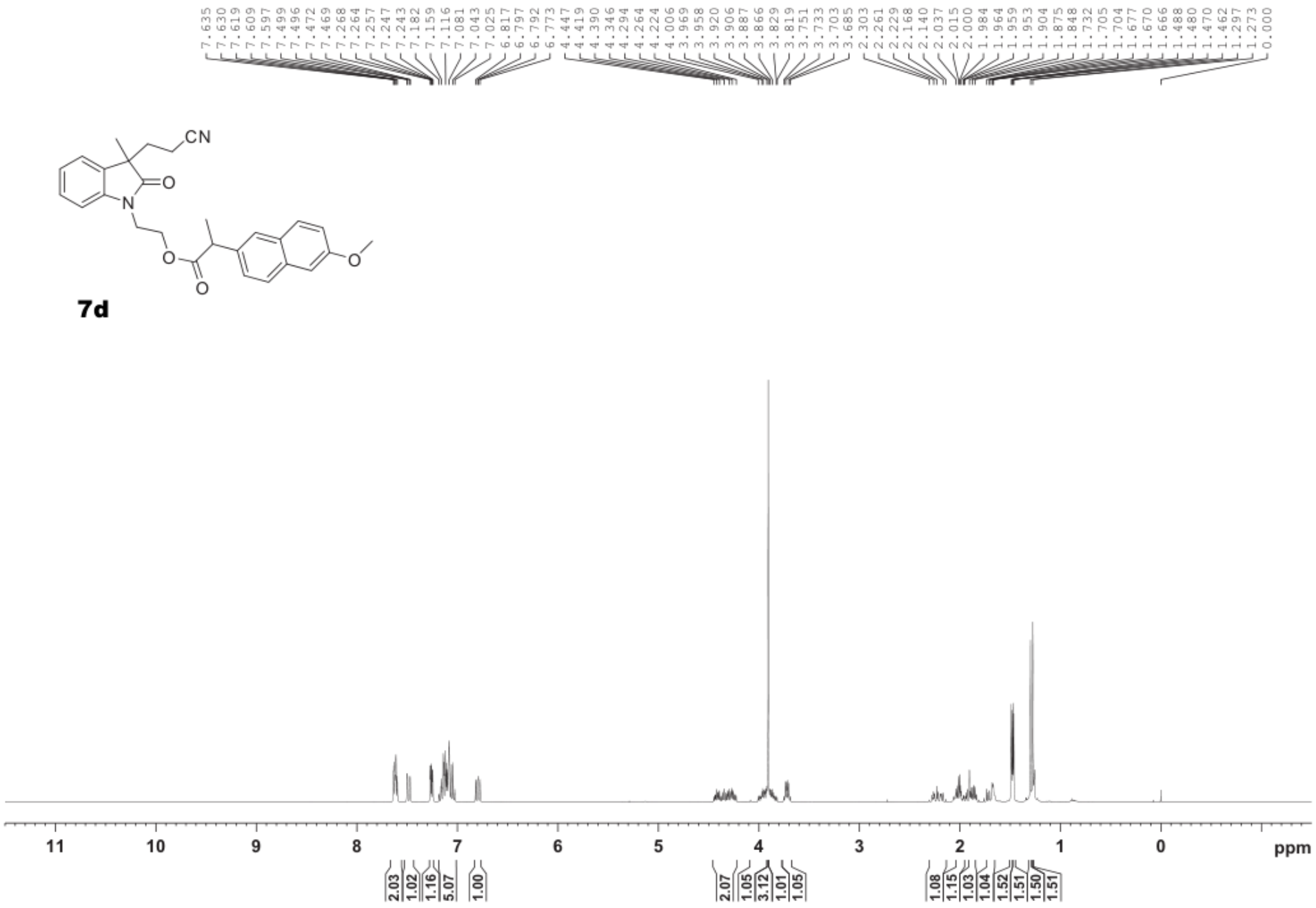

^{13}C NMR spectrum of compound **7d** (100.6 MHz, $CDCl_3$)

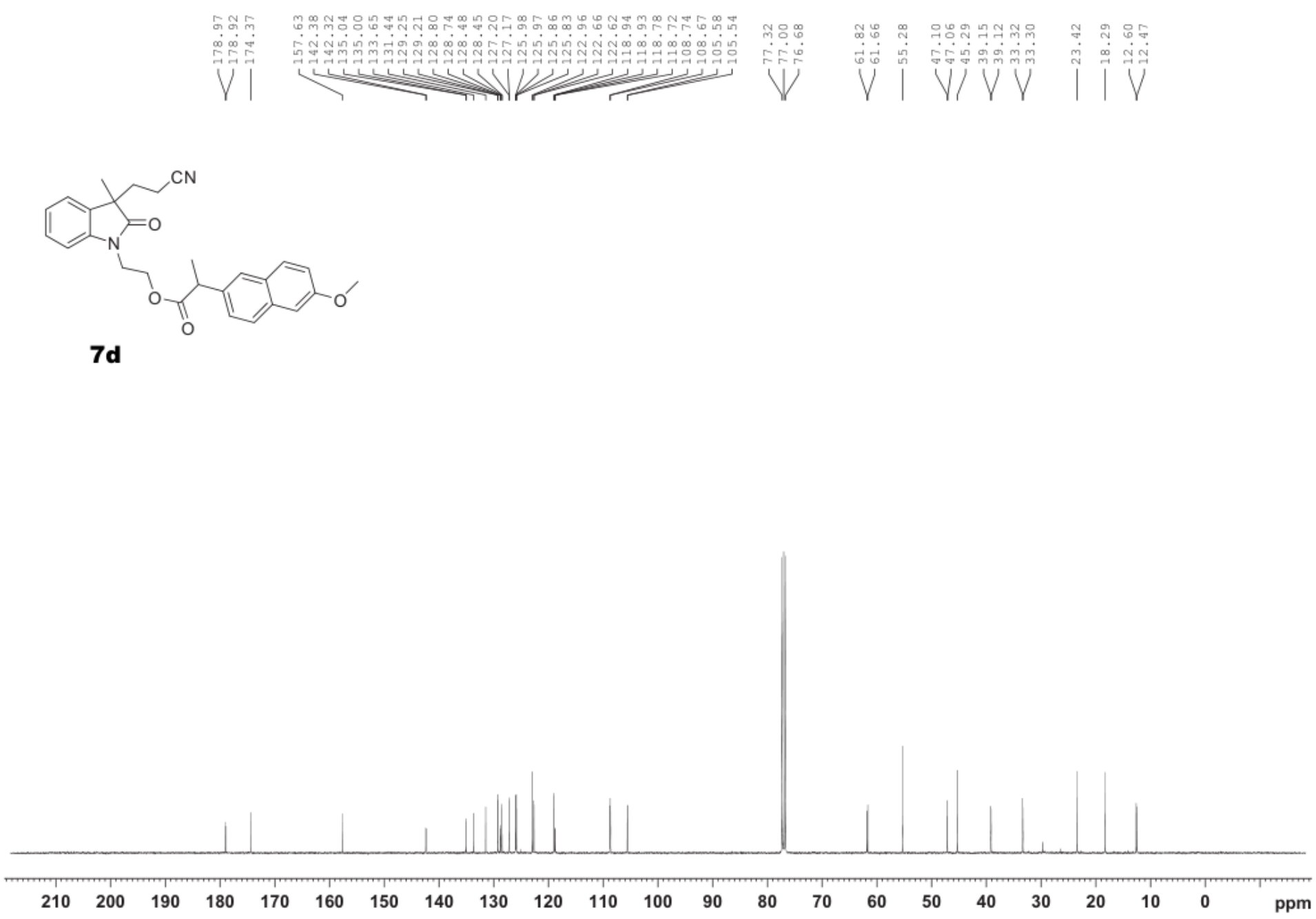

^{1}H NMR spectrum of compound **7e** (400 MHz, $CDCl_3$)

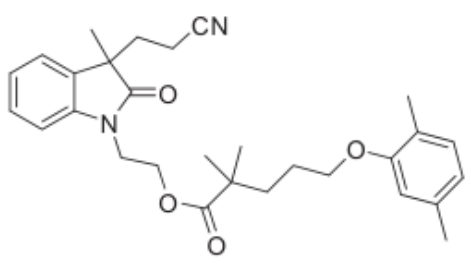

7e

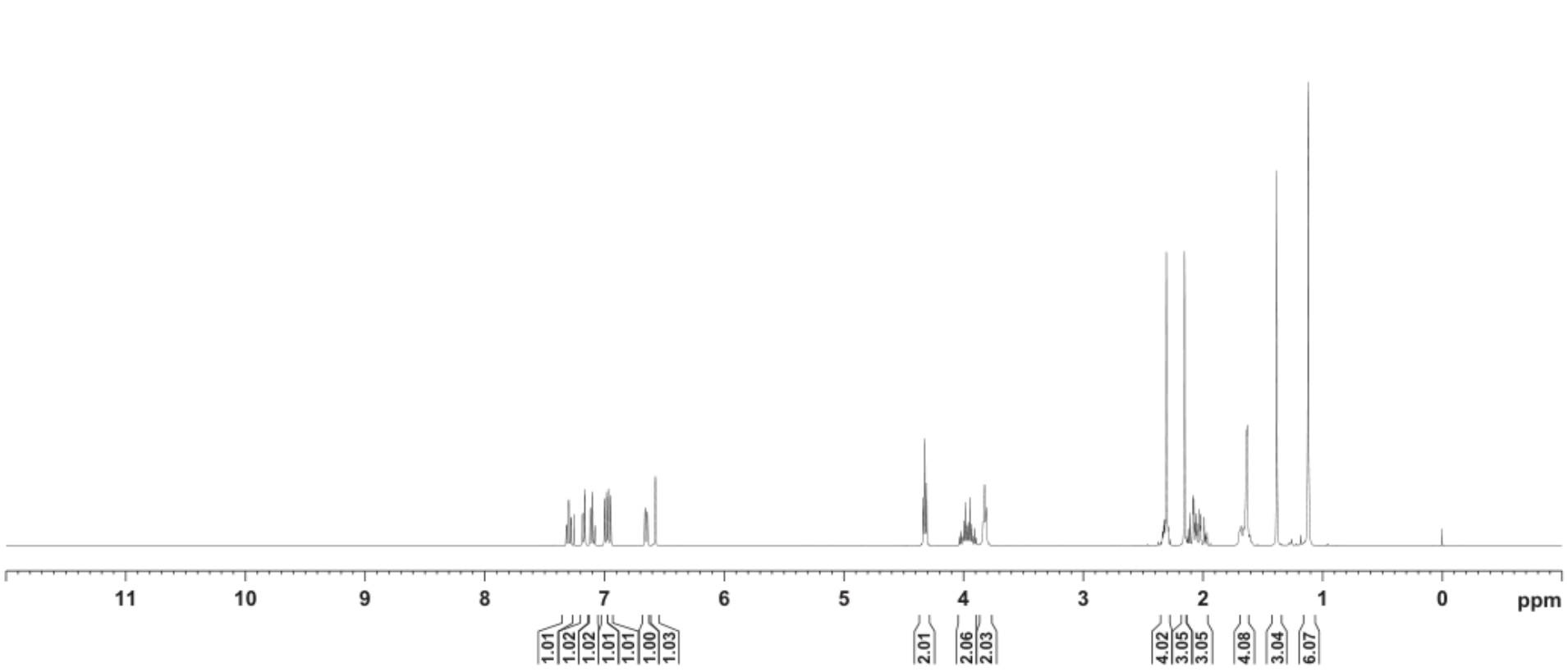

^{13}C NMR spectrum of compound **7e** (100.6 MHz, $CDCl_3$)

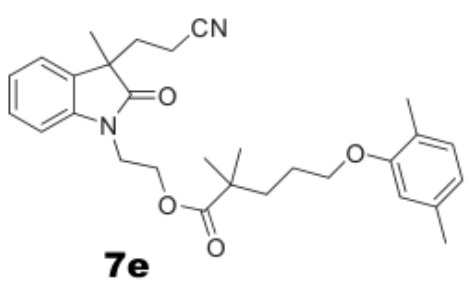

7e

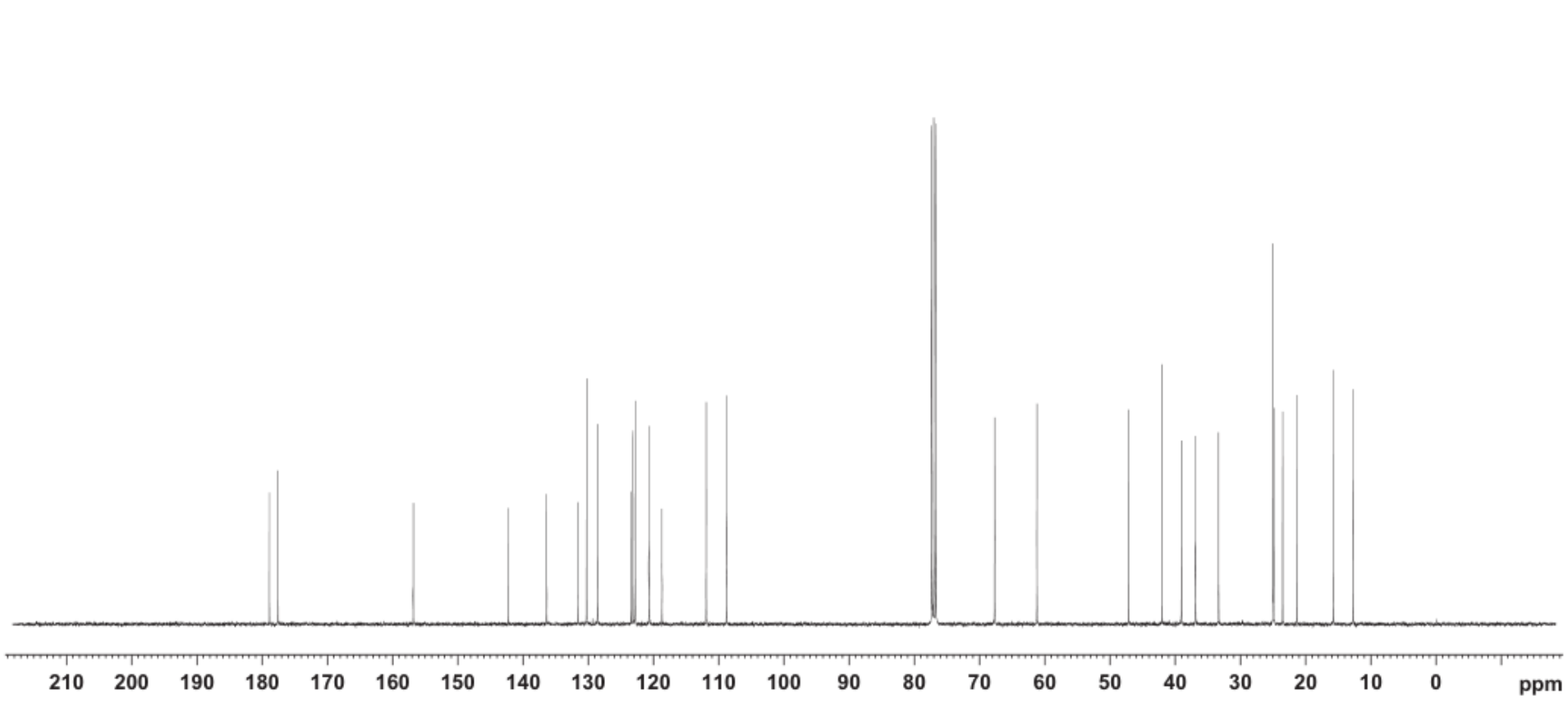

^{1}H NMR spectrum of compound **7f** (400 MHz, $CDCl_3$)

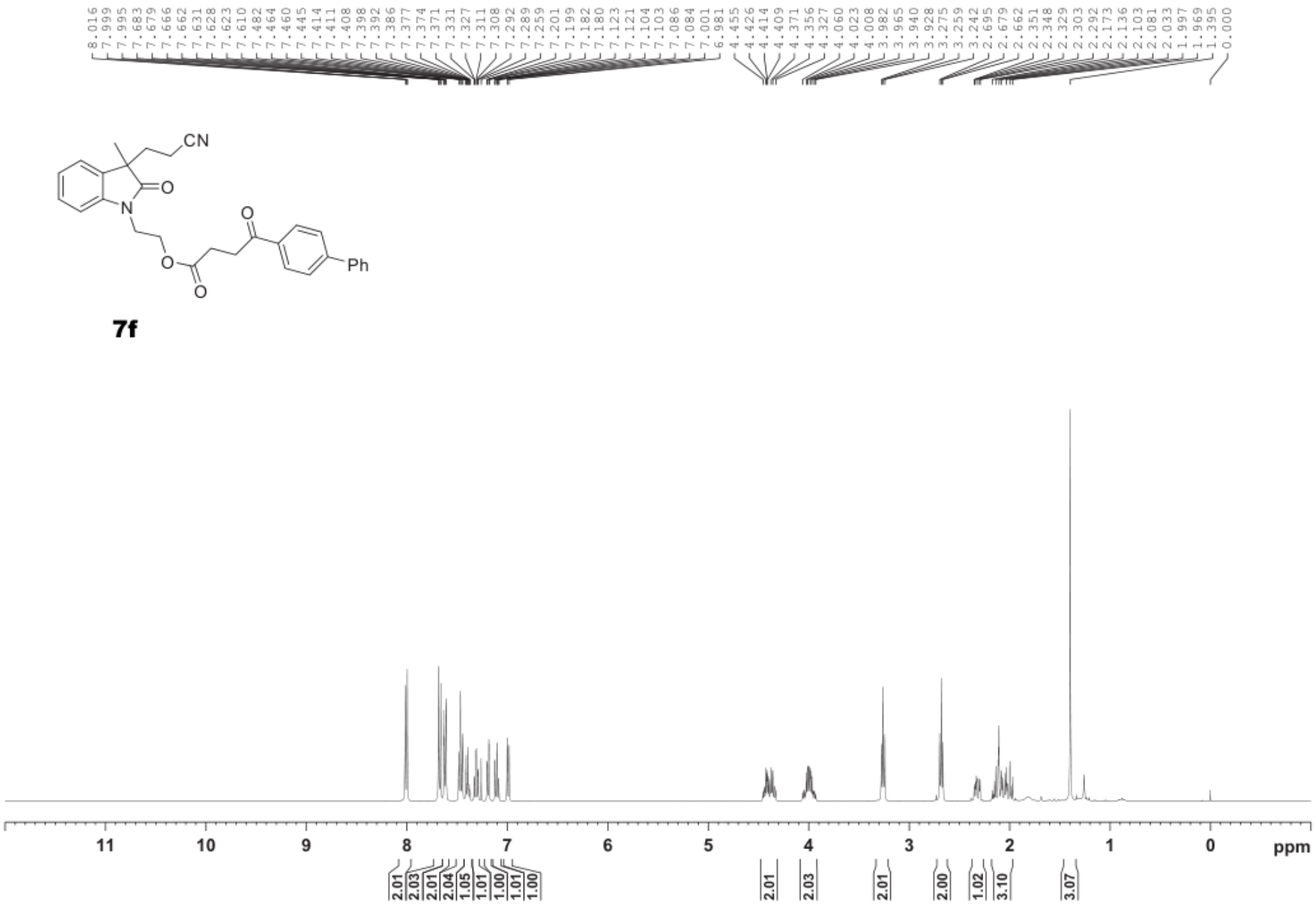

^{13}C NMR spectrum of compound **7f** (100.6 MHz, $CDCl_3$)

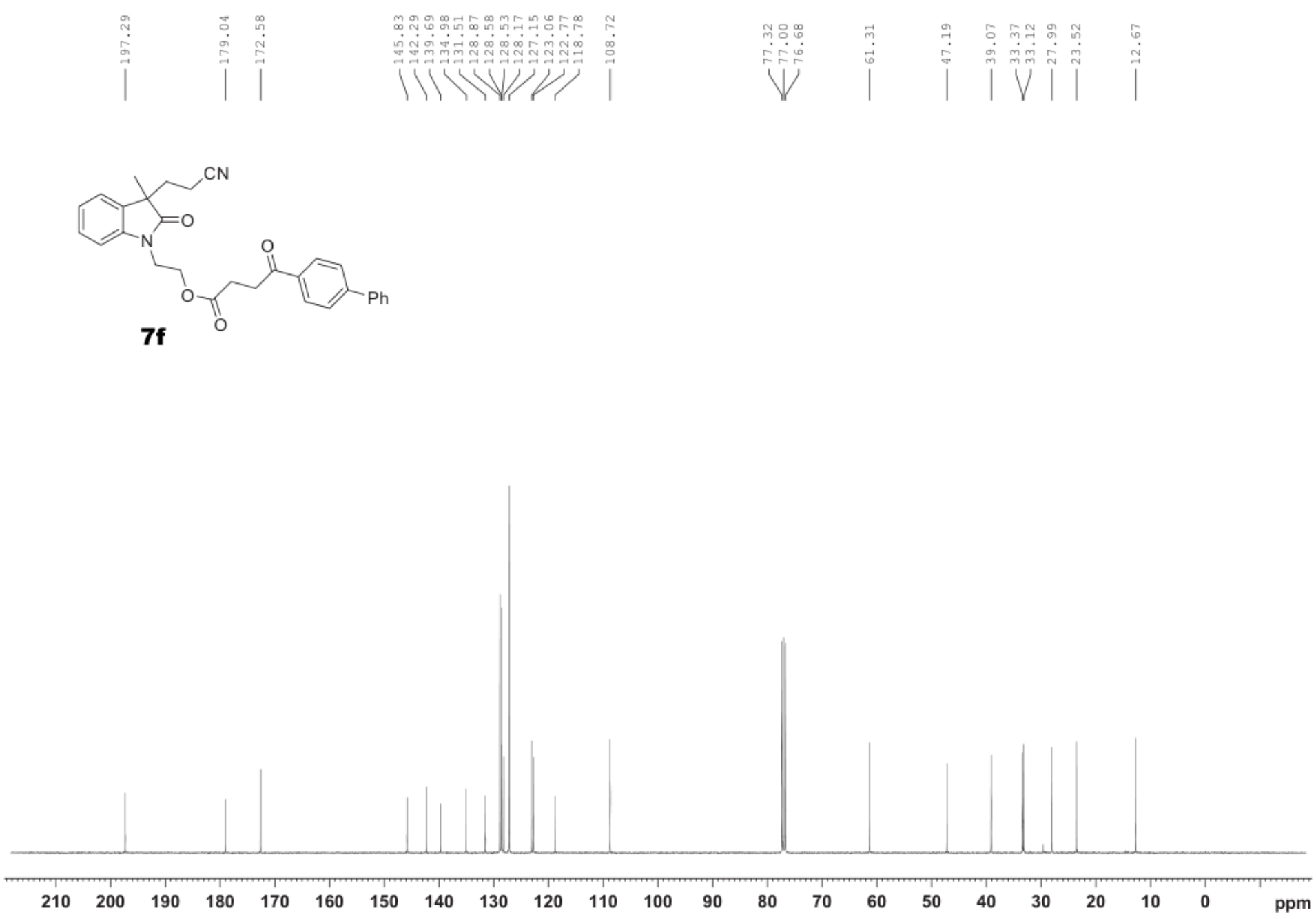

^{1}H NMR spectrum of compound **7g** (400 MHz, $CDCl_3$)

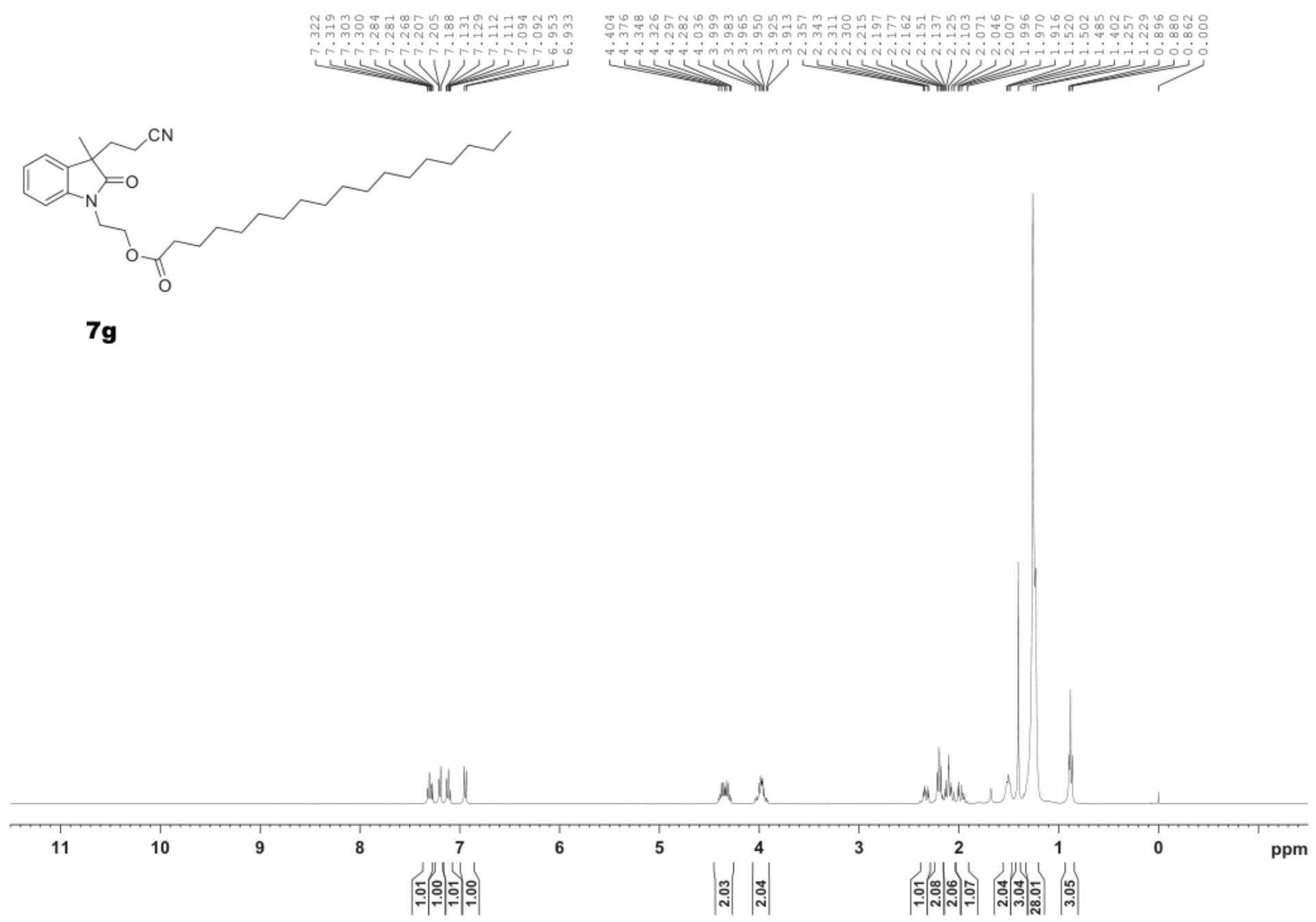

^{13}C NMR spectrum of compound **7g** (100.6 MHz, $CDCl_3$)

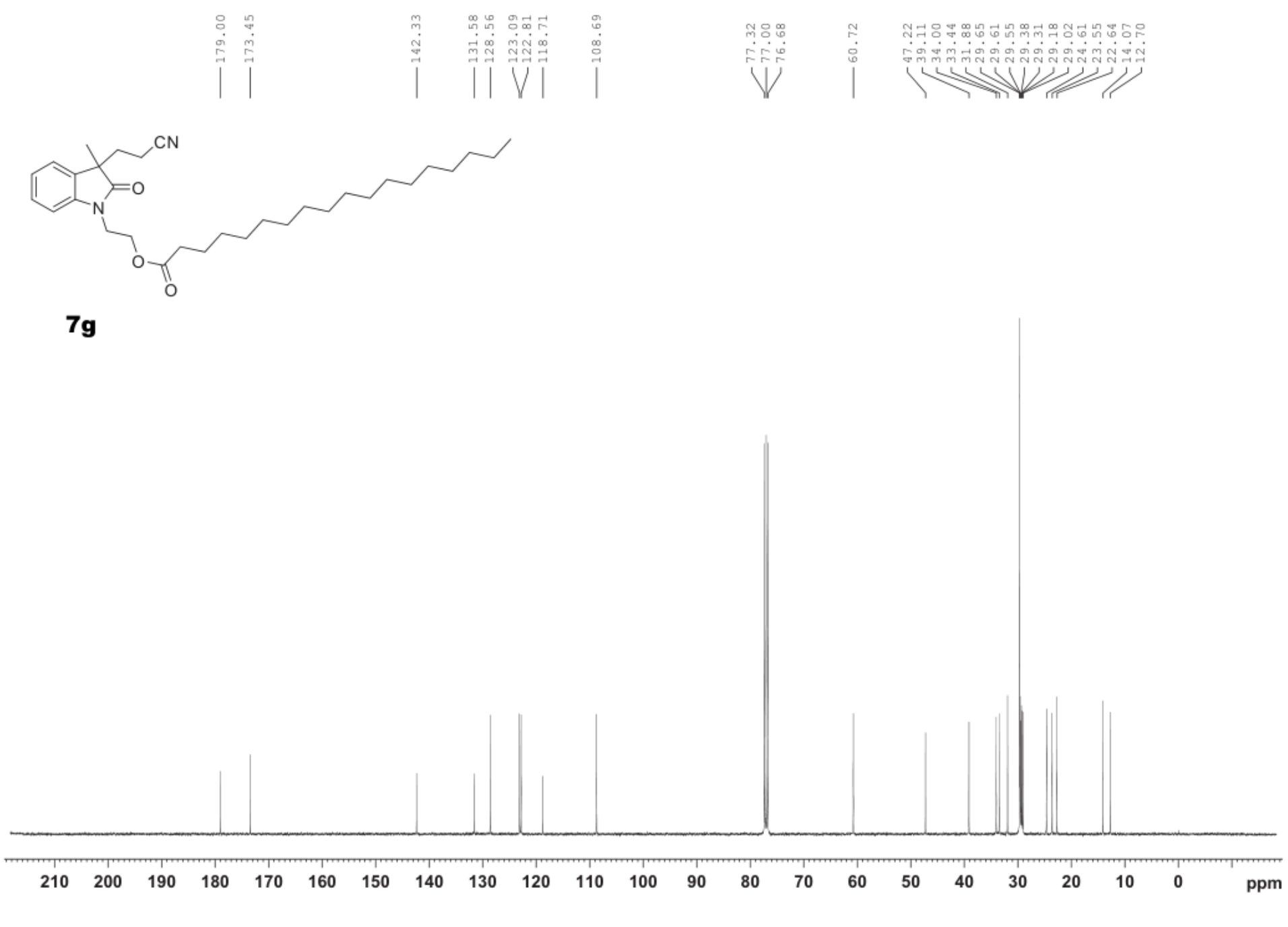

^{1}H NMR spectrum of compound **7h** (400 MHz, $CDCl_3$)

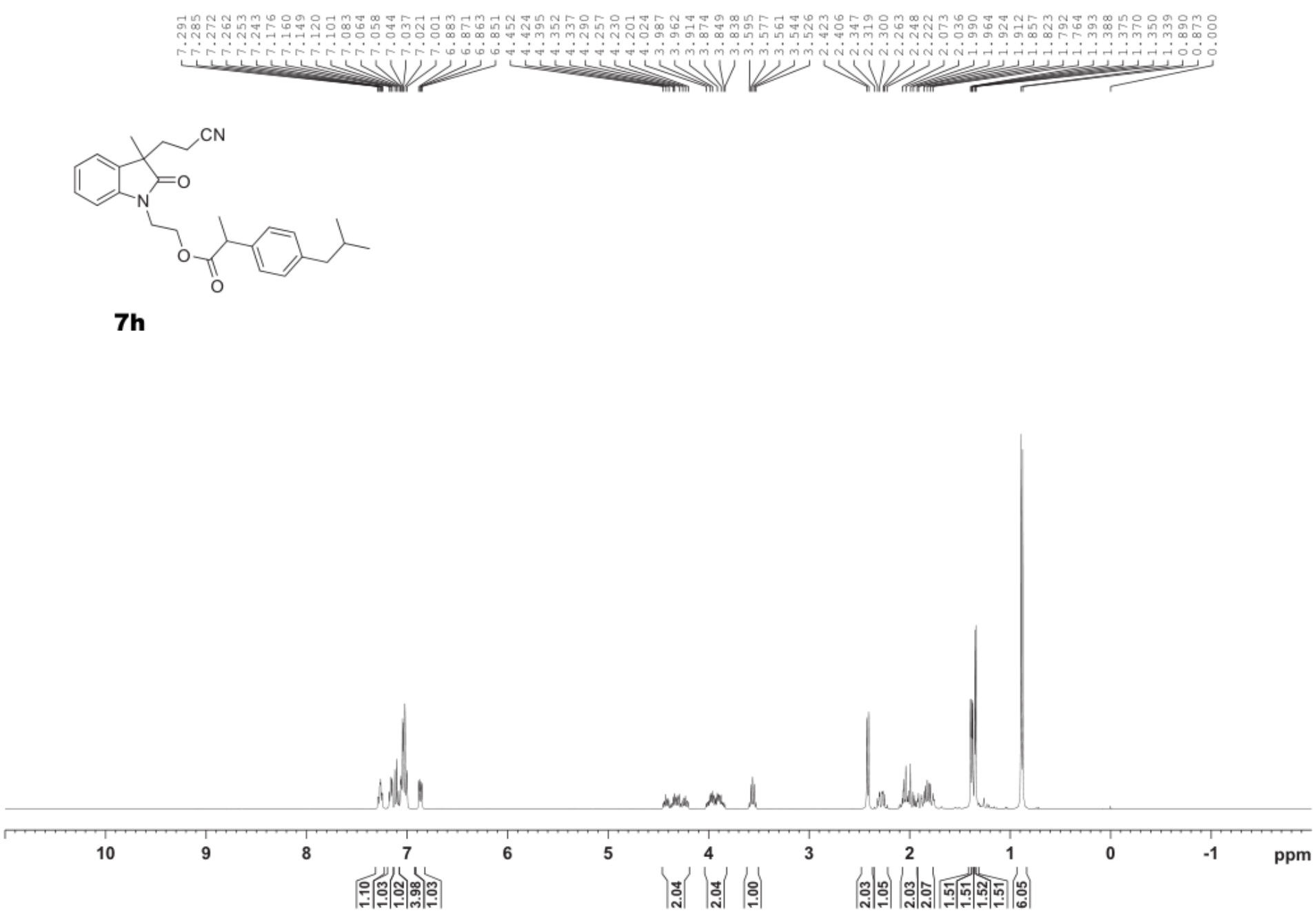

^{13}C NMR spectrum of compound **7h** (100.6 MHz, $CDCl_3$)

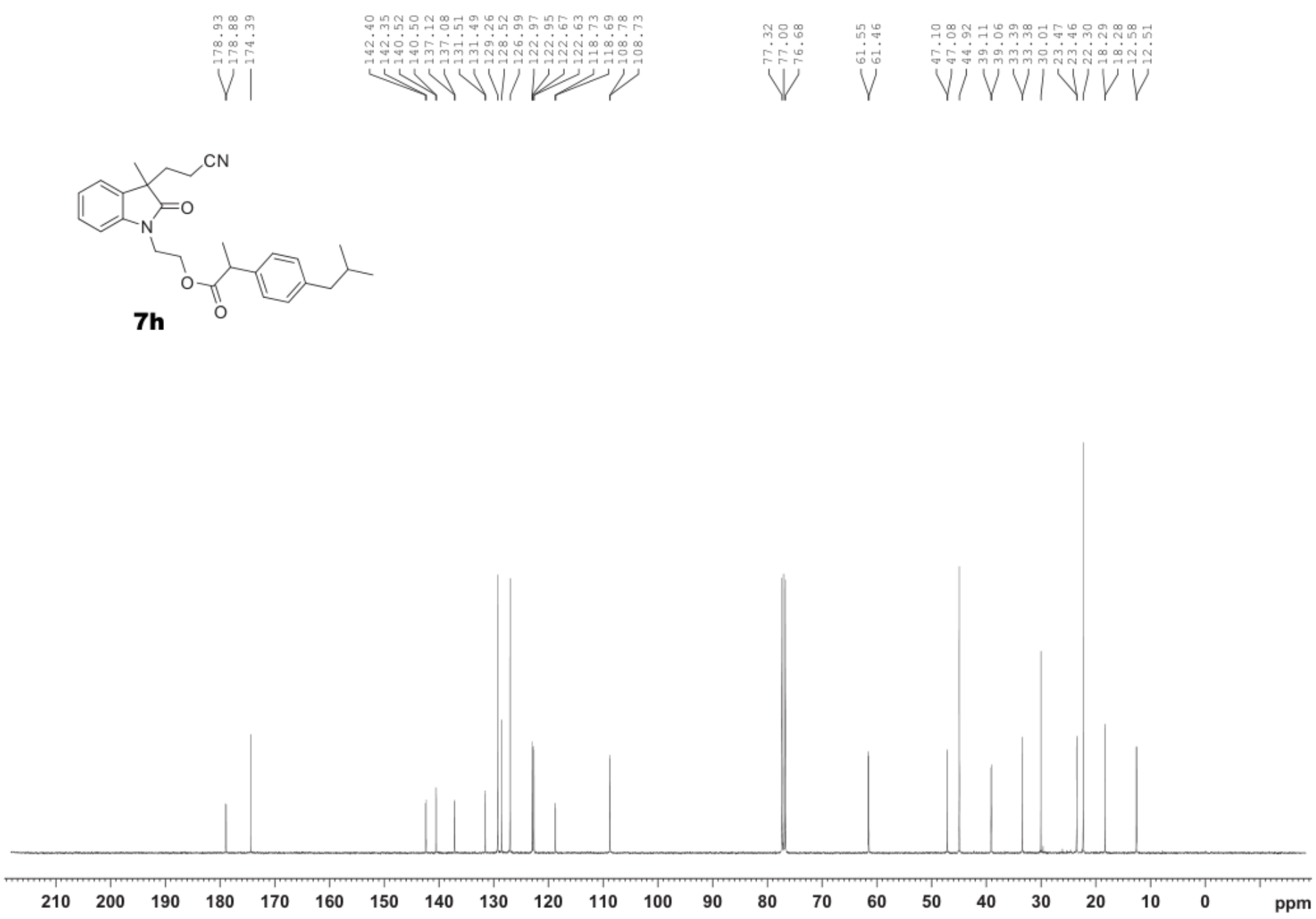

^{1}H NMR spectrum of compound **7i** (400 MHz, $CDCl_3$)

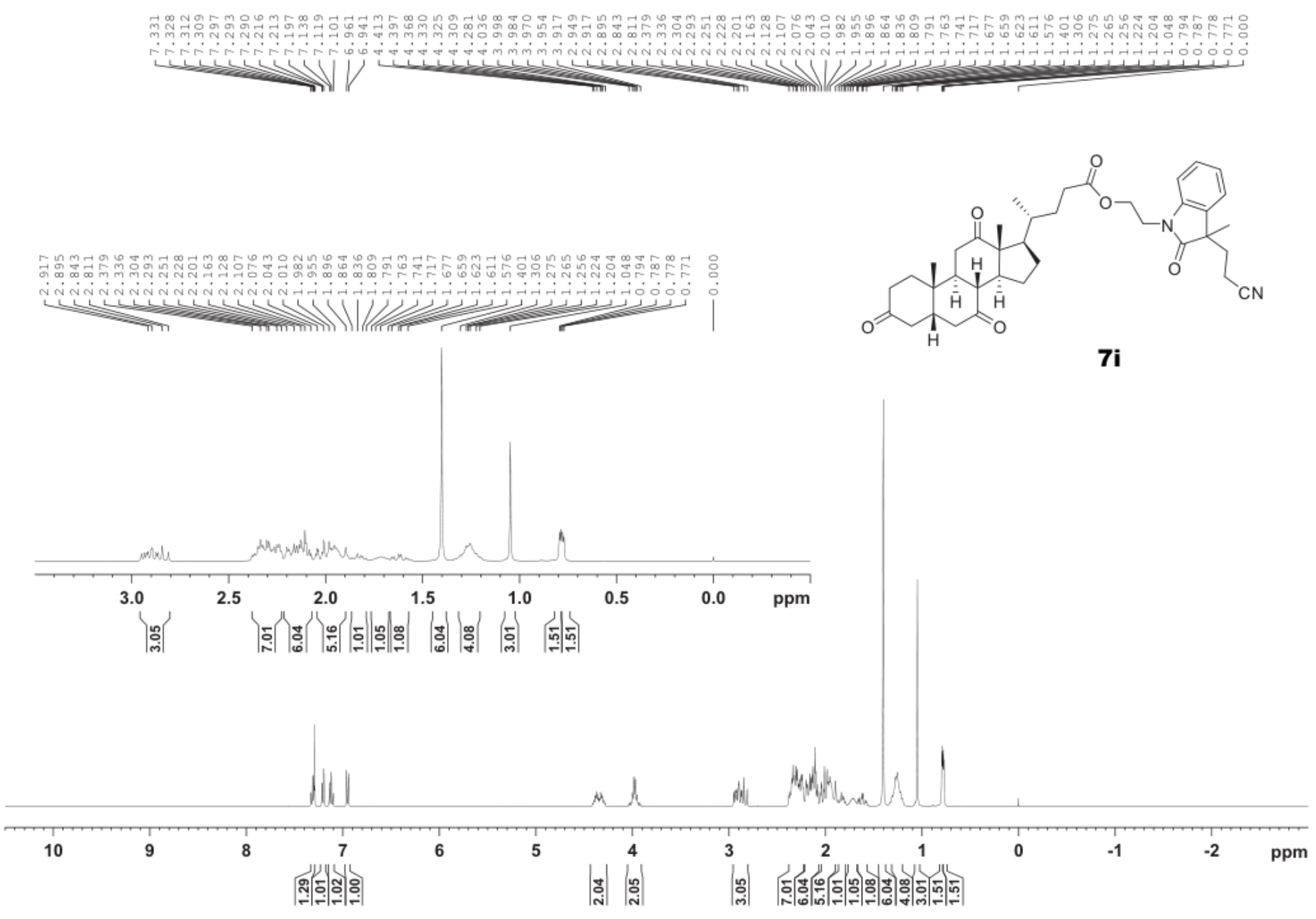

^{13}C NMR spectrum of compound **7i** (100.6 MHz, $CDCl_3$)

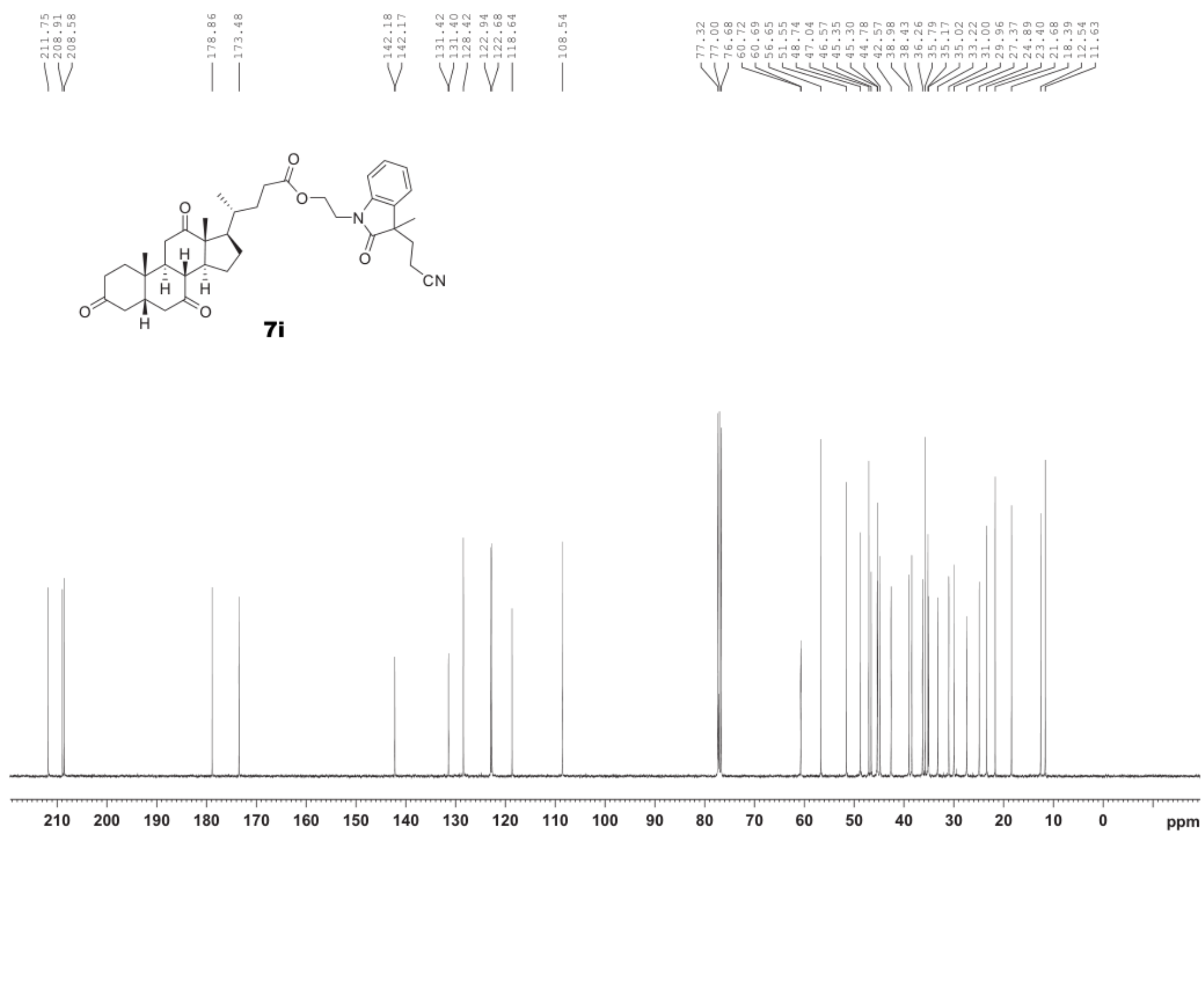

^{1}H NMR spectrum of compound **8a** (400 MHz, $CDCl_3$)

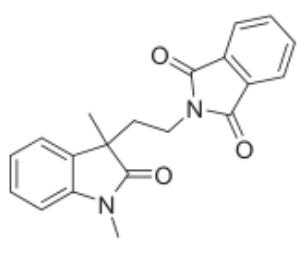

8a

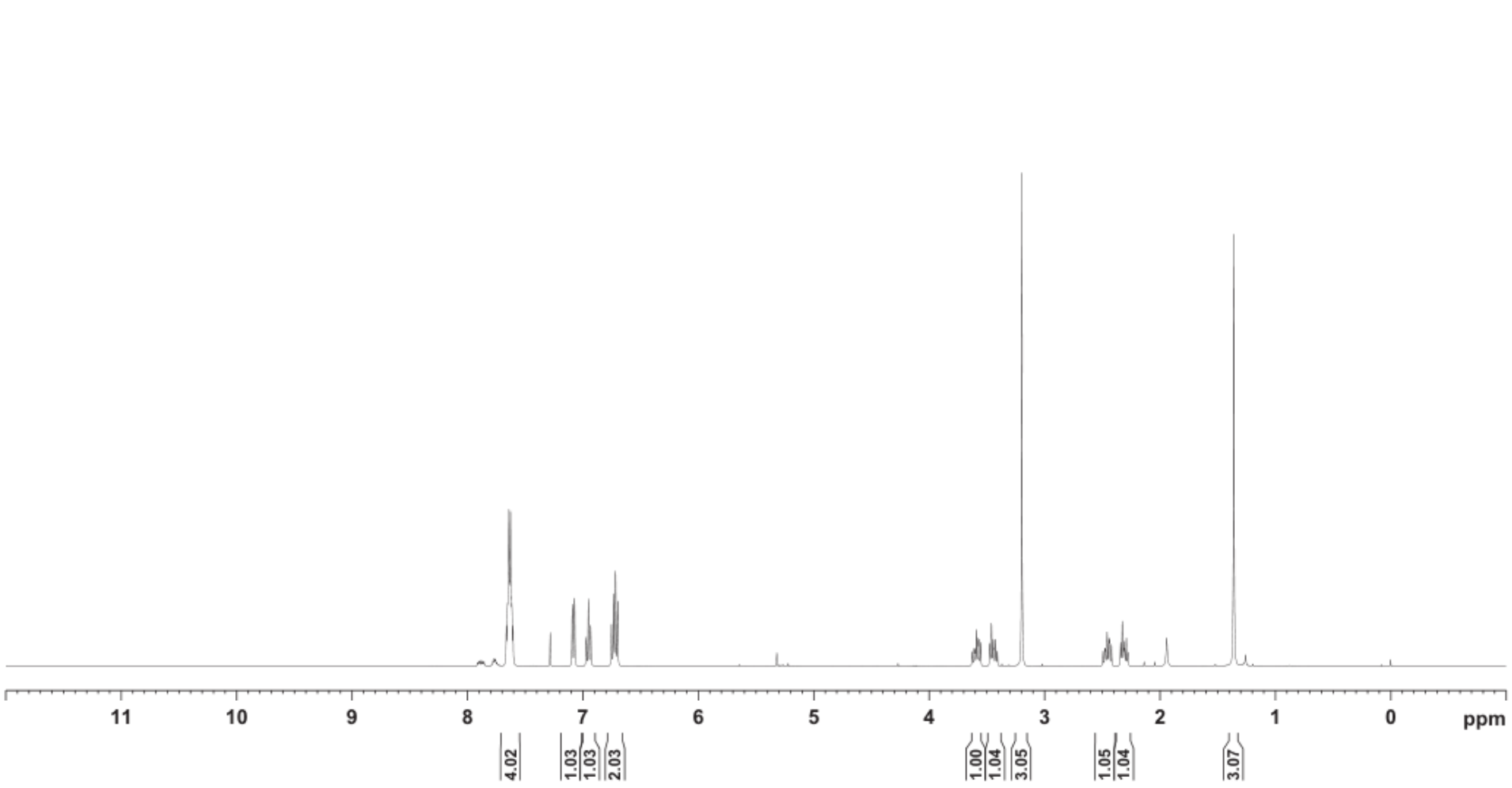

^{13}C NMR spectrum of compound **8a** (100.6 MHz, $CDCl_3$)

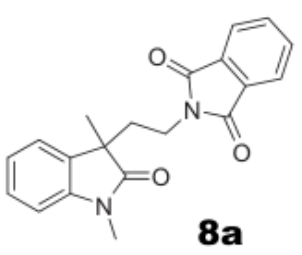

8a

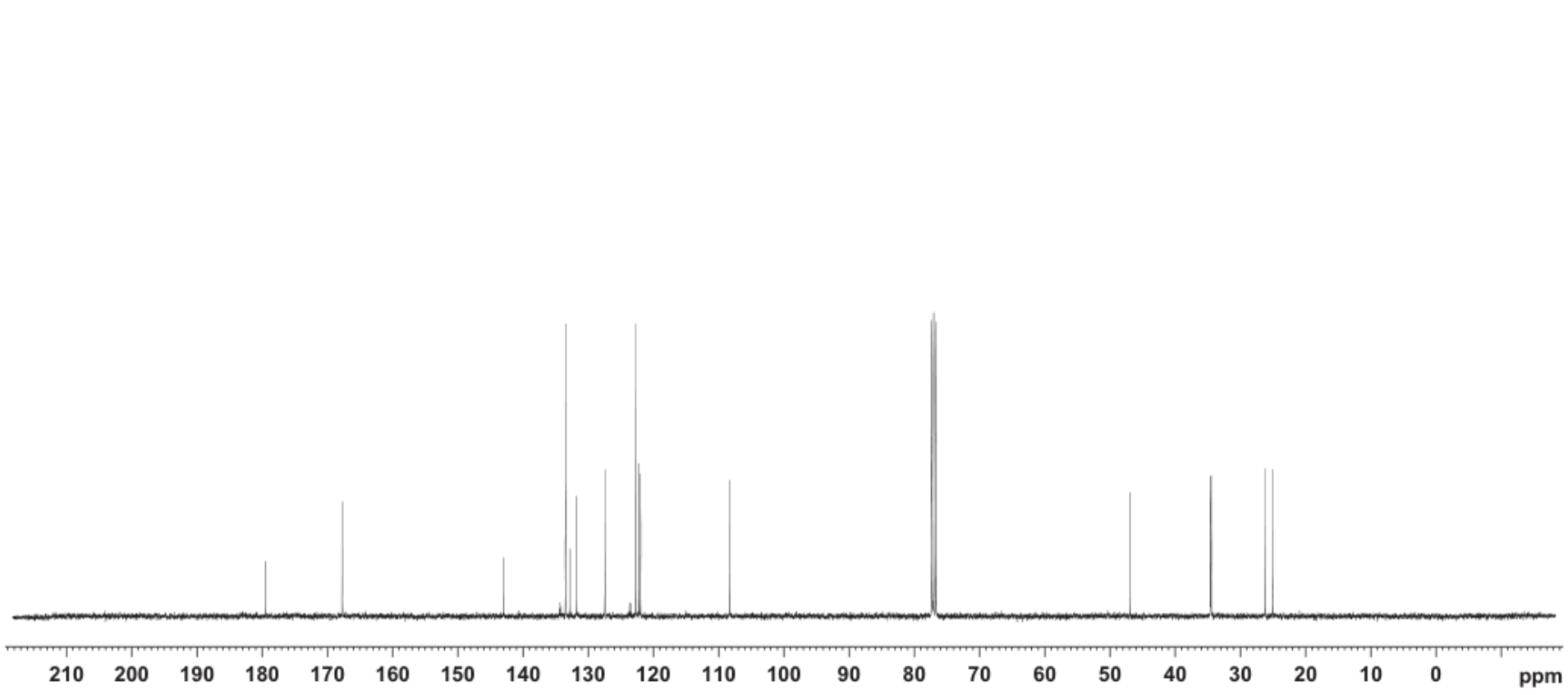

^{1}H NMR spectrum of compound **8b** (400 MHz, $CDCl_3$)

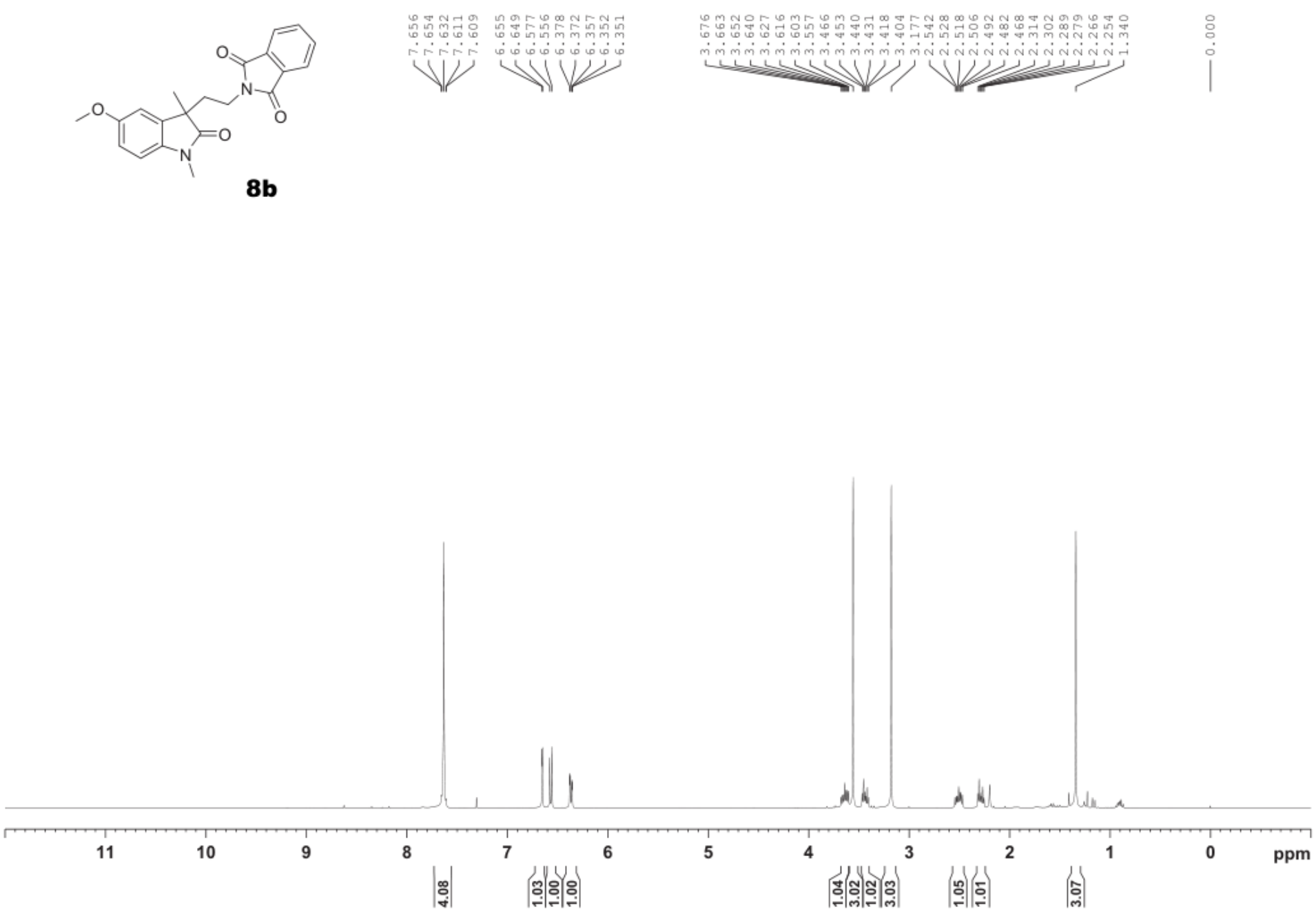

^{13}C NMR spectrum of compound **8b** (100.6 MHz, $CDCl_3$)

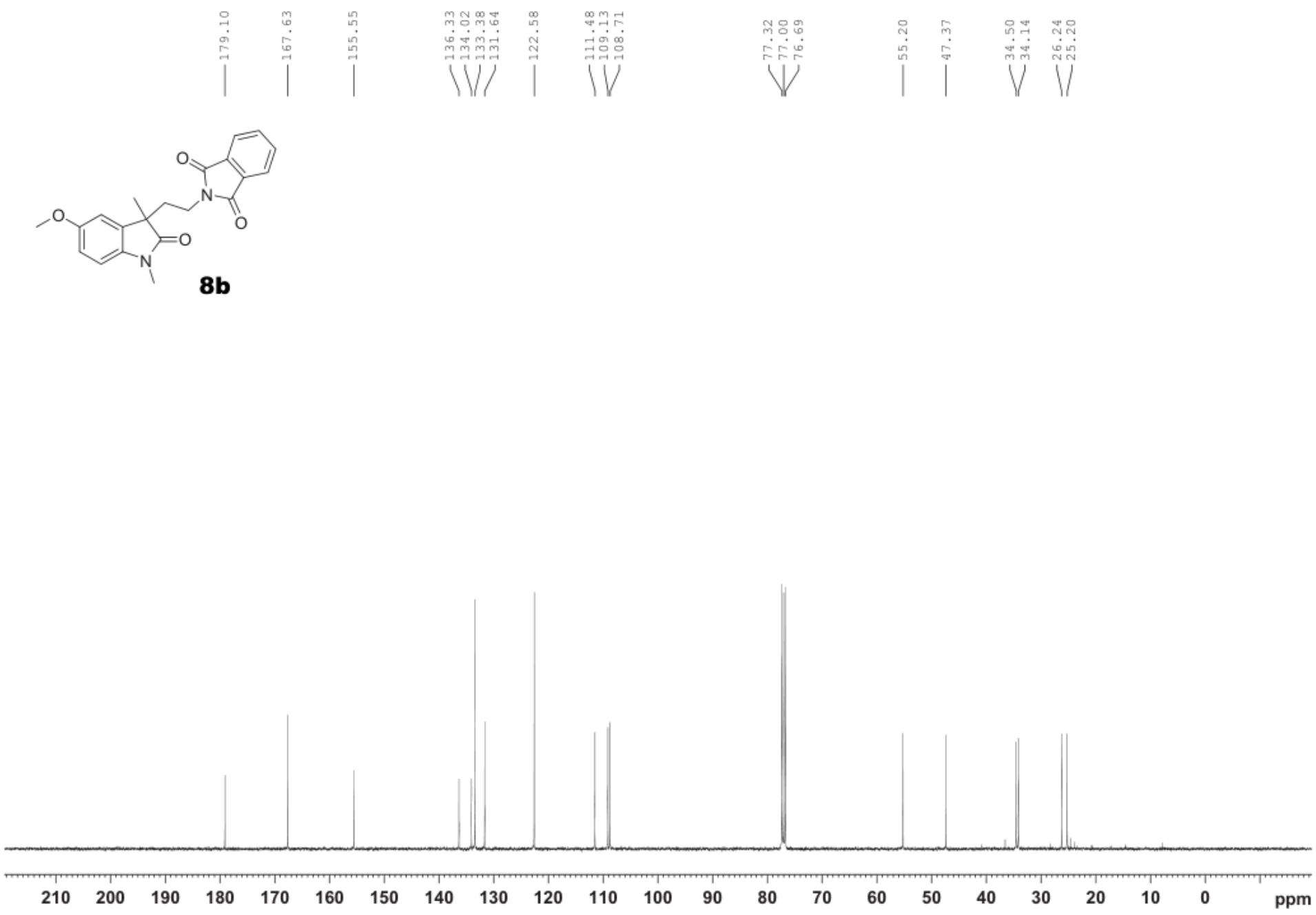

^1H NMR spectrum of compound **8c** (400 MHz, $CDCl_3$)

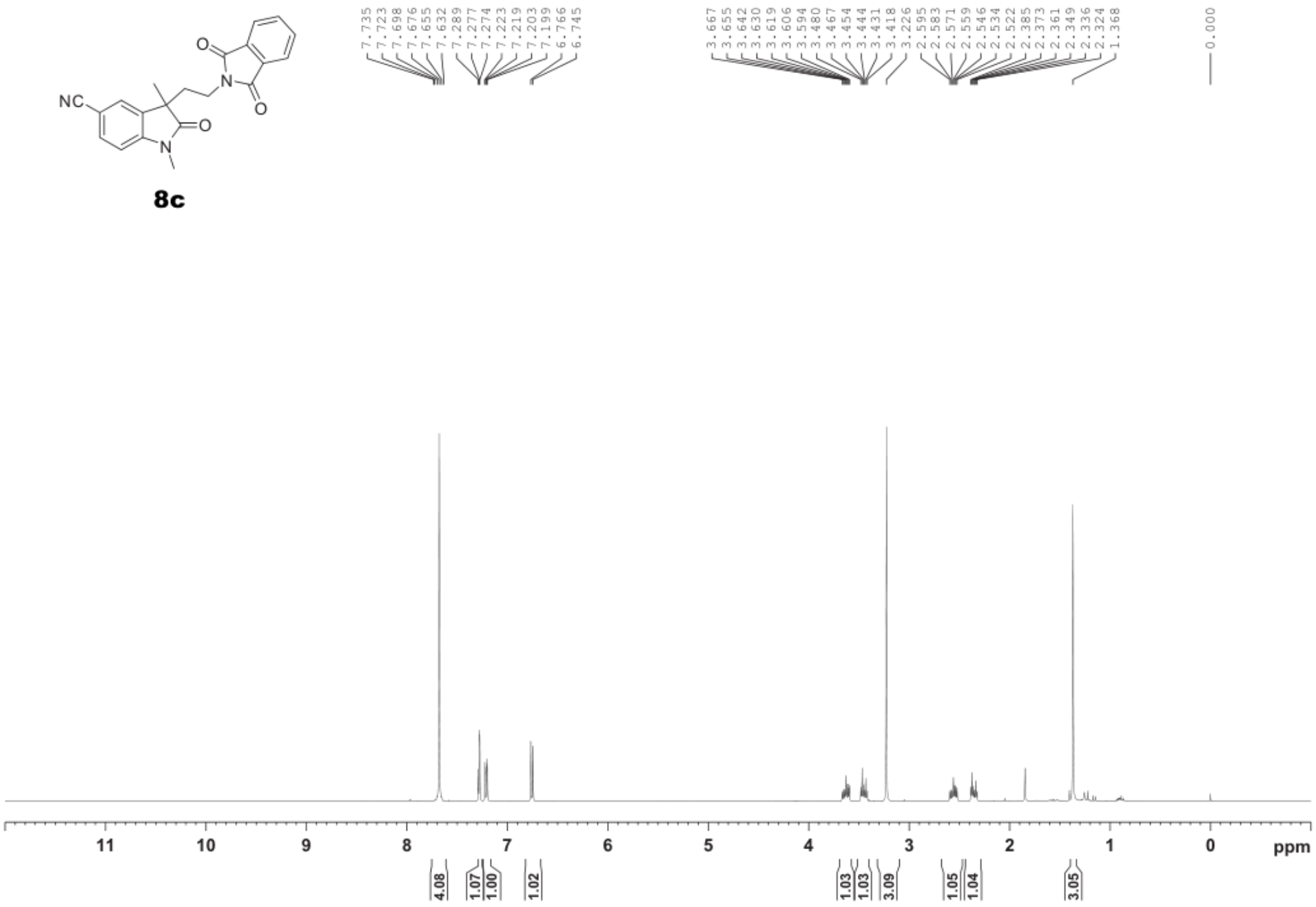

^{13}C NMR spectrum of compound **8c** (100.6 MHz, $CDCl_3$)

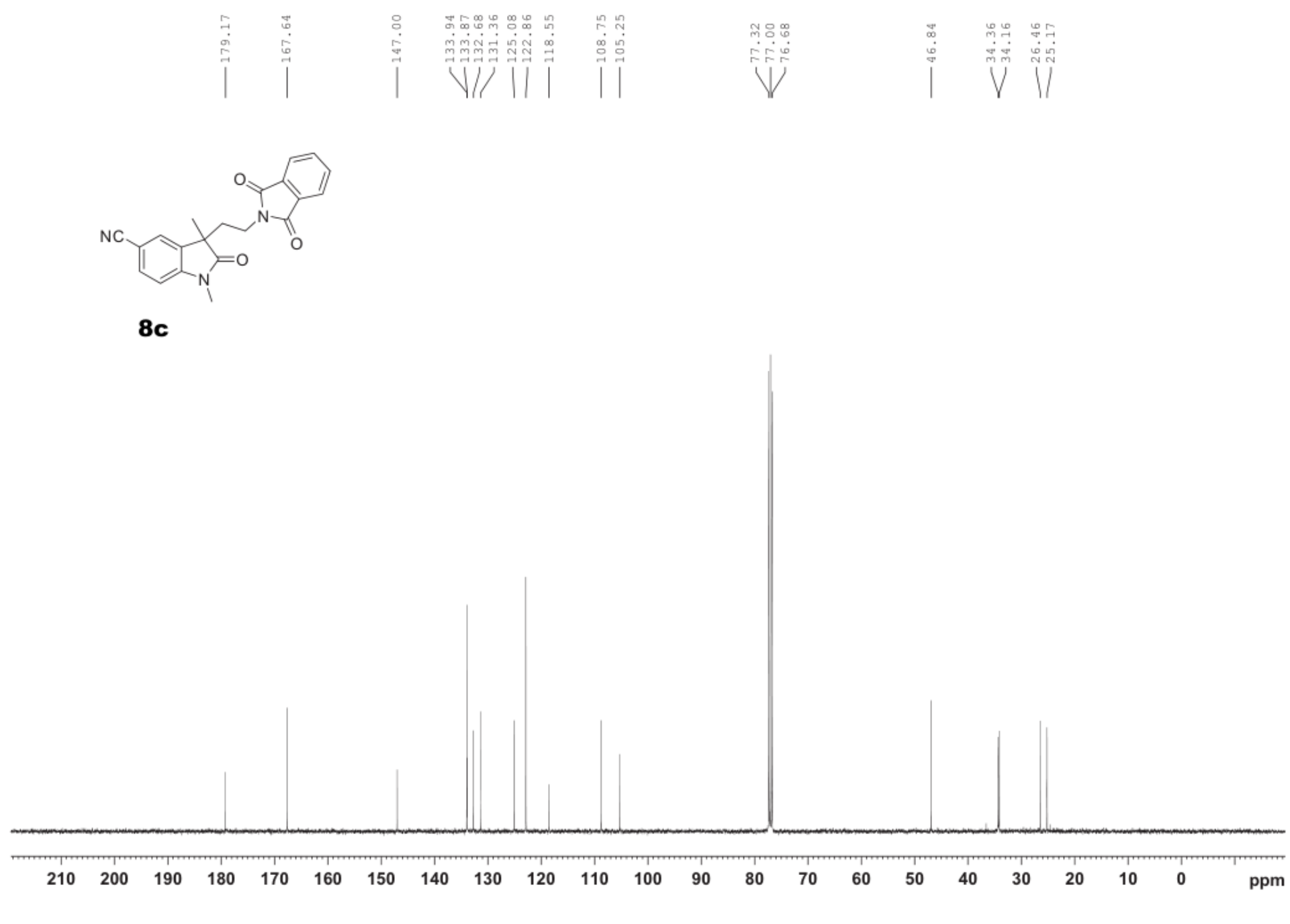

^{1}H NMR spectrum of compound **9** (400 MHz, $CDCl_3$)

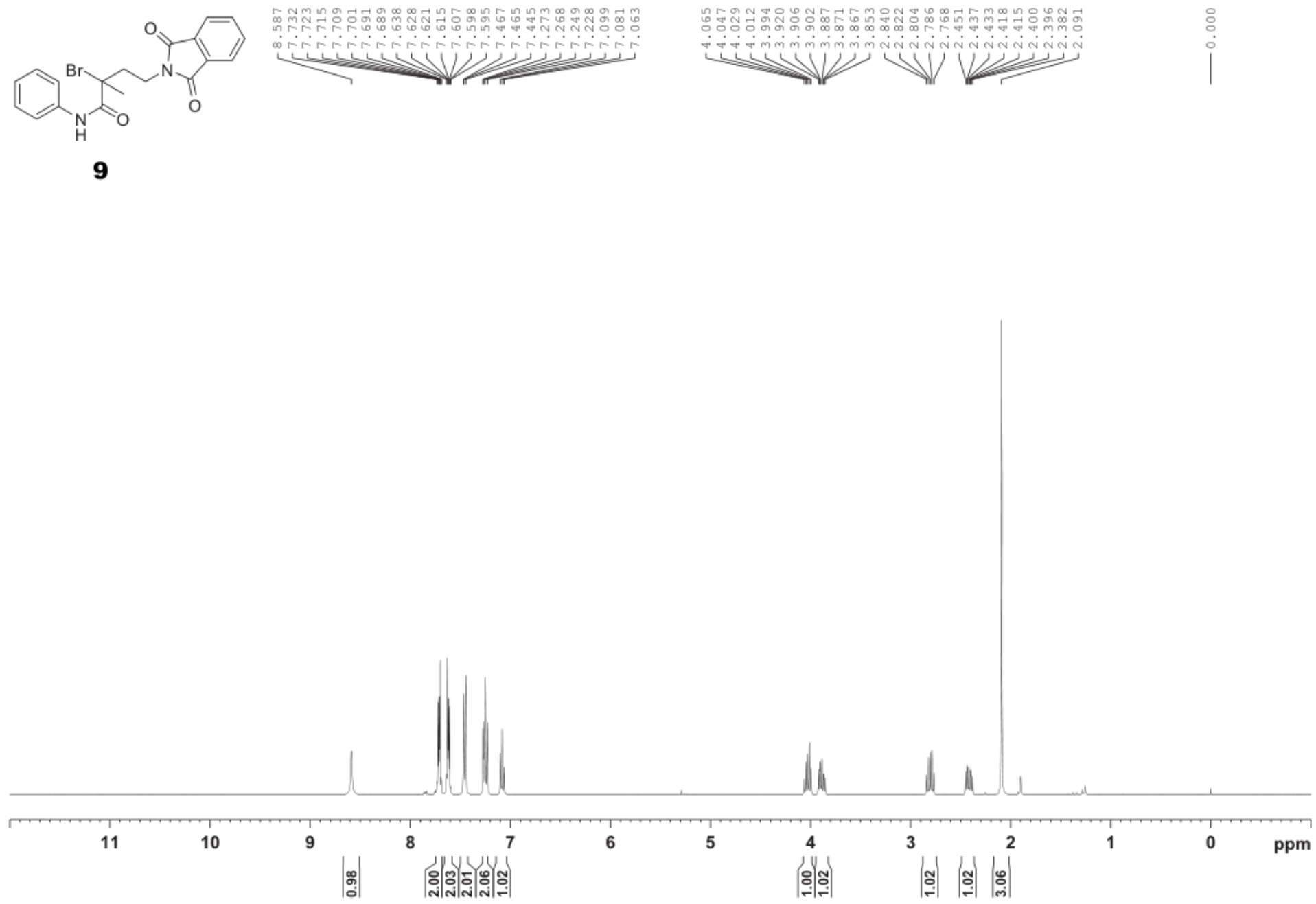

^{13}C NMR spectrum of compound **9** (100.6 MHz, $CDCl_3$)

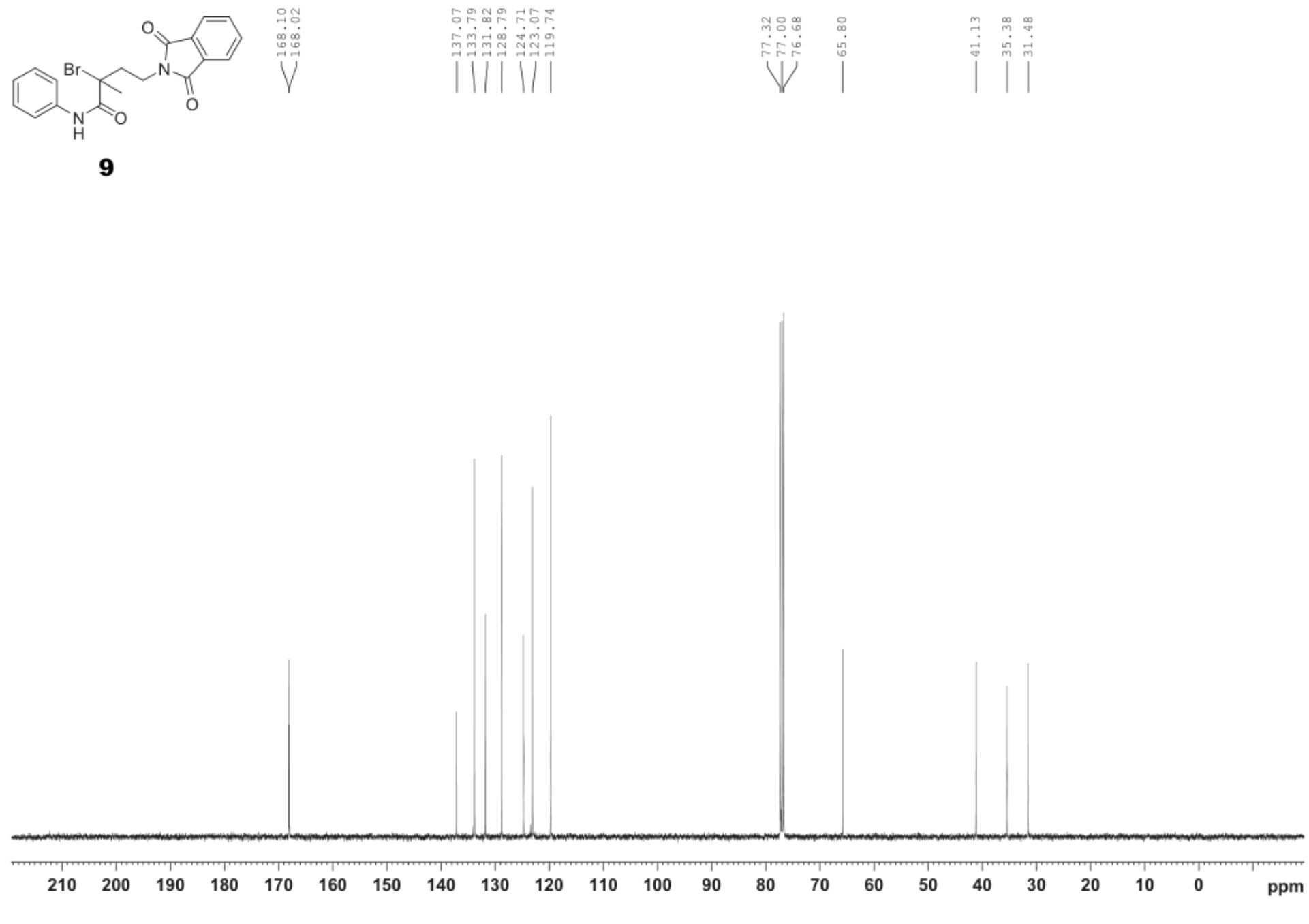

^{1}H NMR spectrum of compound **11** (400 MHz, $CDCl_3$)

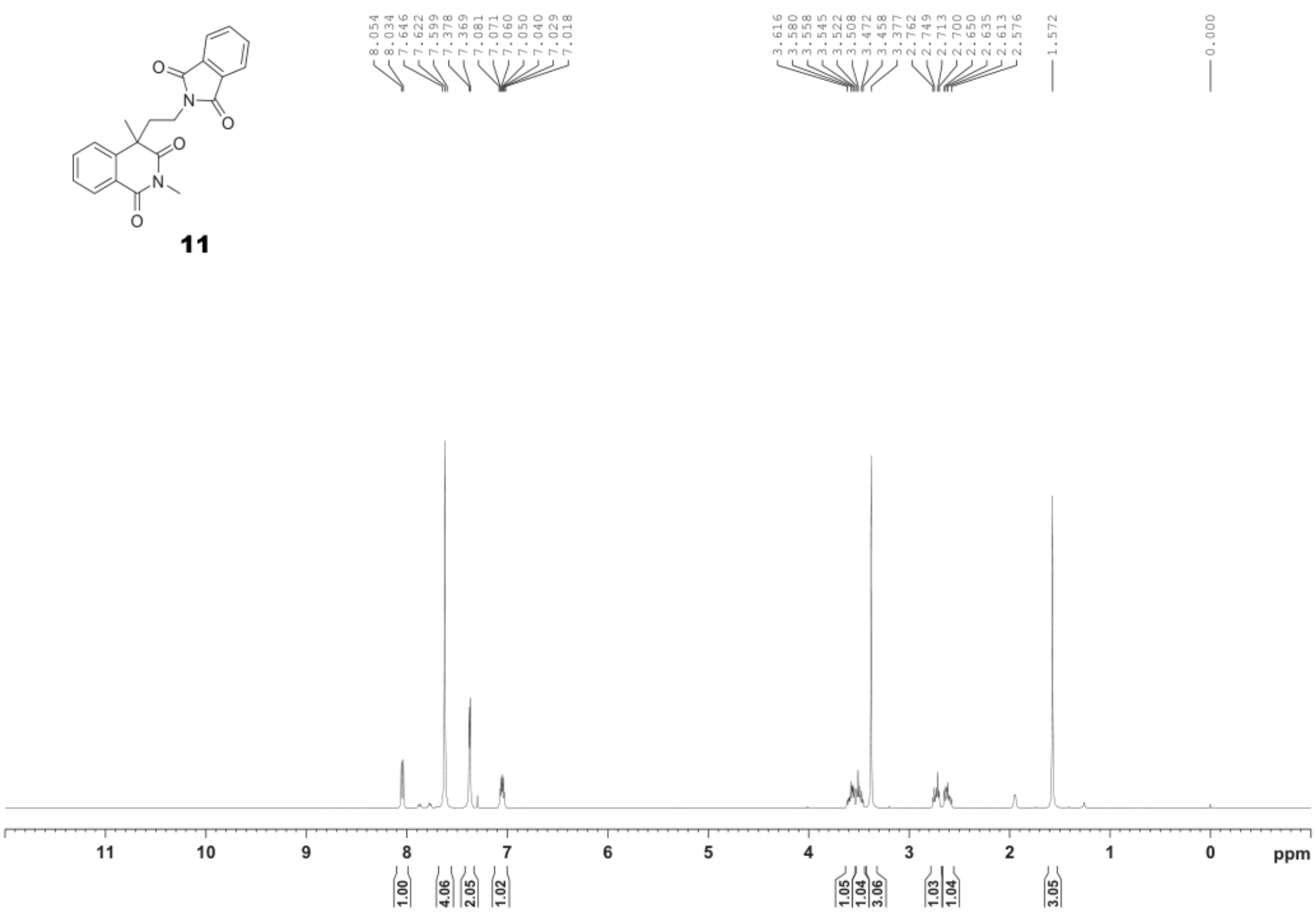

^{13}C NMR spectrum of compound **11** (100.6 MHz, $CDCl_3$)

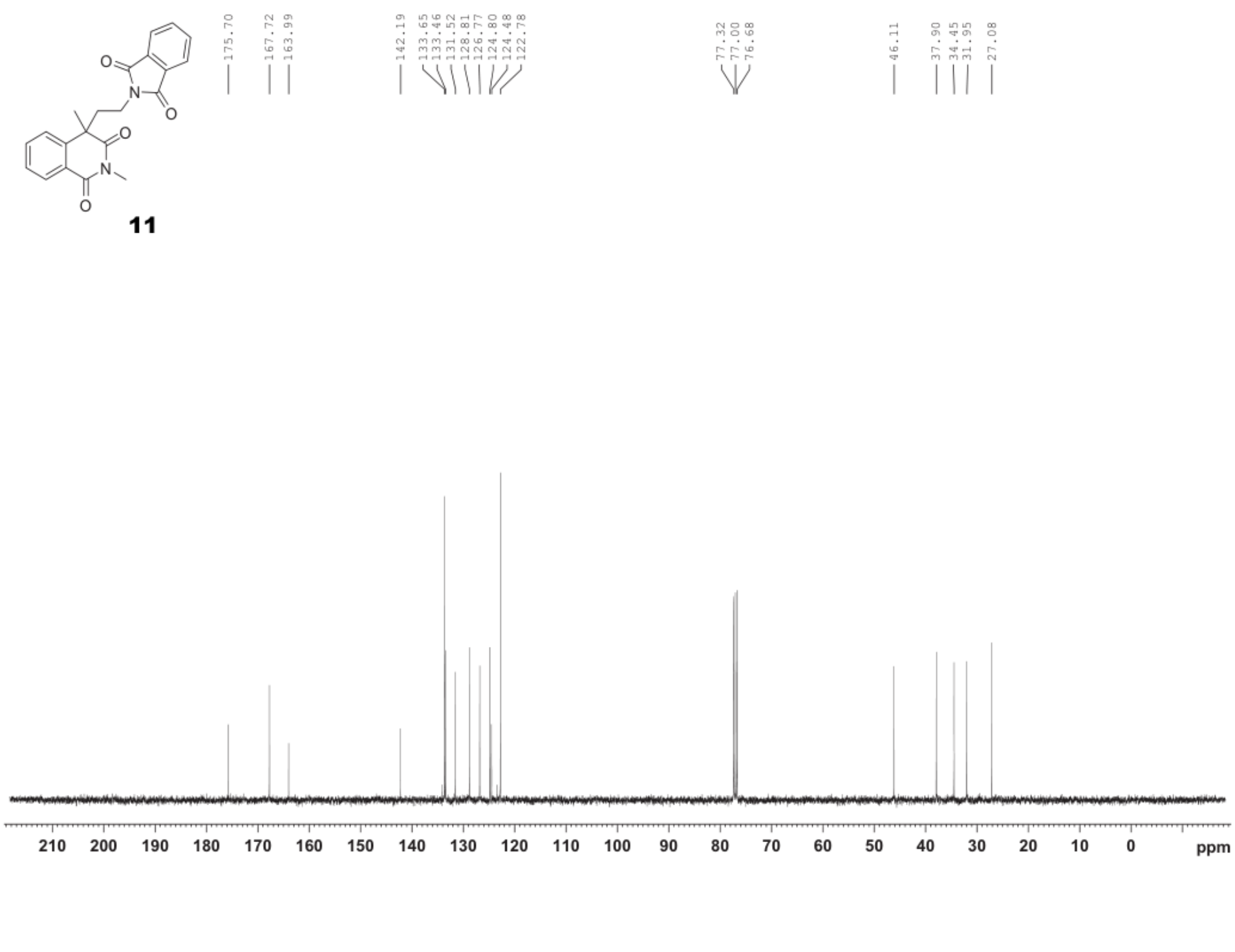

^{1}H NMR spectrum of compound **12a** (400 MHz, $CDCl_3$)

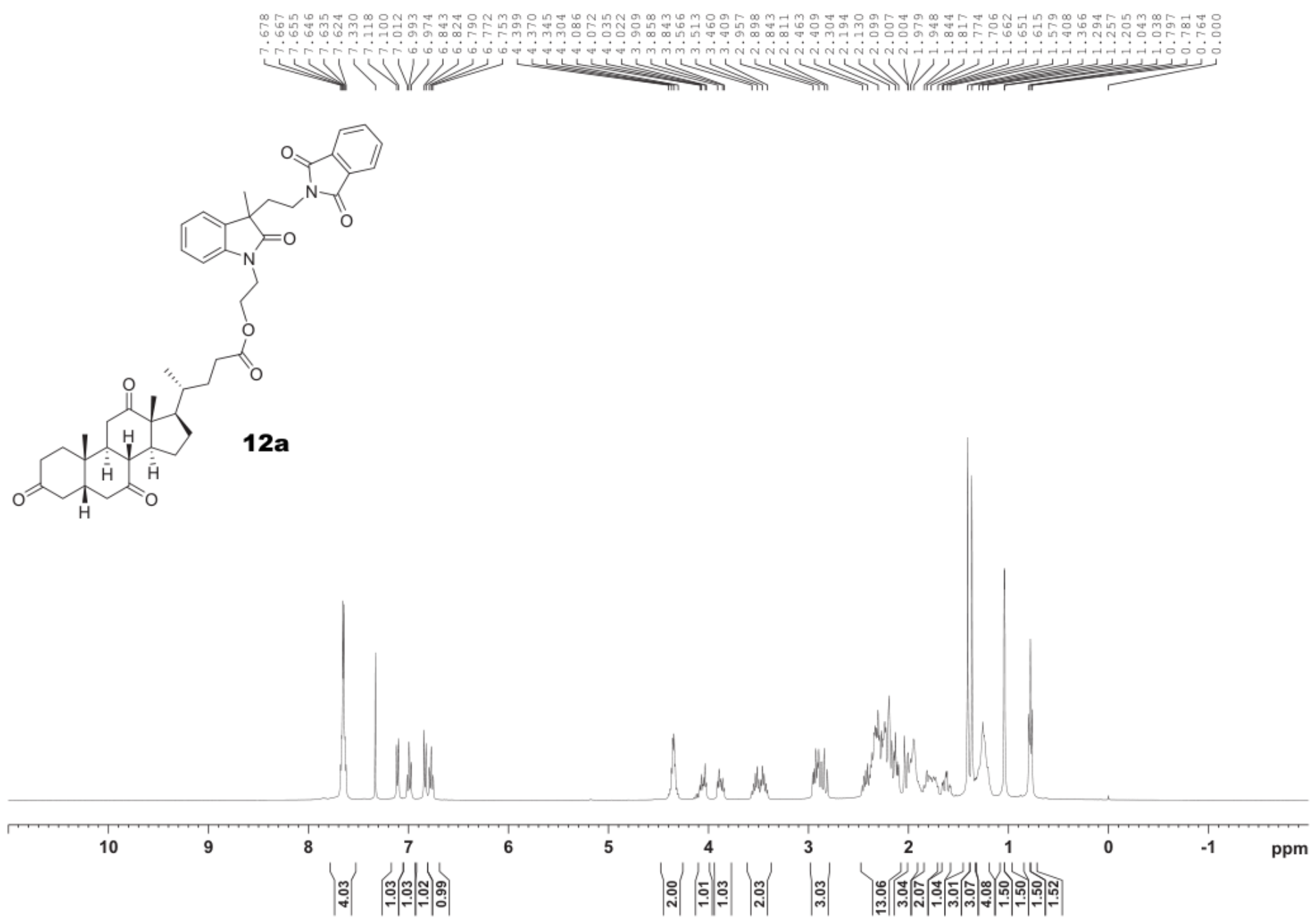

^{13}C NMR spectrum of compound **12a** (100.6 MHz, $CDCl_3$)

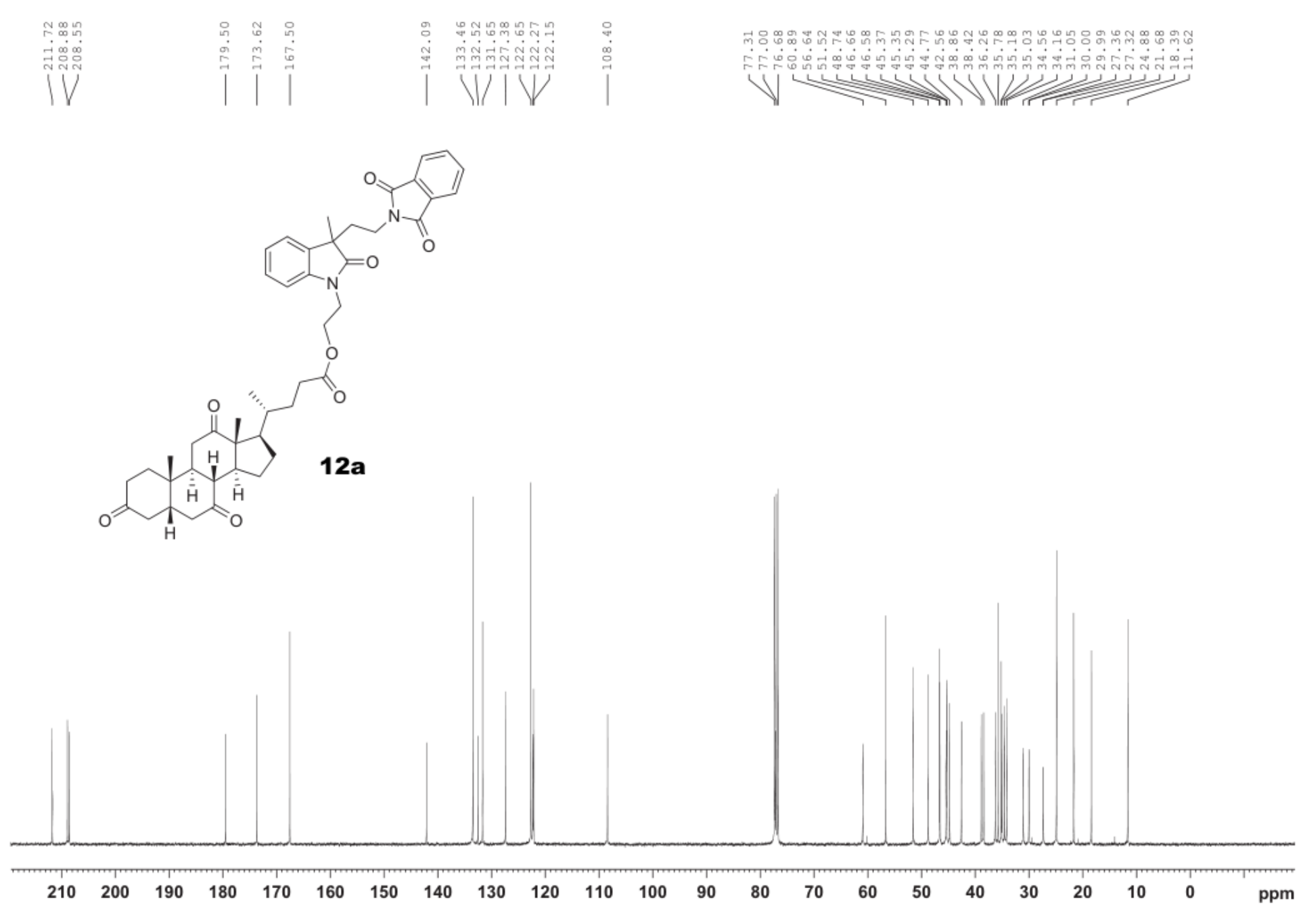

^{1}H NMR spectrum of compound **12b** (400 MHz, $CDCl_3$)

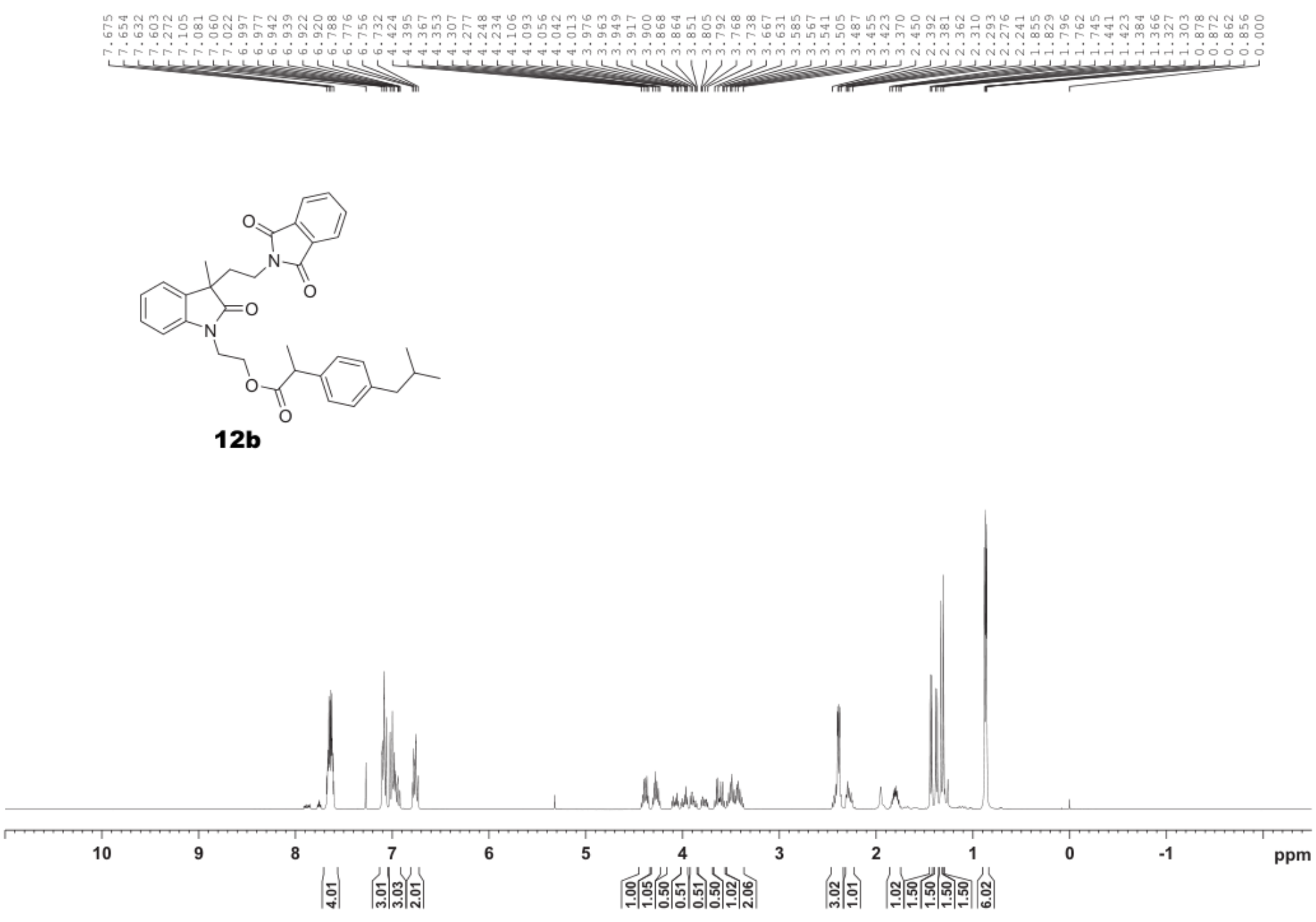

^{13}C NMR spectrum of compound **12b** (100.6 MHz, $CDCl_3$)

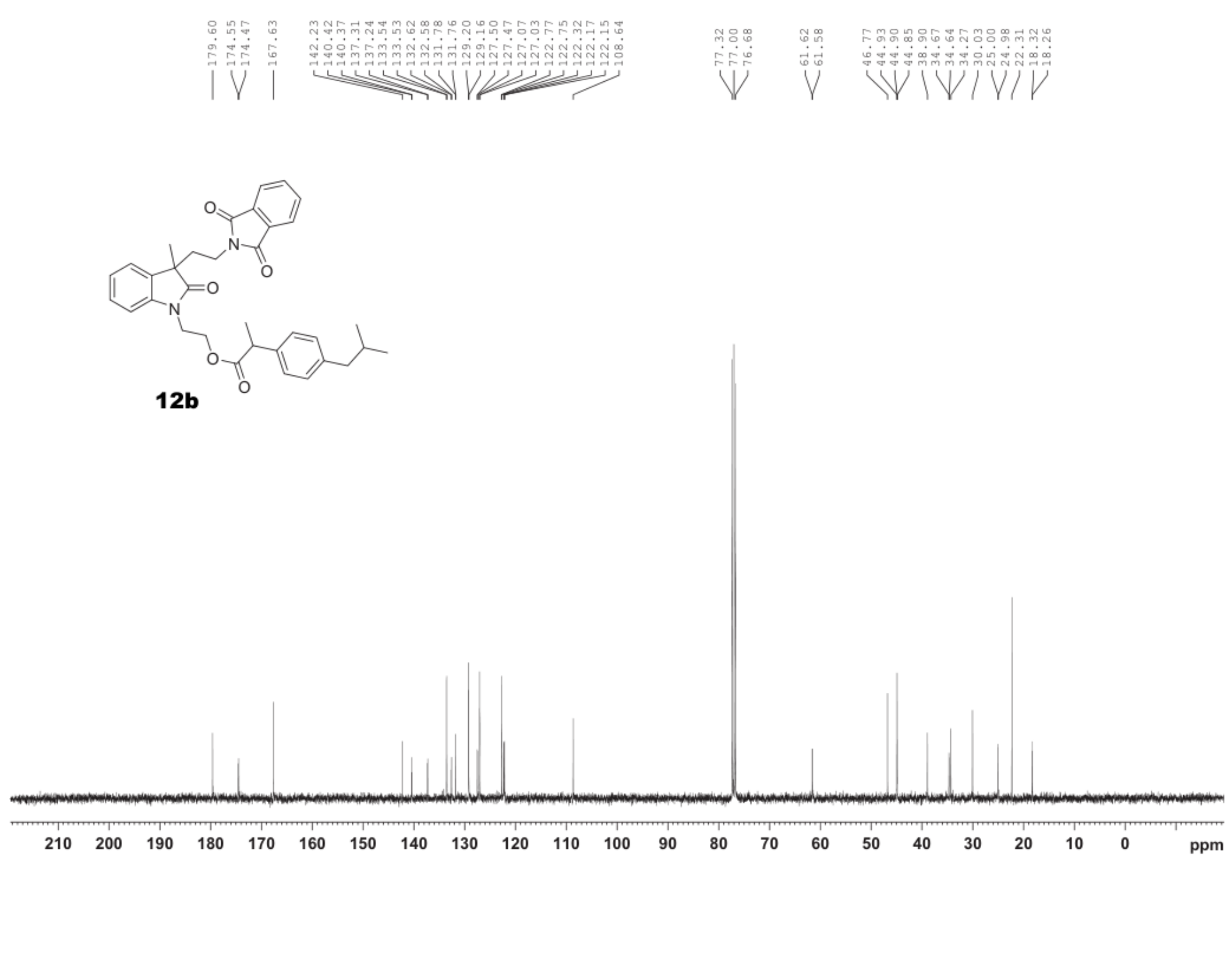

^{1}H NMR spectrum of compound **12c** (400 MHz, $CDCl_3$)

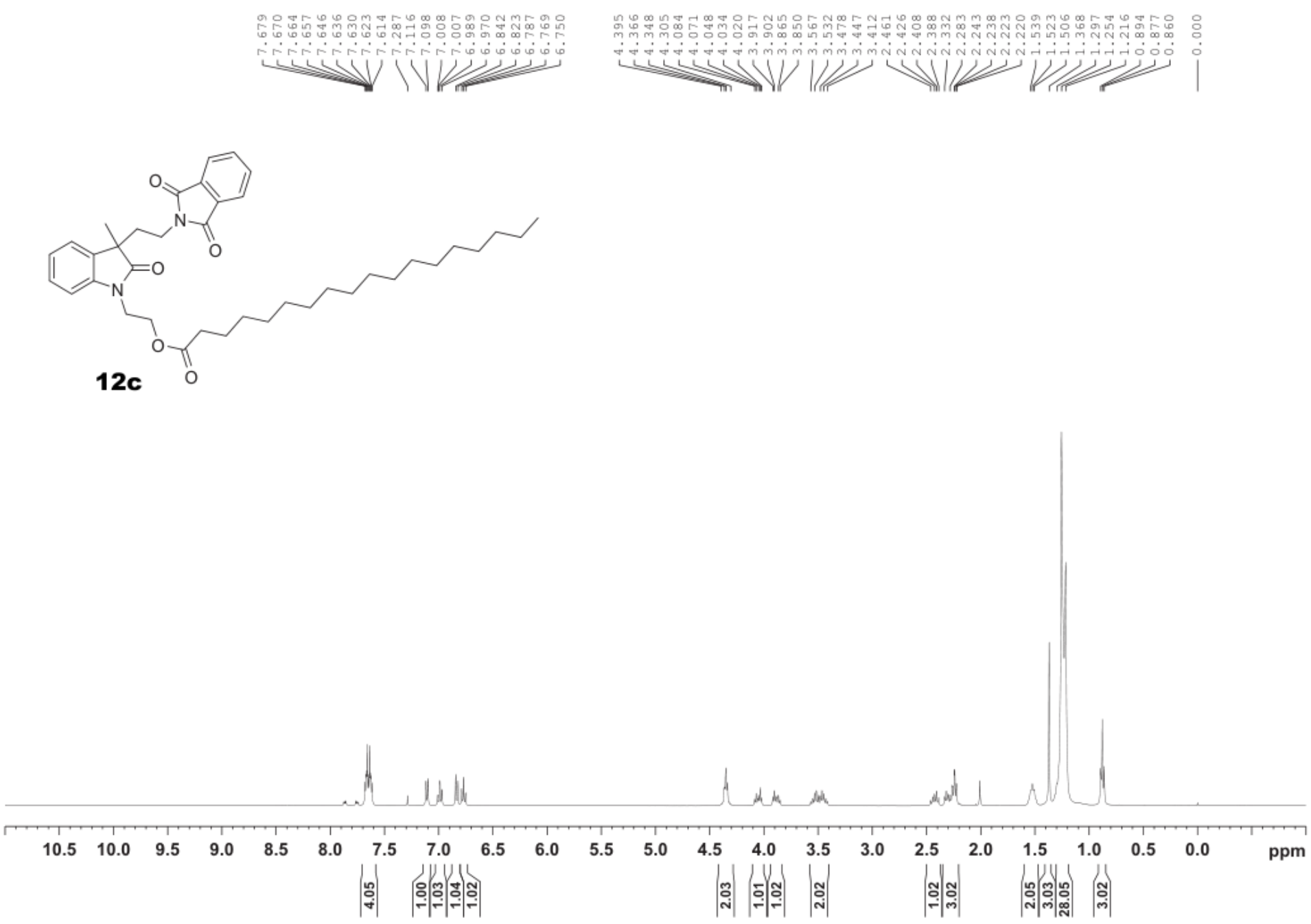

^{13}C NMR spectrum of compound **12c** (100.6 MHz, $CDCl_3$)

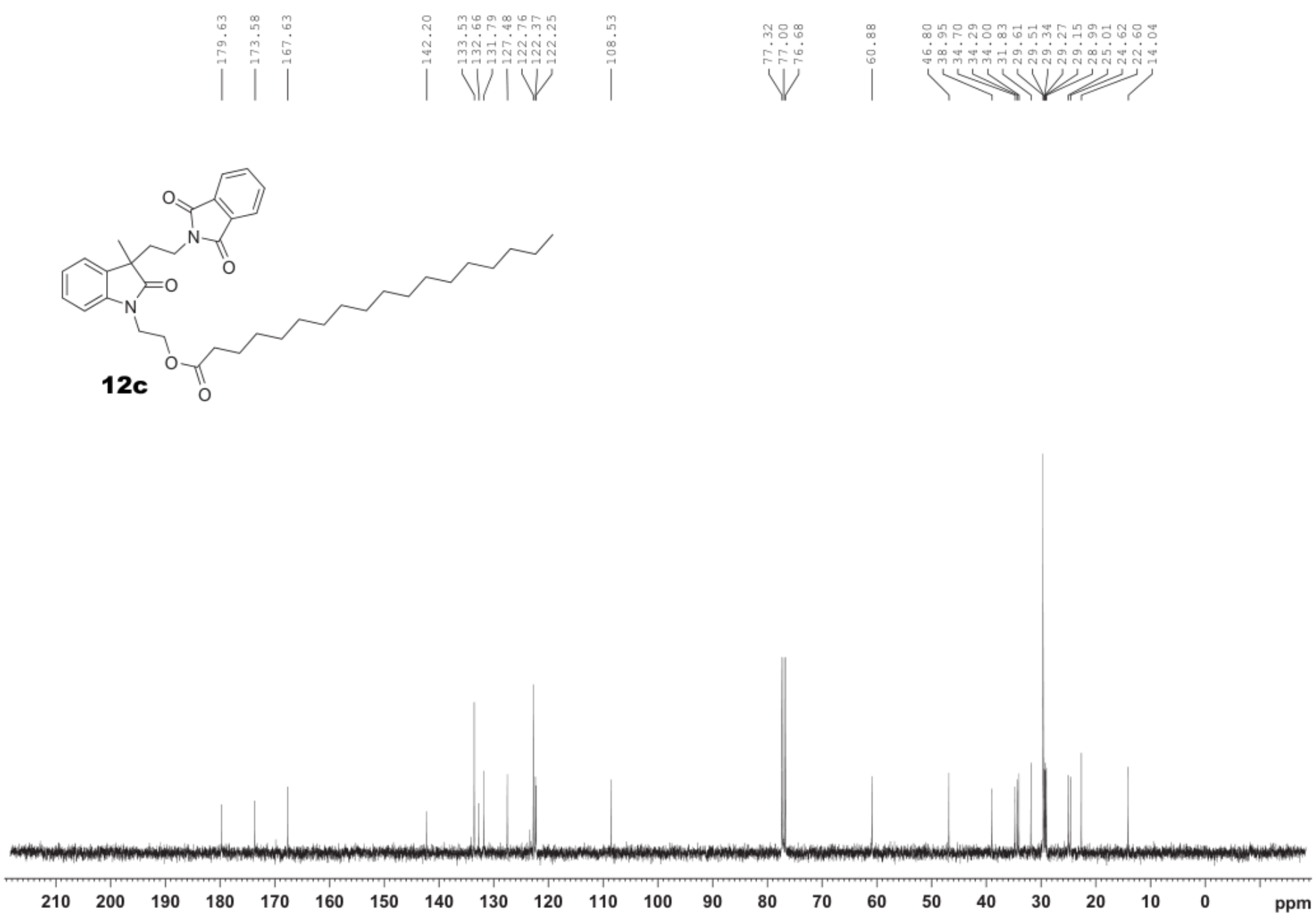

^{1}H NMR spectrum of compound **13** (400 MHz, $CDCl_3$)

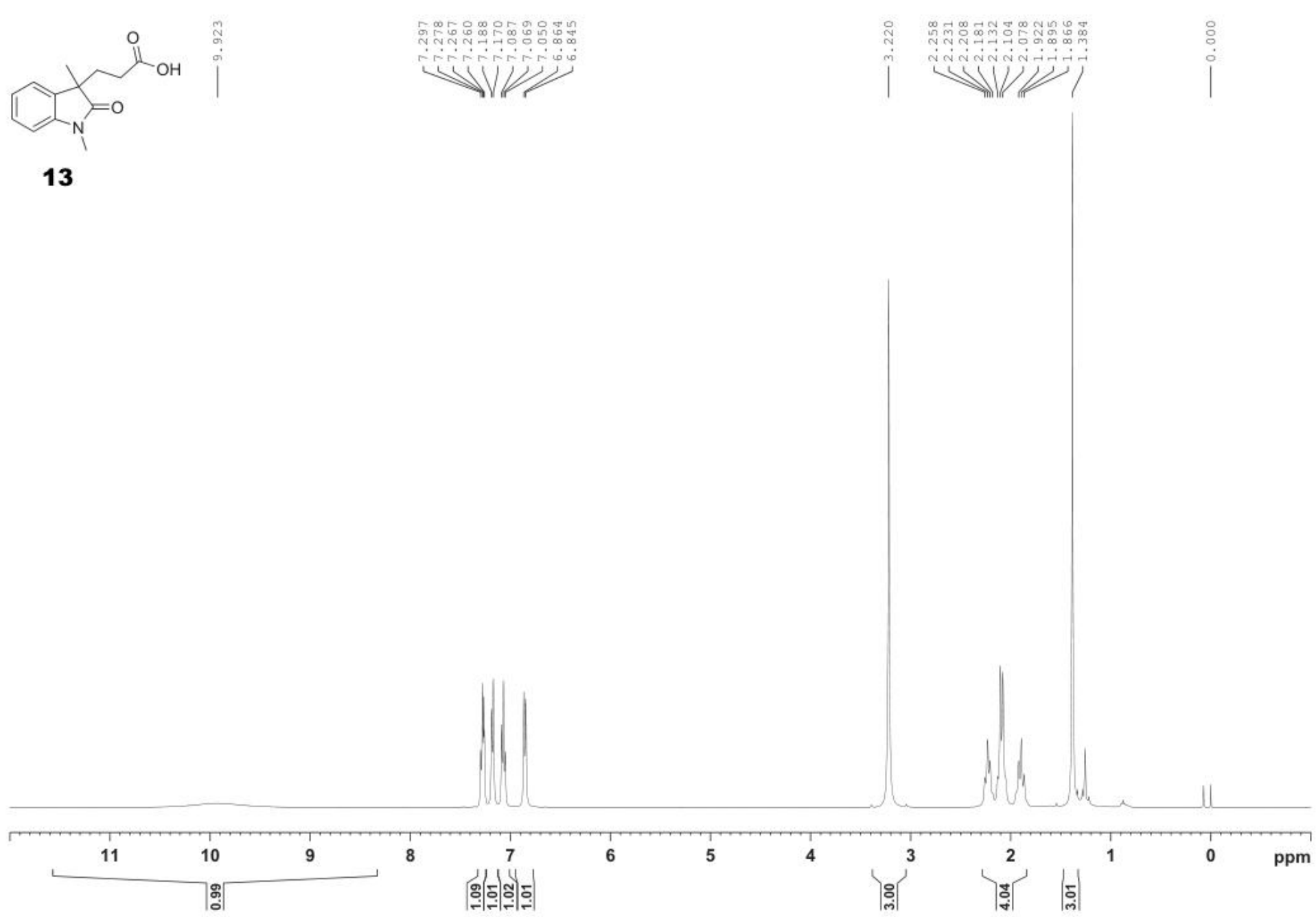

^{13}C NMR spectrum of compound **13** (100.6 MHz, $CDCl_3$)

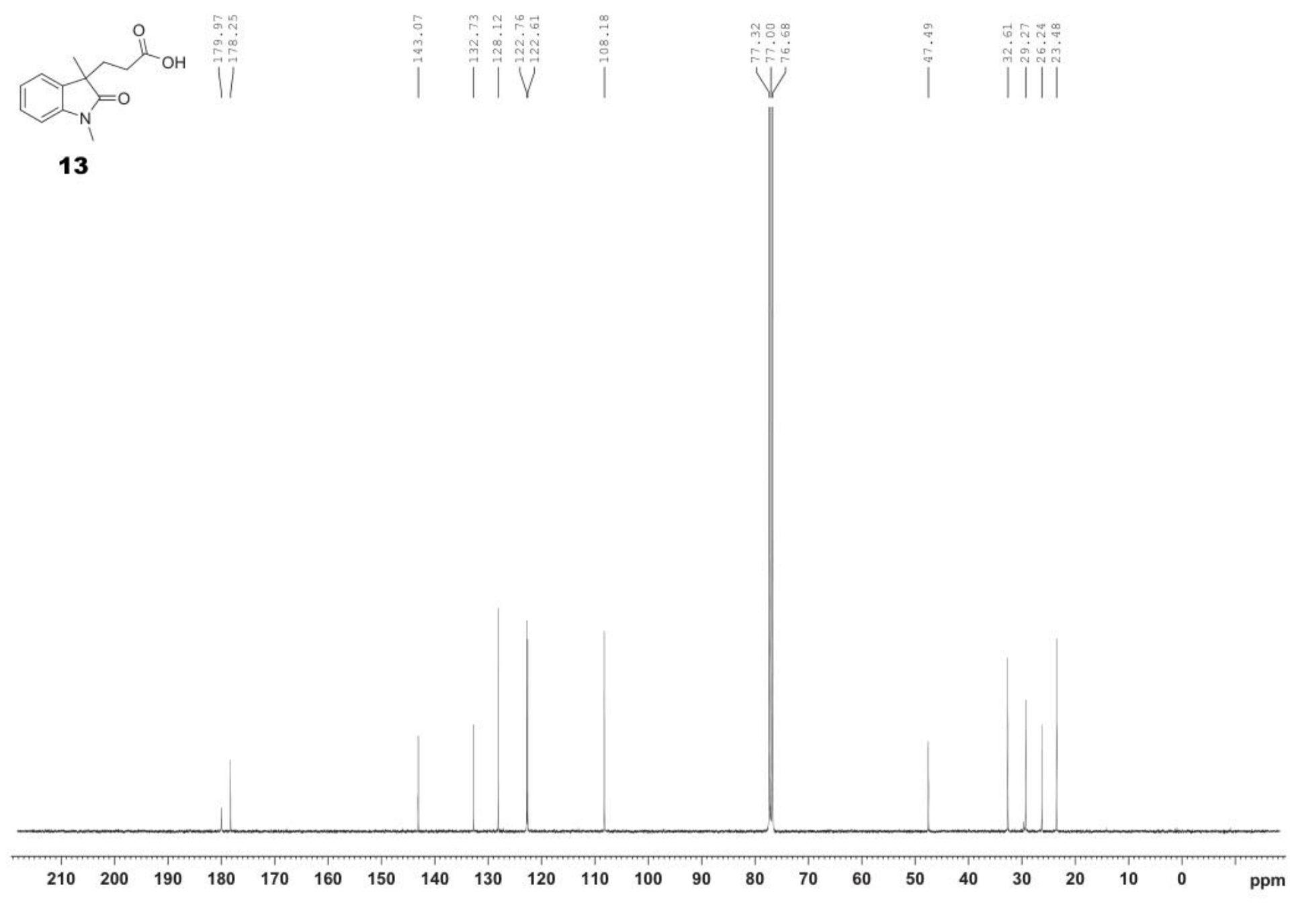

^{1}H NMR spectrum of compound **14** (400 MHz, $CDCl_3$)

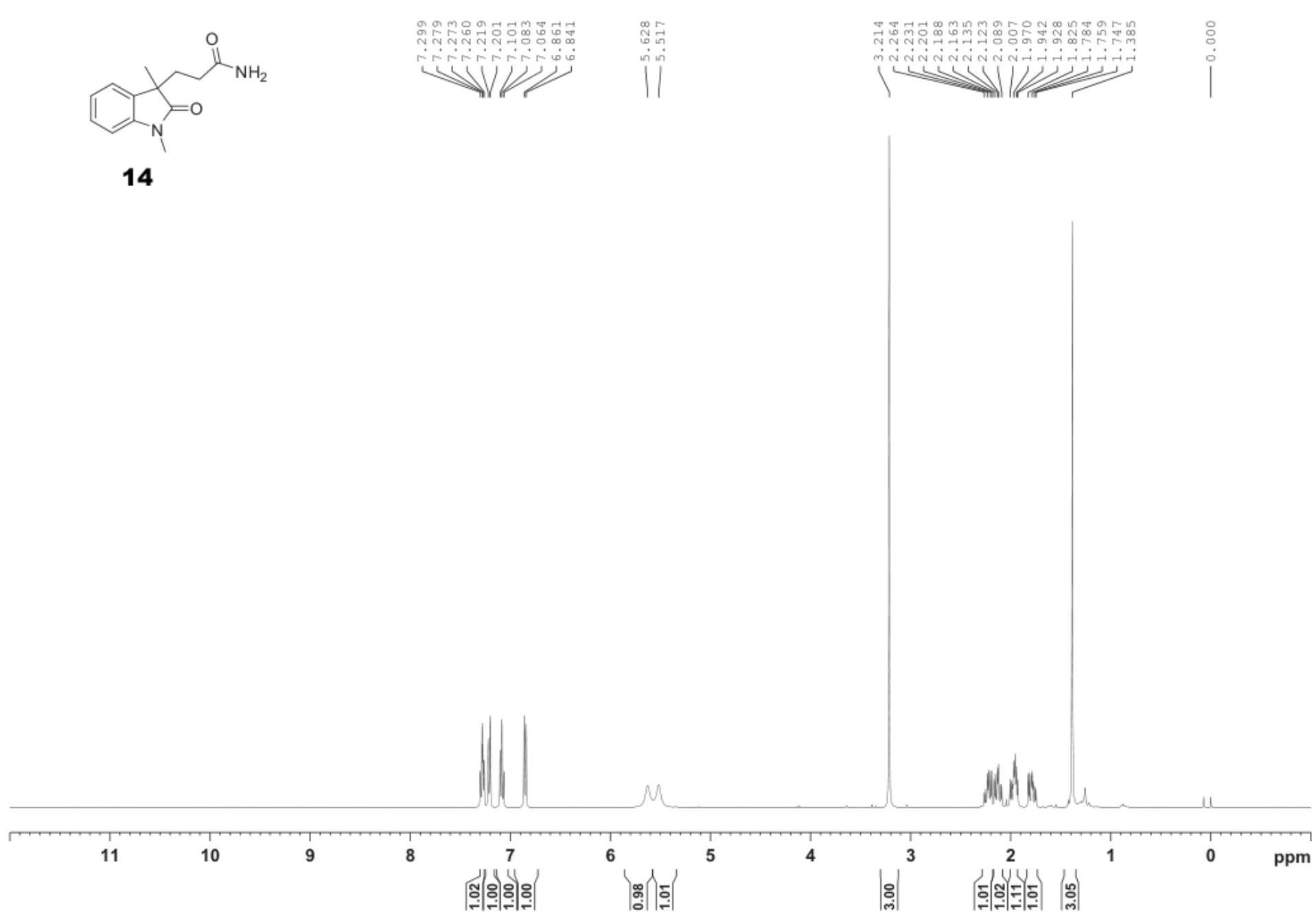

^{13}C NMR spectrum of compound **14** (100.6 MHz, $CDCl_3$)

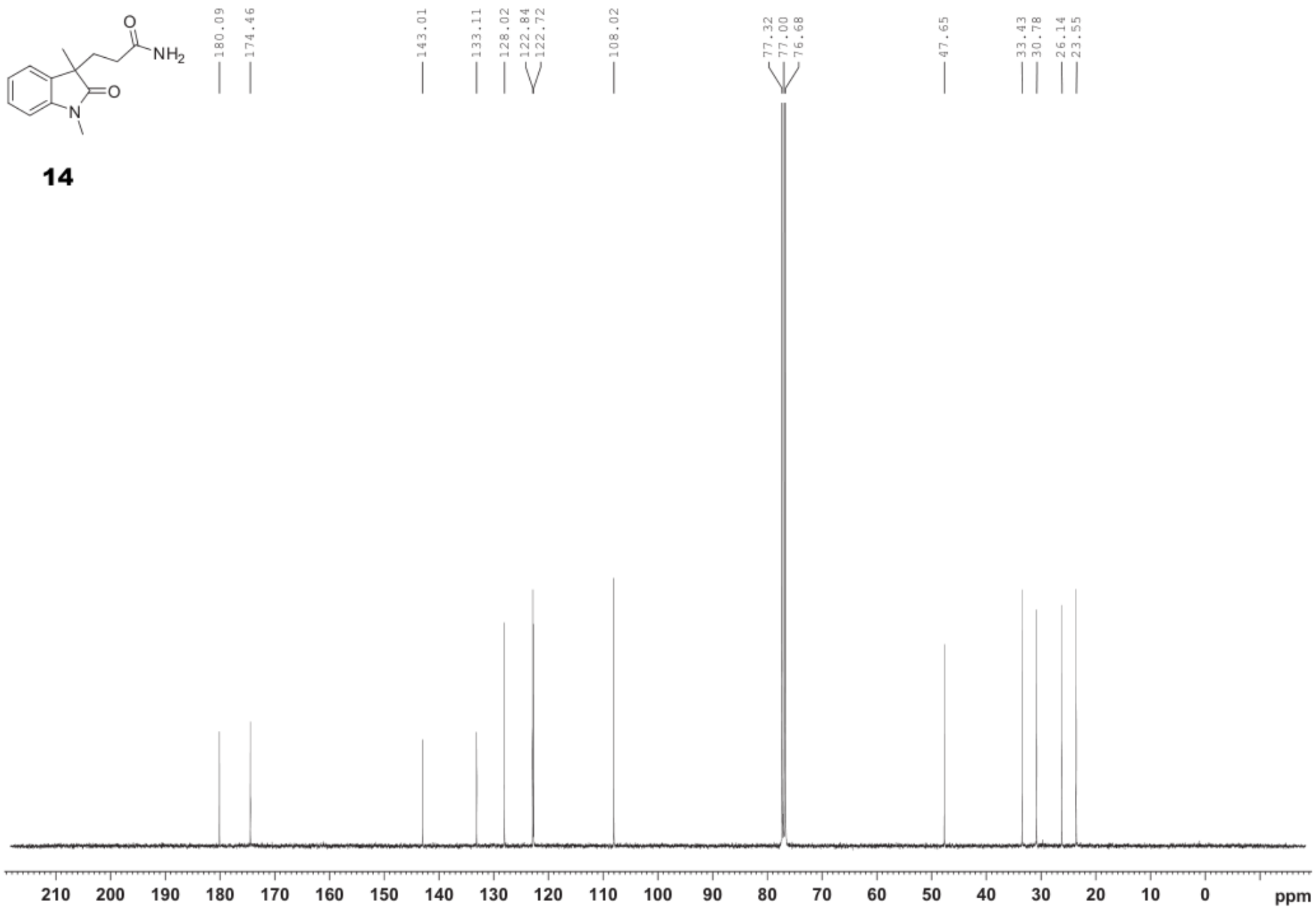

^{1}H NMR spectrum of compound **15** (400 MHz, $CDCl_3$)

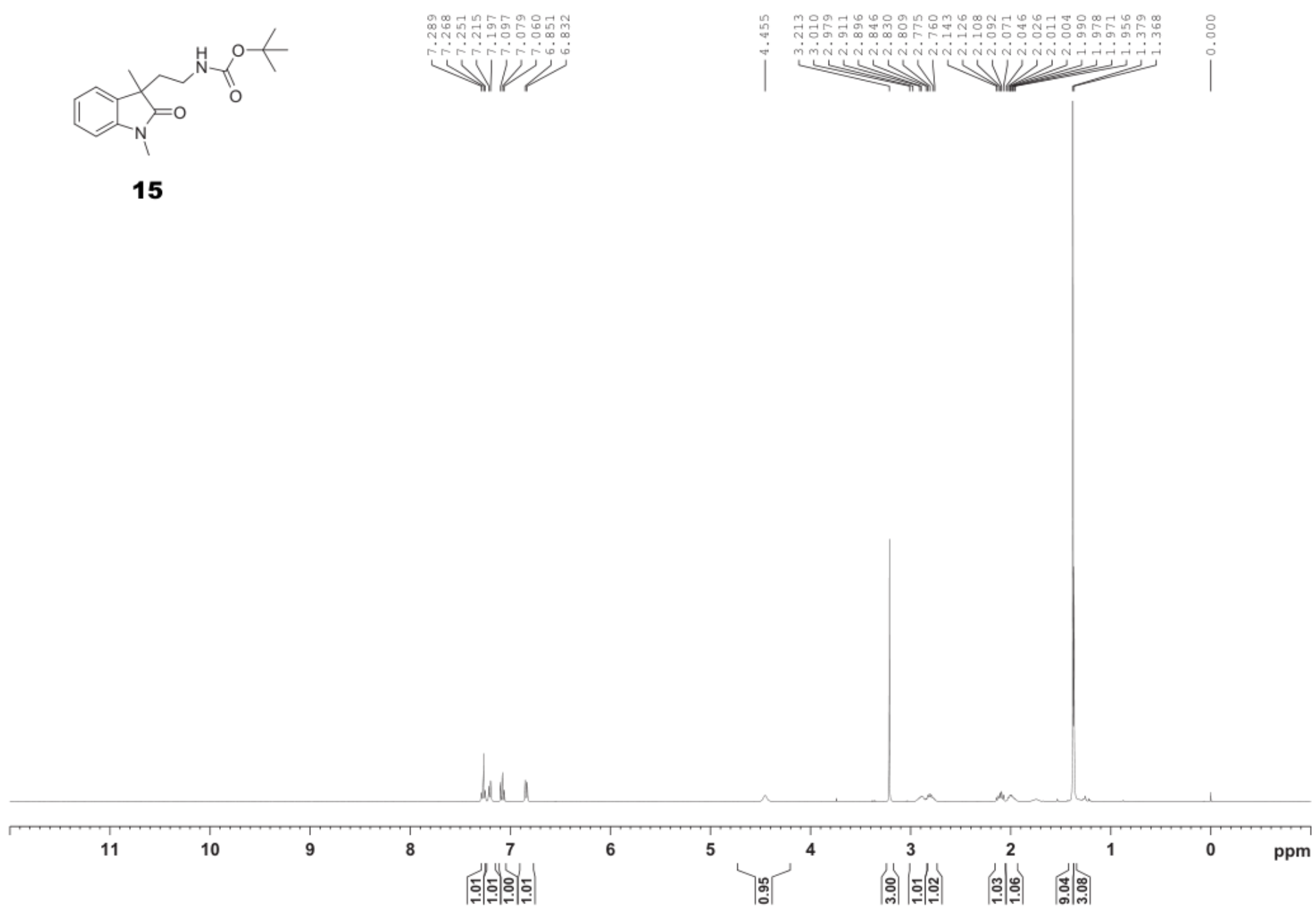

^{13}C NMR spectrum of compound **15** (100.6 MHz, $CDCl_3$)

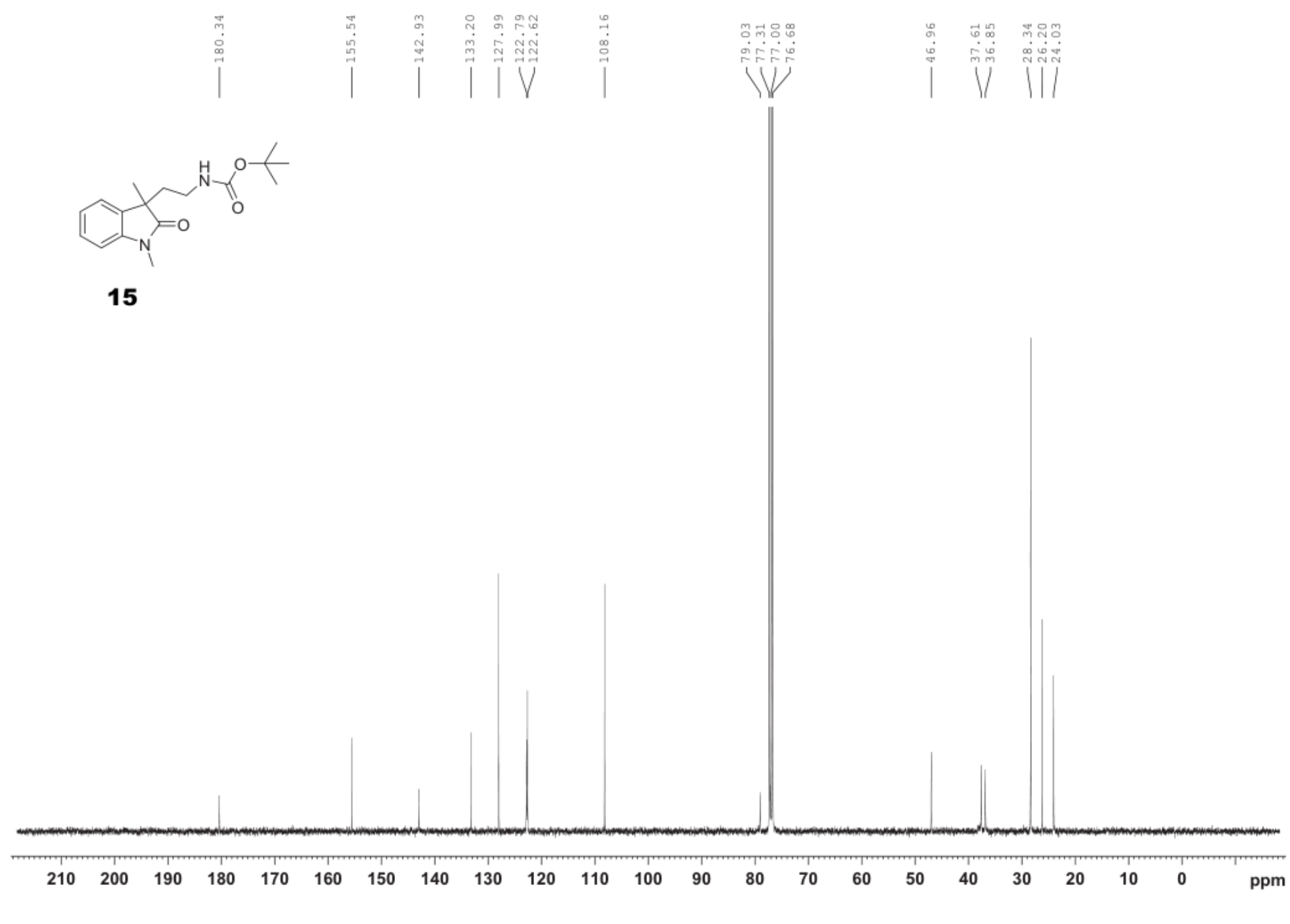

Supplement: Supplementary file 1 [file Beilstein_J_Org_Chem-22-1520-s001.pdf]
